# Tri(*n*-butyl)phosphine-promoted domino reaction for the efficient construction of spiro[cyclohexane-1,3'-indolines] and spiro[indoline-3,2'-furan-3',3''-indolines]

**DOI:** 10.3762/bjoc.18.68

**Published:** 2022-06-14

**Authors:** Hui Zheng, Ying Han, Jing Sun, Chao-Guo Yan

**Affiliations:** 1 College of Chemistry and Chemical Engineering, Yangzhou University, Yangzhou 225002, China

**Keywords:** isatin, spiro[cyclohexane-1,3'-indoline], spiro[indoline-3,2'-furan-3',3''-indoline], spirooxindole, tri(*n*-butyl)phosphine

## Abstract

The tri(*n*-butyl)phosphine-catalyzed reaction of isatylidene malononitriles and bis-chalcones in chloroform at 65 °C afforded functionalized spiro[cyclohexane-1,3'-indolines] in good yields and with good diastereoselectivity. On the other hand, the tri(*n*-butyl)phosphine-catalyzed reaction of 3-(ethoxycarbonylmethylene)oxindoles and bis-chalcones gave functionalized spiro[cyclohexane-1,3'-indolines] with different regioselectivity. Additionally, the tri(*n*-butyl)phosphine-promoted domino annulation reaction of isatins and ethyl isatylidene cyanoacetates produced spiro[indoline-3,2'-furan-3',3''-indolines] in satisfactory yields.

## Introduction

Spirooxindole is a privileged heterocyclic core existing in many natural products and medicinally relevant compounds. A variety of spirooxindole derivatives has been identified with a broad range of biological activities [1–5]. On the other hand, spirooxindoles can be constructed by introduction of various carbocyclic and heterocyclic units, which made them suitable for chemistry and many potential applications [6–9]. The development of unique spirooxindole systems and efficient synthetic methods for these compounds have attracted continual interests in many fields. A literature survey indicated that many convenient and atom-economic synthetic protocols have been developed for the synthesis of diverse spirooxindoles in recent years [10–16].

Nucleophilic tertiary phosphine-catalyzed reactions were successfully applied for the construction of diverse carbocyclic systems [17–24]. In these reactions, the tertiary phosphine firstly adds to electron-deficient alkenes, alkynes, and allenes to give active ionic intermediates. Then, the in situ-generated ionic intermediates further react with various reagents to give versatile cyclic compounds with recovery of the tertiary phosphine [25–34]. The superior catalytic ability of tertiary phosphines was primarily attributed to their excellent nucleophilicity as a nucleophile trigger and decent cleaving ability as a leaving group in the catalytic process [35–43]. The tertiary phosphine-catalyzed reactions have been widely applied to construct diverse spirooxindole systems by using readily available isatins and 3-methyleneoxindoles as key substrates [44–52]. In this respect, we have also developed several domino reactions by employing tertiary phosphine addition to electron-deficient alkynes as key protocol for the construction of diverse polycyclic spirooxindoles [53–59]. In continuation of our aim to explore elegant domino reactions for spiro compounds [60–66] and in order to demonstrate the potential synthetic value of the nucleophilic phosphine-catalyzed annulation reaction, herein we wish to report the tri(*n*-butyl)phosphine-catalyzed reaction of isatylidene malononitriles and bis-chalcones for the synthesis of functionalized various spiro[cyclohexane-1,3'-indolines] and related reactions.

## Results and Discussion

Initially, the reaction conditions were optimized by using isatylidene malononitrile **1a** and bis-chalcone **2a** as standard reaction. Tertiary amines such as DMAP and DABCO did not catalyze this reaction (entries 1 and 2 in Table 1). Additionally, no reaction was observed when triphenylphosphine was used as catalyst (Table 1, entry 3). However, in the presence of tri(*n*-butyl)phosphine, the reaction in methylene dichloride, chloroform, and toluene at room temperature gave the expected functionalized spiro[cyclohexane-1,3'-indoline] **3a** albeit with low yields (entries 4–6 in Table 1). The spiro[cyclohexane-1,3'-indoline] **3a** was clearly produced by a tri(*n*-butyl)phosphine-catalyzed formal [4 + 2] cycloaddition reaction. This result showed that tri(*n*-butyl)phosphine has a higher nucleophilic catalytic ability than triphenylphosphine. It should be pointed out that the synthesis of polysubstituted cyclohexanones was reported by a tri(*n*-butyl)phosphine-catalyzed reaction of 1,4-dien-3-ones with 2-aryl-1,1-dicyanoalkenes [36]. At higher temperature (50 °C), the yield of the product **3a** increased to 40% (in CH_2_Cl_2_), 65% (in toluene), and to 75% (in chloroform), respectively, in a Schlenk flask (entries 7–9 in Table 1). Therefore, chloroform was selected as the suitable solvent. The reaction in chloroform at 65 °C for six hours afforded the product **3a** in 84% yield (entry 10, Table 1). By prolonging the reaction time, the yield of **3a** did not increase further (entries 11 and 12, Table 1). At last, when 10% equiv tri(*n*-butyl)phosphine were used, the yield of product **3a** decreased to 65% (entry 13, Table 1), whereas 50% equiv tri(*n*-butyl)phosphine did not improve the yield of **3a** (82% yield, entry 14, Table 1). It should be pointed out that the total yield of the mixed two isomers was calculated in Table 1. Thus, the best results were obtained by carrying out the reaction in chloroform at 65 °C for six hours in the presence of 20% equiv tri(*n*-butyl)phosphine as catalyst.

**Table 1:** Optimization of reaction conditions.^a^

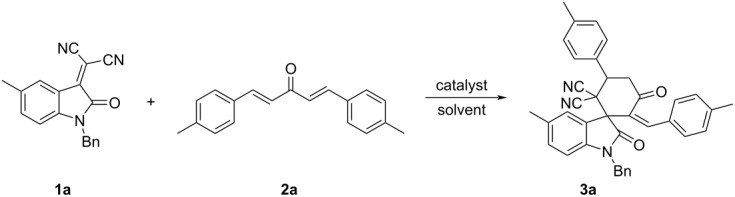					

Entry	Catalyst	Solvent	Temp [°C]	Time [h]	Yield [%]^b^

1	DABCO	CHCl_3_	rt	24	–
2	DMAP	CHCl_3_	rt	24	–
3	PPh_3_	CHCl_3_	rt	24	–
4	P(*n*-Bu)_3_	CH_2_Cl_2_	rt	24	30
5	P(*n*-Bu)_3_	PhMe	rt	24	35
6	P(*n*-Bu)_3_	CHCl_3_	rt	24	45
7	P(*n*-Bu)_3_	CH_2_Cl_2_	50	24	40
8	P(*n*-Bu)_3_	PhMe	50	24	65
9	P(*n*-Bu)_3_	CHCl_3_	50	24	75
**10**	P(*n*-Bu)_3_	**CHCl** **_3_**	**65**	**6**	**84**
11	P(*n*-Bu)_3_	CHCl_3_	65	12	82
12	P(*n*-Bu)_3_	CHCl_3_	65	24	80
13	P(*n*-Bu)_3_^c^	CHCl_3_	65	24	65
14	P(*n*-Bu)_3_^d^	CHCl_3_	65	6	82

^a^Reaction conditions: isatylidene malononitrile (0.5 mmol), bis-chalcone (0.6 mmol), catalyst (20% equiv), solvent (10 mL), N_2_ atmosphere in Schlenk tube; ^b^isolated yields of the mixed diastereoisomers; ^c^PBu_3_ (10% equiv) was used; ^d^PBu_3_ (50% equiv) was used.

**Figure 1:**
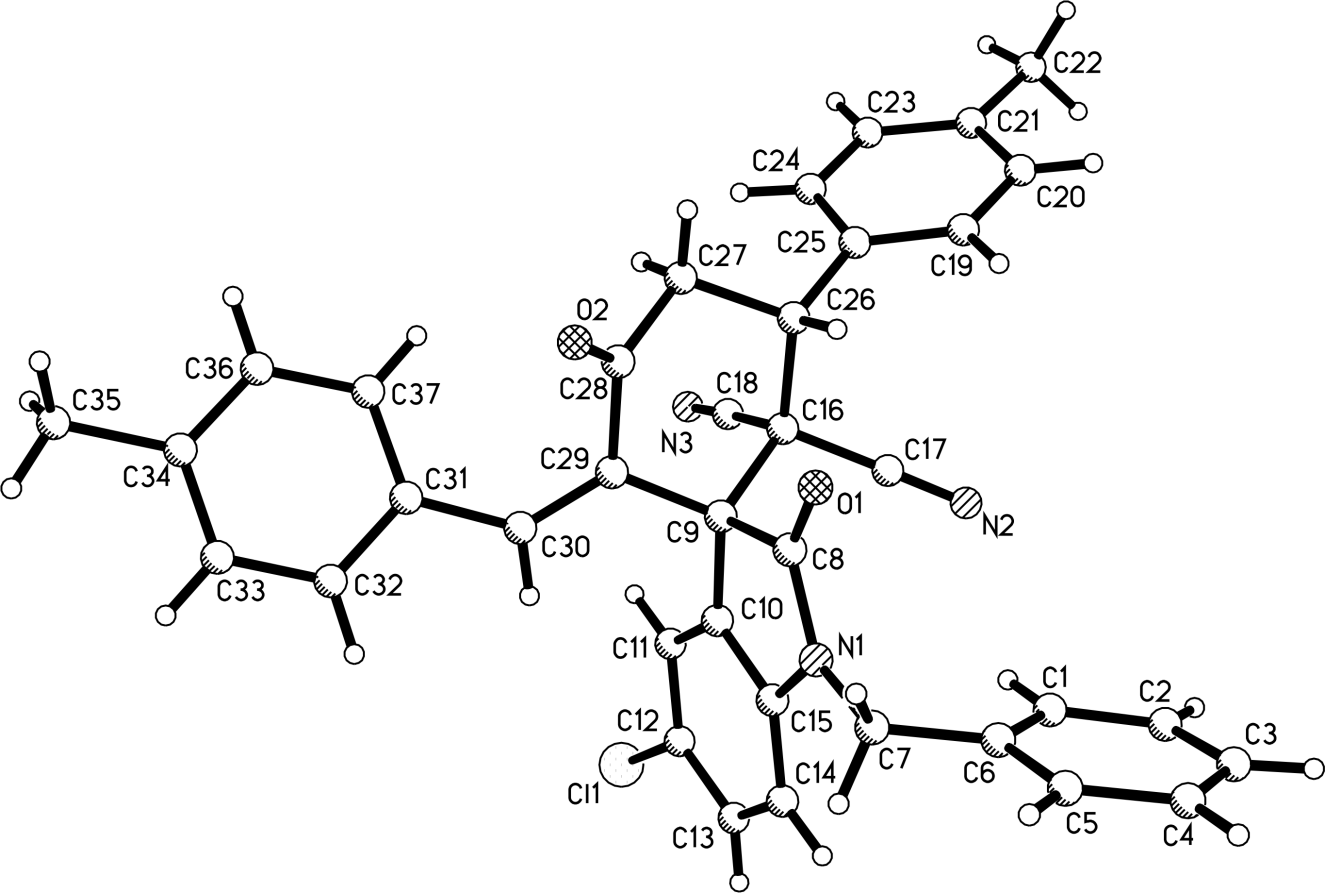
Single crystal structure of compound **3l**.

**Figure 2:**
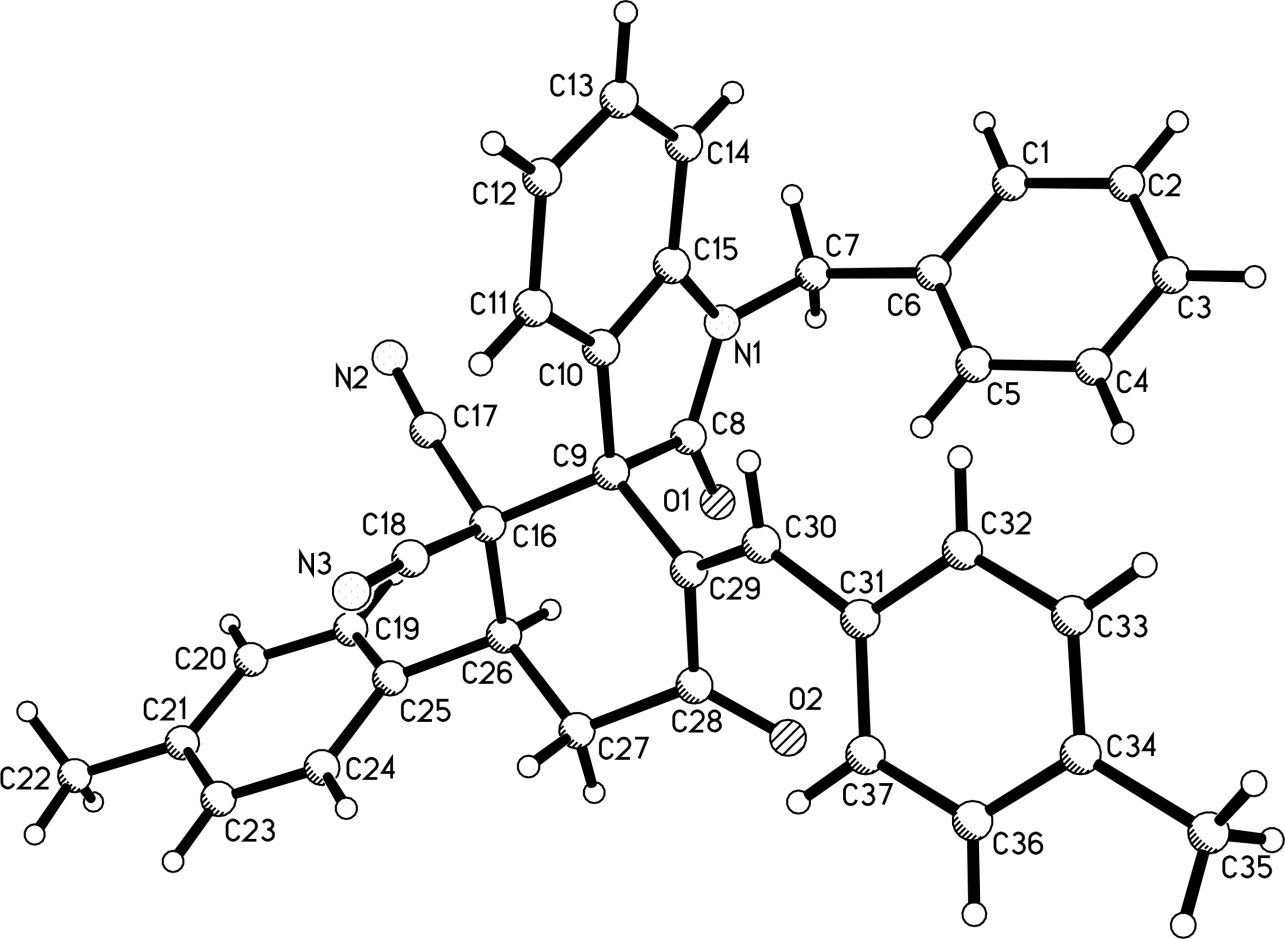
Single crystal structure of compound **3s**.

**Figure 3:**
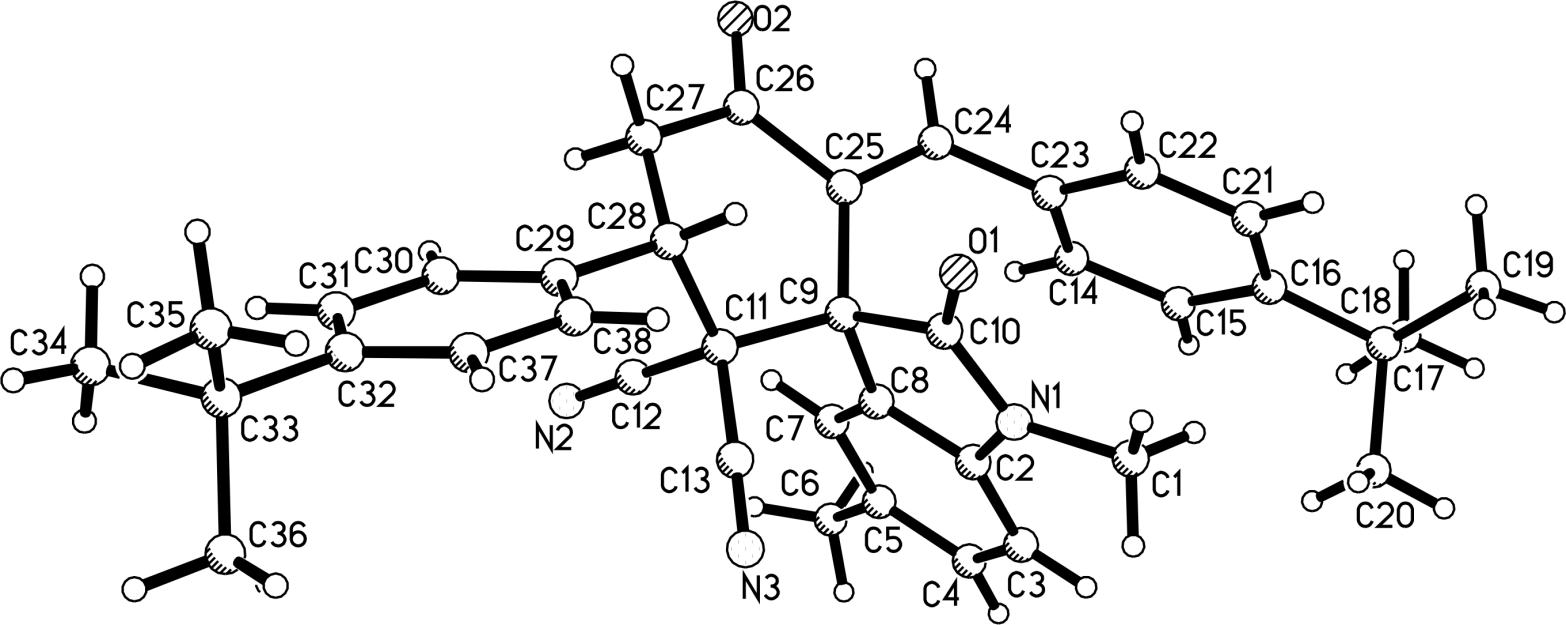
Single crystal structure of compound **3f’**.

With the best reaction conditions in hand, the scope of the reaction was developed by using various substrates and the results are summarized in Table 2. It was found that all reactions proceeded smoothly to give the expected spiro[cyclohexane-1,3'-indolines] **3a**–**z** in moderate to good yields. The isatylidene malononitriles with different substituents at the C5-position and C1-position can be successfully employed in the reaction. The reaction with bis-chalcones with electron-donating groups gave slightly higher yields of the products than that of bis-chalcones with electron-withdrawing groups. Because there are two chiral carbon atoms in the spiro[cyclohexane-1,3'-indolines] and the *Z*/*E*-configuration of the exocyclic C–C double bonds, several diastereoisomers might be formed in the obtained products. The ^1^H NMR spectra clearly indicated that the obtained products contain two isomers with ratios in the range of 3:1 to 6:1. Thus, it was disappointing to find that the diastereoselectivity of this reaction was not very good. Because the polarity of the two diastereoisomers were very similar, it was very difficult to isolate them as pure compounds by column chromatography. For convenience, only the pure major diastereoisomers of the spiro compounds **3a**–**w** were successfully isolated and fully characterized, which also caused the isolated yields of the products slightly decreased. For comparison, the minor isomers **3f’** and **3o’** were also successfully separated and obtained as pure compounds and were fully characterized, respectively. Additionally, 1,5-di(thiophen-2-yl)penta-1,4-dien-3-one was also successfully employed in the reaction. The major isomers of the corresponding spiro compounds **3x** and **3y** were predominately produced in moderate yields. In order to determine the relative configuration of the spiro compounds, the single crystal structures of the major isomers **3l** (Figure 1), **3s** (Figure 2), and **3f’** (Figure 3) were determined by X-ray diffraction analysis. As can be seen from Figure 1 and Figure 2, the aryl group exists in the *trans*-position of the carbonyl group of the oxindole scaffold in the newly formed cyclohexyl ring. On the other hand, the aryl group exist on the *cis*-position of the carbonyl group on the exocyclic C=C bond (*Z*-configuration). Thus, it can be concluded that the major isomers **3a**–**z** have this kind of the relative configuration. In Figure 3, the aryl group and the carbonyl group of the oxindole scaffold also exist on *trans-*position in the newly formed cyclohexyl ring, while an *E*-configuration was observed for the exocyclic C=C double bond. Therefore, the major and minor isomers were actually attributable to the *Z*/*E*-configuration of the C=C bond, not to the *cis/trans*-positions in the newly formed cyclohexyl ring.

**Table 2:** Reaction of isatylidene malononitriles and bis-chalcones.^a^

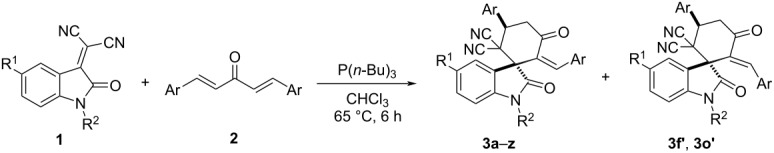						

Entry	Compound	R^1^	R^2^	Ar	Yield [%]^b^	*Z*/*E*^c^

1	**3a**	CH_3_	Bn	*p*-CH_3_C_6_H_4_	67	4:1
2	**3b**	CH_3_	Bn	*p-t*-BuC_6_H_4_	64	4:1
3	**3c**	CH_3_	Bn	C_6_H_5_	64	4:1
4	**3d**	CH_3_	Bn	*p*-ClC_6_H_4_	58	5:1
5	**3e**	CH_3_	Bn	*p*-BrC_6_H_4_	62	5:1
6	**3f** (**3f’**)^d^	CH_3_	CH_3_	*p-t*-BuC_6_H_4_	42 (14)	3:1
7	**3g**	CH_3_	H	*p*-CH_3_C_6_H_4_	45	3:1
8	**3h**	CH_3_	H	*p-t*-BuC_6_H_4_	49	3:1
9	**3i**	CH_3_	*n*-Bu	*p*-BrC_6_H_4_	54	5:1
10	**3j**	CH_3_	*n*-Bu	*p*-CH_3_C_6_H_4_	54	4:1
11	**3k**	CH_3_	*n*-Bu	*p-t*-BuC_6_H_4_	58	5:1
12	**3l**	Cl	Bn	*p*-CH_3_C_6_H_4_	54	5:1
13	**3m**	Cl	Bn	*p-t*-BuC_6_H_4_	60	5:1
14	**3n**	Cl	Bn	*p-*iPrC_6_H_4_	56	4:1
15	**3o** (**3o’**)^d^	Cl	*n*-Bu	*p*-CH_3_C_6_H_4_	42 (8)	5:1
16	**3p**	F	Bn	*p*-CH_3_C_6_H_4_	53	4:1
17	**3q**	F	Bn	*p-*iPrC_6_H_4_	58	4:1
18	**3r**	H	H	*p*-CH_3_C_6_H_4_	48	6:1
19	**3s**	H	Bn	*p*-CH_3_C_6_H_4_	58	4:1
20	**3t**	H	Bn	*p-*iPrC_6_H_4_	57	5:1
21	**3u**	H	Bn	C_6_H_5_	42	4:1
22	**3v**	H	Bn	*p*-ClC_6_H_4_	45	4:1
23	**3w**	H	Bn	*p*-BrC_6_H_4_	47	5:1
24	**3x**	CH_3_	CH_3_	2-thiophenyl	52	trace
25	**3y**	Cl	CH_3_	2-thiophenyl	45	trace
26	**3z**	H	Bn	2-thiophenyl	53	trace

^a^Reaction conditions: isatylidene malononitrile (0.5 mmol), bis-chalcone (0.6 mmol), P(*n*-Bu)_3_ (20% equiv), CHCl_3_ (10.0 mL), N_2_ atmosphere; ^b^isolated yields; ^c^the Z/E ratio was determined by ^1^H NMR spectroscopy; ^d^the minor isomers **3f’** and **3o’** were isolated.

**Figure 4:**
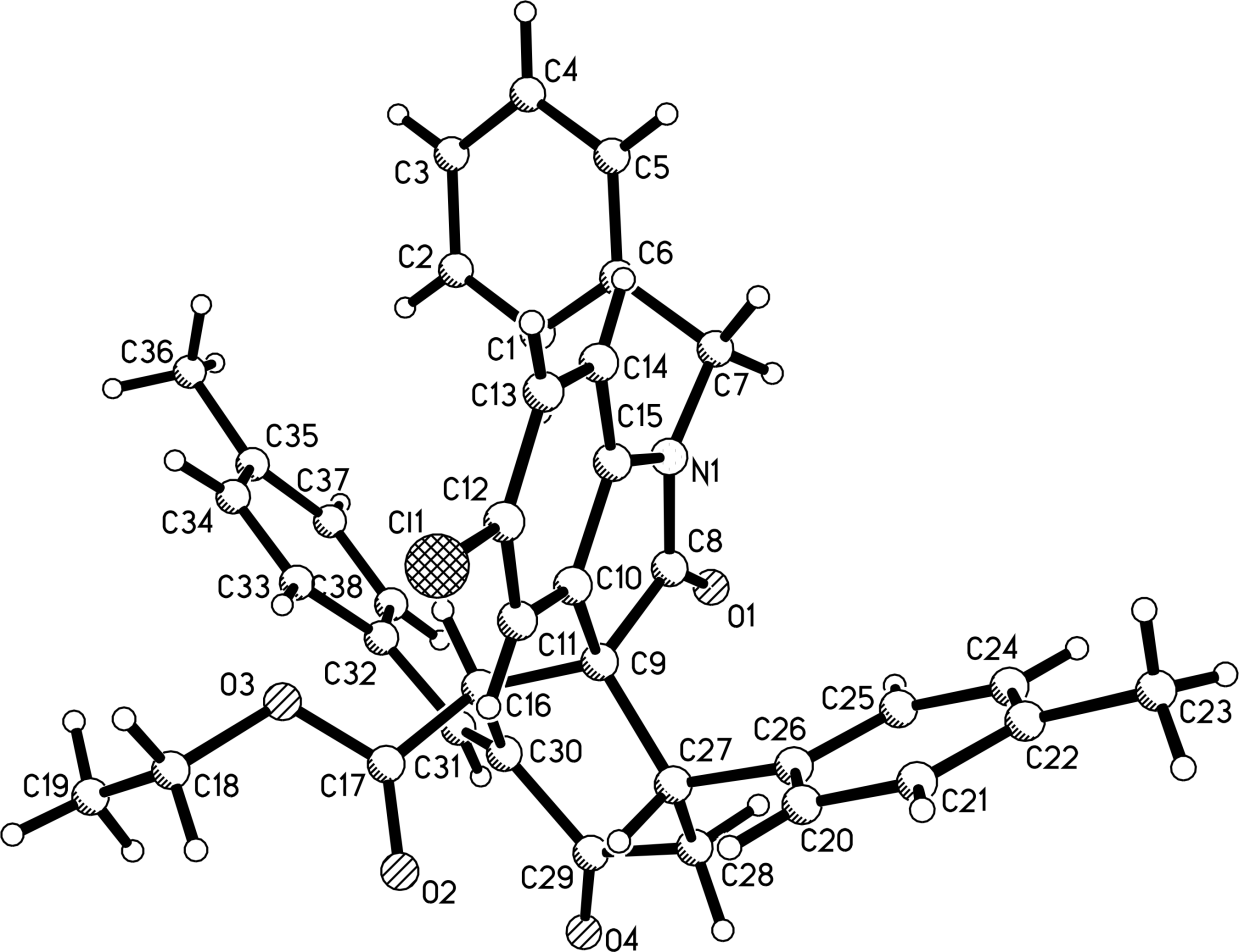
Single crystal structure of compound **5a**.

In order to expand the scope of this reaction, 3-(ethoxycarbonylmethylene)oxindoles **4** were also employed in the reaction with bis-chalcones **2**. We were pleased to find that the reaction proceeded smoothly in the presence of an excess amount of tri(*n*-butyl)phosphine under similar reaction conditions and the results are summarized in Table 3. Because there are three chiral carbon atoms in the molecule, several diastereoisomers can be formed in the reaction. The spiro[cyclohexane-1,3'-indolines] **5a**–**e** were obtained in moderate to good yields. The single crystal structure of compound **5a** was determined by X-ray diffraction method (Figure 4). It can be found that the ethoxycarbonyl group and the phenyl group of the oxindole moiety remained in the *trans*-position as in the starting 3-(ethoxycarbonylmethylene)oxindole. The aryl group and the carbonyl group exist on *trans*-position in the newly formed cyclohexyl ring. It should be pointed out that the exocyclic benzylidene group exists on the C3-position in the newly formed cyclohexyl ring, while it exists on the C6-position in the above obtained spiro compounds **3a**–**z**.

**Table 3:** Reaction of 3-(ethoxycarbonylmethylene)oxindoles and bis-chalcones.^a^

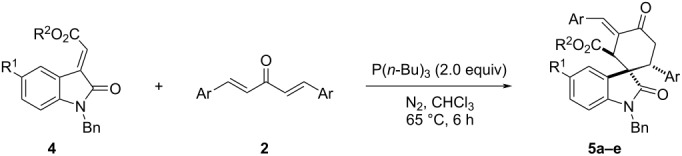					

Entry	Compound	R^1^	R^2^	Ar	Yield (%)^b^

1	**5a**	Cl	Et	*p*-CH_3_C_6_H_4_	60
2	**5b**	Cl	Et	*p*-CH_3_OC_6_H_4_	57
3	**5c**	Cl	Me	*p*-CH_3_C_6_H_4_	42
4	**5d**	Cl	Me	*p*-iPrC_6_H_4_	58
5	**5e**	H	Et	*p*-CH_3_C_6_H_4_	56

^a^Reaction conditions: 3-(ethoxycarbonylmethylene)oxindole (0.5 mmol), bis-chalcone (0.6 mmol), P(*n*-Bu)_3_ (1.0 mmol), CHCl_3_ (10.0 mL), N_2_ atmosphere; ^b^isolated yields.

This result clearly indicated that these two reactions have the opposite regioselectivity. Another kind of readily available 1,3-dipolarophile, 3-phenacylidenoxindole, was also tested in the reaction. However, it was found that the C=C bond in 3-phenacylidenoxindole was directly reduced to give the corresponding saturated 3-(2-oxo-2-phenylethyl)indolin-2-one. A similar reduction reaction of 3-phenacylidenoxindoles by trialkylphosphine has been previously reported in the literature [65].

**Scheme 1:**
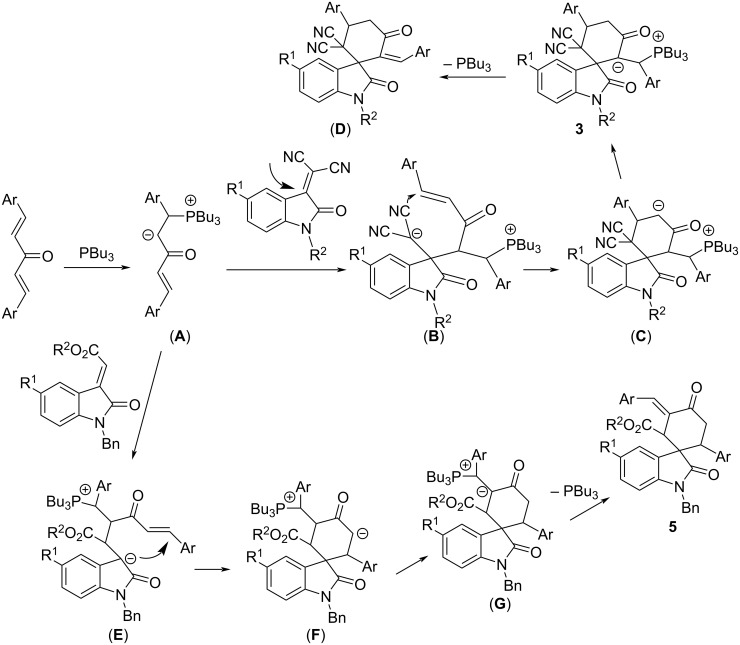
Proposed reaction mechanism for the compounds **3** and **5**.

For explaining the formation of the two kinds of spiro[cyclohexane-1,3'-indolines] **3** and **5**, a plausible reaction mechanism has been proposed (Scheme 1) on the basis of previously reported works and the obtained results from this work. Firstly, the nucleophilic addition of tributylphosphine to the bis-chalcone gives the active zwitterionic species (**A**). Secondly, the Michael addition of the zwitterionic species (**A**) to isatylidene malononitrile at the C3-position of the oxindole scaffold results in adduct (**B**). Thirdly, the intramolecular addition of the carbanion to the enone affords the cyclic intermediate (**C**), which in turn converts into the intermediate (**D**) by transfer of a negative charge. Finally, the spiro compound **3** is formed by elimination of tributylphosphine. When 3-(ethoxycarbonylmethylene)oxindole **4** is employed in the reaction, the nucleophilic addition of the zwitterion (**A**) to this compound takes place at the exocyclic position giving the adduct (**E**), which in turn proceeds with the intermediates (**F**) and (**G**) according to the above mentioned similar processes to give the spiro compound **5**. The different addition direction of the zwitterion **A** to the isatylidene malononitrile **2** and 3-(ethoxycarbonylmethylene)oxindole **4** results in the different regioselectivity in the formation of the spiro compounds **3** and **5**.

For further demonstrate the synthetic value of this procedure, ethyl isatylidene cyanoacetates **6** were also employed in the reaction. However, the reaction did not proceed to give the expected spiro[cyclohexane-1,3'-indoline], while a new spiro[indoline-3,2'-furan-3',3''-indoline] was obtained, which was clearly constructed from the annulation reaction of isatin with ethyl isatylidene cyanoacetate.

**Figure 5:**
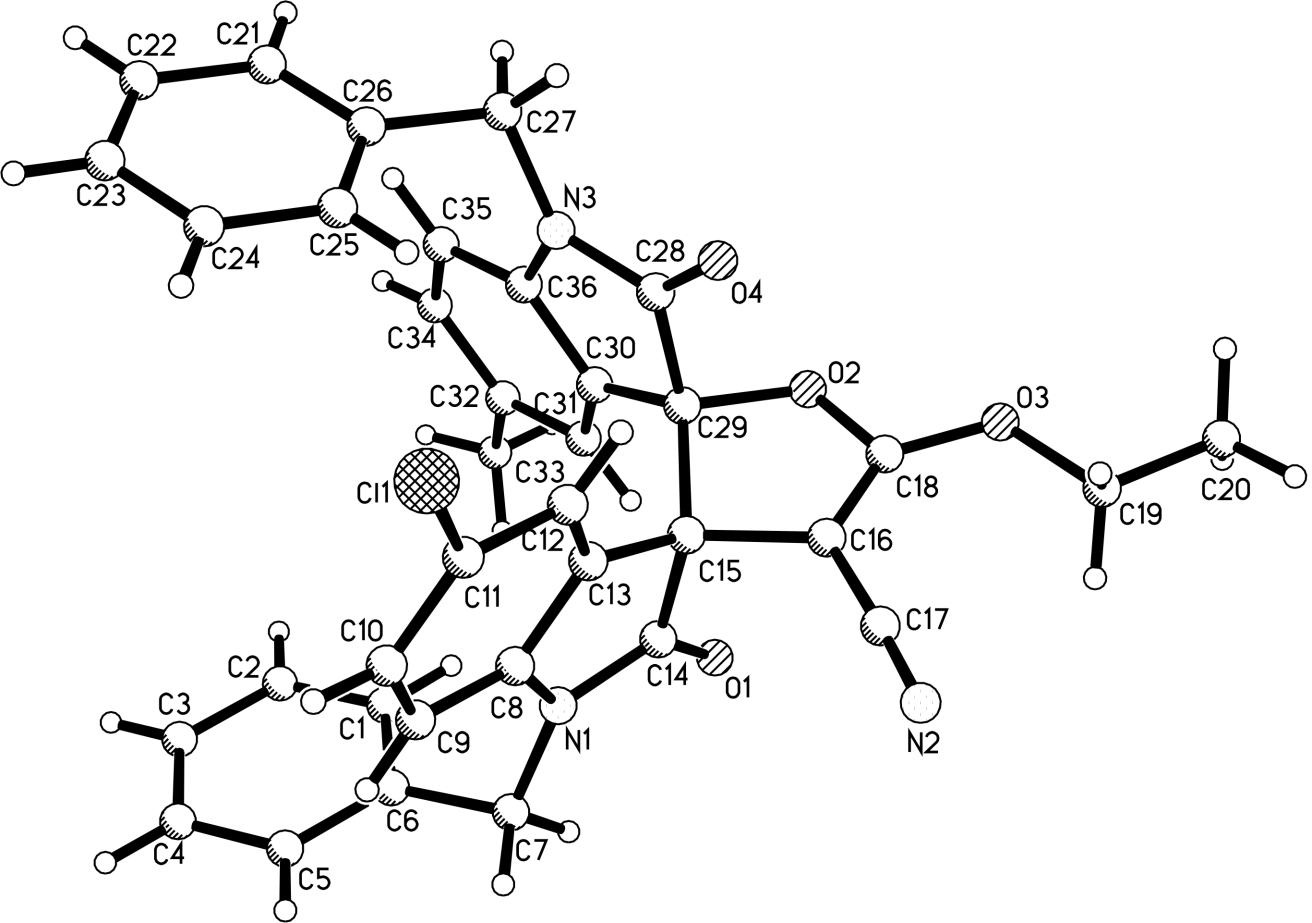
Single crystal struture of compound **8a**.

Therefore, we turned our attention to the examination of this unprecedented reaction of isatins with ethyl isatylidene cyanoacetates. At last, we successfully found that tri(*n*-butyl)phosphine promoted the reaction of ethyl isatylidene cyanoacetate **6a** and isatin **7a** always resulted in spiro[indoline-3,2'-furan-3',3''-indoline] **8a** as the major product. The loading of tri(*n*-butyl)phosphine played an important role for the formation of the products. When 2.0 equiv of P(*n*-Bu)_3_ were used, the reaction gave the spiro compound **8a** in 71% yield. Thus, this reaction is not a simple catalytic reaction and tri(*n*-butyl)phosphine acted not only as a catalyst. It was also noticed that when triphenylphosphine was used instead of tri(*n*-butyl)phosphine, the expected product was not obtained. Under the optimized reaction conditions, we next investigated the scope of the reaction by using various substituted ethyl isatylidene cyanoacetates and isatins and the results are summarized in Table 4. All reactions proceeded smoothly to give the expected spiro[indoline-3,2'-furan-3',3''-indolines] **8a**–**m** in moderate to good yields. The substituents on the isatylidene cyanoacetates had only a marginal effect on the yields, while the presence of electron-donating methyl groups in the isatins gave higher yields than electron-withdrawing groups such as chloro and fluoro substituents. The structures of the spiro compounds **8a**–**m** were fully characterized by their IR, HRMS, ^1^H and ^13^C NMR spectra. In addition, the single crystal structure of compound **8a** (Figure 5) was determined by X-ray diffraction analysis. From Figure 5, it can be seen that the two oxindole moieties exist on the *trans*-configuration. Therefore, this reaction showed very high diastereoselectivity.

**Table 4:** Reaction of ethyl isatylidene cyanoacetates and isatins.^a^

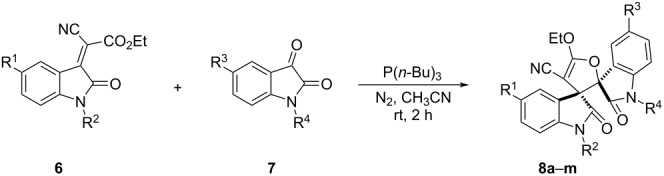						

Entry	Compound	R^1^	R^2^	R^3^	R^4^	Yield (%)^b^

1	**8a**	Cl	Bn	CH_3_	Bn	71
2	**8b**	Cl	Bn	CH_3_	H	70
3	**8c**	Cl	Bn	Cl	Bn	57
4	**8d**	Cl	Bn	F	Bn	56
5	**8e**	Cl	Bn	H	Bn	54
6	**8f**	Cl	*n*-Bu	CH_3_	Bn	76
7	**8g**	Cl	*n*-Bu	CH_3_	H	73
8	**8h**	F	Bn	CH_3_	Bn	69
9	**8i**	F	Bn	Cl	Bn	47
10	**8j**	F	Bn	F	Bn	35
11	**8k**	CH_3_	CH_3_	CH_3_	Bn	77
12	**8l**	H	Bn	CH_3_	Bn	83
13	**8m**	H	Bn	CH_3_	CH_3_	62

^a^Reaction conditions: isatin (0.3 mmol), isatylidene cyanoacetate (0.3 mmol), P(*n*-Bu)_3_ (0.6 mmol), MeCN (5.0 mL), N_2_ atmosphere; ^b^isolated yields.

**Scheme 2:**
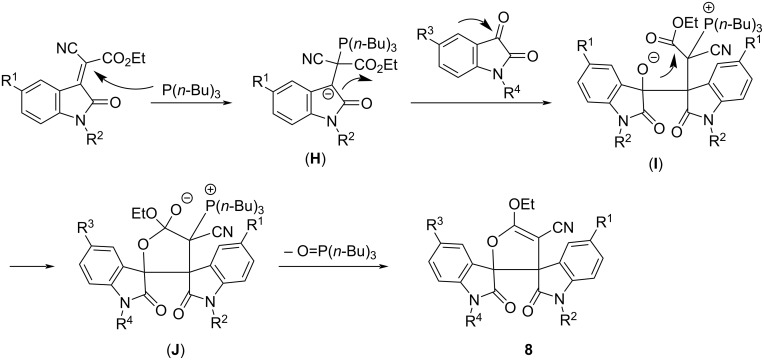
Proposed mechanism for the formation of dispiro compounds **8**.

To explain the formation of the dispiro compounds **8**, a plausible reaction mechanism has been proposed and is shown in Scheme 2. At first, the nucelophilic addition of tributylphosphine to isatylidene cyanoacetate gives a zwitterionic salt (**H**). Secondly, the addition of the carbanion to the carbonyl group of the isatin affords the adduct (**I**). Then, the intramolecular attack of the alkoxide to the carbonyl group in the ester moiety produces the cyclic intermediate (**J**). Finally, the dispiro compound **8** is formed by elimination of tri(*n*-butyl)phosphine oxide.

## Conclusion

In summary, we have investigated tri(*n*-butyl)phosphine-catalyzed annulation reactions of bis-chalcones with isatylidene malononitriles or 3-(ethoxycarbonylmethylene)oxindoles for the efficient construction of two kinds of spiro[cyclohexane-1,3'-indolines] in good yields and with good diastereoselectivity. Additionally, tri(*n*-butyl)phosphine promoted the domino reaction of isatins and ethyl isatylidene cyanoacetates to give selectively spiro[indoline-3,2'-furan-3',3''-indolines] in satisfactory yields. The relative configuration of the complex spirooxindoles was confirmed by determination of several single crystal structures. Also, plausible reaction mechanisms have been proposed. This reaction has the advantages of using readily available substrates, simple operation, good yields, and molecular diversity, which enable it to find potential applications in heterocyclic and medicinal chemistry.

## Experimental

**1. General procedure for the preparation of the spiro[cyclohexane-1,3'-indolines] 3a–w:** In an atmosphere of nitrogen, isatylidene malononitrile (0.5 mmol) and bis-chalcone (0.6 mmol) were dissolved in chloroform (10.0 mL) in a Schlenk bottle. Then, tri(*n*-butyl)phosphine (20% equiv) was added by syringe and the solution was stirred at 65 °C for six hours. After removing the solvent at reduced pressure, the residue was subjected to column chromatography with petroleum ether/ethyl acetate 15:1 (v/v) as eluent to give the pure product **3a**–**m** for analysis.

***rel*-(1*R*,3*R*)-1'-Benzyl-5'-methyl-6-((*Z*)-4-methylbenzylidene)-2',5-dioxo-3-(*p*-tolyl)spiro[cyclohexane-1,3'-indoline]-2,2-dicarbonitrile (3a)**: white solid, 84% yield; mp 213–215 °C; ^1^H NMR (600 MHz, CDCl_3_) δ 7.70 (s, 1H, ArH), 7.43 (s, 2H, ArH), 7.33 (s, 4H, ArH), 7.29 (s, 1H, ArH), 7.25–7.22 (m, 4H, ArH), 7.19–7.18 (m, 1H, ArH), 7.13–7.11 (m, 2H, ArH), 6.76–6.75 (m, 1H, ArH), 6.63 (s, 1H, CH), 5.07 (d, *J* = 15.6 Hz, 1H, CH_2_), 5.02 (d, *J* = 13.2 Hz, 1H, CH_2_), 4.86 (d, *J* = 15.0 Hz, 1H, CH_2_), 3.46 (t, *J* = 13.8 Hz, 1H, CH), 3.08 (d, *J* = 15.6 Hz, 1H, CH_2_), 2.40 (s, 3H, CH_3_), 2.38 (s, 3H, CH_3_), 2.34 (s, 3H, CH_3_); ^13^C NMR (100 MHz, CDCl_3_) δ 196.9, 171.7, 140.7, 140.4, 139.8, 139.6, 134.4, 134.0, 131.9, 131.6, 130.8, 130.1, 129.8, 129.3, 129.1, 129.0, 128.9, 128.0, 127.1, 127.0, 123.0, 112.3, 110.9, 60.3, 49.0, 44.6, 44.4, 42.6, 21.4, 21.2; IR (KBr) ν: 3727, 3405, 3029, 2921, 2863, 2317, 1911, 1709, 1609, 1501, 1443, 1362, 1295, 1185, 1049, 1022, 959, 920, 820, 732 cm^−1^; HRMS–ESI (*m*/*z*): [M + Na]^+^ calcd for C_38_H_31_NaN_3_O_2_, 584.2314; found, 584.2306.

**2. General procedure for the preparation of the spiro[cyclohexane-1,3'-indolines] 5a–e:** In an atmosphere of nitrogen, 3-(ethoxycarbonylmethyl)oxindole (0.5 mmol) and bis-chalcone (0.6 mmol) were dissolved in chloroform (10.0 mL) in a Schlenk bottle. Then, tri(*n*-butyl)phosphine (1.0 mmol) was added by syringe and the solution was stirred at 65 °C for six hours. After removing the solvent at reduced pressure, the residue was subjected to column chromatography with petroleum ether/ethyl acetate 15:1 (v/v) as eluent to give the pure product **5a**–**e** for analysis.

**Ethyl *rel*-(1*S*,2*S*,6*R*)-1'-benzyl-5'-chloro-3-((*Z*)-4-methylbenzylidene)-2',5-dioxo-6-(*p*-tolyl)spiro[cyclohexane-1,3'-indoline]-2-carboxylate (5a)**: white solid, 60% yield; mp 221–223 °C; ^1^H NMR (400 MHz, CDCl_3_) δ 7.67 (s, 1H, ArH), 7.29 (d, *J* = 7.6 Hz, 2H, ArH), 7.21–7.14 (m, 4H, ArH), 7.07 (t, *J* = 7.6 Hz, 2H, ArH), 7.01 (d, *J* = 8.4 Hz, 1H, ArH), 6.91 (d, *J* = 8.0 Hz, 2H, ArH), 6.84 (d, *J* = 8.0 Hz, 2H, ArH), 6.77 (d, *J* = 7.6 Hz, 2H, ArH), 6.34 (d, *J* = 8.4 Hz, 1H, CH), 4.70 (d, *J* = 16.0 Hz, 1H, CH_2_), 4.57 (d, *J* = 15.6 Hz, 1H, CH_2_), 4.52–4.47 (m, 1H, CH_2_), 4.36–4.24 (m, 2H, OCH_2_), 3.94 (s, 1H, CH), 3.80–3.72 (m, 1H, CH), 3.00–2.94 (m, 1H, CH_2_), 2.37 (s, 3H, CH_3_), 2.20 (s, 3H, CH_3_), 1.30 (t, *J* = 7.2 Hz, 3H, CH_3_); ^13^C NMR (100 MHz, CDCl_3_) δ 200.0, 175.1, 170.6, 140.6, 139.0, 138.8, 136.8, 135.0, 134.8, 131.6, 131.2, 130.1, 129.8, 129.3, 129.0, 128.6, 128.5, 127.5, 127.4, 127.1, 124.5, 110.2, 61.8, 53.0, 50.7, 43.4, 42.5, 42.0, 21.4, 21.0, 14.1; IR (KBr) ν: 3723, 3412, 2933, 2871, 2324, 1925, 1817, 1703, 1604, 1474, 1442, 1339, 1172, 1091, 1010, 904, 824, 716 cm^−1^; HRMS–ESI (*m*/*z*): [M + Na]^+^ calcd for C_38_H_34_ClNaNO_4_, 626.2069; found, 626.2066.

**3. General procedure for the preparation of the spiro[cyclohexane-1,3'-indolines] 8a–m:** In an atmosphere of nitrogen, isatylidene cyanoacetate (0.3 mmol) and isatin (0.3 mmol) were dissolved in acetonitrile (10.0 mL). Then, tri(*n*-butyl)phosphine (0.6 mmol) was added by syringe and the solution was stirred at room temperature for two hours. After removing the solvent at reduced pressure, the residue was subjected to column chromatography with petroleum ether/ethyl acetate 15:1 (v/v) as eluent to give the pure products **8a–m** for analysis.

***rel*-(3*R*,3'*R*)-1,1''-Dibenzyl-5''-chloro-5'-ethoxy-5-methyl-2,2''-dioxodispiro[indoline-3,2'-furan-3',3''-indoline]-4'-carbonitrile (8a)**: white solid, 71% yield; mp 175–177 °C; ^1^H NMR (400 MHz, CDCl_3_) δ 7.64 (s, 1H, ArH), 7.53 (s, 1H, ArH), 7.18–7.08 (m, 5H, ArH), 7.03 (t, *J* = 6.8 Hz, 3H, ArH), 6.71 (d, *J* = 7.6 Hz, 2H, ArH), 6.54 (d, *J* = 7.2 Hz, 2H, ArH), 6.39 (d, *J* = 8.0 Hz, 1H, ArH), 6.34 (d, *J* = 8.4 Hz, 1H, ArH), 5.17 (d, *J* = 16.4 Hz, 1H, CH_2_), 5.07 (d, *J* = 16.0 Hz, 1H, CH_2_), 4.65 (q, *J* = 7.2 Hz, 2H, CH_2_), 4.35–4.30 (m, 2H, CH_2_), 2.12 (s, 3H, CH_3_), 1.54 (t, *J* = 7.2 Hz, 3H, CH_3_); ^13^C NMR (100 MHz, CDCl_3_) δ 174.0, 173.1, 170.9, 142.4, 141.5, 134.2, 134.2, 133.6, 132.2, 130.4, 129.2, 128.8, 128.7, 128.6, 127.7, 127.6, 127.4, 126.4, 126.0, 124.0, 120.7, 114.2, 110.4, 109.6, 89.0, 69.1, 62.8, 60.1, 43.9, 20.9, 14.7; IR (KBr) ν: 3467, 3063, 3035, 2990, 2919, 2205, 1739, 1706, 1632, 1602, 1496, 1454, 1434, 1408, 1381, 1361, 1333, 1293, 1258, 1215, 1199, 1186, 1167, 1133, 1089, 1070, 1026, 997, 962, 933, 911, 895, 875, 836, 818, 776, 747 cm^−1^; HRMS–ESI (*m*/*z*): [M + Na]^+^ calcd for C_36_H_28_NaClN_3_O_4_, 624.1666; found, 624.1660.

## Supporting Information

Characterization data and ^1^H NMR, ^13^C NMR, HRMS spectra of the compounds are available. The crystallographic data of the compounds **3l** (CCDC 2166451), **3s** (CCDC 2166452), **3f’** (CCDC 2173182), **5a** (CCDC 2166453), **8a** (CCDC 2166454) have been deposited at the Cambridge Crystallographic Database Center (http://www.ccdc.cam.ac.uk).

File 1 Characterization data and copies of NMR and HRMS spectra.
