# Supplementary material for: Tri(n-butyl)phosphine-promoted domino reaction for the efficient construction of spiro[cyclohexane-1,3'-indolines] and spiro[indoline-3,2'-furan-3',3''-indolines]
Source: Beilstein J Org Chem. 2022 Jun 14;18:669–79. doi: 10.3762/bjoc.18.68 (PMC9235906; doi:10.3762/bjoc.18.68)
Supplement: File 1 — Characterization data and copies of NMR and HRMS spectra. [file Beilstein_J_Org_Chem-18-669-s001.pdf]

BEILSTEIN JOURNAL OF ORGANIC CHEMISTRY

Supporting Information

for

Tri(*n*-butyl)phosphine-promoted domino reaction for the efficient construction of spiro[cyclohexane-1,3'-indolines] and spiro[indoline-3,2'-furan-3',3''-indolines]

Hui Zheng, Ying Han, Jing Sun and Chao-Guo Yan

Beilstein J. Org. Chem. **2022,** *18,* 669–679. doi:10.3762/bjoc.18.68

Characterization data and copies of NMR and HRMS spectra

License and Terms: This is a supporting information file under the terms of the Creative Commons Attribution License (https://creativecommons.org/licenses/by/4.0). Please note that the reuse, redistribution and reproduction in particular requires that the author(s) and source are credited and that individual graphics may be subject to special legal provisions.
The license is subject to the *Beilstein Journal of Organic Chemistry* terms and conditions: (https://www.beilstein-journals.org/bjoc/terms)

***rel*-(1*R*,3*R*)-1'-Benzyl-5'-methyl-6-((*Z*)-4-methylbenzylidene)-2',5-dioxo-3-(*p*-tolyl)spiro[cyclohexane-1,3'-indoline]-2,2-dicarbonitrile (3a)**: white solid, 67%, m.p. 213-215 °C; ^{1}H NMR (600 MHz, $CDCl_3$) δ 7.70 (s, 1H, ArH), 7.43 (s, 2H, ArH), 7.33 (s, 4H, ArH), 7.29 (s, 1H, ArH), 7.25-7.22 (m, 4H, ArH), 7.19-7.18 (m, 1H, ArH), 7.13-7.11 (m, 2H, ArH), 6.76-6.75 (m, 1H, ArH), 6.63 (s, 1H, CH), 5.07 (d, J = 15.6 Hz, 1H, CH_2), 5.02 (d, J = 13.2 Hz, 1H, CH_2), 4.86 (d, J = 15.0 Hz, 1H, CH_2), 3.46 (t, J = 13.8 Hz, 1H, CH), 3.08 (d, J = 15.6 Hz, 1H, CH_2), 2.40 (s, 3H, CH_3), 2.38 (s, 3H, CH_3), 2.34 (s, 3H, CH_3). ^{13}C NMR (100 MHz, $CDCl_3$) δ 196.9, 171.7, 140.7, 140.4, 139.8, 139.6, 134.4, 134.0, 131.9, 131.6, 130.8, 130.1, 129.8, 129.3, 129.1, 129.0, 128.9, 128.0, 127.1, 127.0, 123.0, 112.3, 110.9, 60.3, 49.0, 44.6, 44.4, 42.6, 21.4, 21.2. IR (KBr) υ: 3727, 3405, 3029, 2921, 2863, 2317, 1911, 1709, 1609, 1501, 1443, 1362, 1295, 1185, 1049, 1022, 959, 920, 820, 732 cm^{-1}; MS (*m*/*z*): HRMS (ESI) Calcd. for $C_{38}H_{31}NaN_3O_2$ ([M+Na]$^+$): 584.2314, found: 584.2306.

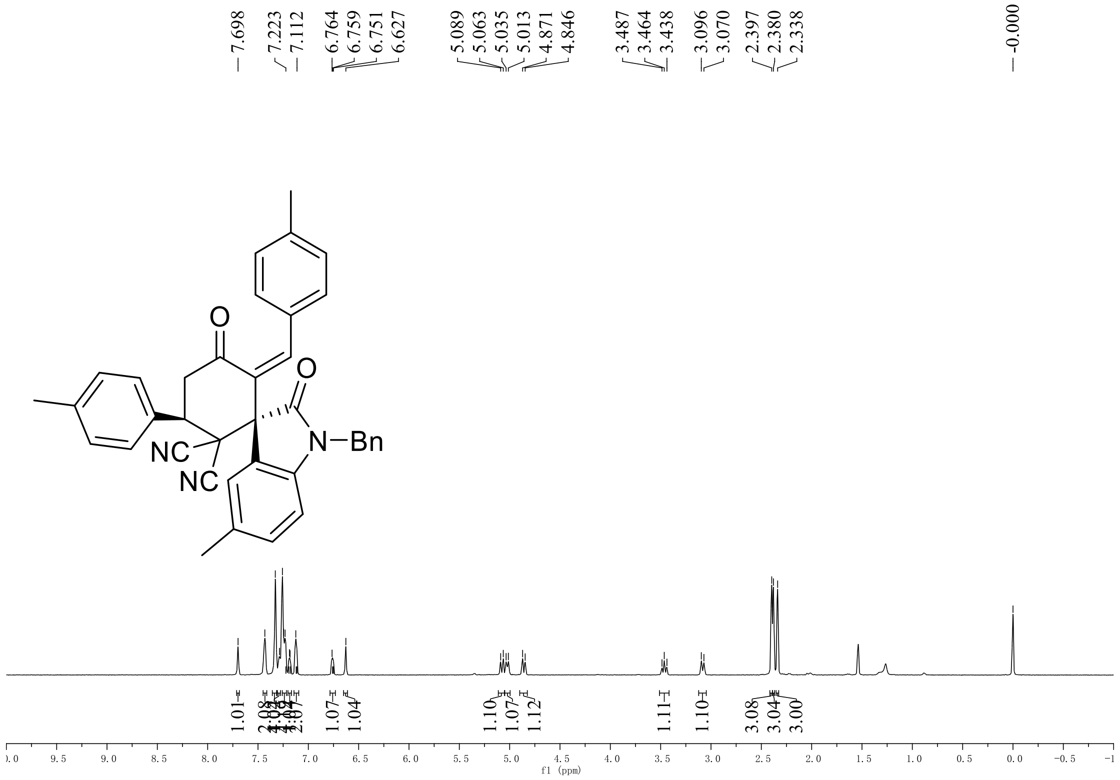

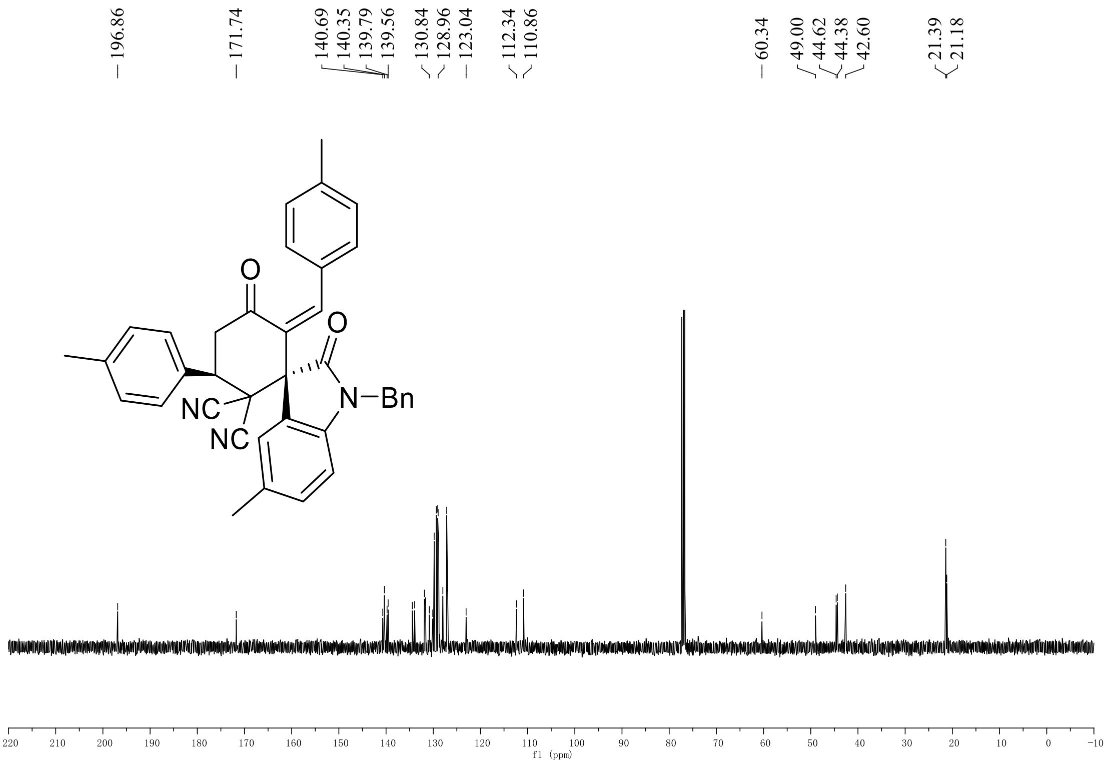
196.86
171.74
140.69
140.35
139.79
139.56
130.84
128.96
123.04
112.34
110.86
60.34
49.00
44.62
44.38
42.60
21.39
21.18
O
O
N−Bn
NC
NC
220 210 200 190 180 170 160 150 140 130 120 110 100 90 80 70 60 50 40 30 20 10 0 −10
f1 (ppm)

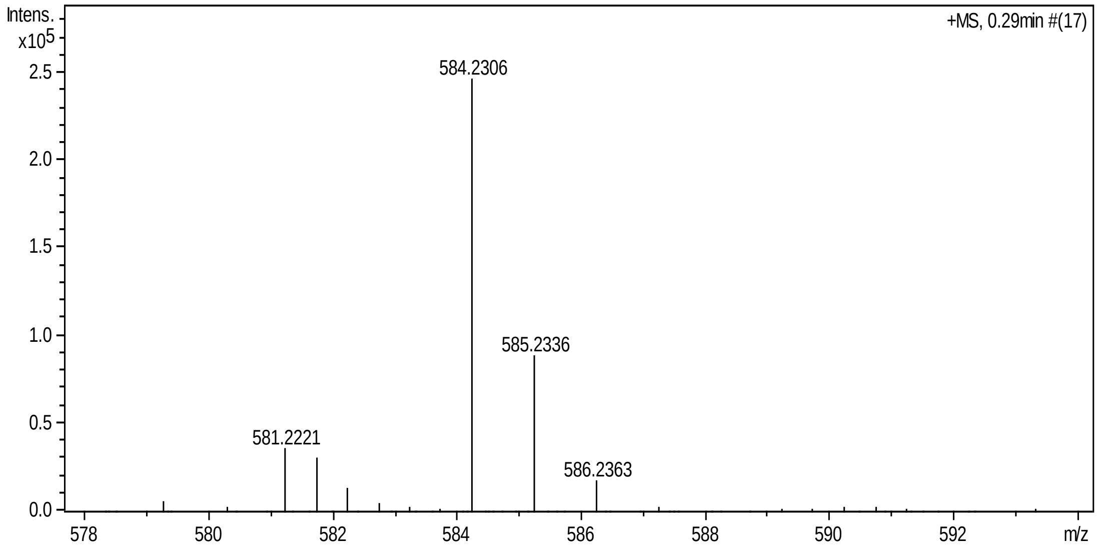
Intens.
x10^5
+MS, 0.29min #(17)
2.5
2.0
1.5
1.0
0.5
0.0
584.2306
585.2336
581.2221
586.2363
578 580 582 584 586 588 590 592 m/z

***rel*-(1*R*,3*R*)-1'-Benzyl-6-((*Z*)-4-(*tert*-butyl)benzylidene)-3-(4-(*tert*-butyl)phenyl)-5'-methyl-2',5-dioxospiro[cyclohexane-1,3'-indoline]-2,2-dicarbonitrile (3b)**: white solid, 64%, m.p. 173-175 °C; ^{1}H NMR (400 MHz, $CDCl_3$) δ 7.70 (s, 1H, ArH), 7.46 (s, 4H, ArH), 7.35-7.28 (m, 9H, ArH), 7.18 (d, *J* = 8.0 Hz, 1H, ArH), 6.77 (d, *J* = 8.0 Hz, 1H, ArH), 6.64 (s, 1H, CH), 5.09-5.01 (m, 2H, CH_2), 4.87 (d, *J* = 15.6 Hz, 1H, CH_2), 3.49 (t, *J* = 14.0 Hz, 1H, CH), 3.13-3.08 (m, 1H, CH_2), 2.38 (s, 3H, CH_3), 1.33 (s, 9H, $(CH_3)_3$), 1.30 (s, 3H, $(CH_3)_3$).^{13}C NMR (100 MHz, $CDCl_3$) δ 196.9, 171.8, 152.9, 152.6, 140.7, 140.2, 134.5, 133.9, 131.9, 131.5, 130.8, 130.1, 129.3, 129.0, 128.7, 128.0, 127.2, 127.1, 126.1, 125.3, 123.1, 112.4, 110.9, 60.4, 49.0, 44.6, 44.4, 42.6, 34.8, 34.7, 31.2, 31.1, 21.4. IR (KBr) υ: 3288, 2963, 1728, 1614, 1499, 1358, 1306, 1266, 1207, 1112, 1058, 1021, 968, 829 cm^{-1}; MS (*m*/*z*): HRMS (ESI) Calcd. for $C_{44}H_{43}NaN_3O_2$ ([M+Na]$^+$): 668.3253, found: 668.3225.

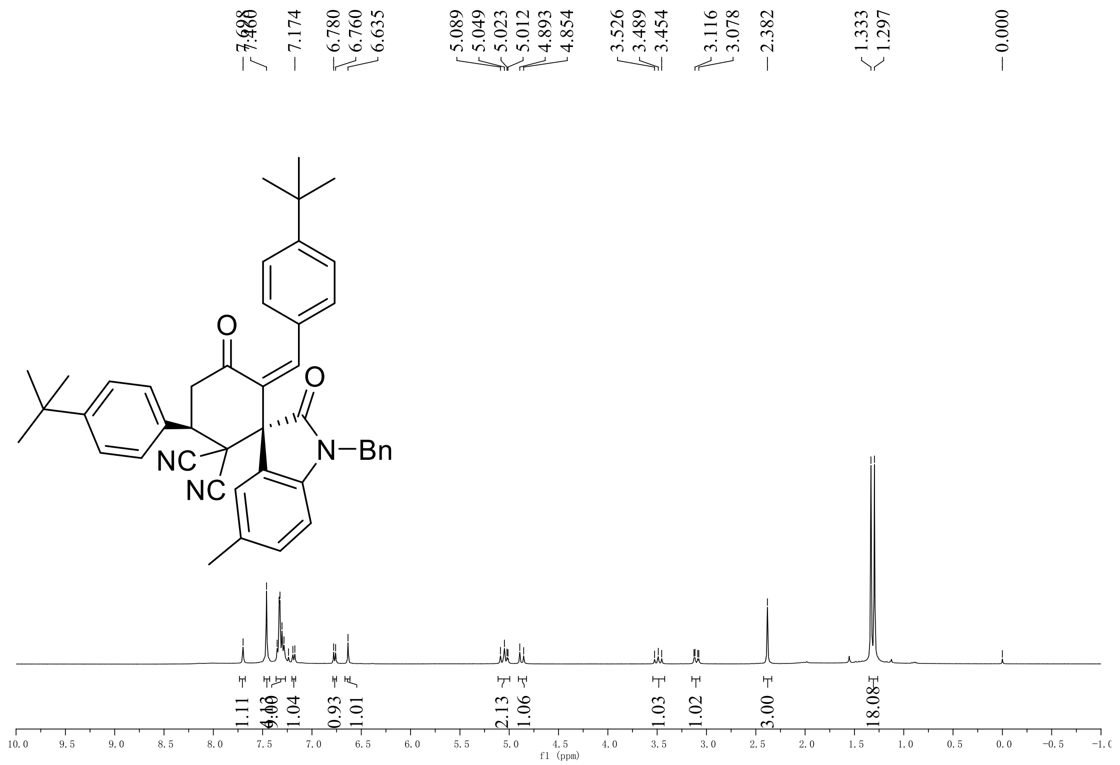

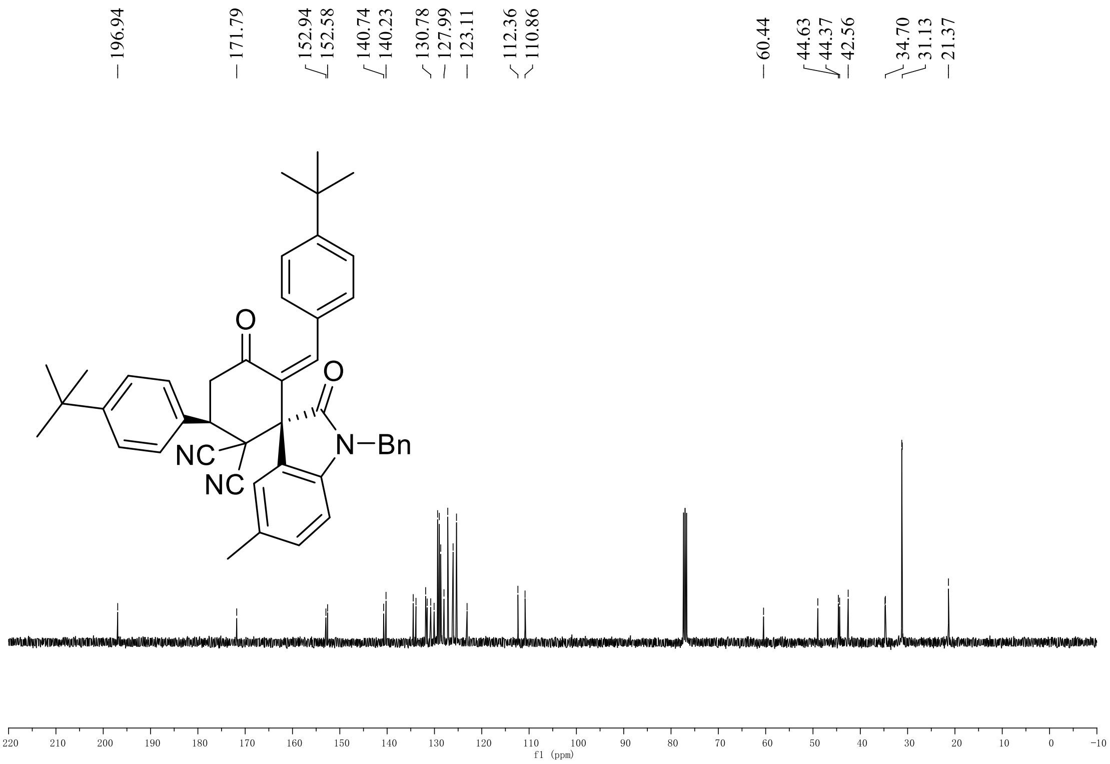
196.94
171.79
152.94
152.58
140.74
140.23
130.78
127.99
123.11
112.36
110.86
60.44
44.63
44.37
42.56
34.70
31.13
21.37
O
O
NC
NC
N−Bn
f1 (ppm)

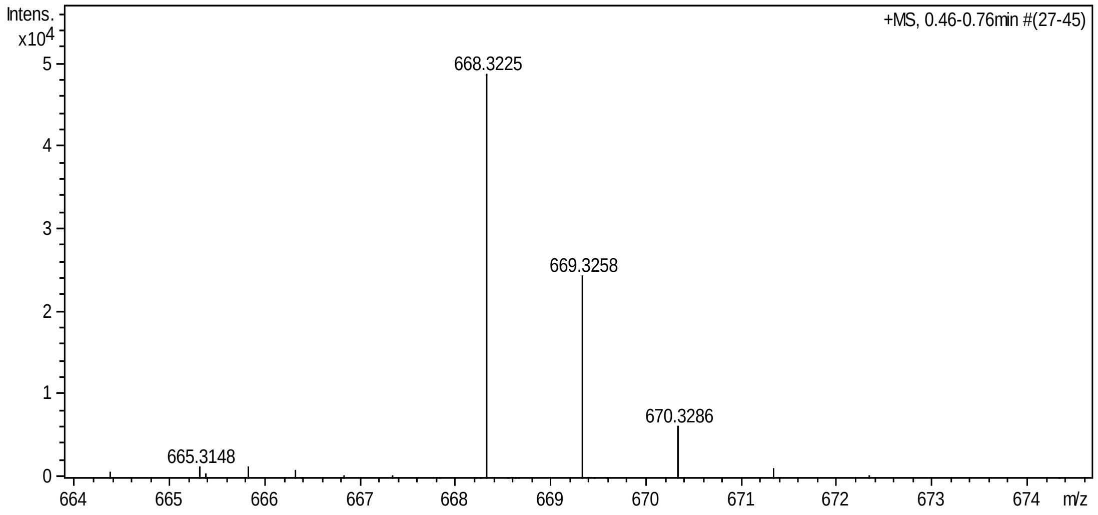
Intens.
x10^4
+MS, 0.46-0.76min #(27-45)
668.3225
669.3258
670.3286
665.3148
m/z

***rel*-(1*R*,3*R*)-1'-Benzyl-6-((*Z*)-benzylidene)-5'-methyl-2',5-dioxo-3-phenylspiro[cyclohexane-1,3'-indoline]-2,2-dicarbonitrile (3c)**: white solid, 64%, m.p. 162-164 °C; ^{1}H NMR (400 MHz, $CDCl_3$) δ 7.71 (s, 1H, ArH), 7.57-7.55 (m, 2H, ArH), 7.48-7.44 (m, 3H, ArH), 7.34-7.33 (m, 10H, ArH), 7.20 (d, *J* = 8.0 Hz, 1H, ArH), 6.78 (d, *J* = 8.4 Hz, 1H, ArH), 6.69 (s, 1H, CH), 5.11-5.05 (m, 2H, CH_2), 4.87 (d, *J* = 16.0 Hz, 1H, CH_2), 3.49 (t, *J* = 13.6 Hz, 1H, CH), 3.14-3.09 (m, 1H, CH_2), 2.40 (s, 3H, CH_3).^{13}C NMR (100 MHz, $CDCl_3$) δ 196.5, 171.6, 140.7, 140.4, 134.5, 134.4, 134.0, 133.8, 132.0, 131.2, 129.6, 129.3, 129.2, 129.1, 129.0, 128.9, 128.3, 128.0, 127.2, 127.0, 122.9, 112.2, 112.1, 110.9, 60.3, 48.9, 44.7, 44.3, 42.9, 29.7, 21.4. IR (KBr) υ: 3728, 3399, 3032, 2918, 2852, 2319, 1948, 1882, 1709, 1609, 1492, 1441, 1348, 1189, 1064, 1018, 926, 811, 772, 733 cm^{-1}; MS (*m*/*z*): HRMS (ESI) Calcd. for $C_{36}H_{27}NaN_3O_2$ ($[M+Na]^+$): 556.2001, found: 556.1991.

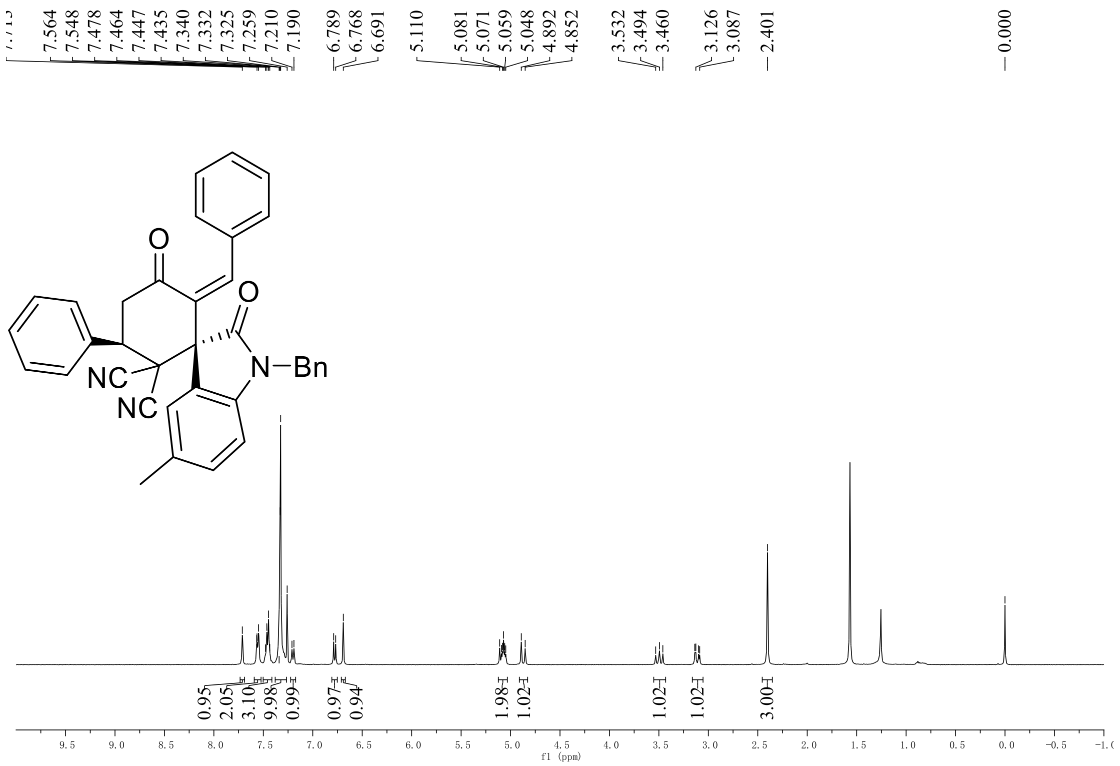

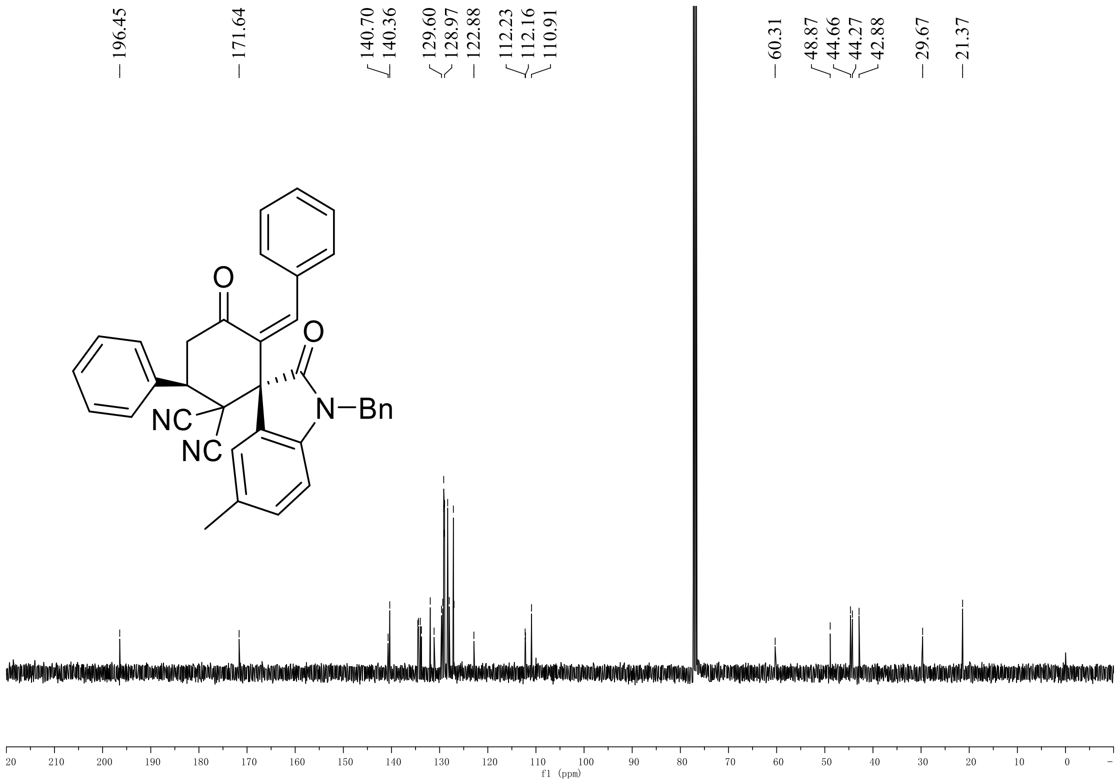
196.45
171.64
140.70
140.36
129.60
128.97
122.88
112.23
112.16
110.91
60.31
48.87
44.66
44.27
42.88
29.67
21.37
O
O
N−Bn
NC
NC
f1 (ppm)

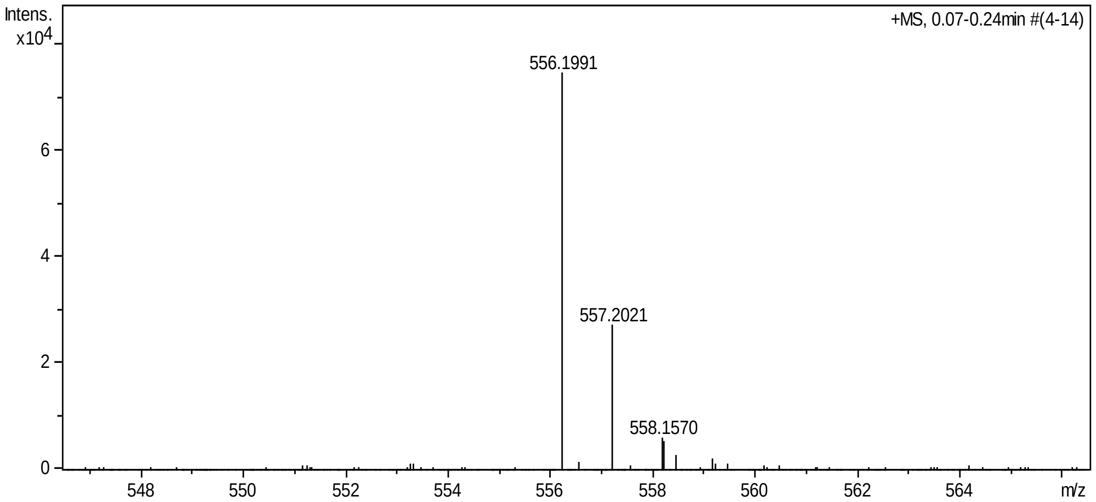
Intens.
x10^4
+MS, 0.07-0.24min #(4-14)
556.1991
557.2021
558.1570
m/z

***rel*-(1*R*,3*R*)-1'-Benzyl-6-((*Z*)-4-chlorobenzylidene)-3-(4-chlorophenyl)-5'-methyl-2',5-dioxospiro[cyclohexane-1,3'-indoline]-2,2-dicarbonitrile (3d)**: pole pink solid, 58%, m.p. 244-246 °C; ^{1}H NMR (400 MHz, $CDCl_3$) δ 7.69 (s, 1H, ArH), 7.49-7.43 (m, 4H, ArH), 7.32-7.28 (m, 9H, ArH), 7.21 (d, *J* = 8.0 Hz, 1H, ArH), 6.80 (d, *J* = 8.0 Hz, 1H, ArH), 6.63 (s, 1H, CH), 5.10-5.04 (m, 2H, CH_2), 4.86 (d, *J* = 15.6 Hz, 1H, CH_2), 3.39 (t, *J* = 14.0 Hz, 1H, CH), 3.10-3.05 (m, 1H, CH_2), 2.41 (s, 3H, CH_3).^{13}C NMR (100 MHz, $CDCl_3$) δ 195.7, 171.5, 140.7, 139.2, 135.8, 135.5, 134.3, 134.2, 132.9, 132.2, 132.1, 131.5, 130.6, 130.3, 129.5, 129.0, 128.6, 128.1, 127.1, 126.9, 122.6, 112.1, 112.0, 111.1, 60.2, 48.7, 44.7, 44.1, 42.2, 21.4. IR (KBr) υ: 3726, 3630, 3405, 3065, 2925, 2316, 1903, 1708, 1610, 1494, 1443, 1414, 1357, 1291, 1187, 1091, 1059, 1013, 917, 824, 732 cm^{-1} MS (*m*/*z*): HRMS (ESI) Calcd. for $C_{36}H_{25}NaCl_2N_3O_2$ ([M+Na]$^{+}$): 624.1222, found: 624.1204.

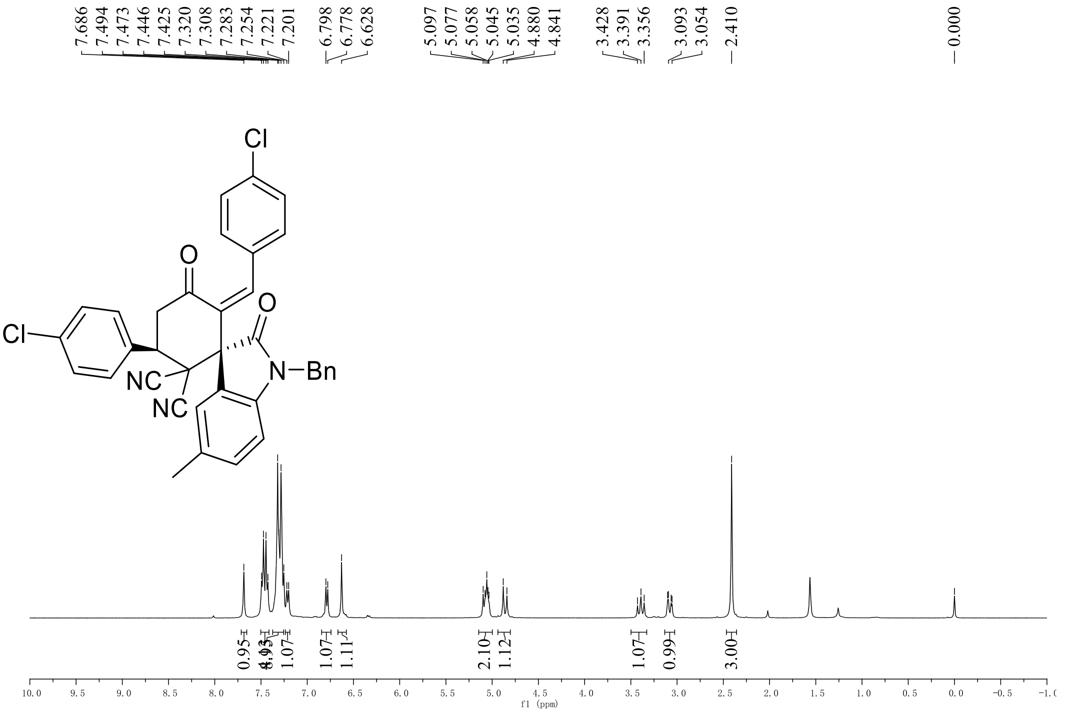

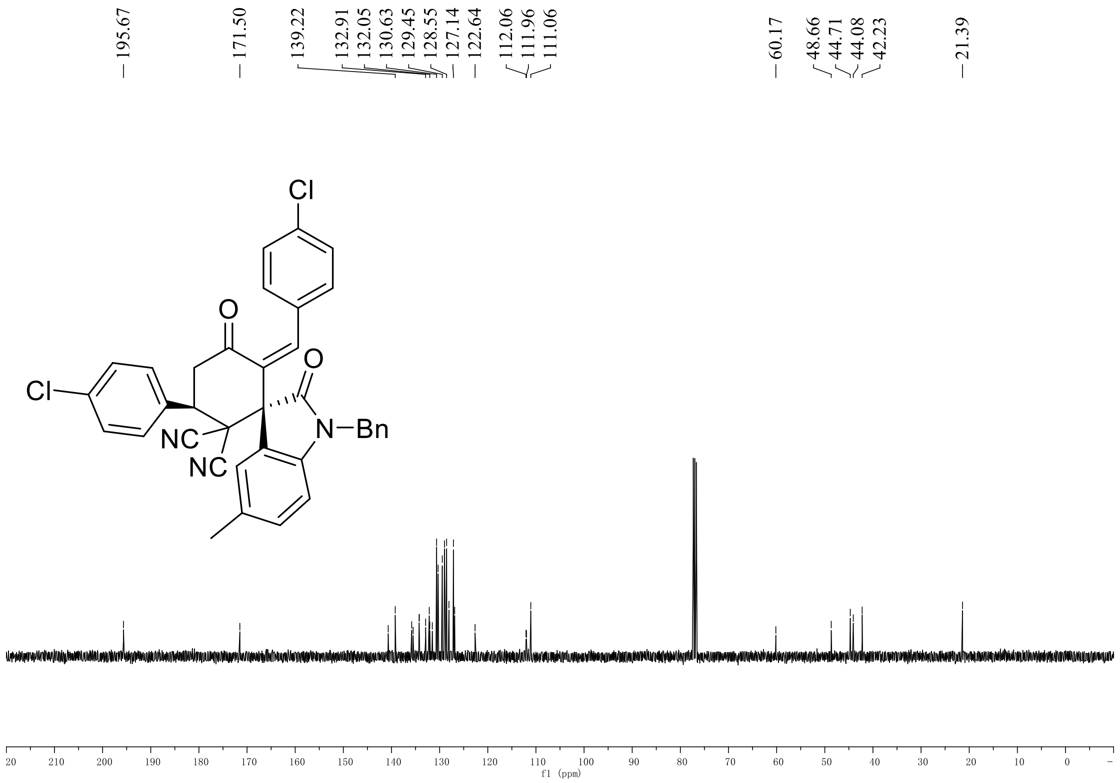
195.67
171.50
139.22
132.91
132.05
130.63
129.45
128.55
127.14
122.64
112.06
111.96
111.06
60.17
48.66
44.71
44.08
42.23
21.39
Cl
O
O
Cl
N−Bn
NC
NC
f1 (ppm)

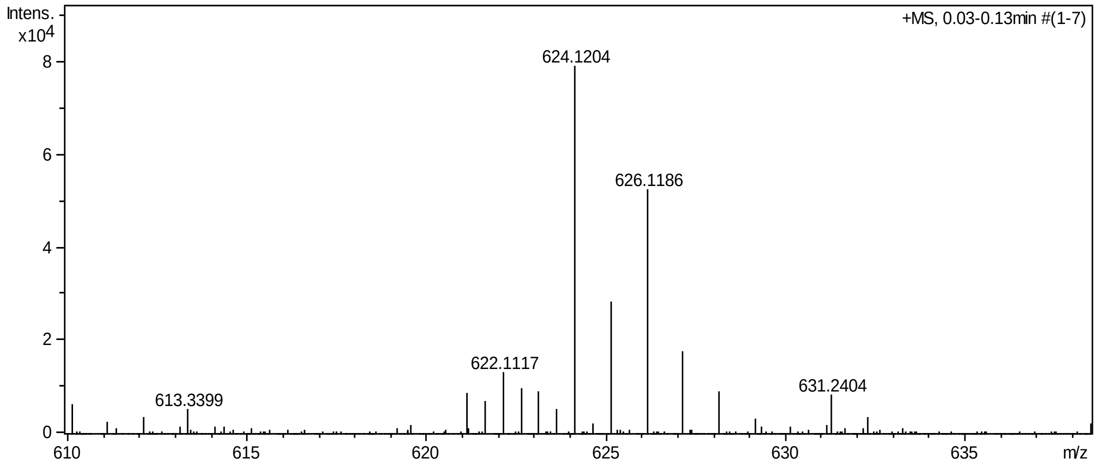
Intens.
x10^4
+MS, 0.03-0.13min #(1-7)
624.1204
626.1186
622.1117
631.2404
613.3399
m/z

***rel*-(1*R*,3*R*)-1'-Benzyl-6-((*Z*)-4-bromobenzylidene)-3-(4-bromophenyl)-5'-methyl-2',5-dioxospiro[cyclohexane-1,3'-indoline]-2,2-dicarbonitrile (3e)**: white solid, 62%, m.p. 233-234 °C; ^{1}H NMR (400 MHz, $CDCl_3$) δ 7.68 (s, 1H, ArH), 7.60 (d, *J* = 6.8 Hz, 2H, ArH), 7.46-7.41 (m, 4H, ArH), 7.36-7.30 (m, 5H, ArH), 7.23-7.19 (m, 3H, ArH), 6.79 (d, *J* = 8.4 Hz, 1H, ArH), 6.61 (s, 1H, CH), 5.10-5.02 (m, 2H, CH_2), 4.86 (d, *J* = 16.0 Hz, 1H, CH_2), 3.39 (t, *J* = 14.8 Hz, 1H, CH), 3.10-3.05 (m, 1H, CH_2), 2.41 (s, 3H, CH_3). ^{13}C NMR (100 MHz, $CDCl_3$) δ 195.6, 171.4, 140.7, 139.3, 134.2, 133.4, 132.5, 132.4, 132.2, 131.6, 131.5, 130.8, 130.6, 129.0, 128.1, 127.1, 126.9, 124.0, 123.8, 122.6, 112.0, 111.9, 111.1, 60.2, 48.5, 44.7, 44.0, 42.3, 21.4. IR (KBr) υ: 3725, 3627, 3401, 3065, 2924, 2316, 1902, 1708, 1613, 1493, 1443, 1411, 1357, 1291, 1187, 1110, 1070, 1009, 959, 917, 821, 733 cm^{-1}; MS (*m/z*): HRMS (ESI) Calcd. for $C_{36}H_{25}NaBr_2N_3O_2$ ([M+Na]$^+$): 712.0211, found: 712.0167.

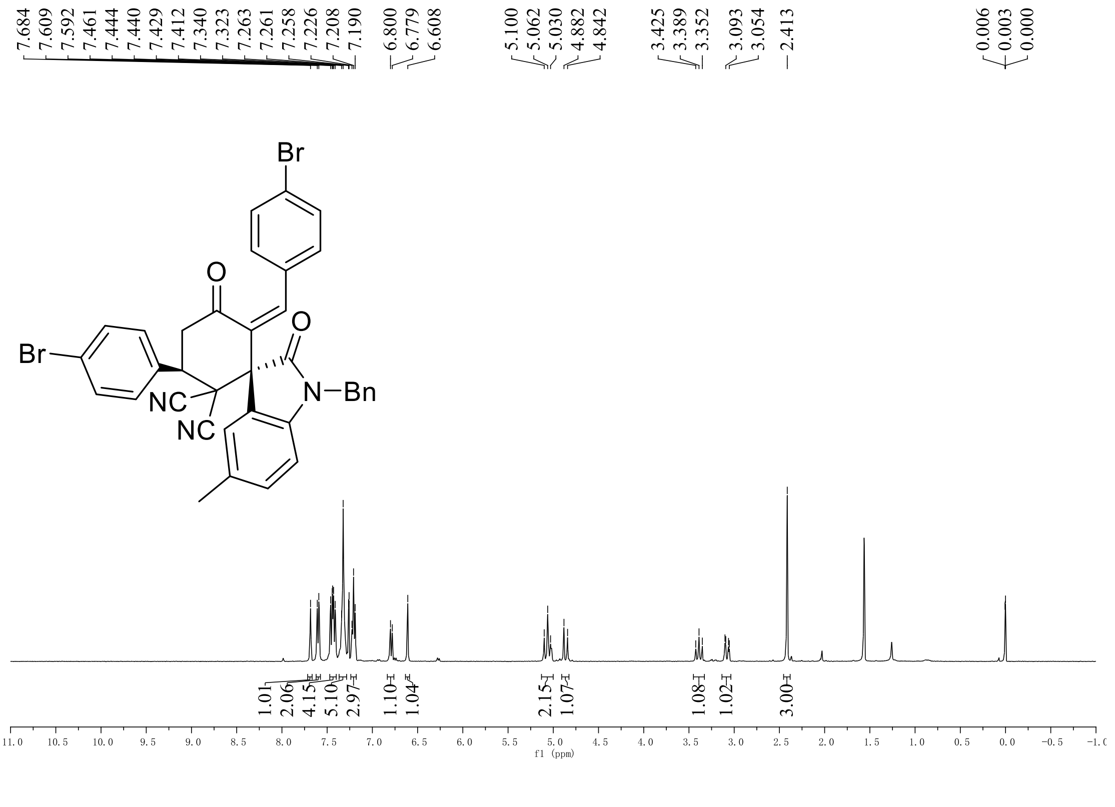

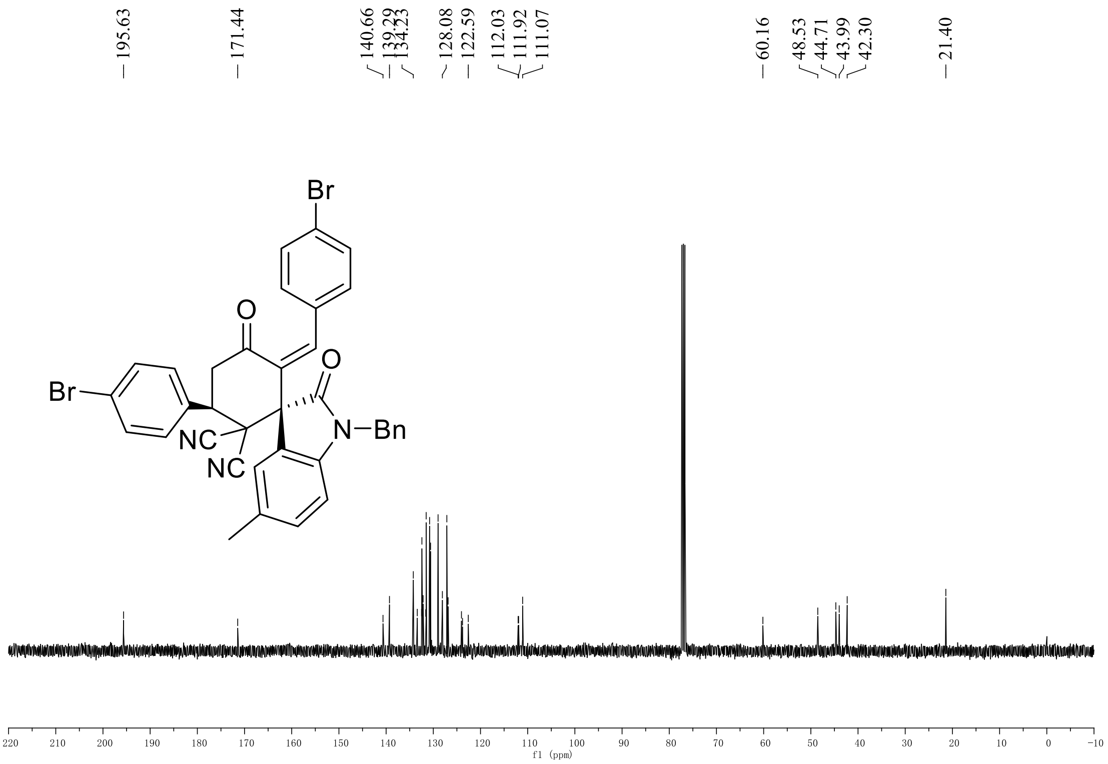

195.63
171.44
140.66
139.29
134.23
128.08
122.59
112.03
111.92
111.07
60.16
48.53
44.71
43.99
42.30
21.40
Br
O
O
Br
NC
NC
N−Bn
220 210 200 190 180 170 160 150 140 130 120 110 100 90 80 70 60 50 40 30 20 10 0 −10
f1 (ppm)

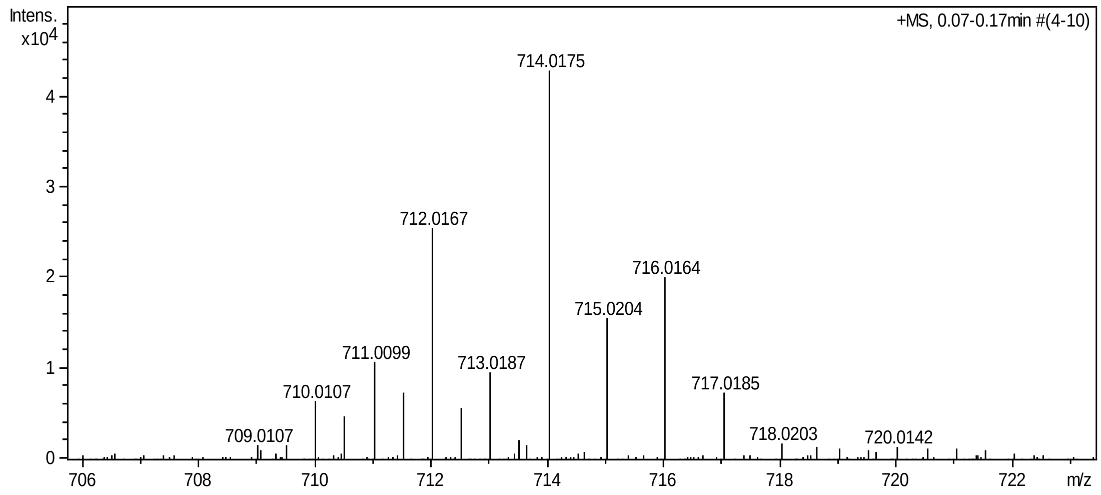

Intens.
x10^4
+MS, 0.07-0.17min #(4-10)
714.0175
712.0167
716.0164
715.0204
711.0099
713.0187
710.0107
717.0185
709.0107
718.0203
720.0142
0
1
2
3
4
706 708 710 712 714 716 718 720 722 m/z

***rel*-(1*R*,3*R*)-6-((*Z*)-4-(*tert*-Butyl)benzylidene)-3-(4-(*tert*-butyl)phenyl)-1',5'-dimethyl-2',5-dioxospiro[cyclohexane-1,3'-indoline]-2,2-dicarbonitrile (3f)**: yellow solid, 42%, m.p. 212-214 °C; ^{1}H NMR (400 MHz, $CDCl_3$) δ 7.68 (s, 1H, ArH), 7.43 (s, 4H, ArH), 7.32-7.26 (m, 5H, ArH), 6.95 (s, 1H, ArH), 6.65 (s, 1H, CH), 4.96 (d, *J* = 11.2 Hz,, 1H, CH_2), 3.45 (t, *J* = 16.0 Hz, 1H, CH), 3.29 (s, 3H, CH_3), 3.07 (d, *J* = 10.8 Hz, 1H, CH_2), 2.44 (s, 3H, CH_3), 1.32 (s, 9H, $(CH_3)_3$), 1.29 (s, 9H, $(CH_3)_3$). ^{13}C NMR (100 MHz, $CDCl_3$) δ 193.5, 171.9, 142.7, 139.1, 137.3, 136.0, 135.9, 134.1, 131.9, 129.9, 128.1, 127.5, 127.1, 126.7, 126.5, 125.4, 121.5, 112.0, 111.9, 111.0, 59.6, 48.9, 44.3, 37.7, 27.2. IR (KBr) υ: 3030, 2956, 2869, 1710, 1611, 1502, 1424, 1358, 1195, 1149, 1052, 939, 817, 738 cm^{-1}; MS (*m*/*z*): HRMS (ESI) Calcd. for $C_{38}H_{39}NaN_3O_2$ ([M+Na]$^+$): 592.2940, found: 592.2919.

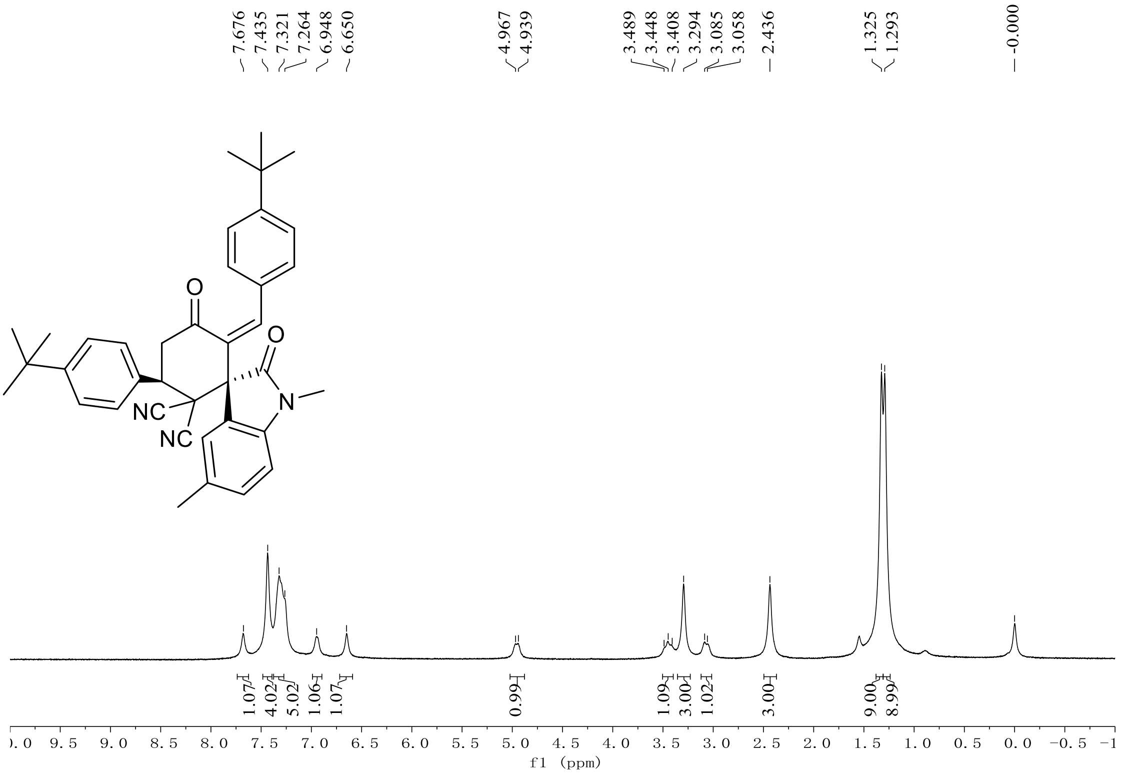

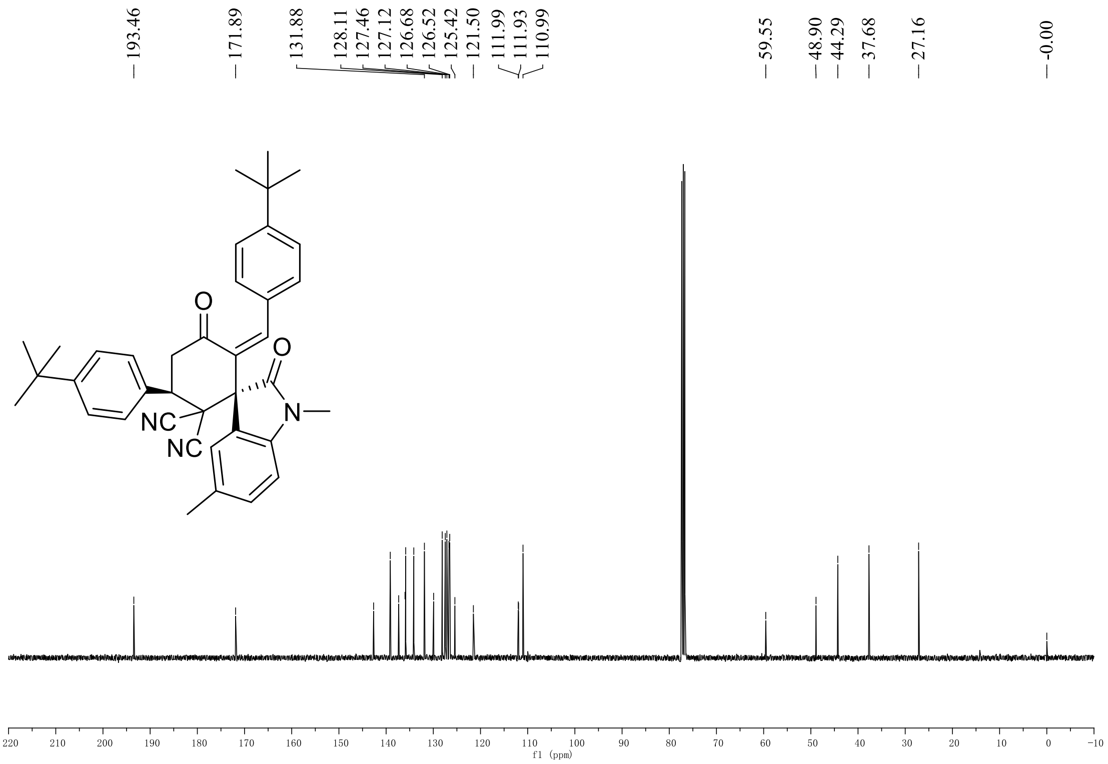
193.46
171.89
131.88
128.11
127.46
127.12
126.68
126.52
125.42
121.50
111.99
111.93
110.99
59.55
48.90
44.29
37.68
27.16
-0.00
O
O
N
NC
NC
220 210 200 190 180 170 160 150 140 130 120 110 100 90 80 70 60 50 40 30 20 10 0 −10
f1 (ppm)

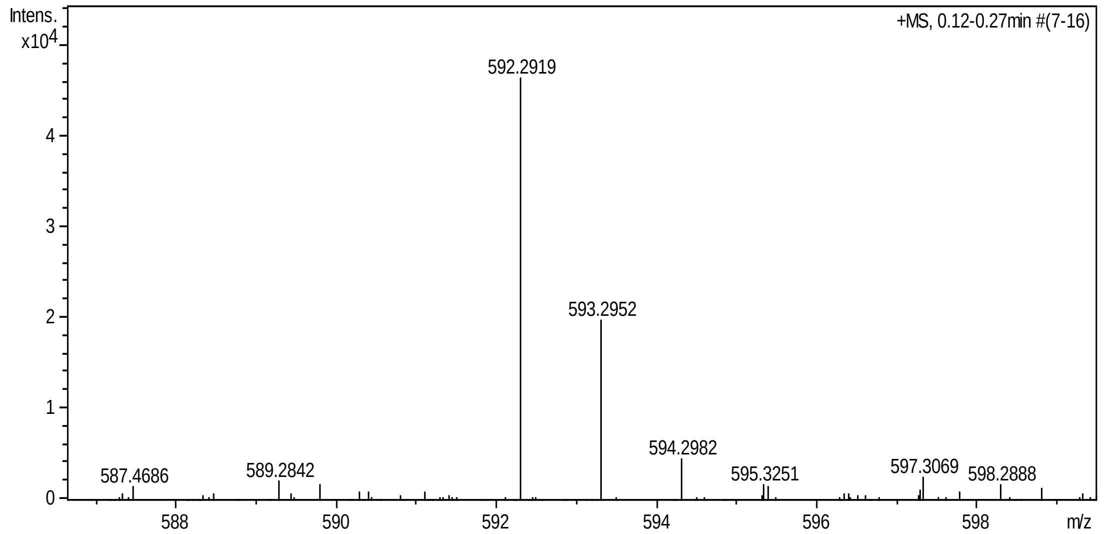
Intens.
x10^4
+MS, 0.12-0.27min #(7-16)
592.2919
593.2952
594.2982
587.4686
589.2842
595.3251
597.3069
598.2888
0
1
2
3
4
588
590
592
594
596
598
m/z

***rel*-(1*R*,3*R*)-6-((*E*)-4-(*tert*-Butyl)benzylidene)-3-(4-(*tert*-butyl)phenyl)-1',5'-dimethyl-2',5-dioxospiro[cyclohexane-1,3'-indoline]-2,2-dicarbonitrile (3f')**: yellow solid, 14%, m.p. 172-174 °C; ^{1}H NMR (400 MHz, $CDCl_3$) δ 8.10 (s, 1H, ArH), 7.43 (s, 4H, ArH), 7.01-6.94 (m, 4H, ArH), 6.48-6.47 (m, 3H, ArH), 5.02-4.99 (m, 1H, CH_2), 3.43 (m, 1H, CH), 3.21 (s, 1H, CH_2), 3.15 (s, 3H, CH_3), 2.13 (s, 3H, CH_3), 1.32 (s, 9H, $(CH_3)_3$), 1.22 (s, 9H, $(CH_3)_3$). ^{13}C NMR (100 MHz, $CDCl_3$) δ 194.4, 172.9, 152.4, 150.7, 147.5, 141.0, 133.1, 132.2, 131.5, 130.7, 128.4, 127.2, 126.6, 126.1, 125.2, 124.2, 112.4, 111.7, 108.6, 56.3, 49.1, 41.0, 39.0, 34.7, 34.5, 31.2, 31.0, 26.8, 21.0; IR (KBr) υ: 3125, 3021, 2764, 1715, 1610, 1531, 1415, 1342, 1143, 1121, 1035, 940, 815, 732 cm^{-1}; MS (*m*/*z*): HRMS (ESI) Calcd. for $C_{38}H_{39}NaN_3O_2$ ($[M+Na]^+$): 592.2940, found: 592.2920.

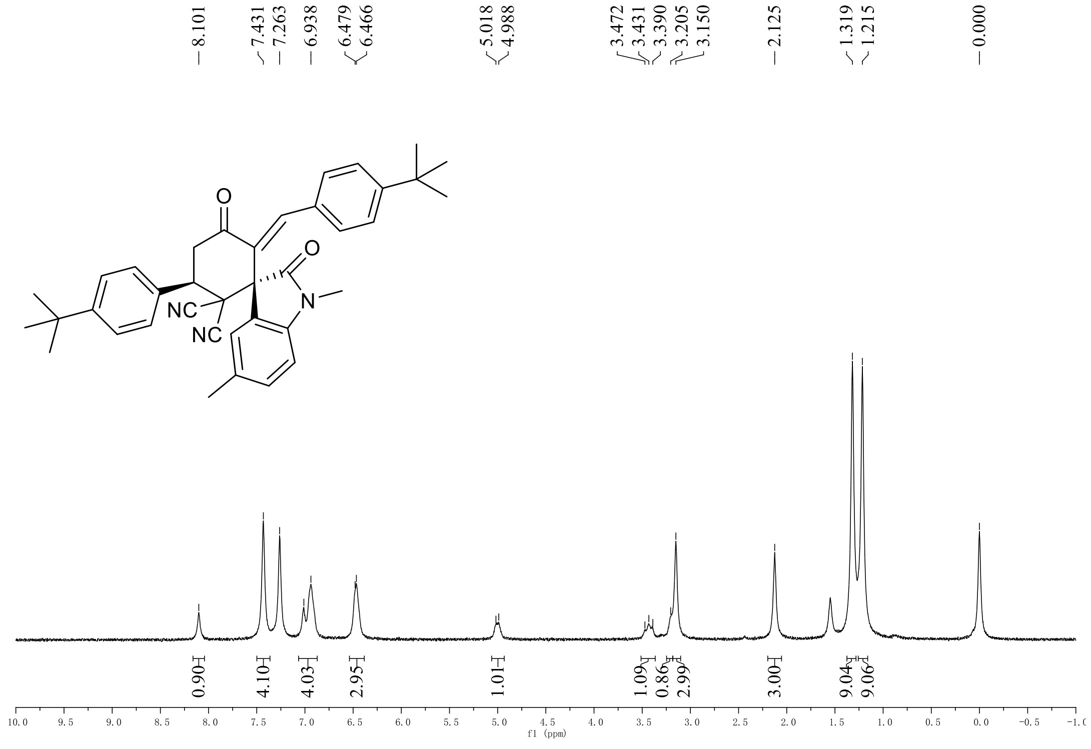

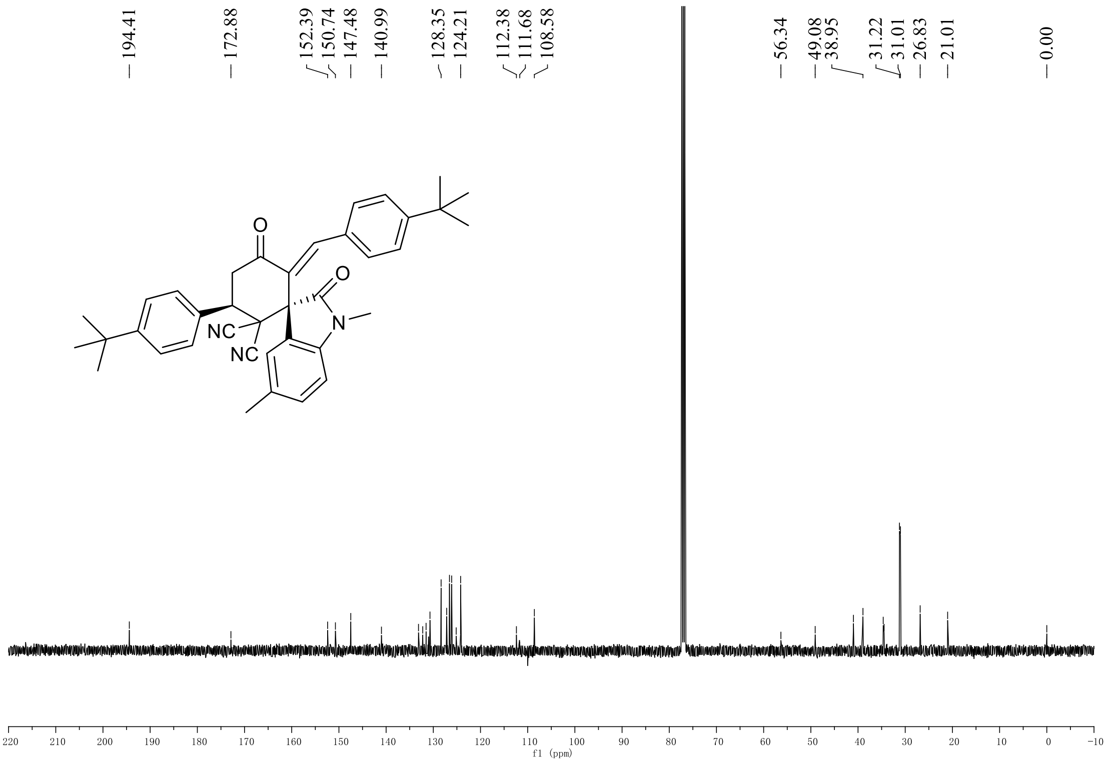
194.41
172.88
152.39
150.74
147.48
140.99
128.35
124.21
112.38
111.68
108.58
56.34
49.08
38.95
31.22
31.01
26.83
21.01
0.00
O
O
N
NC
NC
f1 (ppm)

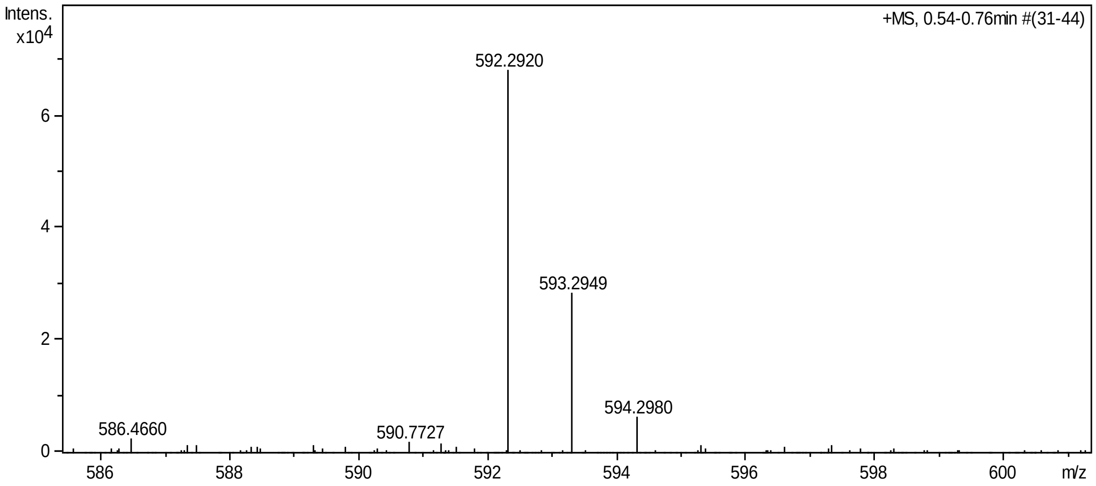
Intens.
x10^4
+MS, 0.54-0.76min #(31-44)
592.2920
593.2949
594.2980
586.4660
590.7727
m/z

***rel*-(1*R*,3*R*)-5'-Methyl-6-((*Z*)-4-methylbenzylidene)-2',5-dioxo-3-(*p*-tolyl)spiro[cyclohexane-1,3'-indoline]-2,2-dicarbonitrile (3g)**: white solid, 45%, m.p. 252-254 °C; ^{1}H NMR (400 MHz, $CDCl_3$) δ 8.26 (s, 1H, NH), 7.64 (s, 1H, ArH), 7.42 (d, *J* = 8.0 Hz, 2H, ArH), 7.25-7.21 (m, 5H, ArH), 7.12 (d, *J* = 8.0 Hz, 2H, ArH), 6.96 (d, *J* = 8.0 Hz, 1H, ArH), 6.68 (s, 1H, CH), 4.93-4.88 (m, 1H, CH_2), 3.50-3.42 (m, 1H, CH), 3.08-3.03 (m, 1H, CH_2), 2.41 (s, 3H, CH_3), 2.37 (s, 3H, CH_3), 2.33 (s, 3H, CH_3). ^{13}C NMR (100 MHz, $CDCl_3$) δ 197.3, 173.1, 141.0, 139.8, 139.6, 138.6, 133.8, 132.0, 131.6, 130.9, 129.8, 129.6, 129.2, 129.1, 128.8, 127.2, 123.5, 112.2, 112.0, 111.4, 60.7, 49.1, 44.4, 42.4, 21.4, 21.4, 21.2. IR (KBr) υ: 3311, 3035, 2920, 1901, 1726, 1615, 1450, 1449, 1419, 1359, 1305, 1197, 1052, 959, 888, 818, 746 cm^{-1}; MS (*m*/*z*): HRMS (ESI) Calcd. for $C_{31}H_{25}NaN_3O_2$ ($[M+Na]^+$): 494.1844, found: 494.1830.

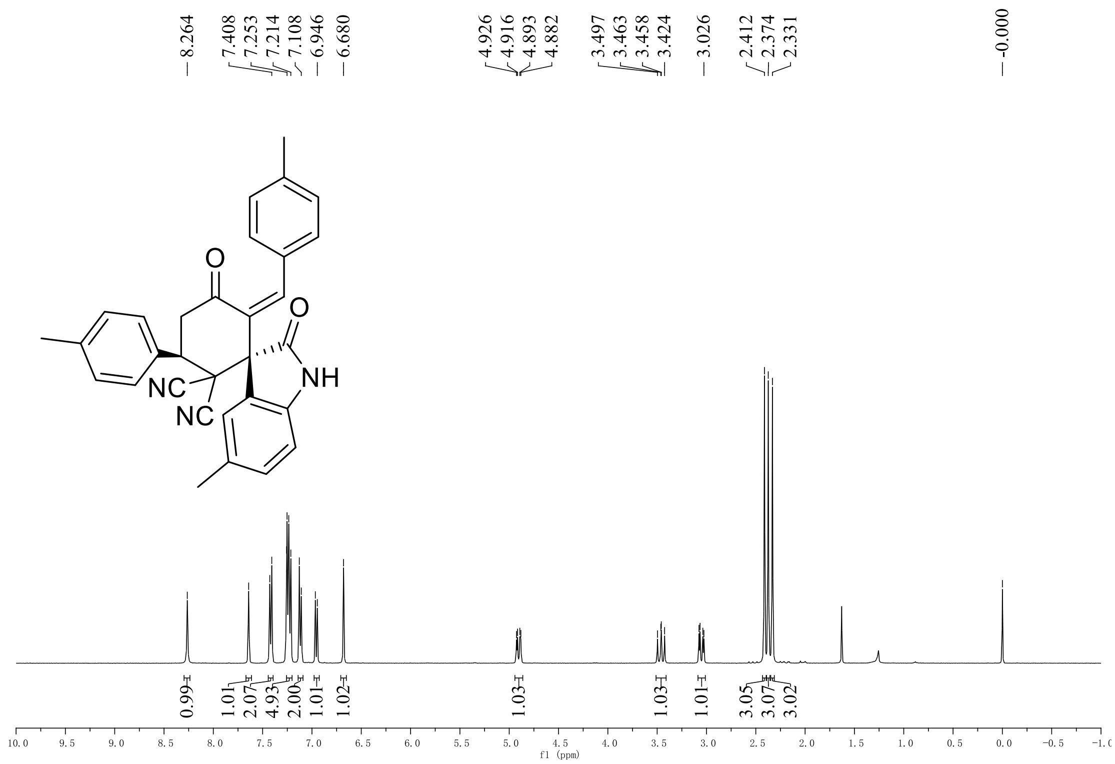

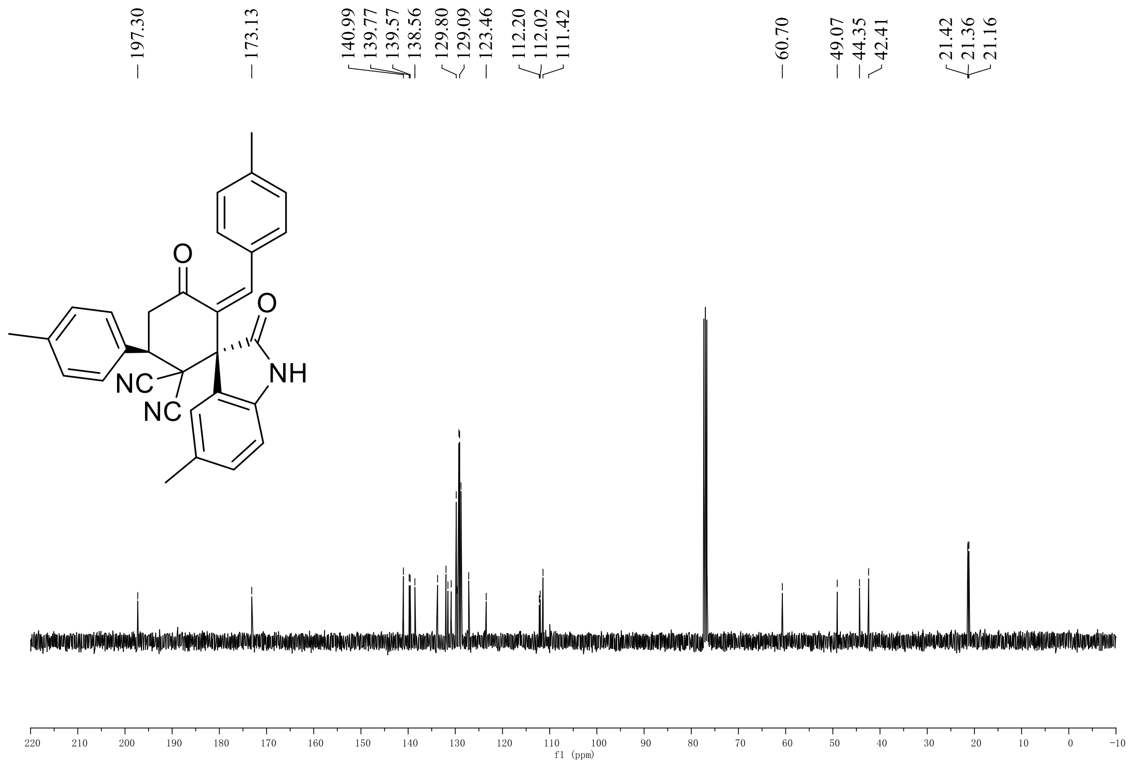

197.30
173.13
140.99
139.77
139.57
138.56
129.80
129.09
123.46
112.20
112.02
111.42
60.70
49.07
44.35
42.41
21.42
21.36
21.16
O
O
NH
NC
NC
220 210 200 190 180 170 160 150 140 130 120 110 100 90 80 70 60 50 40 30 20 10 0 −10
f1 (ppm)

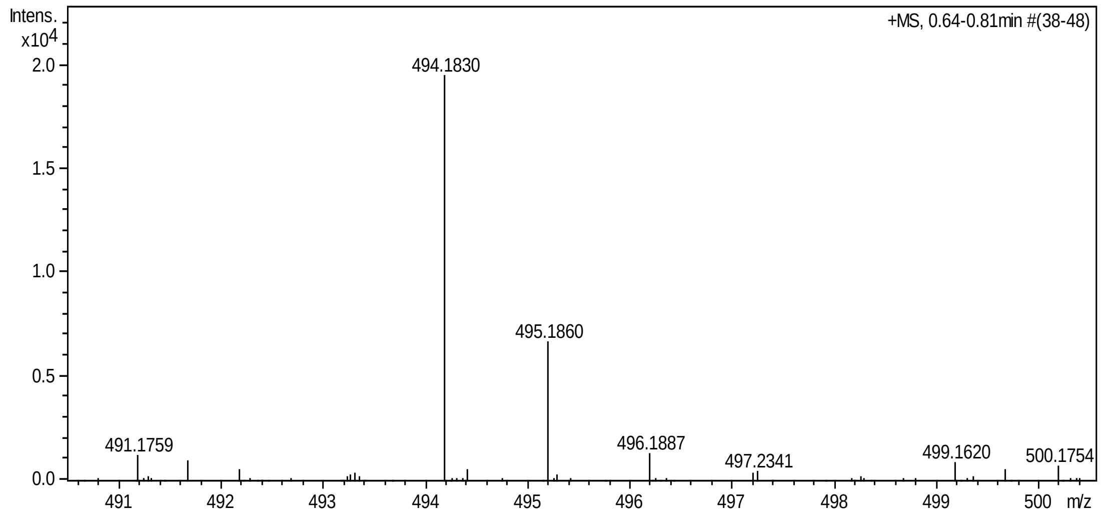

Intens.
x10^4
+MS, 0.64-0.81min #(38-48)
494.1830
495.1860
491.1759
496.1887
497.2341
499.1620
500.1754
2.0
1.5
1.0
0.5
0.0
491 492 493 494 495 496 497 498 499 500 m/z

***rel*-(1*R*,3*R*)-6-((*Z*)-4-(*tert*-Butyl)benzylidene)-3-(4-(*tert*-butyl)phenyl)-5'-methyl-2',5-dioxospiro[cyclohexane-1,3'-indoline]-2,2-dicarbonitrile (3h)**: pale yellow solid, 49%, m.p. 238-240 °C; ^{1}H NMR (400 MHz, $CDCl_3$) δ 7.84 (s, 1H, NH), 7.65 (s, 1H, ArH), 7.45 (s, 4H, ArH), 7.34 (d, *J* = 8.4 Hz, 2H, ArH), 7.29 (d, *J* = 8.4 Hz, 3H, ArH), 6.97 (d, *J* = 8.0 Hz, 1H, ArH), 6.67 (s, 1H, CH), 4.93-4.89 (m, 1H, CH_2), 3.51-3.43 (m, 1H, CH), 3.09-3.04 (m, 1H, CH_2), 2.41 (s, 3H, CH_3), 1.33 (s, 9H, $(CH_3)_3$), 1.30 (s, 3H, $(CH_3)_3$). ^{13}C NMR (100 MHz, $CDCl_3$) δ 197.4, 173.1, 152.9, 152.6 140.9, 138.5, 133.8, 132.0, 131.4, 130.8, 129.5, 129.2, 128.6, 127.2, 126.1, 125.4, 123.5, 112.2, 112.0, 111.4, 60.8, 49.0, 44.3, 42.4, 34.8, 34.7, 31.2, 31.1, 21.4. IR (KBr) υ: 3684, 3288, 2964, 1728, 1614, 1500, 1358, 1307, 1266, 1207, 1112, 1058, 1021, 968, 830 cm^{-1}; MS (*m*/*z*): HRMS (ESI) Calcd. for $C_{37}H_{37}NaN_3O_2$ ($[M+Na]^+$): 578.2783, found: 578.2766.

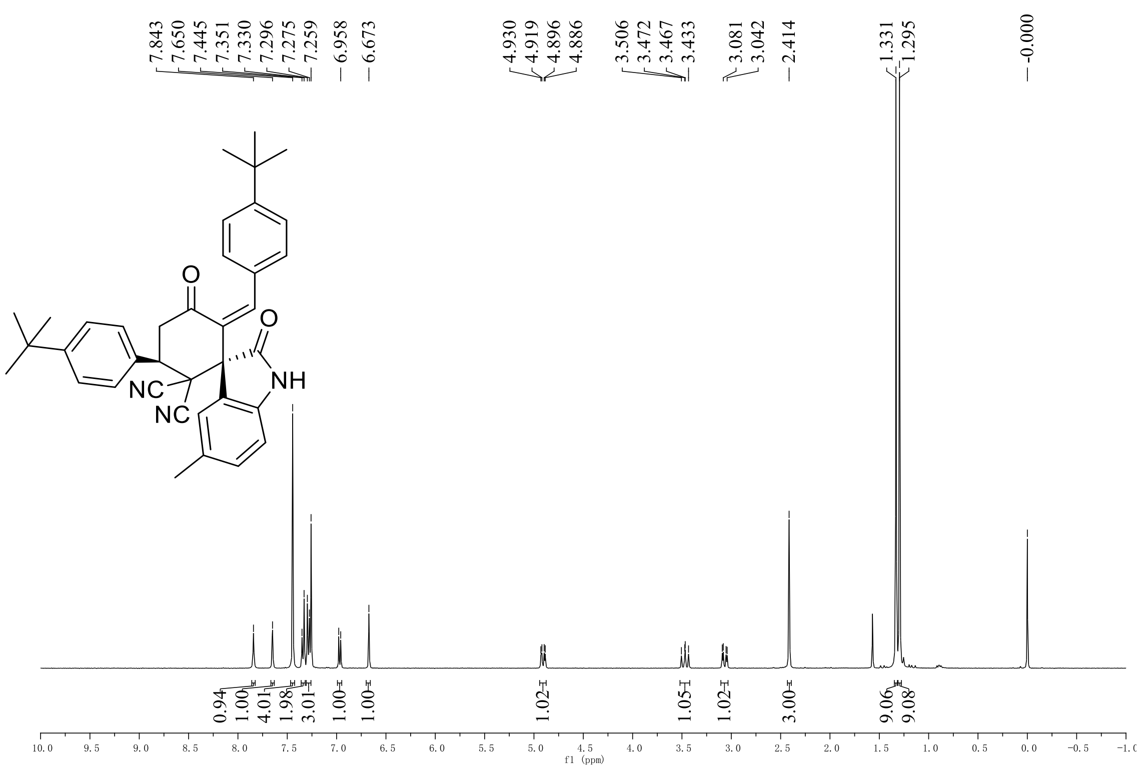

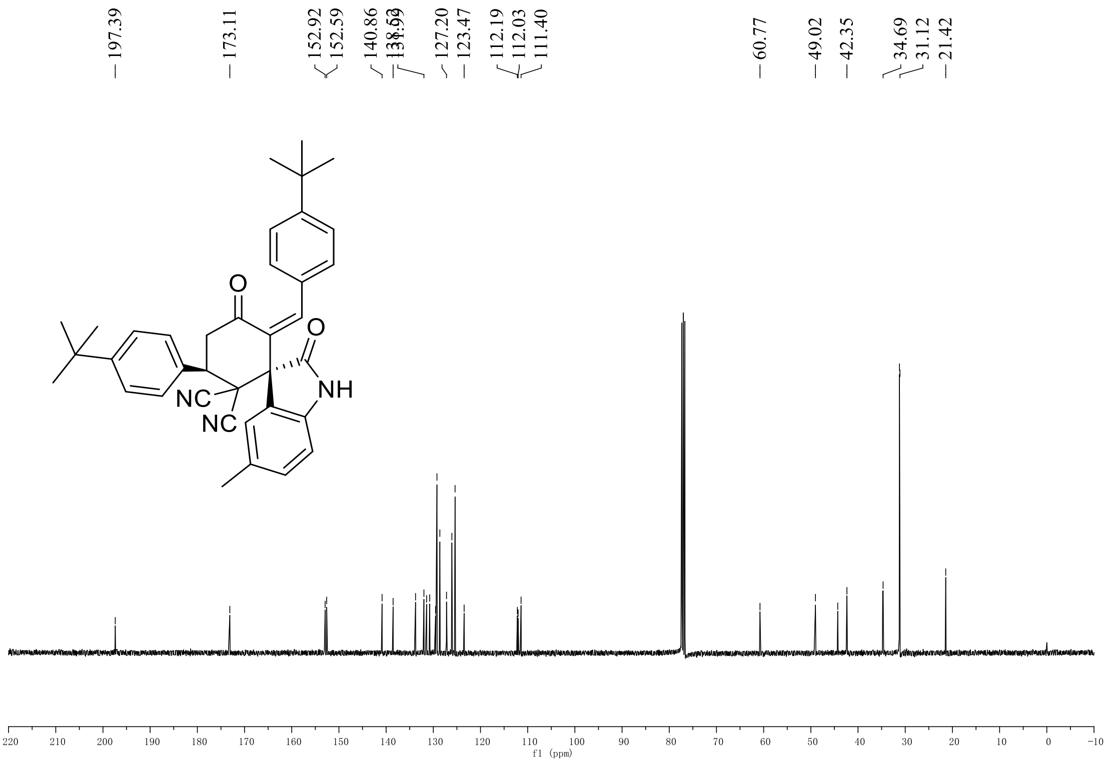

197.39
173.11
152.92
152.59
140.86
127.20
123.47
112.19
112.03
111.40
60.77
49.02
42.35
34.69
31.12
21.42
O
O
NH
NC
NC
f1 (ppm)

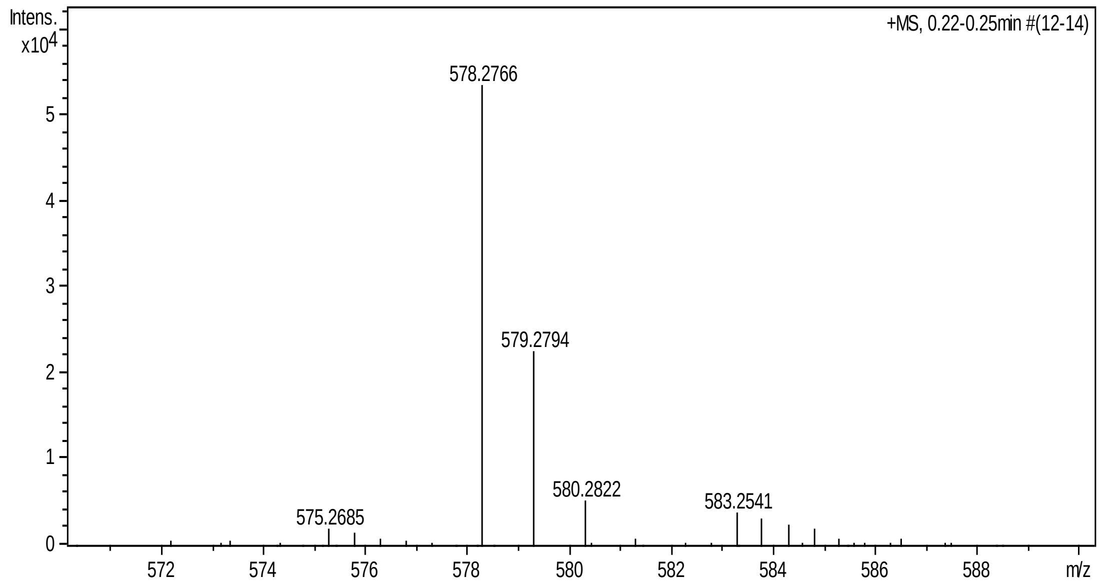

Intens.
x10^4
+MS, 0.22-0.25min #(12-14)
578.2766
579.2794
580.2822
583.2541
575.2685
m/z

***rel*-(1*R*,3*R*)-6-((*Z*)-4-Bromobenzylidene)-3-(4-bromophenyl)-1'-butyl-5'-methyl-2',5-dioxospiro[cyclohexane-1,3'-indoline]-2,2-dicarbonitrile (3i)**: white solid, 54%, m.p. 170-172 °C; ^{1}H NMR (400 MHz, $CDCl_3$) δ 7.66 (s, 1H, ArH), 7.59 (d, *J* = 8.0 Hz, 2H, ArH), 7.43-7.38 (m, 4H, ArH), 7.34 (d, *J* = 8.0 Hz, 1H, ArH), 7.19 (d, *J* = 8.0 Hz, 2H, ArH), 6.96 (d, *J* = 8.0 Hz, 1H, ArH), 6.61 (s, 1H, CH), 5.00-4.96 (m, 1H, CH_2), 3.91-3.84 (m, 1H, CH_2), 3.66-3.58 (m, 1H, CH_2), 3.35 (t, *J* = 14.0 Hz, 1H, CH), 3.07-3.02 (m, 1H, CH_2), 2.45 (s, 3H, CH_3), 1.75-1.68 (m, 2H, CH_2), 1.45-1.40 (m, 2H, CH_2), 0.97 (t, *J* = 7.2 Hz, 3H, CH_3). ^{13}C NMR (100 MHz, $CDCl_3$) δ 195.7, 171.1, 141.0, 139.0, 133.9, 133.5, 132.6, 132.4, 132.2, 131.5, 130.8, 130.6, 127.0, 124.0, 123.7, 122.7, 112.0, 111.7, 110.1, 60.0, 48.7, 44.0, 42.2, 40.7, 29.2, 21.4, 20.1, 13.6. IR (KBr) υ: 3094, 2934, 2868, 1701, 1615, 1592, 1495, 1436, 1352, 1329, 1271, 1238, 1198, 1164, 1120, 1079, 1055, 991, 916, 857, 837, 815, 736, 713 cm^{-1}; MS (*m*/*z*): HRMS (ESI) Calcd. for $C_{33}H_{27}NaBr_2N_3O_2$ ([M+Na]$^+$): 678.0368, found: 678.0340.

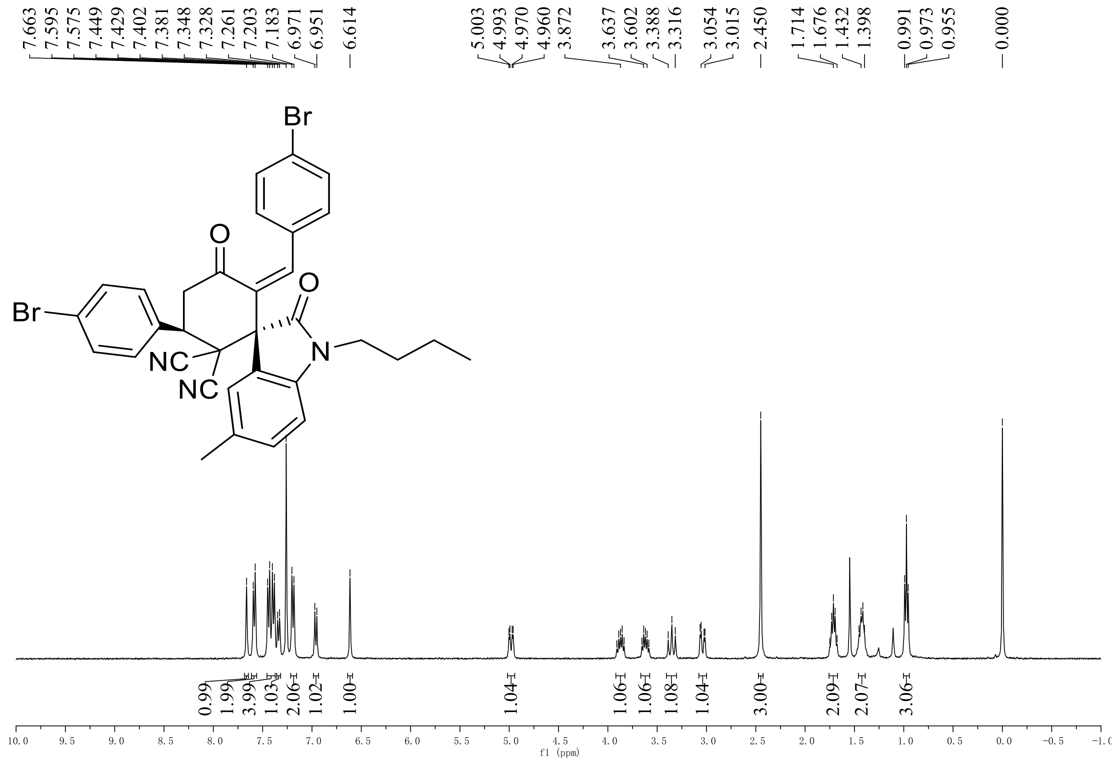

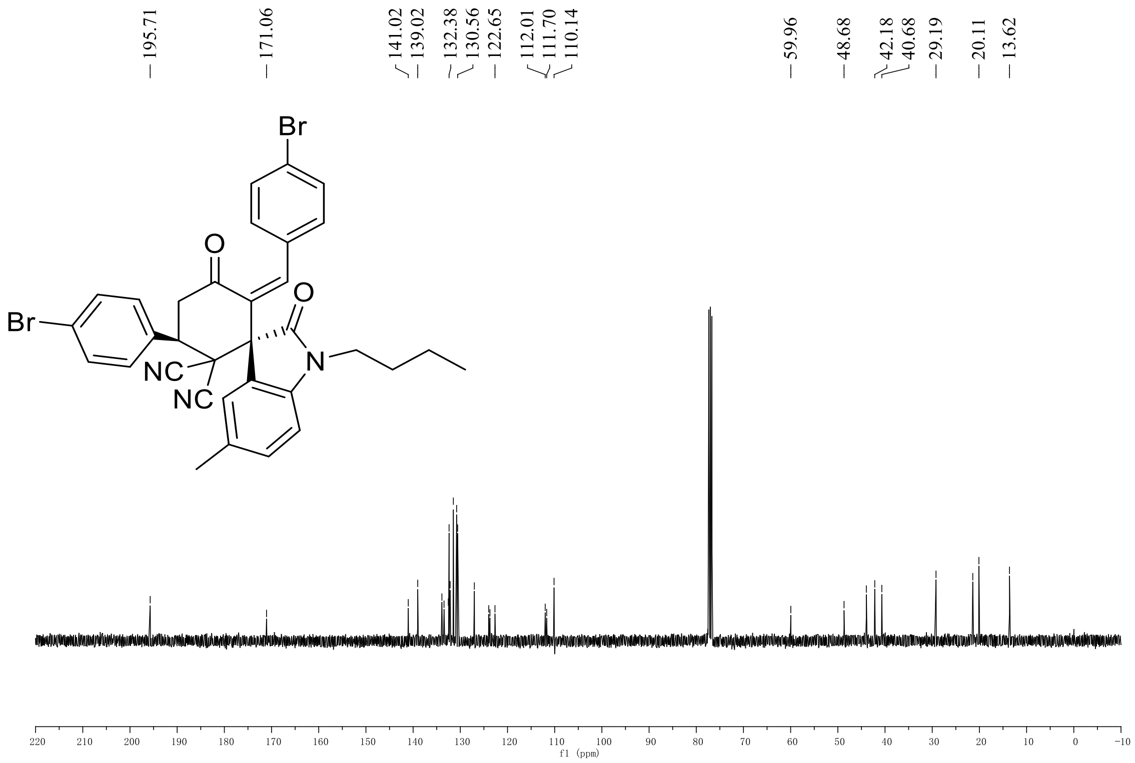
195.71
171.06
141.02
139.02
132.38
130.56
122.65
112.01
111.70
110.14
59.96
48.68
42.18
40.68
29.19
20.11
13.62
Br
O
O
Br
NC
NC
N
f1 (ppm)

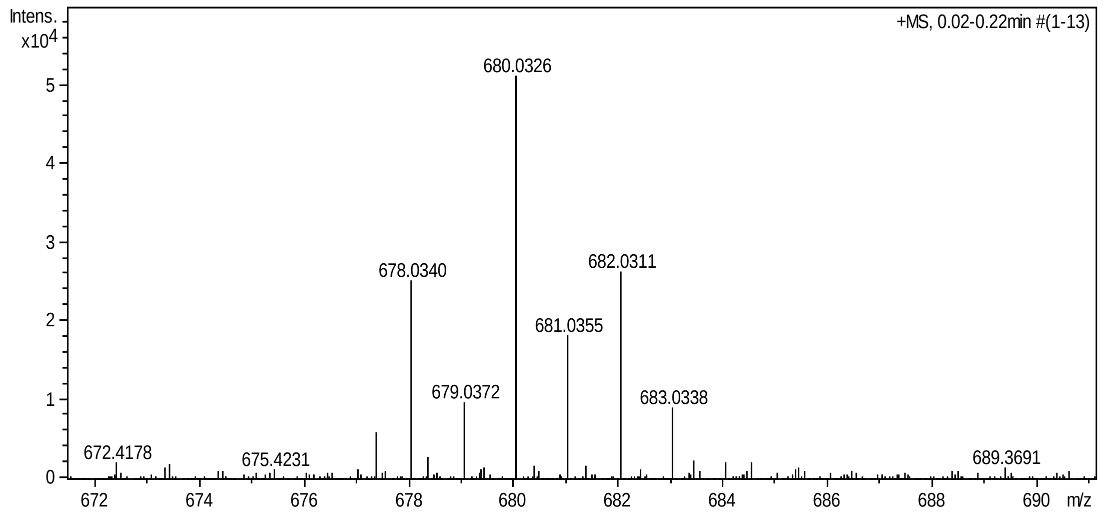
Intens.
x10^4
+MS, 0.02-0.22min #(1-13)
680.0326
678.0340
682.0311
681.0355
679.0372
683.0338
672.4178
675.4231
689.3691
m/z

***rel*-(1*R*,3*R*)-1'-Butyl-5'-methyl-6-((*Z*)-4-methylbenzylidene)-2',5-dioxo-3-(*p*-tolyl)spiro[cyclohexane-1,3'-indoline]-2,2-dicarbonitrile (3j)**: white solid, 54%, m.p. 214-216 °C; ^{1}H NMR (400 MHz, $CDCl_3$) δ 7.68 (s, 1H, ArH), 7.41 (d, *J* = 8.4 Hz, 2H, ArH), 7.31 (d, *J* = 8.0 Hz, 1H, ArH), 7.24-7.22 (m, 4H, ArH), 7.11 (d, *J* = 8.0 Hz, 2H, ArH), 6.94 (d, *J* = 8.0 Hz, 1H, ArH), 6.64 (s, 1H, CH), 4.99-4.94 (m, 1H, CH_2), 3.90-3.83 (m, 1H, CH_2), 3.65-3.58 (m, 1H, CH_2), 3.46-3.39 (m, 1H, CH), 3.07-3.02 (m, 1H, CH_2), 2.43 (s, 3H, CH_3), 2.37 (s, 3H, CH_3), 2.33 (s, 3H, CH_3), 1.75-1.68 (m, 2H, CH_2), 1.46-1.39 (m, 2H, CH_2), 0.97 (t, *J* = 7.2 Hz, 3H, CH_3). ^{13}C NMR (100 MHz, $CDCl_3$) δ 196.8, 171.4, 141.1, 140.1, 139.7, 139.5, 133.6, 131.8, 131.7, 130.9, 130.0, 129.8, 129.3, 129.0, 128.8, 127.2, 123.2, 112.3, 112.1, 109.9, 60.1, 49.2, 44.4, 42.5, 40.6, 29.2, 21.4, 21.3, 21.2, 20.1, 13.6. IR (KBr) υ: 3030, 2956, 2869, 1710, 1611, 1502, 1424, 1358, 1195, 1149, 1052, 939, 817, 738 cm^{-1}; MS (*m/z*): HRMS (ESI) Calcd. for $C_{35}H_{33}NaN_3O_2$ ([M+Na]$^+$): 550.2470, found: 550.2454.

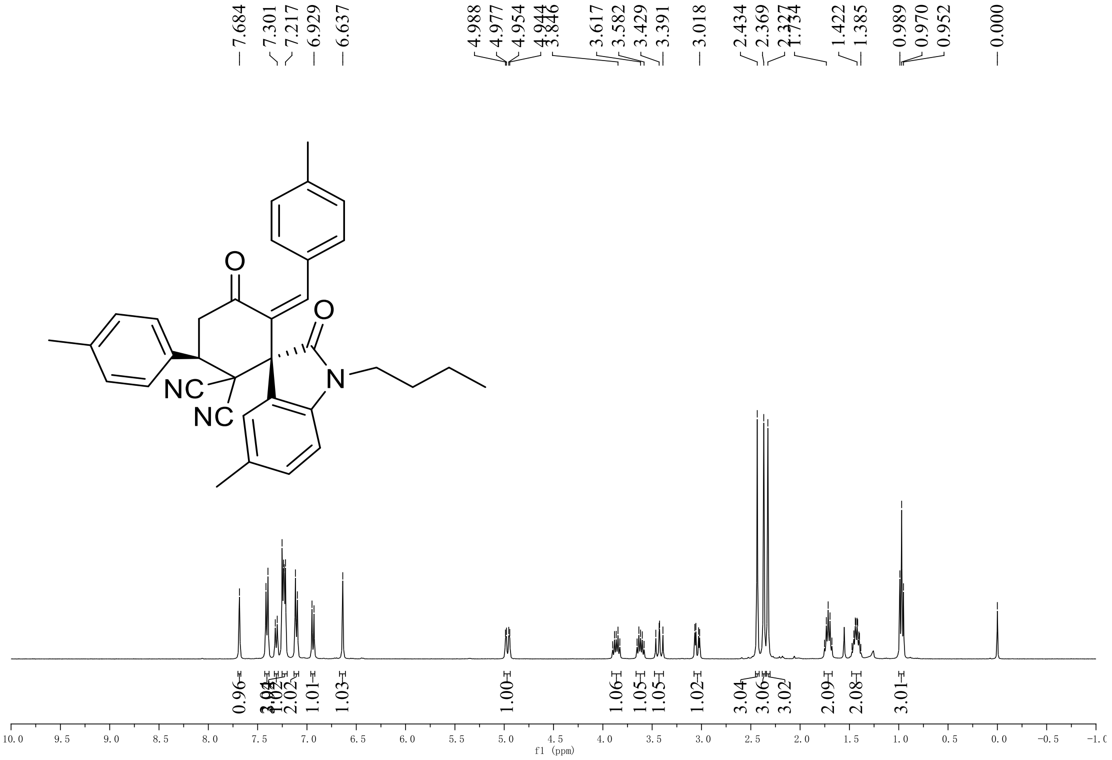

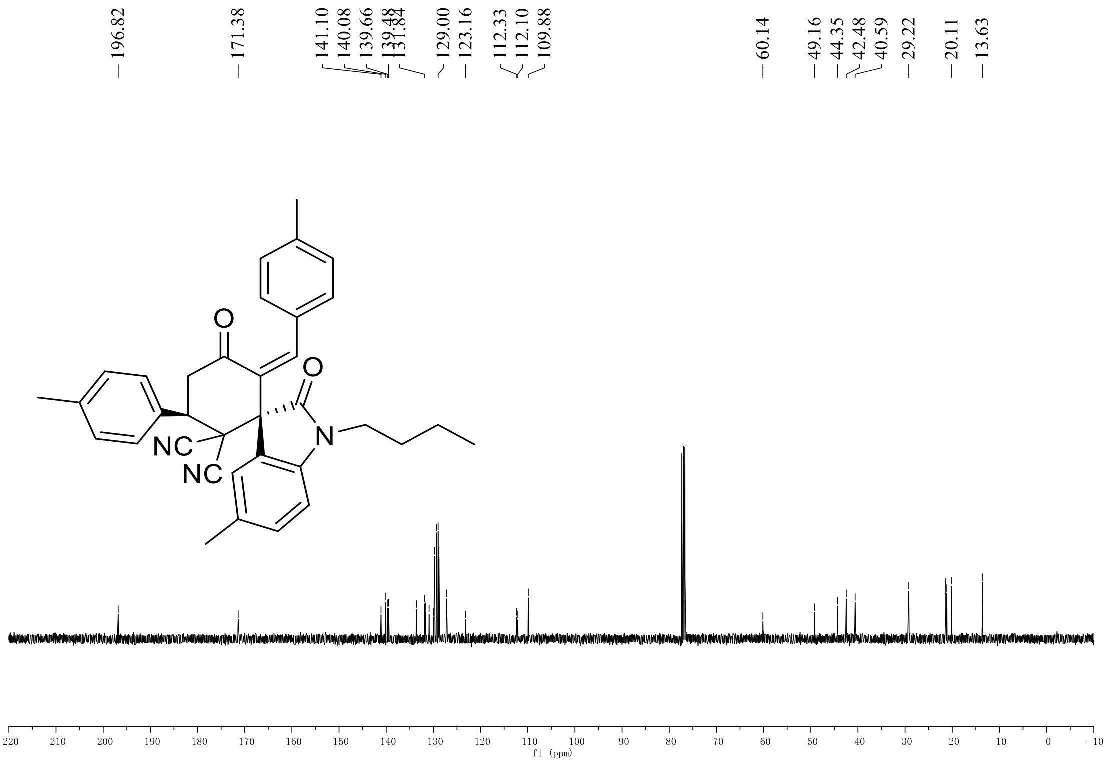
196.82
171.38
141.10
140.08
139.66
139.48
131.84
129.00
123.16
112.33
112.10
109.88
60.14
49.16
44.35
42.48
40.59
29.22
20.11
13.63
O
O
N
NC
NC
f1 (ppm)

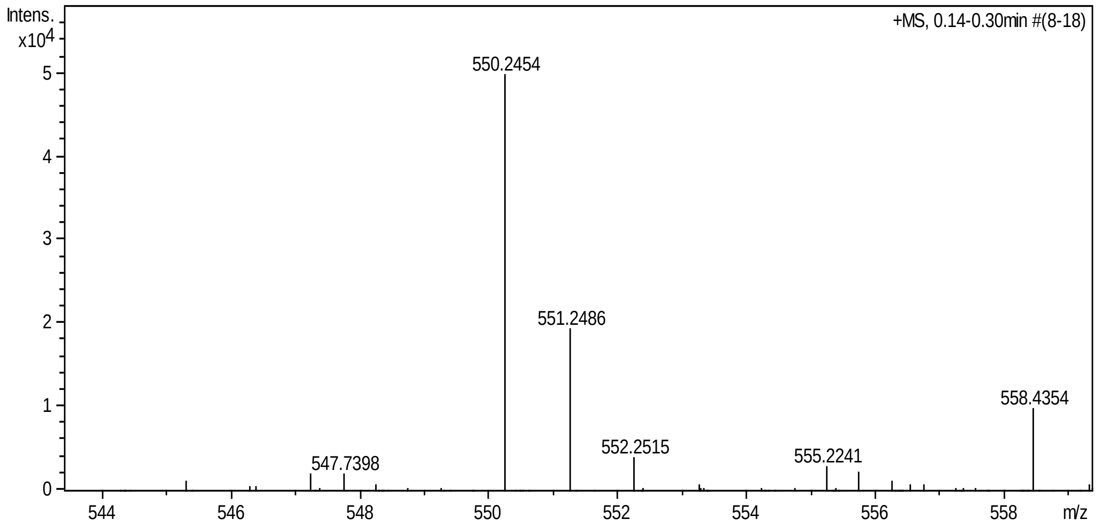
Intens.
x10^4
+MS, 0.14-0.30min #(8-18)
550.2454
551.2486
558.4354
552.2515
555.2241
547.7398
m/z

***rel*-(1*R*,3*R*)-1'-Butyl-6-((*Z*)-4-(*tert*-butyl)benzylidene)-3-(4-(*tert*-butyl)phenyl)-5'-methyl-2',5-dioxospiro[cyclohexane-1,3'-indoline]-2,2-dicarbonitrile (3k)**: white solid, 58%, m.p. 143-145 °C; ^{1}H NMR (400 MHz, $CDCl_3$) δ 7.68 (s, 1H, ArH), 7.44 (s, 4H, ArH), 7.34-7.27 (m, 5H, ArH), 6.95 (d, *J* = 8.0 Hz, 1H, ArH), 6.64 (s, 1H, CH), 4.99-4.95 (m, 1H, CH_2), 3.90-3.83 (m, 1H, CH_2), 3.67-3.60 (m, 1H, CH_2), 3.49-3.42 (m, 1H, CH), 3.09-3.04 (m, 1H, CH_2), 2.43 (s, 3H, CH_3), 1.76-1.68 (m, 2H, CH_2), 1.48-1.40 (m, 2H, CH_2), 1.33 (s, 9H, $(CH_3)_3$), 1.29 (s, 9H, $(CH_3)_3$), 0.97 (t, *J* = 7.2 Hz, 3H, CH_3). ^{13}C NMR (100 MHz, $CDCl_3$) δ 197.0, 171.4, 152.8, 152.5, 141.1, 140.0, 133.6, 131.8, 131.6, 130.8, 130.0, 129.3, 128.6, 127.2, 126.0, 125.3, 123.2, 112.3, 112.1, 109.9, 60.2, 49.1, 44.3, 42.4, 40.6, 34.8, 34.7, 31.2, 31.1, 29.2, 21.4, 20.1, 13.7. IR (KBr) υ: 2963, 2872, 1711, 1608, 1500, 1462, 1423, 1360, 1264, 1197, 1151, 1114, 1058, 932, 831 cm^{-1}; MS (*m*/*z*): HRMS (ESI) Calcd. for $C_{41}H_{45}NaN_3O_2$ ($[M+Na]^+$): 634.3409, found: 634.3394.

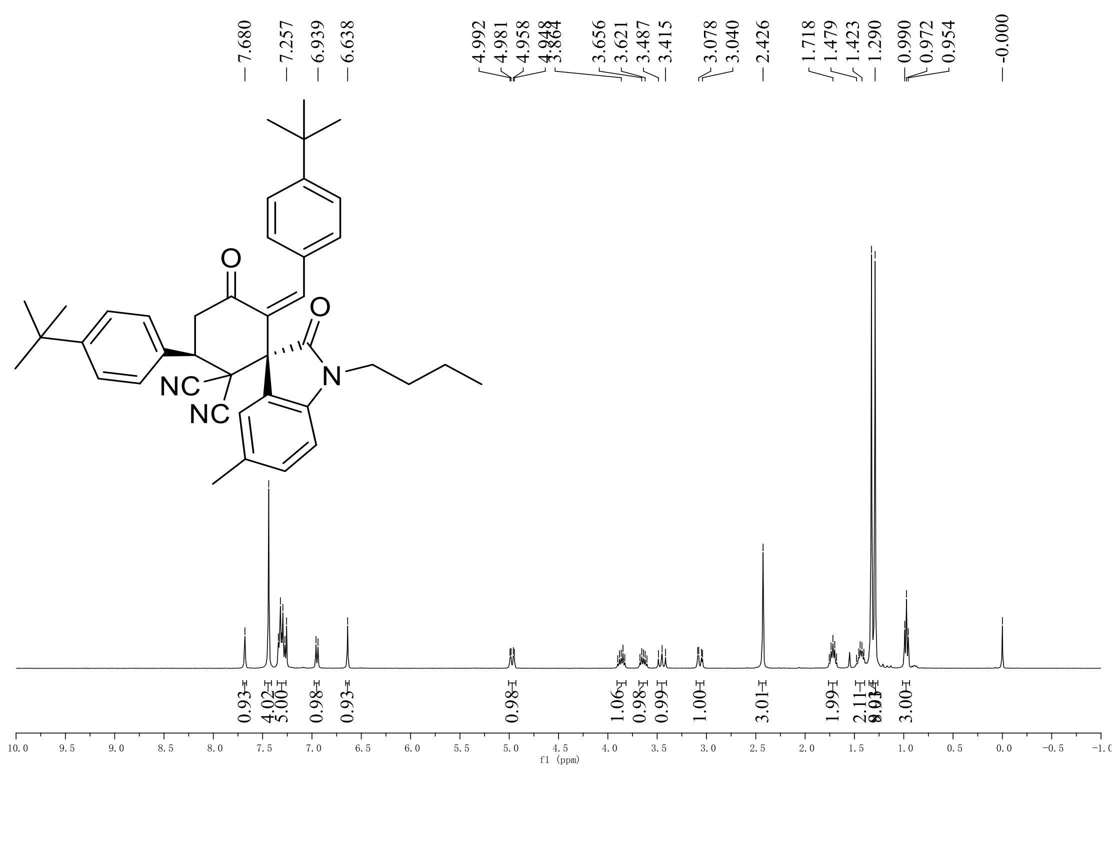

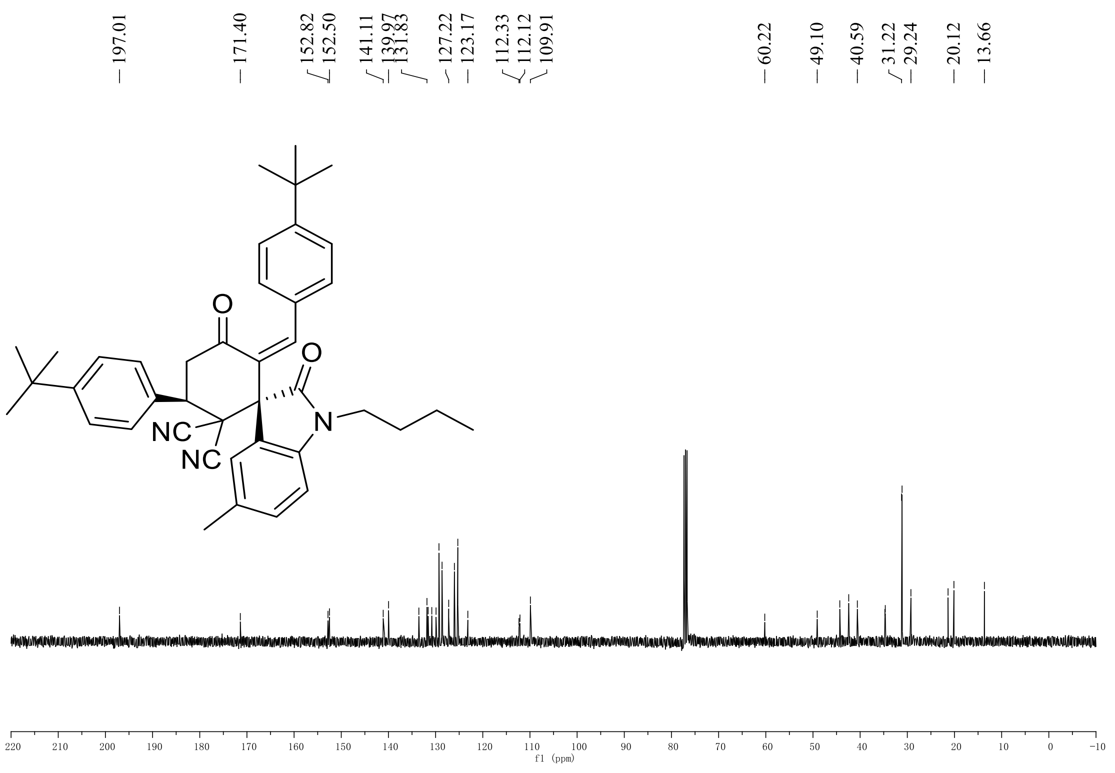
197.01
171.40
152.82
152.50
141.11
139.97
131.83
127.22
123.17
112.33
112.12
109.91
60.22
49.10
40.59
31.22
29.24
20.12
13.66
O
O
N
NC
NC
220 210 200 190 180 170 160 150 140 130 120 110 100 90 80 70 60 50 40 30 20 10 0 −10
f1 (ppm)

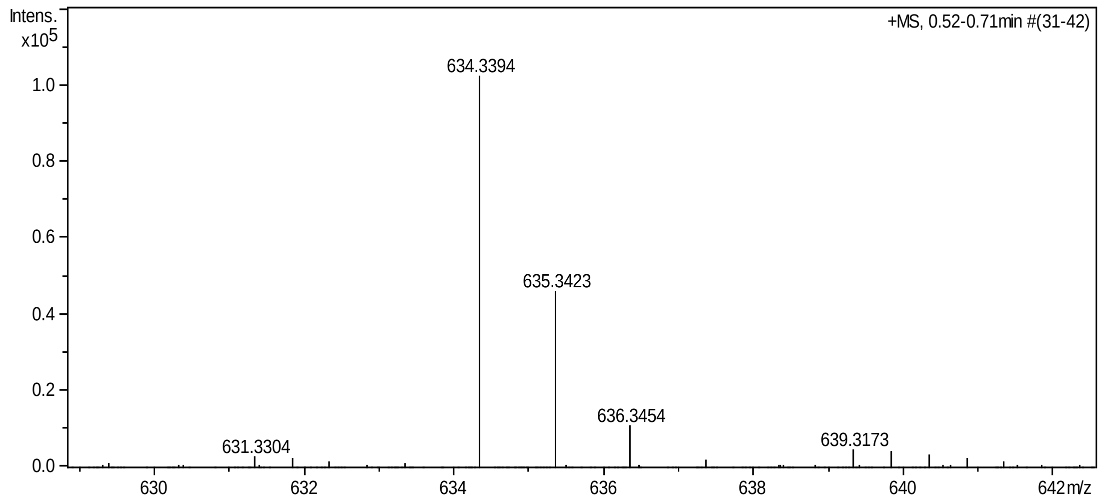
Intens.
x10^5
+MS, 0.52-0.71min #(31-42)
634.3394
635.3423
636.3454
631.3304
639.3173
1.0
0.8
0.6
0.4
0.2
0.0
630 632 634 636 638 640 642
m/z

***rel*-(1*R*,3*R*)-1'-Benzyl-5'-chloro-6-((*Z*)-4-methylbenzylidene)-2',5-dioxo-3-(*p*-tolyl)spiro[cyclohexane-1,3'-indoline]-2,2-dicarbonitrile (3l)**: white solid, 54%, m.p. 235-237 °C; ^{1}H NMR (400 MHz, $CDCl_3$) δ 7.89 (s, 1H, ArH), 7.42 (d, *J* = 8.0 Hz, 2H, ArH), 7.39-7.30 (m, 7H, ArH), 7.27 (s, 1H, ArH), 7.23 (s, 2H, ArH), 7.13 (d, *J* = 8.0 Hz, 2H, ArH), 6.80 (d, *J* = 8.8 Hz, 1H, ArH), 6.60 (s, 1H, CH), 5.09 (d, *J* = 15.6 Hz, 1H, CH_2), 5.01-4.96 (m, 1H, CH_2), 4.86 (d, *J* = 15.6 Hz, 1H, CH_2), 3.47 (t, *J* = 14.0 Hz, 1H, CH), 3.12-3.07 (m, 1H, CH_2), 2.38 (s, 3H, CH_3), 2.35 (s, 3H, CH_3). ^{13}C NMR (100 MHz, $CDCl_3$) δ 196.3, 171.4, 141.7, 140.5, 140.1, 139.7, 133.8, 131.6, 131.3, 130.5, 129.9, 129.7, 129.4, 129.3, 129.1, 128.8, 128.2, 127.1, 126.83, 124.6, 112.1, 112.0, 60.3, 48.8, 44.8, 44.3, 42.6, 21.4, 21.2. IR (KBr) υ: 3728, 3407, 3033, 2919, 2318, 1899, 1776, 1717, 1611, 1485, 1425, 1349, 1259, 1188, 1083, 1020, 957, 883, 816, 724 cm^{-1}; MS (*m*/*z*): HRMS (ESI) Calcd. for $C_{37}H_{28}NaClN_3O_2$ ([M+Na]$^{+}$): 604.1768, found: 604.1754.

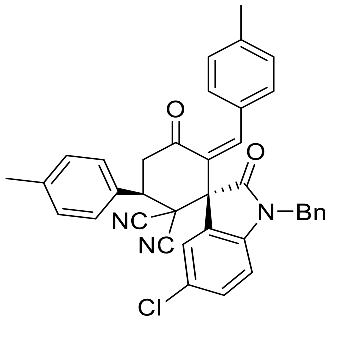

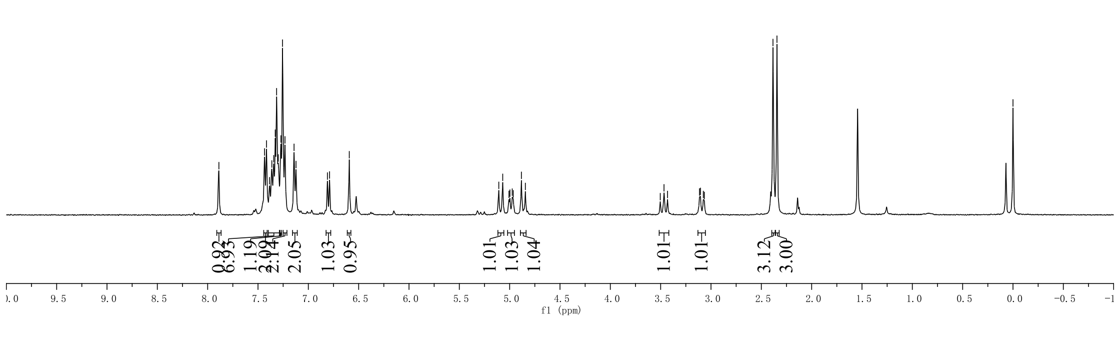

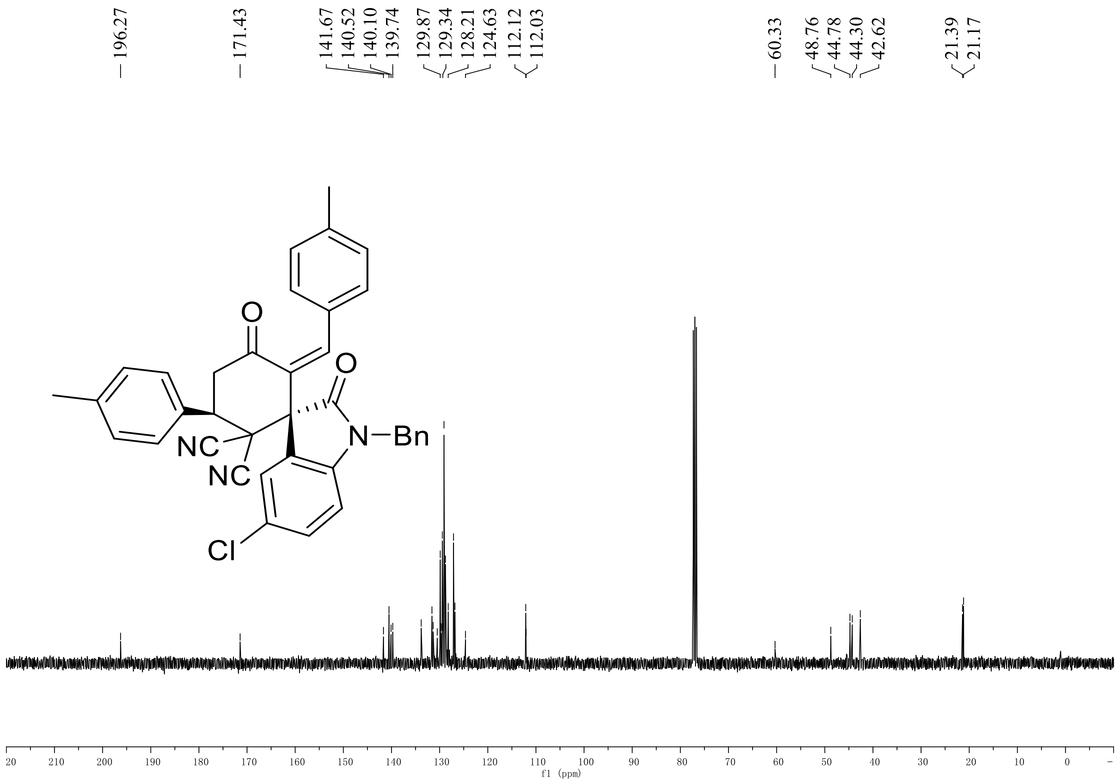
196.27
171.43
141.67
140.52
140.10
139.74
129.87
129.34
128.21
124.63
112.12
112.03
60.33
48.76
44.78
44.30
42.62
21.39
21.17
O
O
NC
NC
N−Bn
Cl
f1 (ppm)

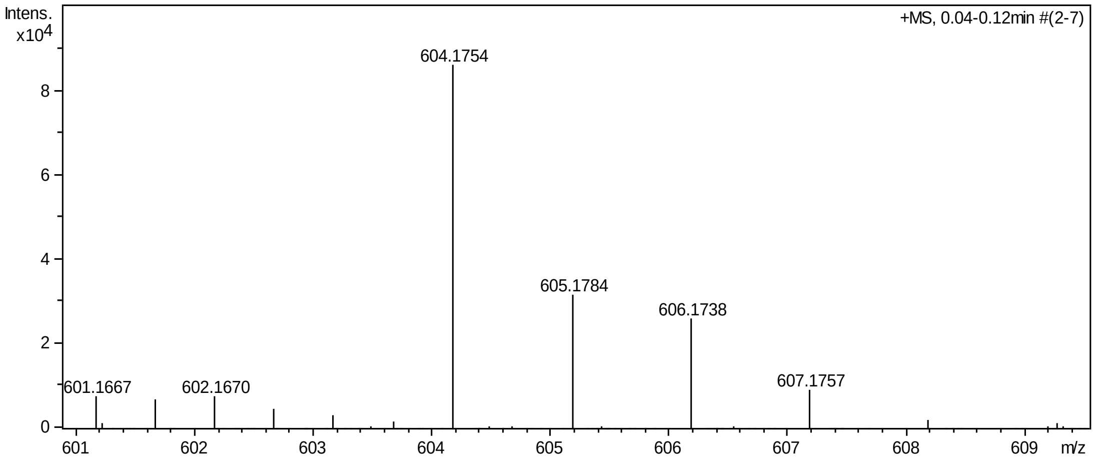
Intens.
x10^4
+MS, 0.04-0.12min #(2-7)
604.1754
605.1784
606.1738
607.1757
601.1667
602.1670
m/z

***rel*-(1*R*,3*R*)-1'-Benzyl-6-((*Z*)-4-(*tert*-butyl)benzylidene)-3-(4-(*tert*-butyl)phenyl)-5'-chloro-2',5-dioxospiro[cyclohexane-1,3'-indoline]-2,2-dicarbonitrile (3m)**: white solid, 60%, m.p. 184-186 °C; ^{1}H NMR (400 MHz, $CDCl_3$) δ 7.88 (s, 1H, ArH), 7.46 (s, 4H, ArH), 7.39-7.29 (m, 10H, ArH), 6.81 (d, J = 8.4 Hz, 1H, ArH), 6.60 (s, 1H, CH), 5.08 (d, J = 16.0 Hz, 1H, CH_2), 5.01-4.97 (m, 1H, CH_2), 4.99 (d, J = 15.6 Hz, 1H, CH_2), 3.49 (t, J = 13.6 Hz, 1H, CH), 3.13-3.08 (m, 1H, CH_2), 1.34 (s, 9H, $(CH_3)_3$), 1.30 (s, 9H, $(CH_3)_3$).^{13}C NMR (100 MHz, $CDCl_3$) δ 196.4, 171.5, 153.3, 152.8, 141.7, 140.4, 133.9, 131.6, 131.2, 130.4, 129.7, 129.4, 129.3, 129.1, 128.6, 128.2, 127.1, 126.8, 126.1, 125.4, 124.7, 112.1, 60.4, 48.7, 44.8, 44.3, 42.6, 34.8, 34.7, 31.2, 31.1, 26.9. IR (KBr) υ: 3685, 3062, 2962, 1718, 1607, 1486, 1424, 1354, 1265, 1189, 1114, 1019, 958, 832 cm^{-1}; MS (m/z): HRMS (ESI) Calcd. for $C_{43}H_{40}NaClN_3O_2$ ($[M+Na]^+$): 688.2707, found: 688.2677.

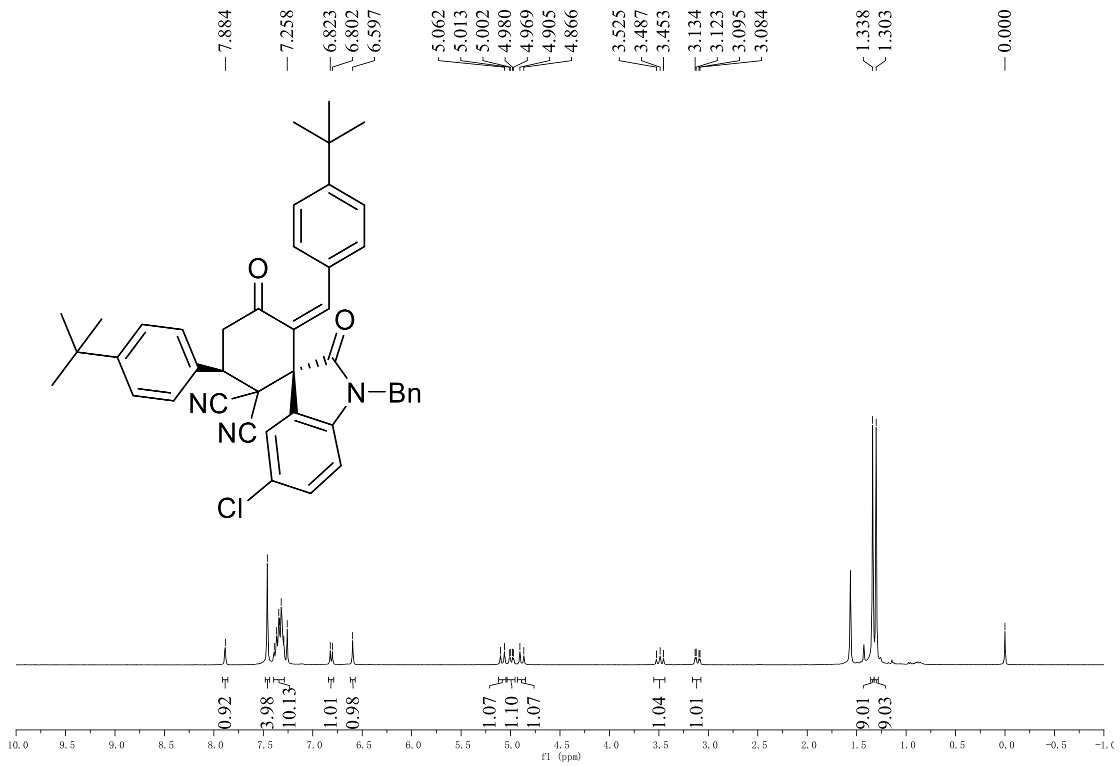

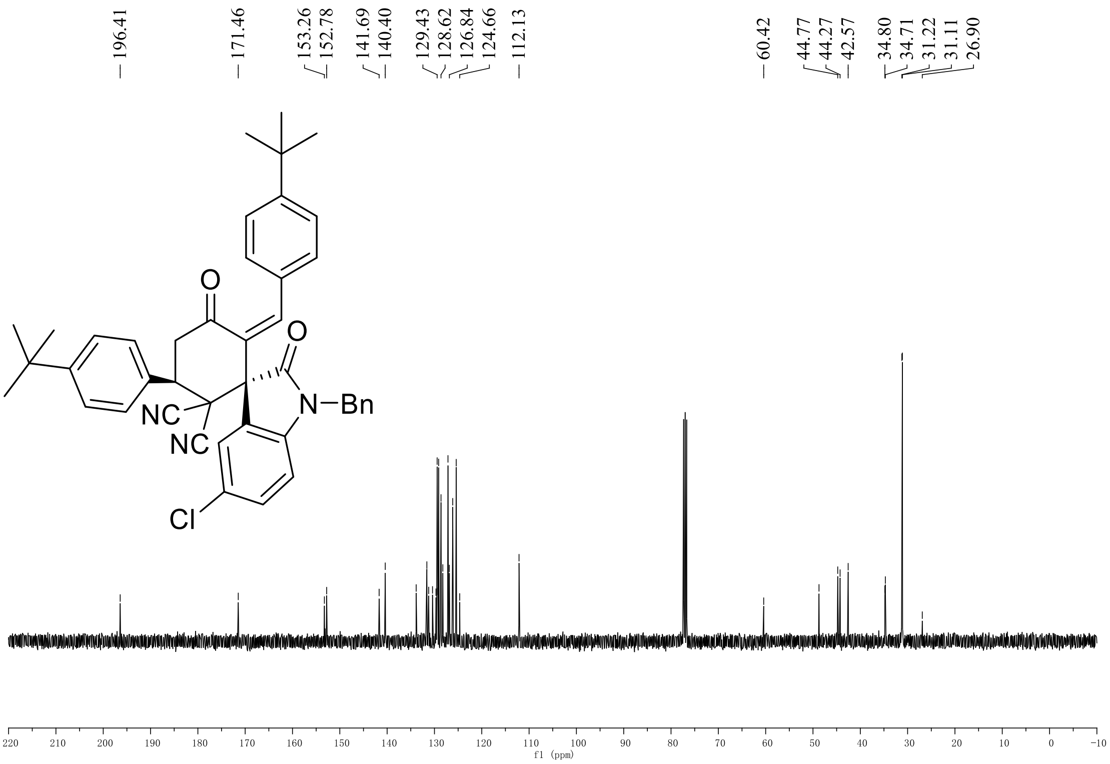
196.41
171.46
153.26
152.78
141.69
140.40
129.43
128.62
126.84
124.66
112.13
60.42
44.77
44.27
42.57
34.80
34.71
31.22
31.11
26.90
O
O
N−Bn
NC
NC
Cl
f1 (ppm)

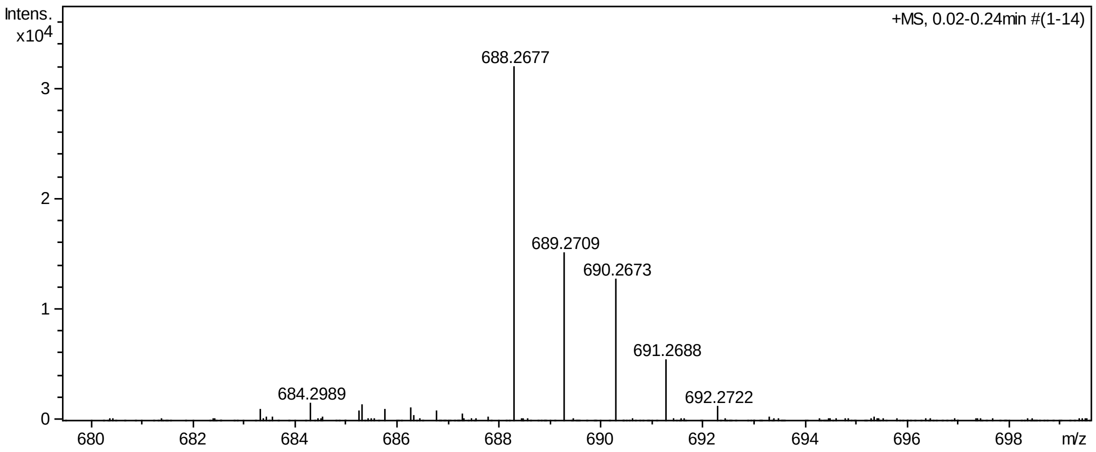
Intens.
x10^4
+MS, 0.02-0.24min #(1-14)
688.2677
689.2709
690.2673
691.2688
692.2722
684.2989
m/z

***rel*-(1*R*,3*R*)-1'-Benzyl-5'-chloro-6-((*Z*)-4-isopropylbenzylidene)-3-(4-isopropylphenyl)-2',5-dioxospiro[cyclohexane-1,3'-indoline]-2,2-dicarbonitrile (3n)**: white solid, 56%, m.p. 242-244 °C; ^{1}H NMR (400 MHz, $CDCl_3$) δ 7.89 (s, 1H, ArH), 7.45 (d, *J* = 7.6 Hz, 2H, ArH), 7.38-7.30 (m, 10H, ArH), 7.19 (d, *J* = 7.6 Hz, 2H, ArH), 6.81 (d, *J* = 8.4 Hz, 1H, ArH), 6.59 (s, 1H, CH), 5.08 (d, *J* = 16.0 Hz, 1H, CH_2), 4.99 (d, *J* = 12.8 Hz, 1H, CH_2), 4.88 (d, *J* = 15.2 Hz, 1H, CH_2), 3.48 (t, *J* = 14.4 Hz, 1H, CH), 3.10 (d, *J* = 15.2 Hz, 1H, CH_2), 2.96-2.88 (m, 2H, CH), 1.27 (d, *J* = 6.4 Hz, 6H, $(CH_3)_2$), 1.23 (d, *J* = 6.8 Hz, 6H, $(CH_3)_2$). ^{13}C NMR (100 MHz, $CDCl_3$) δ 196.4, 171.5, 151.0, 150.5, 141.7, 140.5, 133.9, 131.6, 130.8, 129.7, 129.2, 129.1, 128.9, 128.2, 127.3, 127.1, 126.8, 126.5, 124.7, 112.1, 112.0, 60.4, 48.8, 44.8, 44.3, 42.7, 34.0, 33.8, 23.8, 23.8, 23.7. IR (KBr) υ: 3035, 2965, 1718, 1610, 1483, 1423, 1351, 1188, 1056, 1016, 958, 921, 887, 831, 721 cm^{-1}; MS (*m/z*): HRMS (ESI) Calcd. for $C_{41}H_{36}NaClN_3O_2$ ($[M+Na]^+$): 660.2394, found: 660.2367.

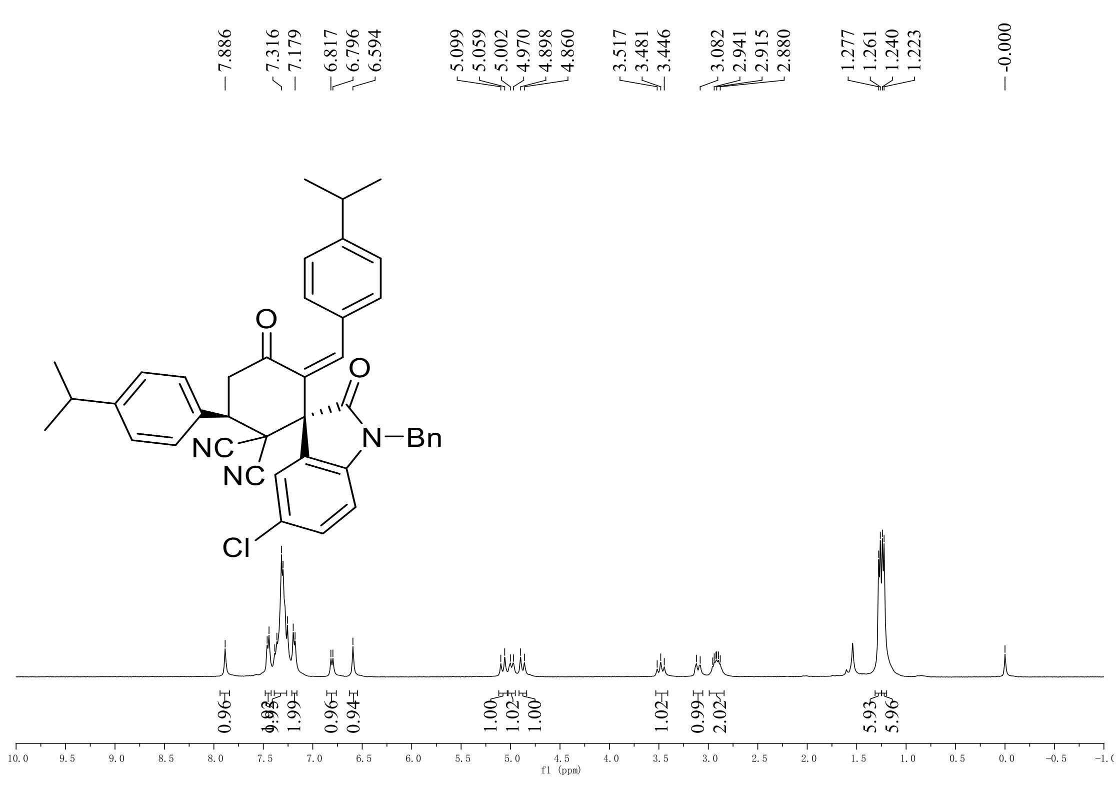

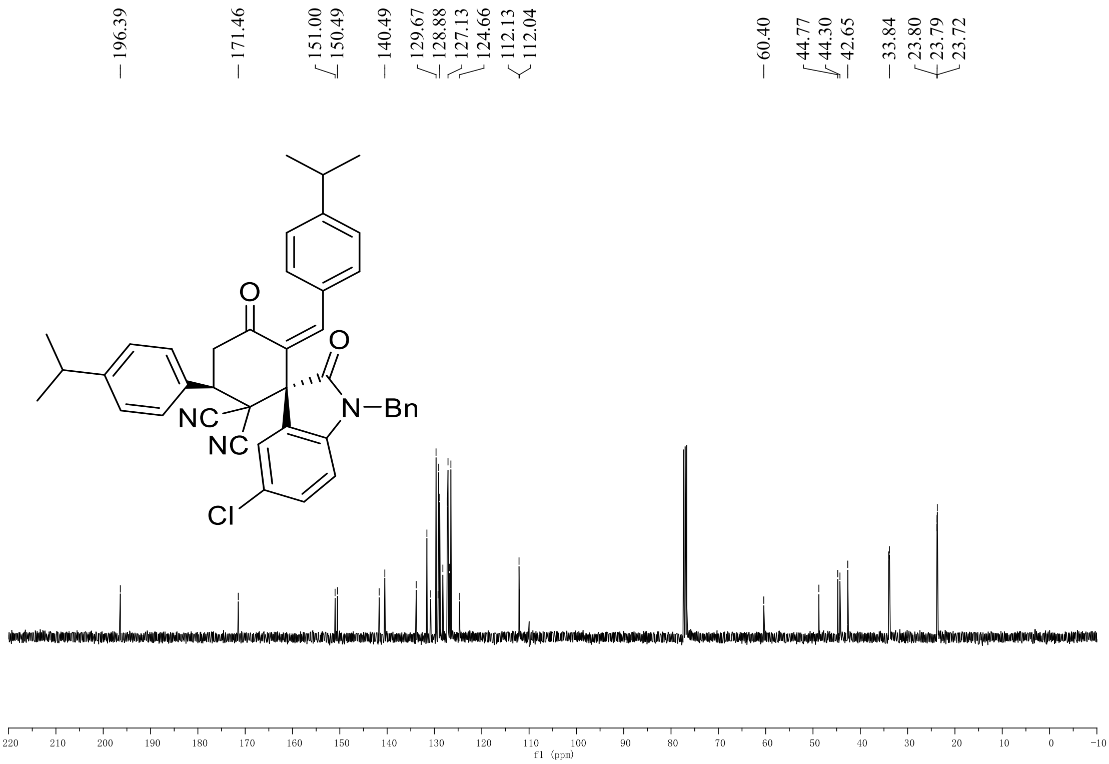

196.39
171.46
151.00
150.49
140.49
129.67
128.88
127.13
124.66
112.13
112.04
60.40
44.77
44.30
42.65
33.84
23.80
23.79
23.72
O
O
N−Bn
NC
NC
Cl
f1 (ppm)

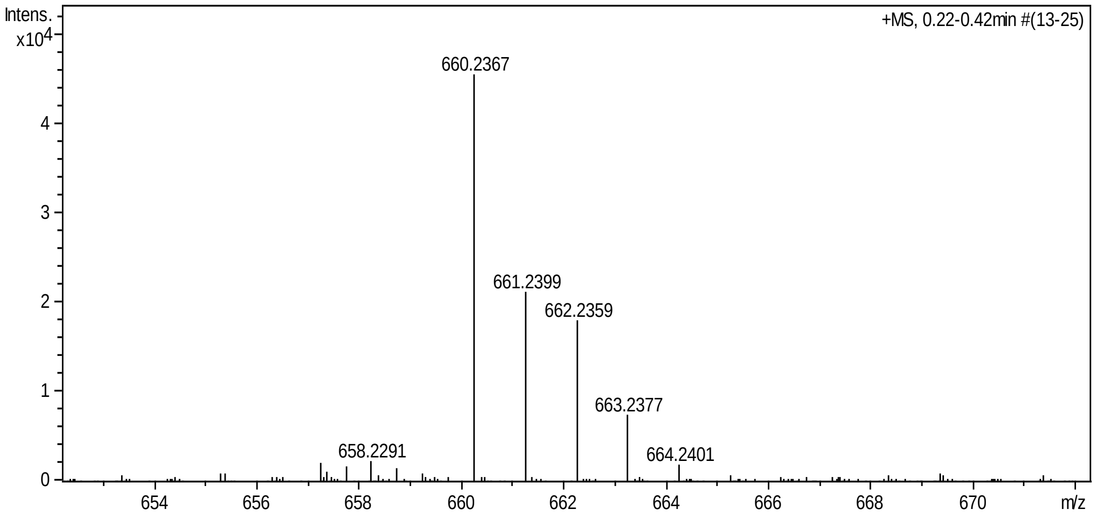

Intens.
x10^4
+MS, 0.22-0.42min #(13-25)
660.2367
661.2399
662.2359
663.2377
658.2291
664.2401
m/z

***rel*-(1*R*,3*R*)-1'-Butyl-5'-chloro-6-((*Z*)-4-methylbenzylidene)-2',5-dioxo-3-(*p*-tolyl)spiro[cyclohexane-1,3'-indoline]-2,2-dicarbonitrile (3o)**: white solid, 42%, m.p. 225-227 °C; ^{1}H NMR (400 MHz, $CDCl_3$) δ 7.88 (s, 1H, ArH), 7.51 (d, *J* = 7.2 Hz, 1H, ArH), 7.40 (d, *J* = 7.2 Hz, 2H, ArH), 7.24-7.23 (m, 4H, ArH), 7.12 (d, *J* = 6.8 Hz, 2H, ArH), 6.99 (d, *J* = 8.0 Hz, 1H, ArH), 6.60 (s, 1H, CH), 4.93-4.90 (m, 1H, CH_2), 3.91-3.84 (m, 1H, CH_2), 3.64-3.60 (m, 1H, CH_2), 3.47-3.40 (m, 1H, CH), 3.07-3.03 (m, 1H, CH_2), 2.37-2.33 (s, 3H, CH_3), 1.72-1.69 (m, 2H, CH_2), 1.43-1.42 (m, 2H, CH_2), 0.97 (d, *J* = 5.6 Hz, 1H, CH_3). ^{13}C NMR (100 MHz, $CDCl_3$) δ 196.3, 171.1, 142.1, 140.3, 140.0, 139.7, 131.6, 131.4, 130.5, 129.9, 129.4, 129.3, 129.2, 129.1, 128.8, 127.0, 124.7, 112.2, 111.9, 111.1, 60.1, 48.9, 44.3, 42.5, 40.8, 29.1, 21.4, 21.2, 20.1, 13.6. IR (KBr) υ: 3689, 2962, 2317, 1713, 1595, 1501, 1468, 1388, 1354, 1324, 1271, 1244, 1212, 1161, 1135, 1047, 882, 831 cm^{-1}; MS (*m*/*z*): HRMS (ESI) Calcd. for $C_{34}H_{30}ClNaN_3O_2$ ($[M+Na]^+$): 570.1924, found: 570.1912.

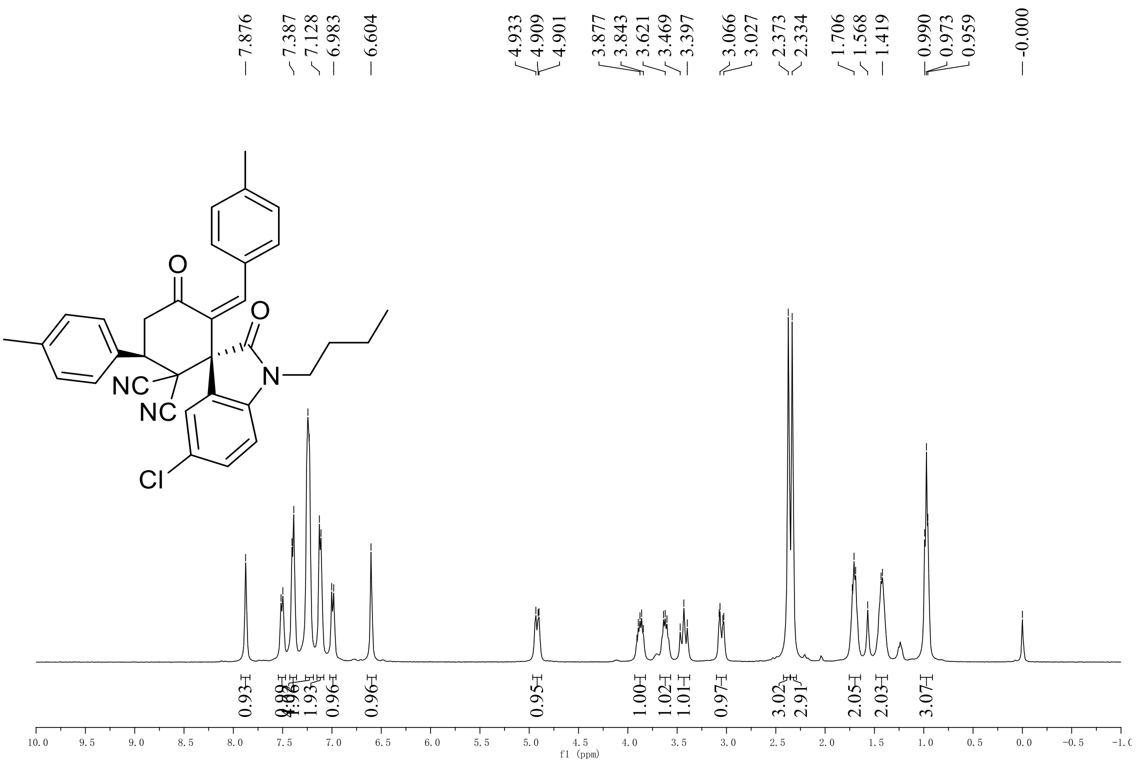

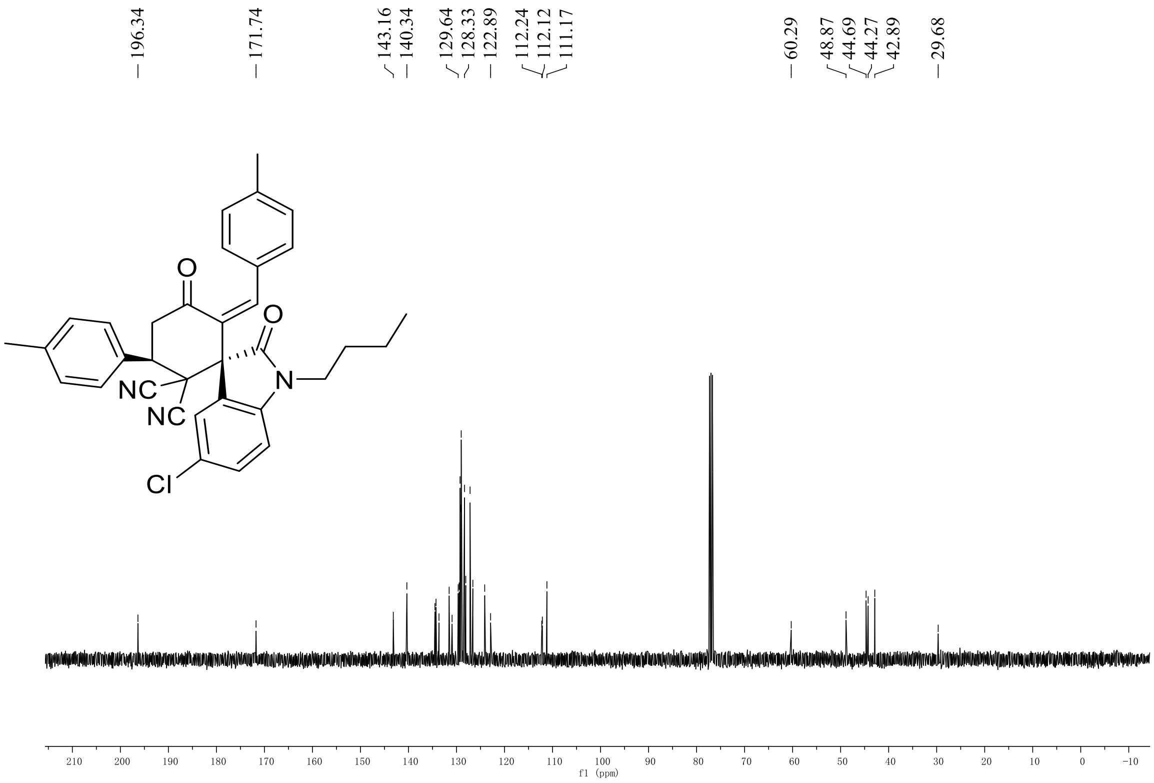
196.34
171.74
143.16
140.34
129.64
128.33
122.89
112.24
112.12
111.17
60.29
48.87
44.69
44.27
42.89
29.68
O
O
N
NC
NC
Cl
f1 (ppm)

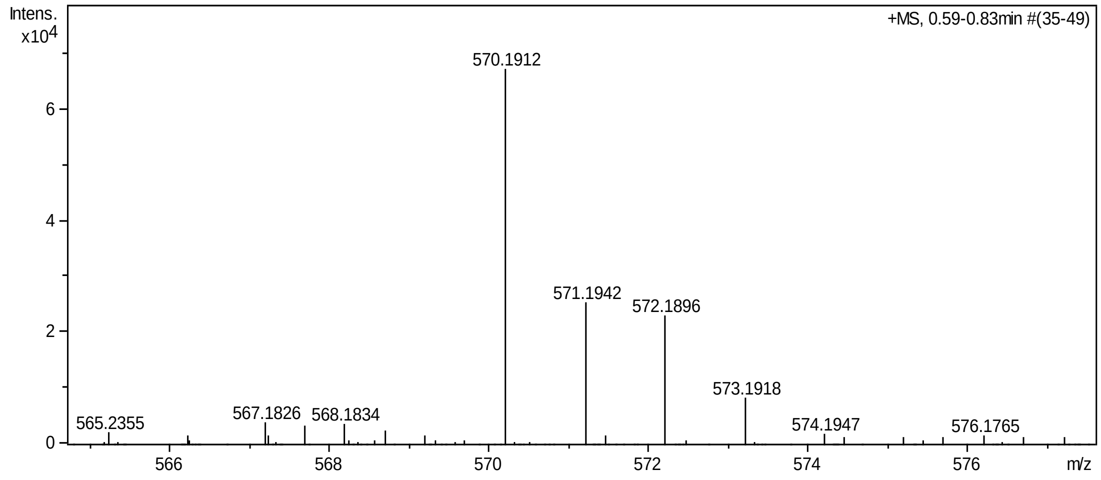
Intens.
x10^4
+MS, 0.59-0.83min #(35-49)
570.1912
571.1942
572.1896
573.1918
574.1947
576.1765
565.2355
567.1826
568.1834
m/z

***rel-(1R,3R)*-1'-Butyl-5'-chloro-6-((*E*)-4-methylbenzylidene)-2',5-dioxo-3-(p-tolyl)spiro[cyclohexane-1,3'-indoline]-2,2-dicarbonitrile (3o')**: lilac solid, 8%, m.p. 167-169 °C; ^{1}H NMR (400 MHz, $CDCl_3$) δ 8.12 (s, 1H, ArH), 7.39 (d, *J* = 8.4 Hz, 2H, ArH), 7.26-7.23 (m, 2H, ArH), 7.13-7.09 (m, 2H, ArH), 6.77 (d, *J* = 7.6 Hz, 2H, ArH), 6.56 (d, *J* = 8.4 Hz, 1H, CH), 6.48 (d, *J* = 8.0 Hz, 2H, ArH)), 4.96-4.91 (m, 1H, CH_2), 3.86-3.78 (m, 1H, CH_2), 3.48-3.43 (m, 1H, CH_2), 3.41-3.37 (m, 1H, CH), 3.21-3.15 (m, 1H, CH_2), 2.36 (s, 3H, CH_3), 2.21 (s, 3H, CH_3), 1.71-1.63 (m, 2H, CH_2), 1.52-1.42 (m, 2H, CH_2), 1.00 (t, *J* = 7.2 Hz, 3H, CH_3). ^{13}C NMR (100 MHz, $CDCl_3$) δ 194.0, 172.2, 147.5, 141.5, 139.5, 138.4, 132.0, 131.3, 130.1, 130.0, 129.9, 128.8, 128.5, 128.2, 127.0, 126.6, 126.3, 112.1, 111.5, 109.9, 56.1, 49.0, 40.9, 39.3, 29.0, 21.2, 21.1, 20.4, 13.7; IR (KBr) υ: 3628, 3413, 3041, 2910, 2832, 1958, 1845, 1813, 1710, 1543, 1483, 1342, 1231, 1123, 1043, 1010, 945, 821, 753, 721 cm^{-1}; MS (*m*/*z*): HRMS (ESI) Calcd. for $C_{34}H_{30}ClNaN_3O_2$ ([M+Na]$^+$): 570.1924, found: 570.1910.

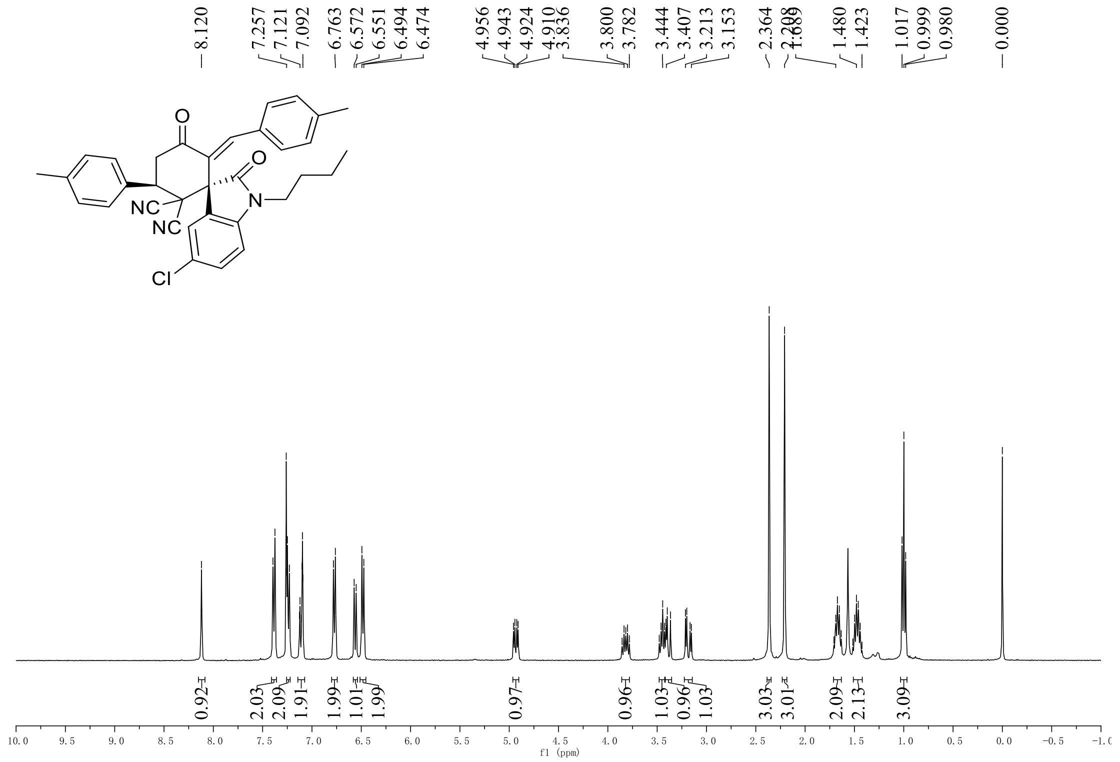

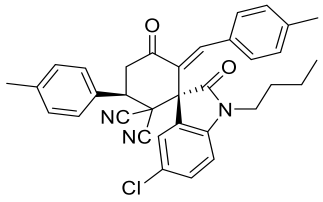
O
O
N
NC
NC
Cl

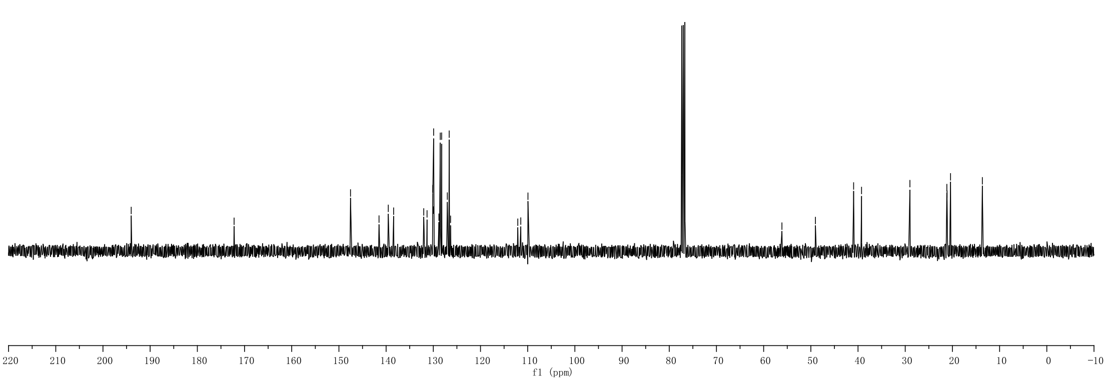
194.01
172.21
147.54
138.40
129.93
128.23
126.34
112.10
111.46
109.94
56.12
49.02
40.93
39.25
29.01
20.40
13.66
220
210
200
190
180
170
160
150
140
130
120
110
100
90
80
70
60
50
40
30
20
10
0
−10
f1 (ppm)

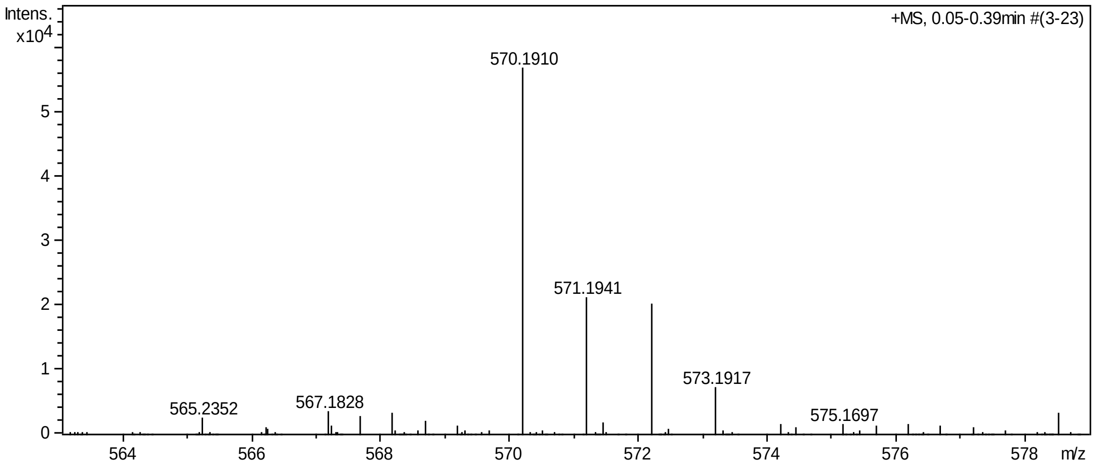
Intens.
x10^4
+MS, 0.05-0.39min #(3-23)
570.1910
571.1941
573.1917
565.2352
567.1828
575.1697
0
1
2
3
4
5
564
566
568
570
572
574
576
578
m/z

***rel*-(1*R*,3*R*)-1'-Benzyl-5'-fluoro-6-((*Z*)-4-methylbenzylidene)-2',5-dioxo-3-(*p*-tolyl)spiro[cyclohexane-1,3'-indoline]-2,2-dicarbonitrile (3p)**: white solid, 53%, m.p. 223-225 °C; ^{1}H NMR (400 MHz, $CDCl_3$) δ 7.70-7.67 (m, 1H, ArH), 7.43 (d, *J* = 8.0 Hz, 2H, ArH), 7.36-7.30 (m, 5H, ArH), 7.27 (s, 2H, ArH), 7.24 (s, 2H, ArH), 7.14-7.09 (m, 3H, ArH), 6.83-6.79 (m, 1H, ArH), 6.60 (s, 1H, CH), 5.09 (d, *J* = 15.6 Hz, 1H, CH_2), 5.03-4.98 (m, 1H, CH_2), 4.86 (d, *J* = 16.0 Hz, 1H, CH_2), 3.46 (t, *J* = 14.8 Hz, 1H, CH), 3.12-3.07 (m, 1H, CH_2), 2.38 (s, 3H, CH_3), 2.34 (s, 3H, CH_3). ^{13}C NMR (100 MHz, $CDCl_3$) δ 196.3, 171.6, 160.6, 158.1, 140.4, 140.1, 139.7, 139.2, 134.0, 131.4, 130.5, 129.9, 129.5, 129.4, 129.1, 129.1, 128.8, 128.2, 127.1, 124.5, 124.4, 118.3, 118.1, 114.9, 114.6, 112.1, 112.0, 112.0, 60.5, 48.8, 44.8, 44.3, 42.6, 21.4, 21.2. IR (KBr) υ: 3725, 3407, 3031, 2917, 2319, 1881, 1715, 1614, 1494, 1452, 1421, 1352, 1270, 1185, 1145, 1021, 965, 928, 875, 819, 731 cm^{-1}; MS (*m*/*z*): HRMS (ESI) Calcd. for $C_{37}H_{28}NaFN_3O_2$ ([M+Na]$^+$): 588.2063, found: 588.2053.

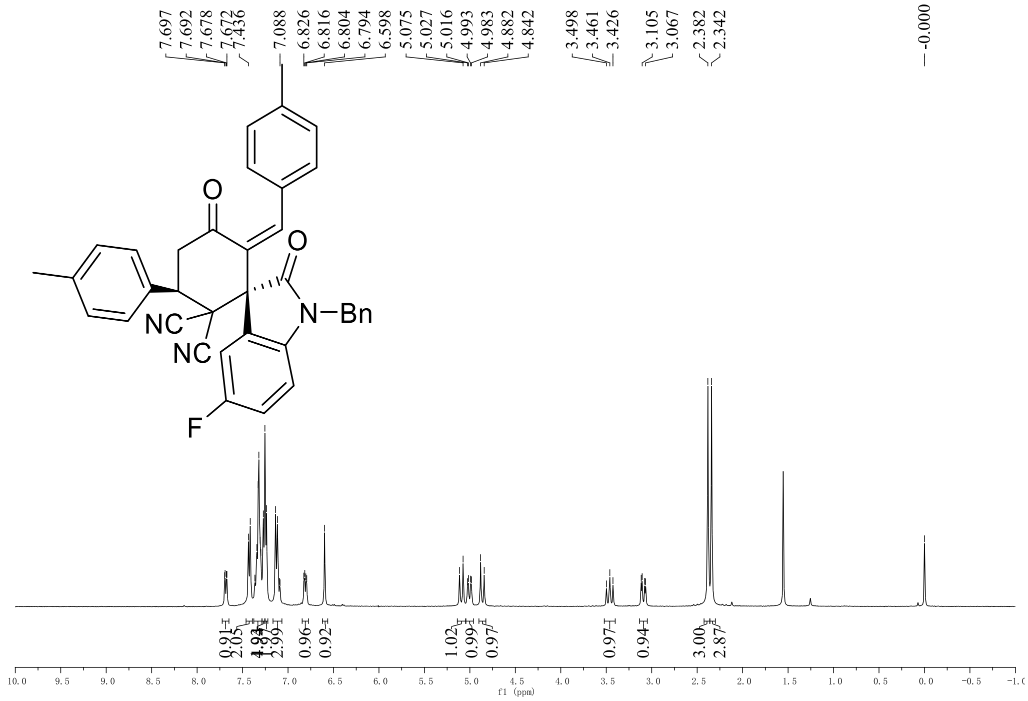

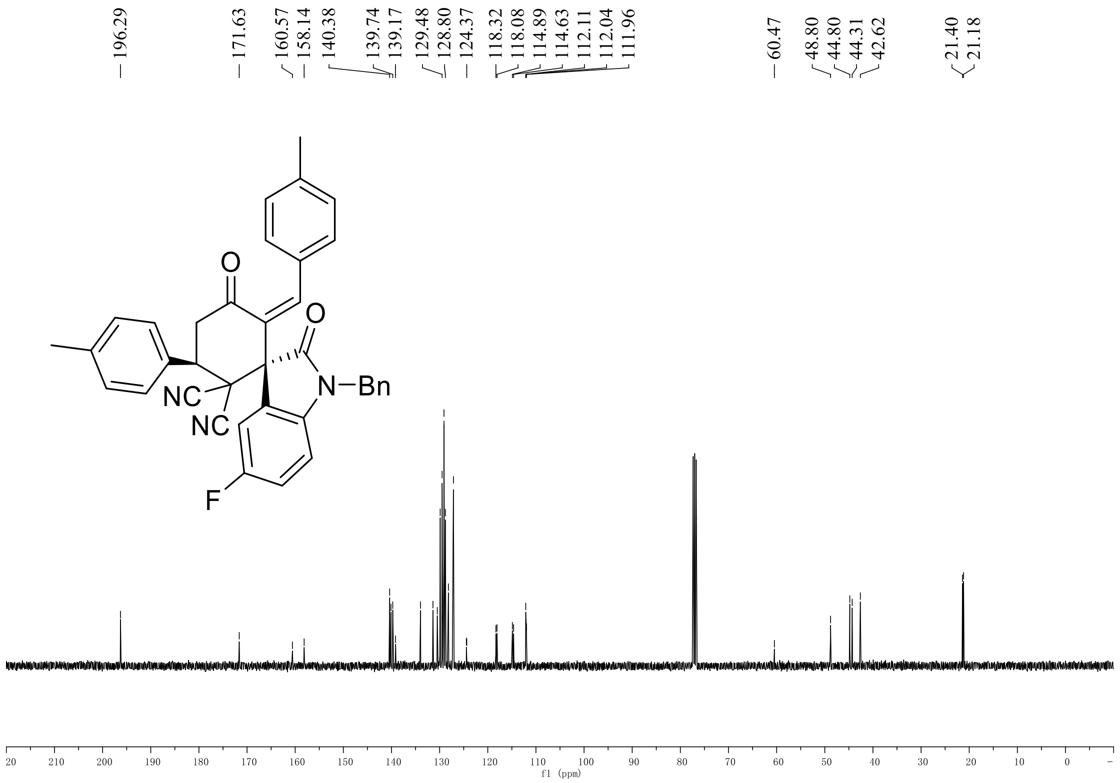
196.29
171.63
160.57
158.14
140.38
139.74
139.17
129.48
128.80
124.37
118.32
118.08
114.89
114.63
112.11
112.04
111.96
60.47
48.80
44.80
44.31
42.62
21.40
21.18
O
O
N−Bn
NC
NC
F
f1 (ppm)

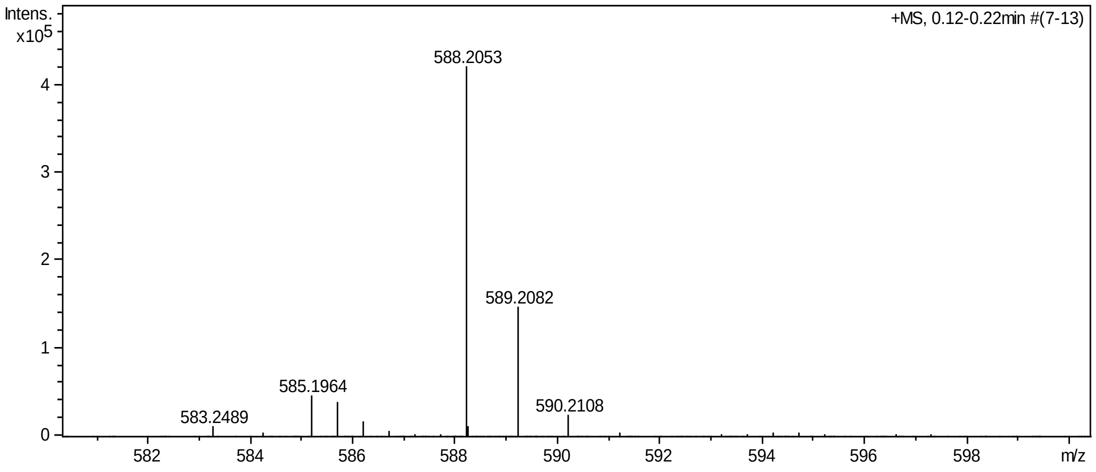
Intens.
x10^5
+MS, 0.12-0.22min #(7-13)
588.2053
589.2082
585.1964
583.2489
590.2108
m/z

***rel*-(1*R*,3*R*)-1'-Benzyl-5'-fluoro-6-((*Z*)-4-isopropylbenzylidene)-3-(4-isopropylphenyl)-2',5-dioxospiro[cyclohexane-1,3'-indoline]-2,2-dicarbonitrile (3q)**: white solid, 58%, m.p. 195-197 °C; ^{1}H NMR (400 MHz, $CDCl_3$) δ 7.70-7.67 (m, 1H, ArH), 7.45 (d, *J* = 8.4 Hz, 2H, ArH), 7.37-7.29 (m, 9H, ArH), 7.18 (d, *J* = 8.4 Hz, 2H, ArH), 7.14-7.09 (m, 1H, ArH), 6.83-6.80 (m, 1H, ArH), 6.60 (s, 1H, CH), 5.09 (d, *J* = 16.0 Hz, 1H, CH_2), 5.03-4.99 (m, 1H, CH_2), 4.88 (d, *J* = 16.0 Hz, 1H, CH_2), 3.47 (t, *J* = 15.2 Hz, 1H, CH), 3.13-3.08 (m, 1H, CH_2), 2.97-2.86 (m, 2H, CH), 1.27 (d, *J* = 6.8 Hz, 6H, $(CH_3)_2$), 1.23 (d, *J* = 6.8 Hz, 6H, $(CH_3)_2$). ^{13}C NMR (100 MHz, $CDCl_3$) δ 196.4, 171.6, 158.1, 151.0, 150.5, 140.3, 139.2, 139.1, 134.0, 131.6, 130.8, 129.7, 129.3, 129.1, 128.9, 128.2, 127.3, 127.1, 126.5, 124.5, 118.3, 118.1, 114.9, 114.6, 112.1, 112.0, 111.9, 60.5, 48.8, 44.8, 44.3, 42.6, 34.0, 33.8, 26.9, 23.8, 23.8, 23.7, 23.7. IR (KBr) υ: 3059, 2961, 1715, 1612, 1492, 1454, 1420, 1353, 1270, 1185, 1058, 1018, 966, 926, 831, 727 cm^{-1}; MS (*m*/*z*): HRMS (ESI) Calcd. for $C_{41}H_{36}NaFN_3O_2$ ($[M+Na]^+$): 644.2689, found: 644.2666.

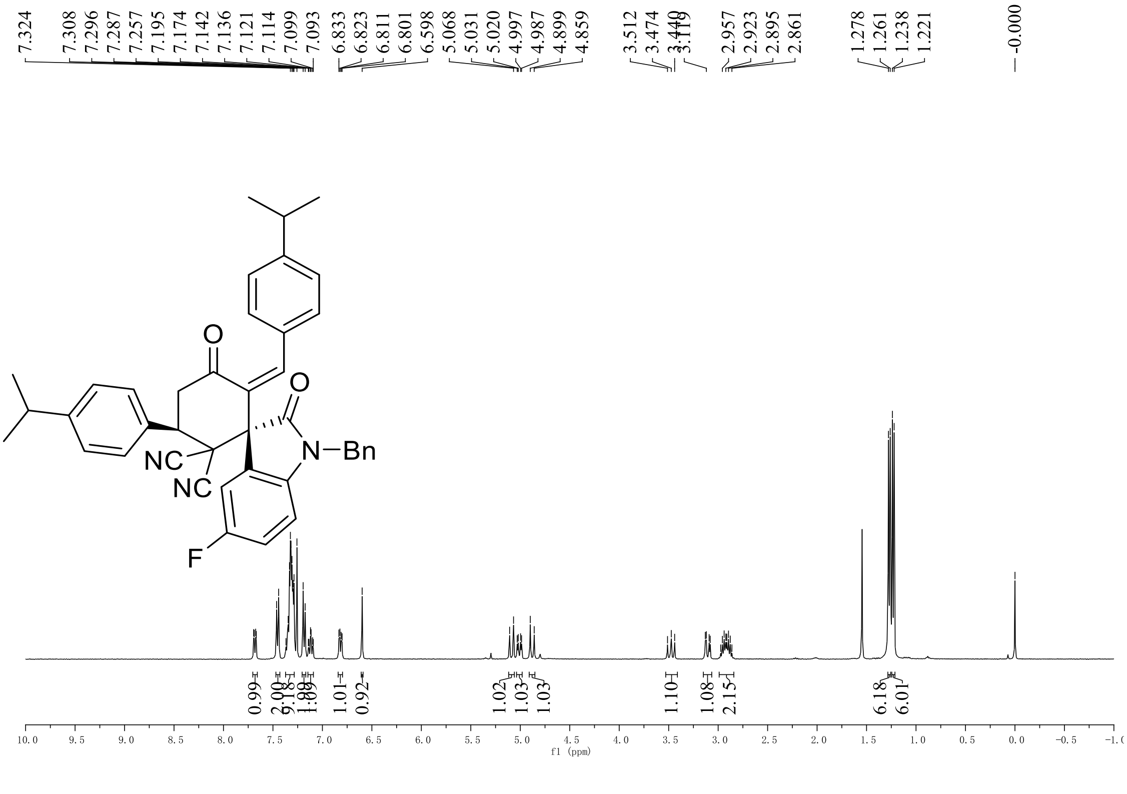

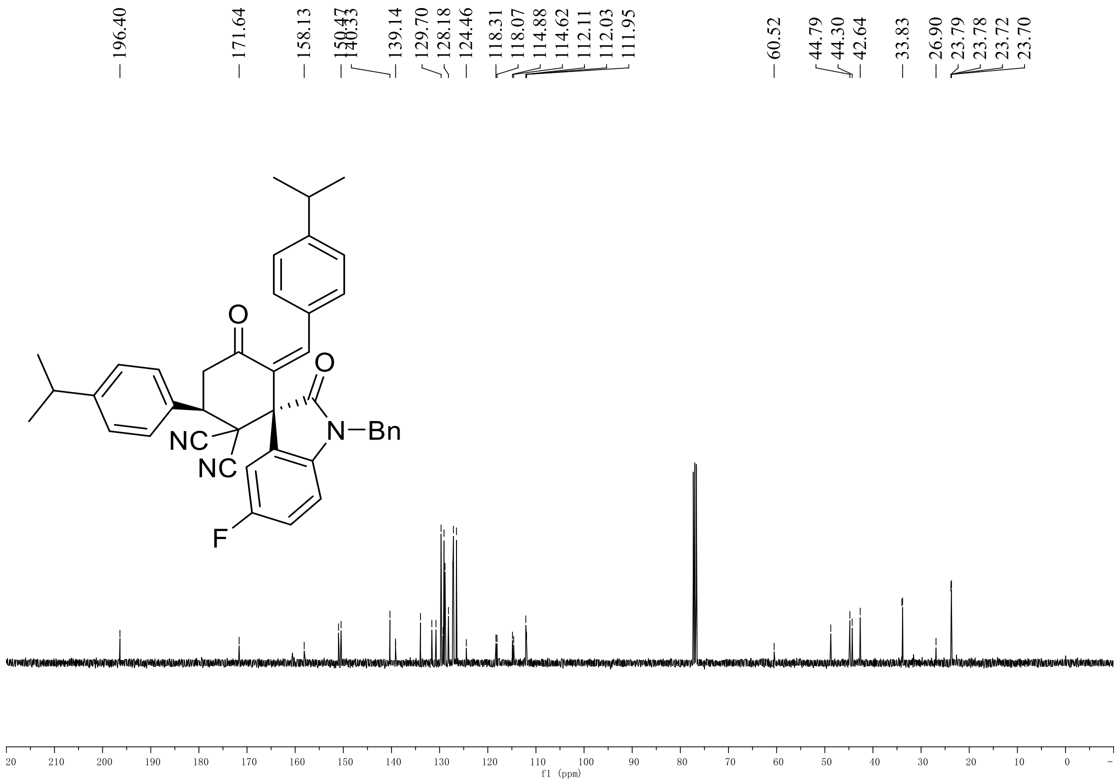
196.40
171.64
158.13
150.47
139.14
129.70
128.18
124.46
118.31
118.07
114.88
114.62
112.11
112.03
111.95
60.52
44.79
44.30
42.64
33.83
26.90
23.79
23.78
23.72
23.70
O
O
N−Bn
NC
NC
F
f1 (ppm)

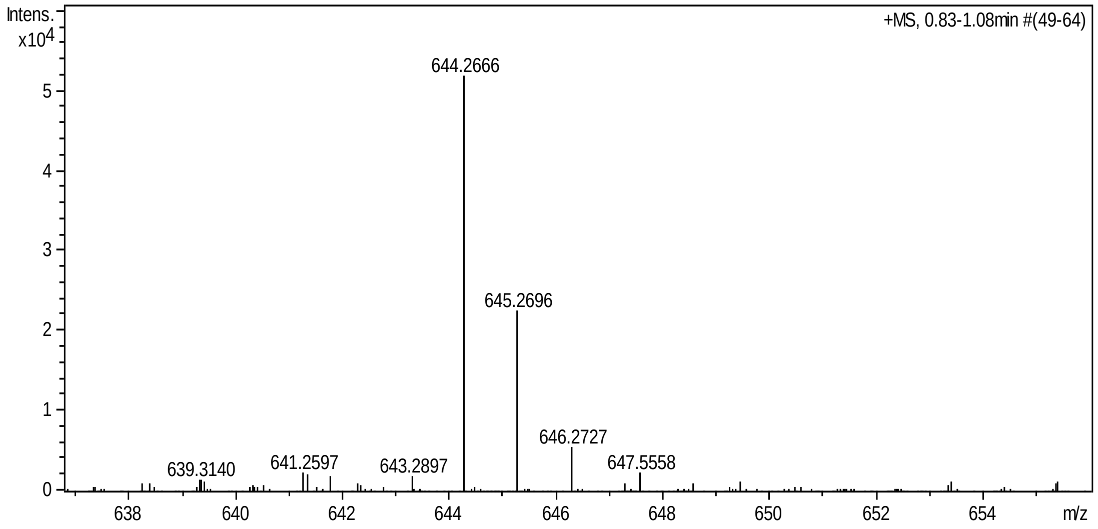
Intens.
x10^4
+MS, 0.83-1.08min #(49-64)
644.2666
645.2696
646.2727
647.5558
639.3140
641.2597
643.2897
m/z

***rel*-(1*R*,3*R*)-6-((*Z*)-4-Methylbenzylidene)-2',5-dioxo-3-(*p*-tolyl)spiro[cyclohexane-1,3'-indoline]-2,2-dicarbonitrile (3r)**: pale pink solid, 48%, m.p. 252-254 °C; ^{1}H NMR (400 MHz, $CDCl_3$) δ 8.12 (s, 1H, NH), 7.87 (d, *J* = 7.6 Hz, 1H, ArH), 7.49-7.41 (m, 3H, ArH), 7.29-7.27 (m, 2H, ArH), 7.26-7.22 (m, 3H, ArH), 6.67 (s, 1H, CH), 4.92 (m, 1H, CH_2), 3.50-3.43 (m, 1H, CH), 3.09-3.04 (m, 1H, CH_2). ^{13}C NMR (100 MHz, $CDCl_3$) δ 197.6, 173.2, 141.1, 141.0, 139.9, 139.7, 131.5, 131.4, 130.7, 129.8, 129.2, 129.3, 129.1, 128.8, 126.7, 124.0, 123.4, 112.2, 112.0, 111.8, 60.7, 49.1, 44.3, 42.4, 21.4, 21.2. IR (KBr) υ: 3686, 3067, 1723, 1567, 1514, 1474, 1420, 1381, 1323, 1191, 1045, 821, 759 cm^{-1}; MS (*m*/*z*): HRMS (ESI) Calcd. for $C_{30}H_{23}NaN_3O_2$ ([M+Na]$^+$): 480.1688, found: 480.1675.

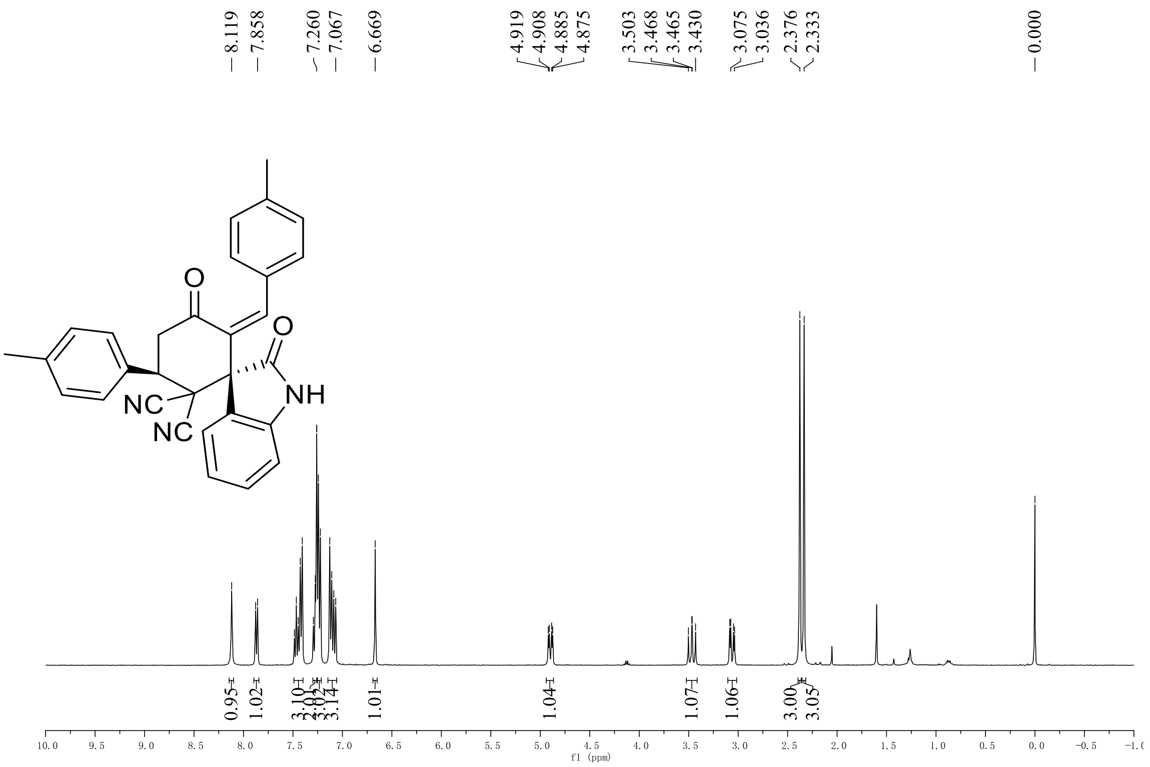

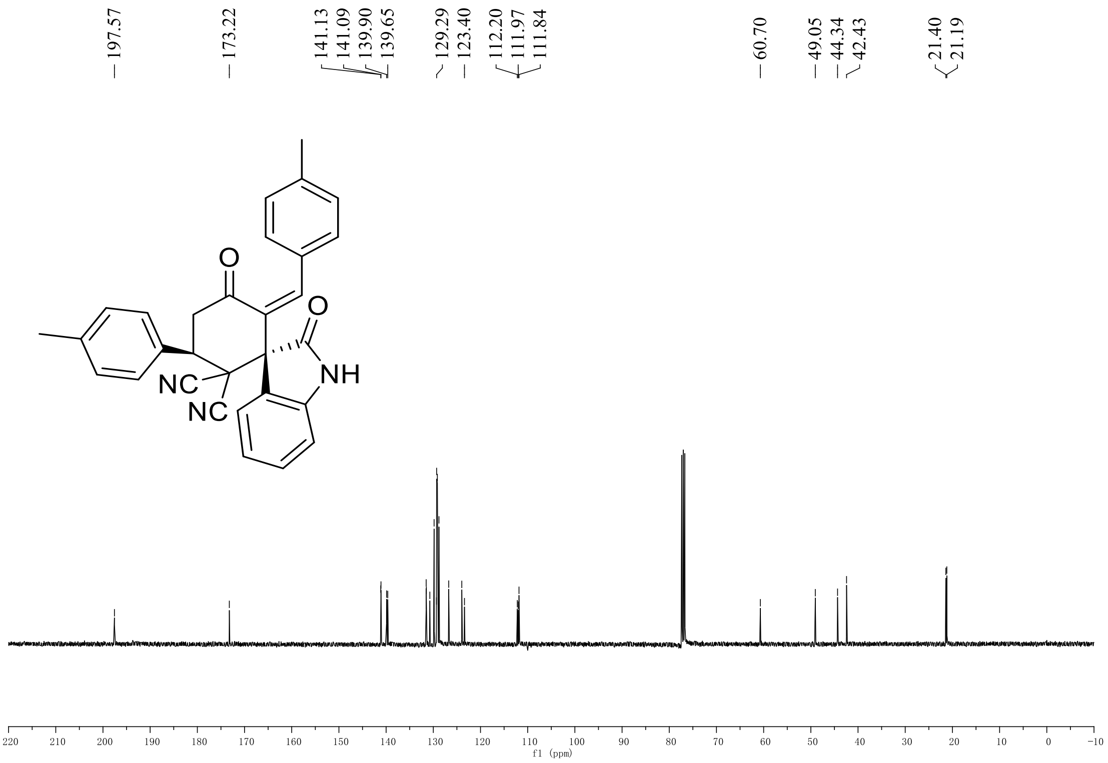

197.57
173.22
141.13
141.09
139.90
139.65
129.29
123.40
112.20
111.97
111.84
60.70
49.05
44.34
42.43
21.40
21.19
O
O
NH
NC
NC
220 210 200 190 180 170 160 150 140 130 120 110 100 90 80 70 60 50 40 30 20 10 0 −10
f1 (ppm)

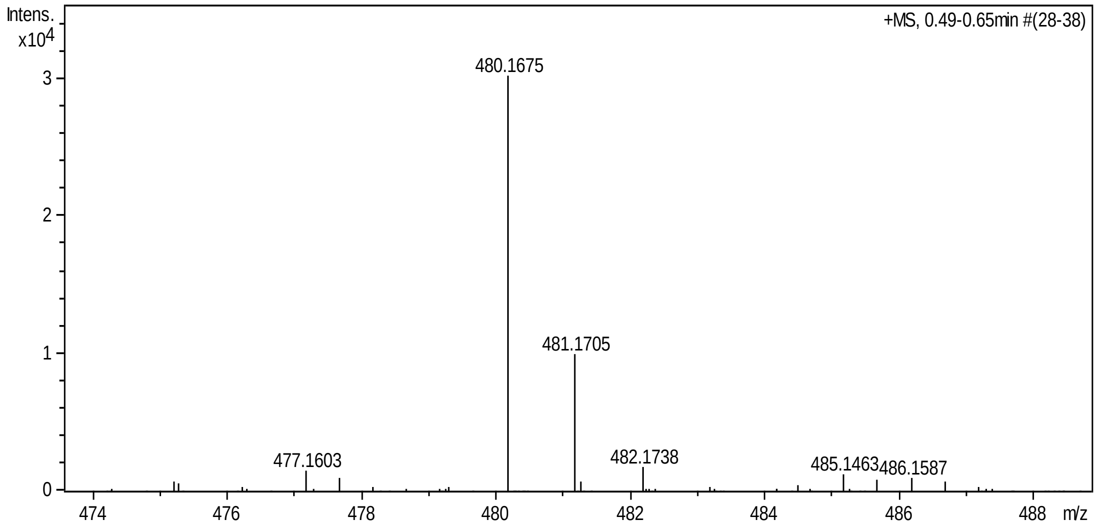

Intens.
x10^4
+MS, 0.49-0.65min #(28-38)
480.1675
481.1705
477.1603
482.1738
485.1463
486.1587
0
1
2
3
474
476
478
480
482
484
486
488
m/z

***rel*-(1*R*,3*R*)-1'-Benzyl-6-((*Z*)-4-methylbenzylidene)-2',5-dioxo-3-(*p*-tolyl)spiro[cyclohexane-1,3'-indoline]-2,2-dicarbonitrile (3s)**: pale yellow solid, 58%, m.p. 212-214 °C; ^{1}H NMR (600 MHz, $CDCl_3$) δ 7.91 (d, *J* = 6.6 Hz, 1H, ArH), 7.44-7.39 (m, 3H, ArH), 7.34 (s, 4H, ArH), 7.30-7.27 (m, 2H, ArH), 7.25-7.23 (m, 4H, ArH), 7.11 (d, *J* = 7.2 Hz, 2H, ArH), 6.88 (d, *J* = 7.2 Hz, 1H, ArH), 6.62 (s, 1H, CH), 5.10 (d, *J* = 15.6 Hz, 1H, CH_2), 5.03-5.00 (m, 1H, CH_2), 4.88 (d, *J* = 16.2 Hz, 1H, CH_2), 3.47 (t, *J* = 13.2 Hz, 1H, CH), 3.11-3.07 (m, 1H, CH_2), 2.38 (s, 3H, CH_3), 2.34 (s, 3H, CH_3).^{13}C NMR (100 MHz, $CDCl_3$) δ 196.7, 171.9, 143.2, 140.3, 139.9, 139.6, 134.3, 131.6, 131.4, 130.7, 129.9, 129.8, 129.5, 129.1, 129.0, 128.8, 128.0, 127.2, 126.6, 124.1, 123.1, 112.2, 112.3, 111.1, 60.3, 49.0, 44.7, 44.4, 42.6, 21.4, 21.2. IR (KBr) υ: 3729, 3407, 3029, 2965, 2922, 2859, 2319, 1980, 1903, 1805, 1710, 1607, 1482, 1362, 1298, 1183, 1153, 1120, 1045, 953, 894, 812, 763, 733 cm^{-1}; MS (*m*/*z*): HRMS (ESI) Calcd. for $C_{37}H_{29}NaN_3O_2$ ([M+Na]$^{+}$): 570.2157, found: 570.2152.

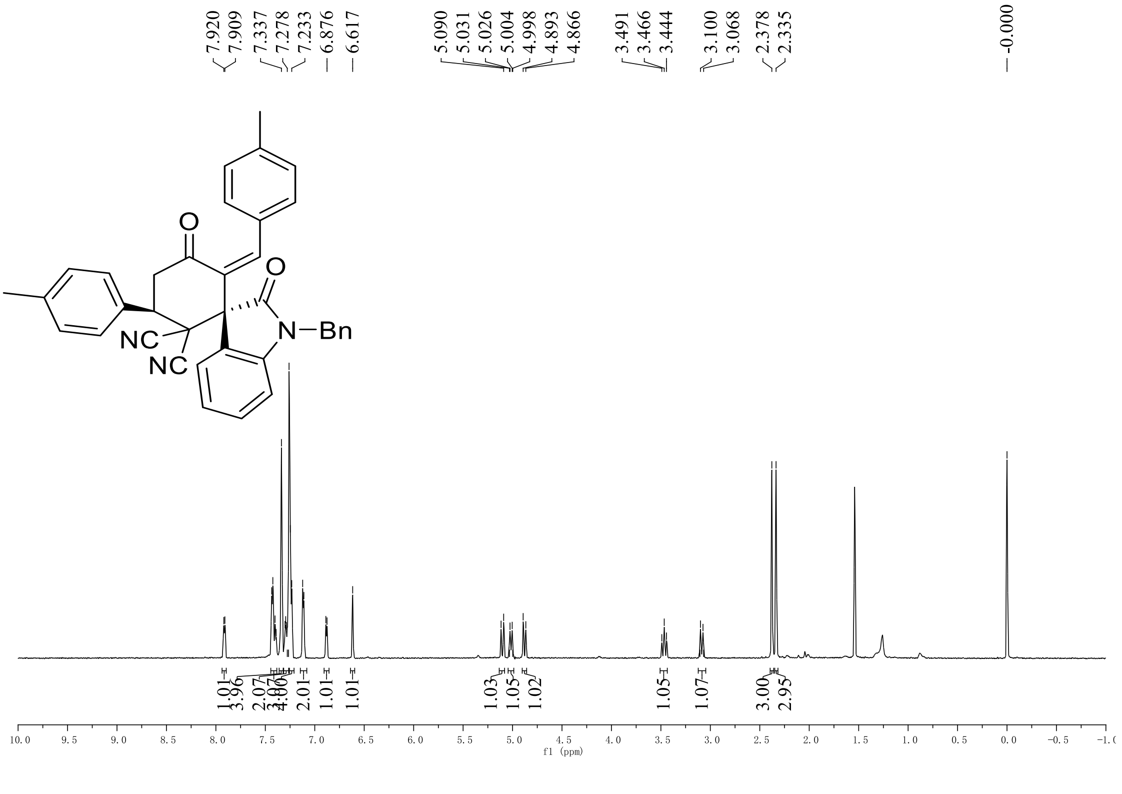

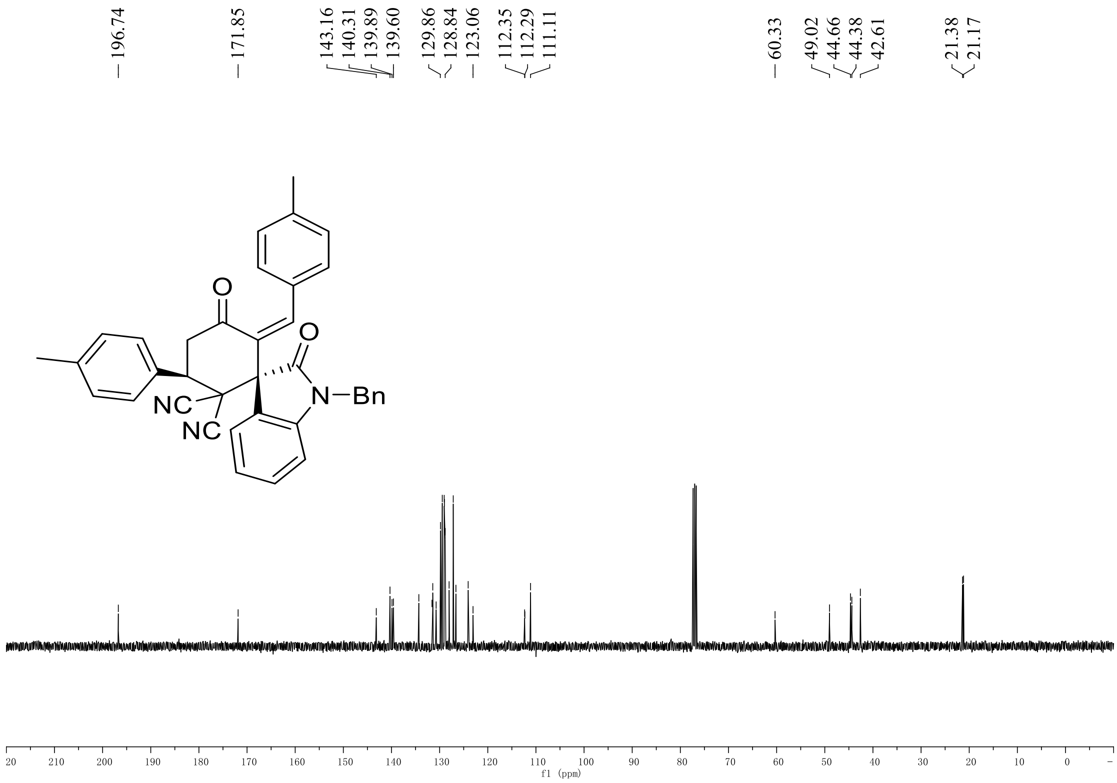

196.74
171.85
143.16
140.31
139.89
139.60
129.86
128.84
123.06
112.35
112.29
111.11
60.33
49.02
44.66
44.38
42.61
21.38
21.17
O
O
N−Bn
NC
NC
f1 (ppm)

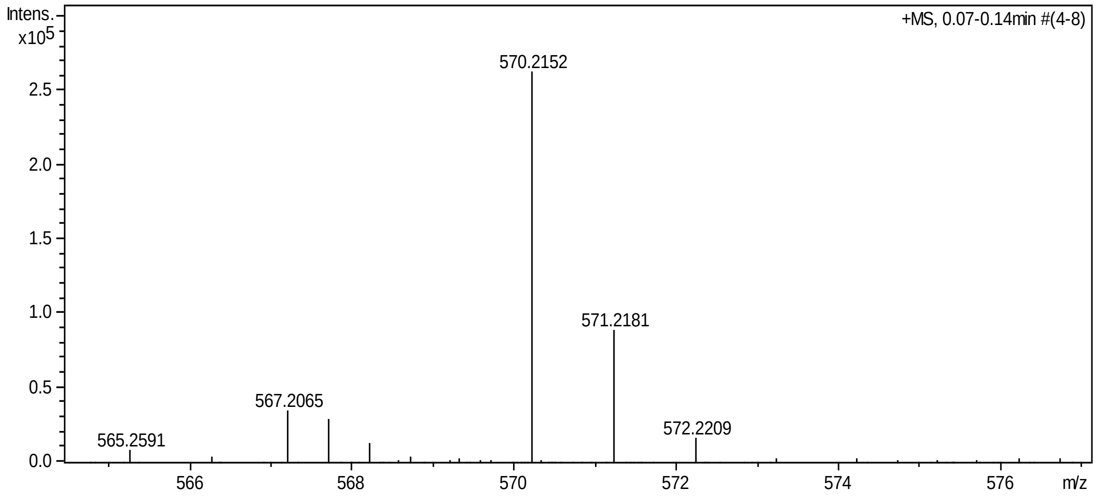

Intens.
x10^5
+MS, 0.07-0.14min #(4-8)
570.2152
571.2181
567.2065
572.2209
565.2591
m/z

***rel*-(1*R*,3*R*)-1'-Benzyl-6-((*Z*)-4-isopropylbenzylidene)-3-(4-isopropylphenyl)-2',5-dioxospiro[cyclohexane-1,3'-indoline]-2,2-dicarbonitrile (3t)**: white solid, 57%, m.p. 168-170 °C; ^{1}H NMR (400 MHz, $CDCl_3$) δ 7.91 (d, *J* = 7.6 Hz, 1H, ArH), 7.46 (d, *J* = 8.0 Hz, 2H, ArH), 7.40 (d, *J* = 8.0 Hz, 1H, ArH), 7.35-7.33 (m, 4H, ArH), 7.35-7.33 (m, 4H, ArH), 7.31-7.28 (m, 5H, ArH), 7.24 (s, 1H, ArH), 7.17 (d, *J* = 8.0 Hz, 2H, ArH), 6.89 (d, *J* = 8.0 Hz, 1H, ArH), 6.62 (s, 1H, CH), 5.09 (d, *J* = 15.6 Hz, 1H, CH_2), 5.04-5.00 (m, 1H, CH_2), 4.89 (d, *J* = 16.0 Hz, 1H, CH_2), 3.48 (t, *J* = 14.0 Hz, 1H, CH), 3.12-3.07 (m, 1H, CH_2), 2.97-2.85 (m, 2H, CH), 1.26 (d, *J* = 6.8 Hz, 6H, $(CH_3)_2$), 1.22 (d, *J* = 6.8 Hz, 6H, $(CH_3)_2$). ^{13}C NMR (100 MHz, $CDCl_3$) δ 196.8, 171.9, 150.8, 150.3, 143.2, 140.3, 134.3, 131.8, 131.4, 131.0, 129.8, 129.7, 129.0, 128.9, 128.0, 127.2, 127.1, 126.6, 126.5, 124.0, 123.1, 112.2, 111.1, 60.4, 49.0, 44.7, 44.4, 42.7, 34.0, 33.8, 23.8, 23.8, 23.7, 23.6. IR (KBr) υ: 3685, 3057, 2962, 1711, 1609, 1479, 1420, 1364, 1191, 1056, 1014, 957, 845, 754 cm^{-1}; MS (*m*/*z*): HRMS (ESI) Calcd. for $C_{41}H_{37}NaN_3O_2$ ($[M+Na]^+$): 626.2783, found: 626.2762.

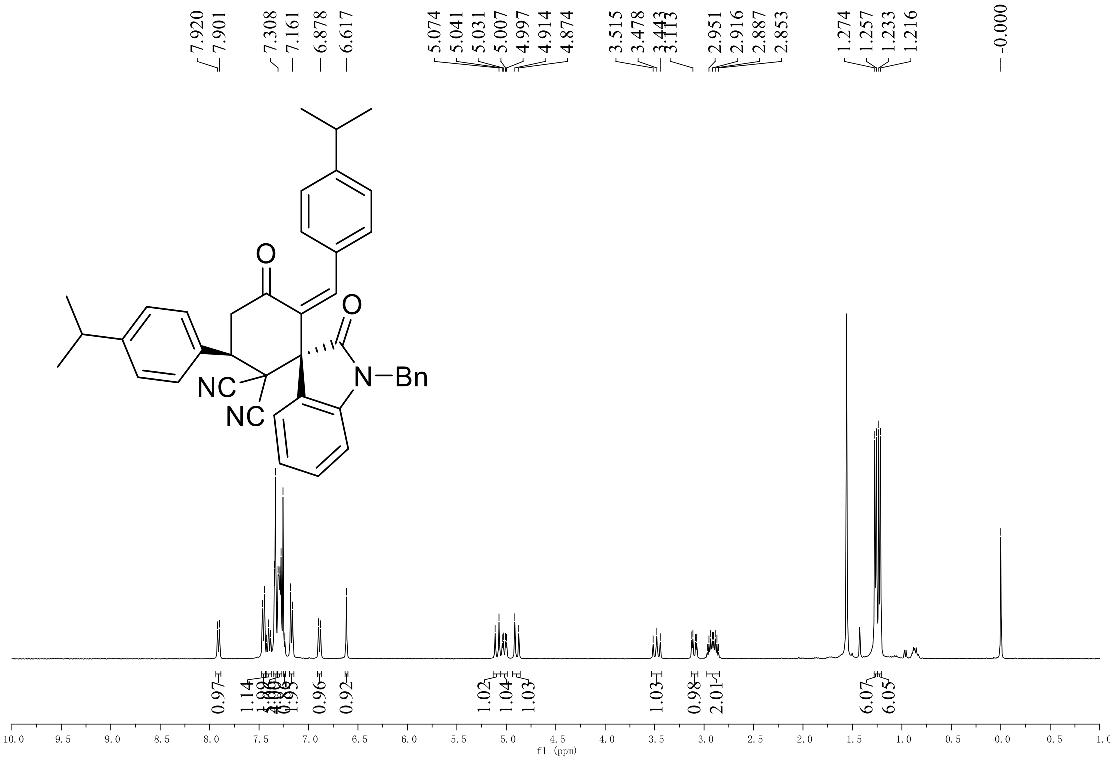

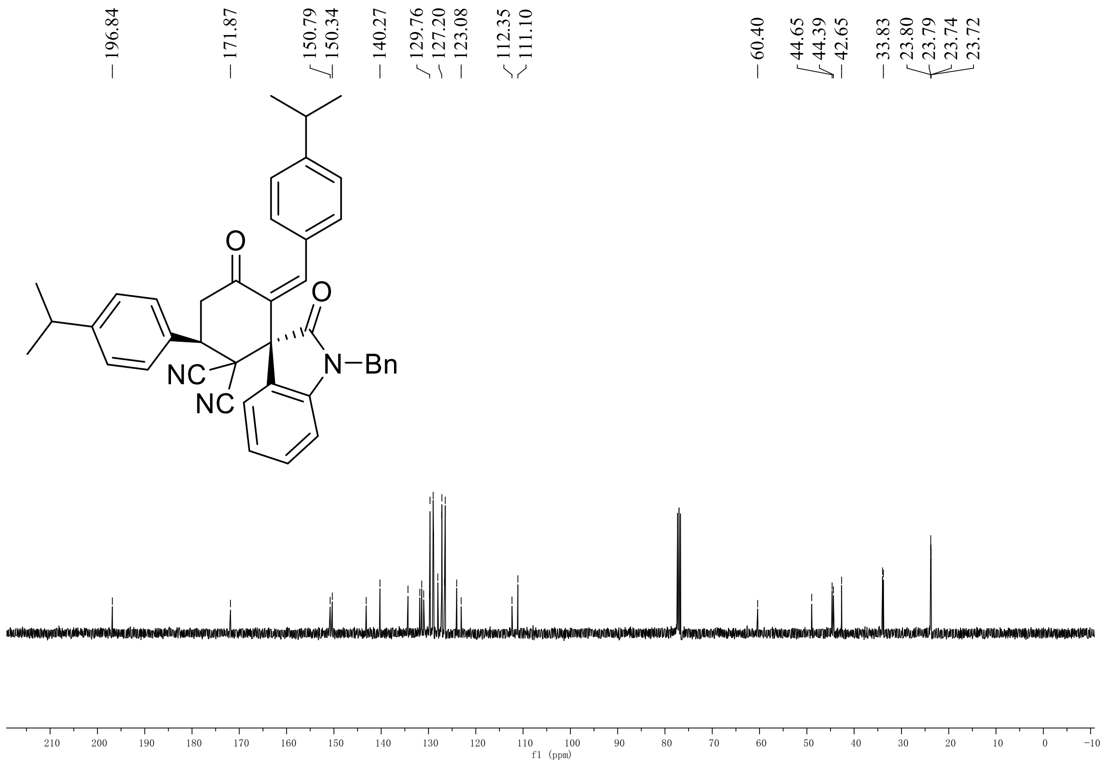

196.84
171.87
150.79
150.34
140.27
129.76
127.20
123.08
112.35
111.10
60.40
44.65
44.39
42.65
33.83
23.80
23.79
23.74
23.72
O
O
NC
NC
N−Bn
210 200 190 180 170 160 150 140 130 120 110 100 90 80 70 60 50 40 30 20 10 0 −10
f1 (ppm)

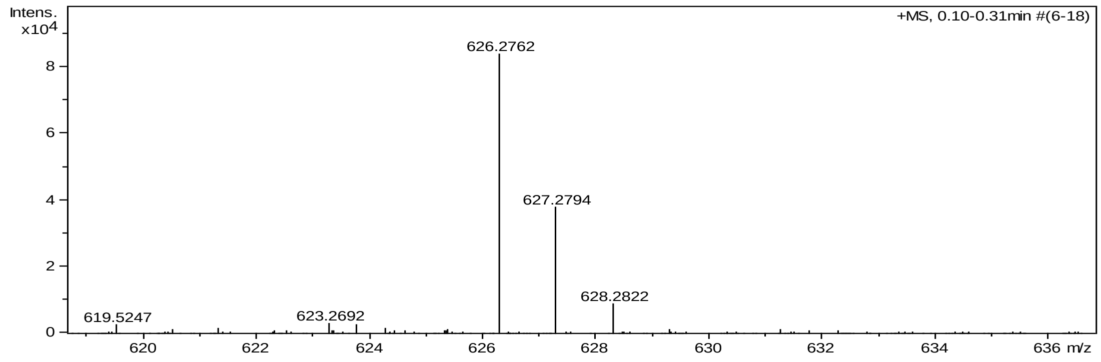

Intens.
x10^4
+MS, 0.10-0.31min #(6-18)
626.2762
627.2794
628.2822
619.5247
623.2692
0
2
4
6
8
620 622 624 626 628 630 632 634 636 m/z

***rel*-(1*R*,3*R*)-1'-Benzyl-6-((*Z*)-benzylidene)-2',5-dioxo-3-phenylspiro[cyclohexane-1,3'-indoline]-2,2-dicarbonitrile (3u)**: pale yellow solid, 42%, m.p. 211-213 °C; ^{1}H NMR (400 MHz, $CDCl_3$) δ 7.93 (d, *J* = 7.6 Hz, 1H, ArH), 7.56-7.54 (m, 2H, ArH), 7.46-7.39 (m, 4H, ArH), 7.35-7.27 (m, 10H, ArH), 7.25 (d, *J* = 7.2 Hz, 1H, ArH), 6.90 (d, *J* = 8.0 Hz, 1H, ArH), 6.68 (s, 1H, CH), 5.11 (d, *J* = 16.0 Hz, 1H, CH_2), 5.08-5.04 (m, 1H, CH_2), 4.89 (d, *J* = 16.0 Hz, 1H, CH_2), 3.50 (t, *J* = 14.0 Hz, 1H, CH), 3.14-3.09 (m, 1H, CH_2). ^{13}C NMR (100 MHz, $CDCl_3$) δ 196.3, 171.7, 143.2, 140.3, 134.5, 134.3, 133.7, 131.5, 130.9, 129.6, 129.4, 129.3, 129.2, 129.0, 128.3, 128.1, 127.2, 126.6, 124.1, 122.9, 112.2, 112.1, 111.2, 60.3, 48.9, 44.7, 44.3, 42.9, 29.7. IR (KBr) υ: 3728, 3413, 3040, 2919, 2852, 2317, 1958, 1888, 1813, 1711, 1607, 1483, 1457, 1356, 1231, 1187, 1069, 1009, 951, 835, 784, 746 cm^{-1}; MS (*m*/*z*): HRMS (ESI) Calcd. for $C_{35}H_{25}NaN_3O_2$ ([M+Na]$^+$): 542.1844, found: 542.1844.

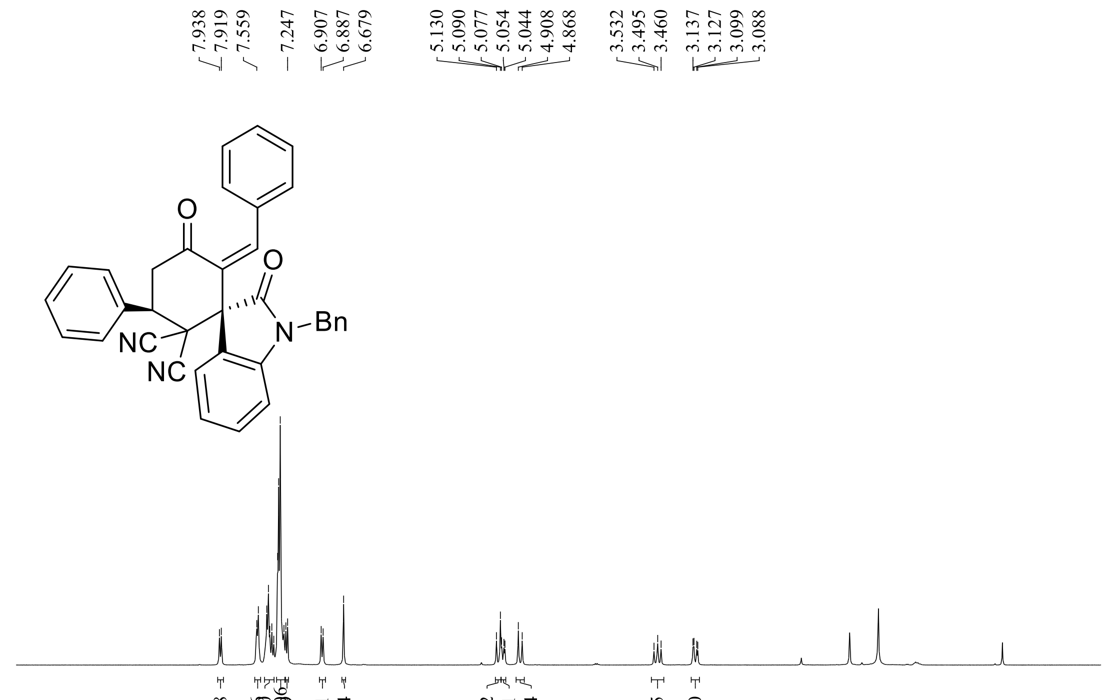

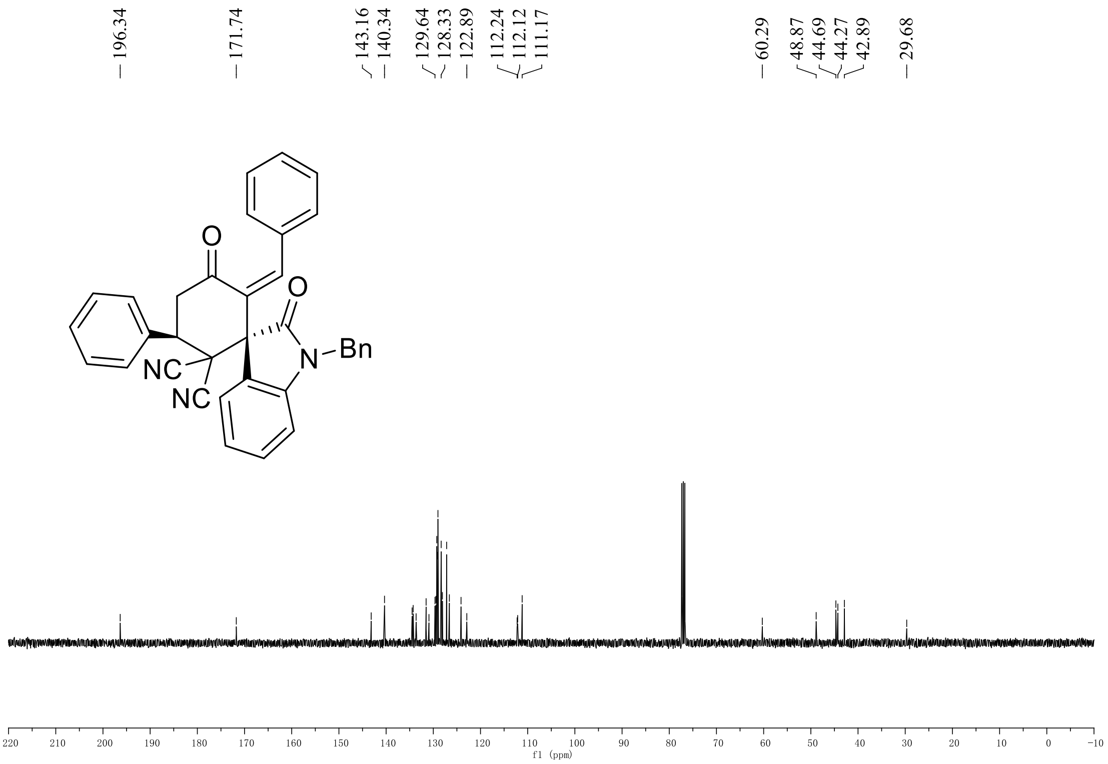
196.34
171.74
143.16
140.34
129.64
128.33
122.89
112.24
112.12
111.17
60.29
48.87
44.69
44.27
42.89
29.68
O
O
N
Bn
NC
NC
f1 (ppm)

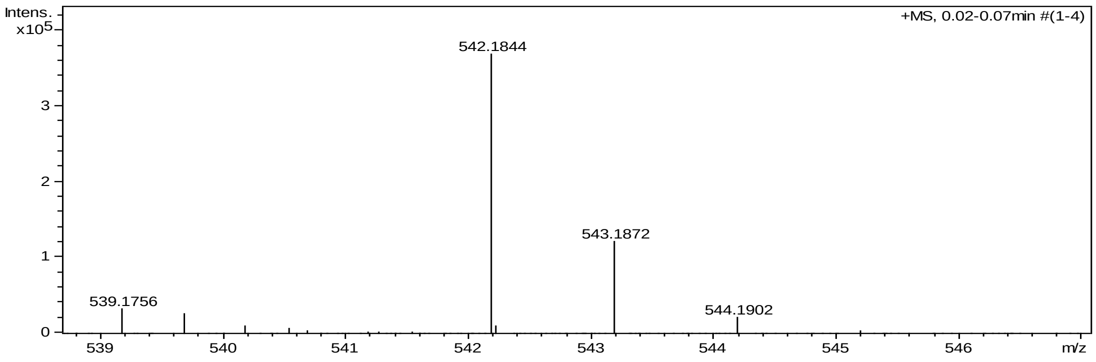
Intens.
x10^5
+MS, 0.02-0.07min #(1-4)
542.1844
543.1872
539.1756
544.1902
m/z

***rel*-(1*R*,3*R*)-1'-Benzyl-6-((*Z*)-4-chlorobenzylidene)-3-(4-chlorophenyl)-2',5-dioxospiro[cyclohexane-1,3'-indoline]-2,2-dicarbonitrile (3v)**: white solid, 45%, m.p. 222-224 °C; ^{1}H NMR (400 MHz, $CDCl_3$) δ 7.90 (d, J = 7.6 Hz, 1H, ArH), 7.49-7.41 (m, 5H, ArH), 7.37-7.29 (m, 10H, ArH), 6.91 (d, J = 8.0 Hz, 1H, ArH), 6.62 (s, 1H, CH), 5.11 (d, J = 16.0 Hz, 1H, CH_2), 5.07-5.03 (m, 1H, CH_2), 4.88 (d, J = 16.0 Hz, 1H, CH_2), 3.39 (t, J = 15.2 Hz, 1H, CH), 3.11-3.06 (m, 1H, CH_2). ^{13}C NMR (100 MHz, $CDCl_3$) δ 195.6, 171.6, 143.1, 139.2, 135.9, 135.6, 134.1, 132.8, 131.9, 131.7, 131.3, 130.7, 130.3, 129.5, 129.0, 128.6, 128.1, 127.2, 126.5, 124.3, 122.6, 112.0, 111.9, 111.3, 60.1, 48.65, 44.7, 44.1, 42.2. IR (KBr) υ: 3726, 3629, 3405, 3063, 2973, 2924, 2853, 2343, 1896, 1709, 1610, 1486, 1410, 1363, 1293, 1187, 1152, 1092, 1049, 1011, 953, 893, 826, 756, 734 cm^{-1}; MS (m/z): HRMS (ESI) Calcd. for $C_{35}H_{23}NaClN_3O_2$ ($[M+Na]^+$): 610.1065, found: 610.1054.

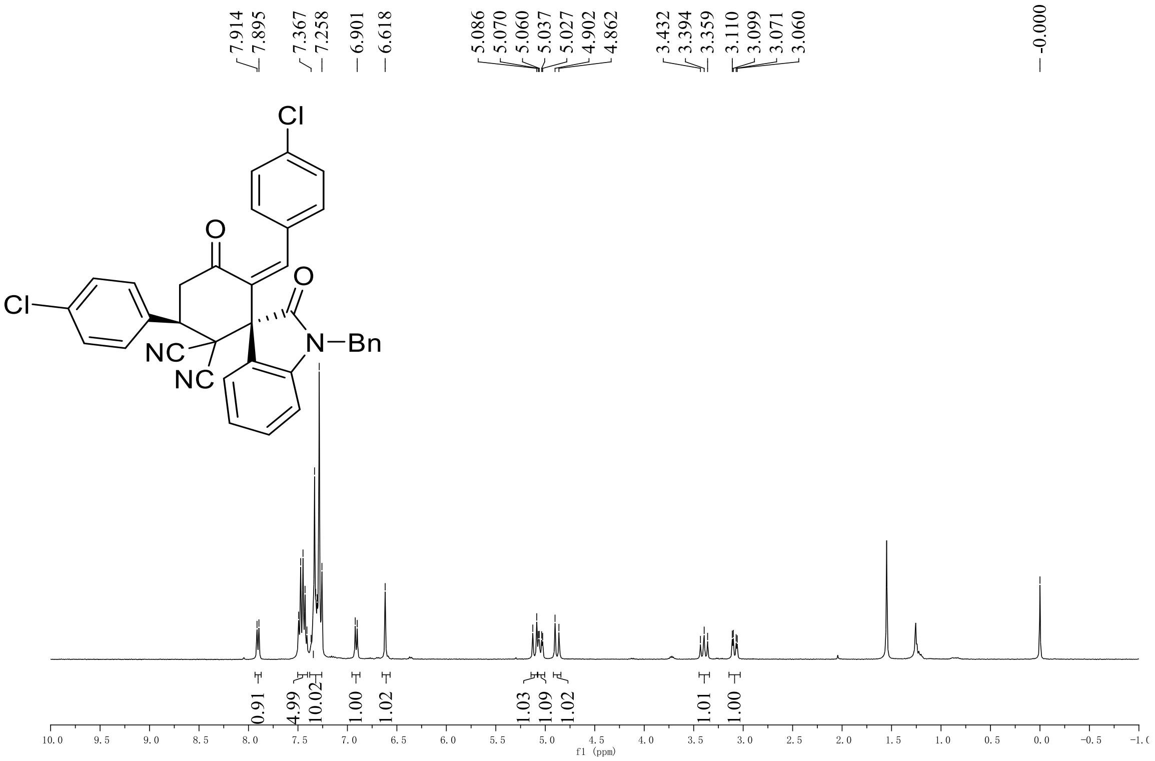

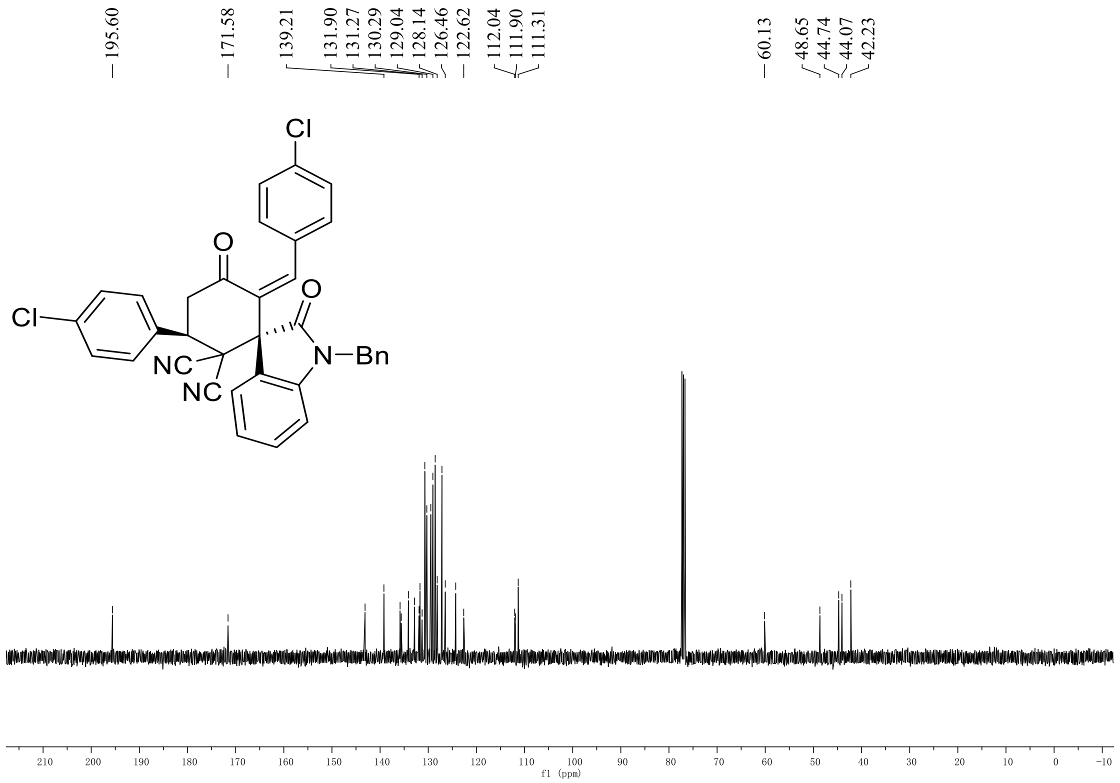
195.60
171.58
139.21
131.90
131.27
130.29
129.04
128.14
126.46
122.62
112.04
111.90
111.31
60.13
48.65
44.74
44.07
42.23
Cl
O
O
Cl
NC
NC
N−Bn
210
200
190
180
170
160
150
140
130
120
110
100
90
80
70
60
50
40
30
20
10
0
−10
f1 (ppm)

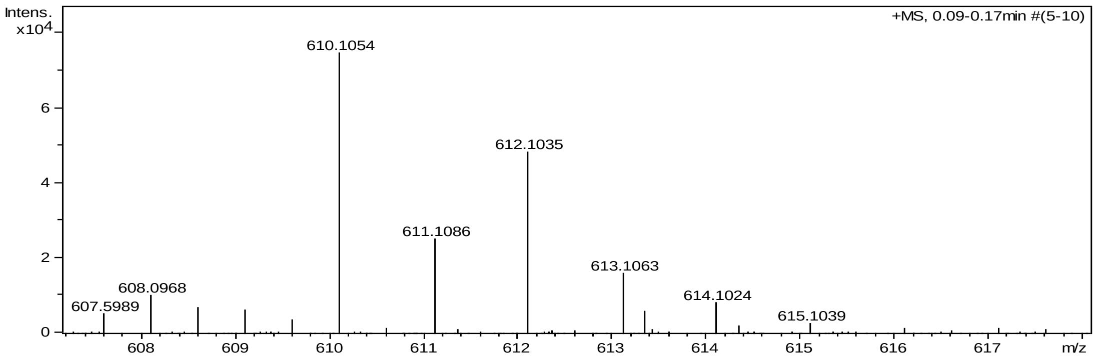
Intens.
x10^4
+MS, 0.09-0.17min #(5-10)
610.1054
612.1035
611.1086
613.1063
608.0968
607.5989
614.1024
615.1039
6
4
2
0
608
609
610
611
612
613
614
615
616
617
m/z

***rel*-(1*R*,3*R*)-1'-Benzyl-6-((*Z*)-4-bromobenzylidene)-3-(4-bromophenyl)-2',5-dioxospiro[cyclohexane-1,3'-indoline]-2,2-dicarbonitrile (3w)**: white solid, 47%, m.p. 221-223 °C; ^{1}H NMR (400 MHz, $CDCl_3$) δ 7.80 (d, *J* = 7.2 Hz, 1H, ArH), 7.59 (d, *J* = 8.0 Hz, 2H, ArH), 7.45-7.40 (m, 5H, ArH), 7.33-7.28 (m, 6H, ArH), 7.21 (d, *J* = 8.0 Hz, 2H, ArH), 6.91 (d, *J* = 8.0 Hz, 1H, ArH), 6.60 (s, 1H, CH), 5.10 (d, *J* = 15.6 Hz, 1H, CH_2), 5.06-5.01 (m, 1H, CH_2), 4.88 (d, *J* = 16.0 Hz, 1H, CH_2), 3.39 (t, *J* = 14.4 Hz, 1H, CH), 3.10-3.06 (m, 1H, CH_2).^{13}C NMR (100 MHz, $CDCl_3$) δ 195.6, 171.5, 143.1, 139.3, 134.1, 133.4, 132.4, 132.3, 131.7, 131.5, 131.4, 130.9, 130.6, 129.1, 128.2, 127.2, 126.5, 124.3, 124.1, 123.9, 122.6, 112.0, 111.9, 111.3, 60.1, 48.5, 44.8, 44.0, 42.3. IR (KBr) υ: 3727, 3632, 3404, 3063, 2971, 2928, 2344, 1896, 1709, 1610, 1483, 1409, 1361, 1292, 1186, 1152, 1118, 1069, 1005, 951, 893, 821, 753 cm^{-1}; MS (*m*/*z*): HRMS (ESI) Calcd. for $C_{35}H_{23}NaBr_2N_3O_2$ ($[M+Na]^+$): 698.0055, found: 698.0007.

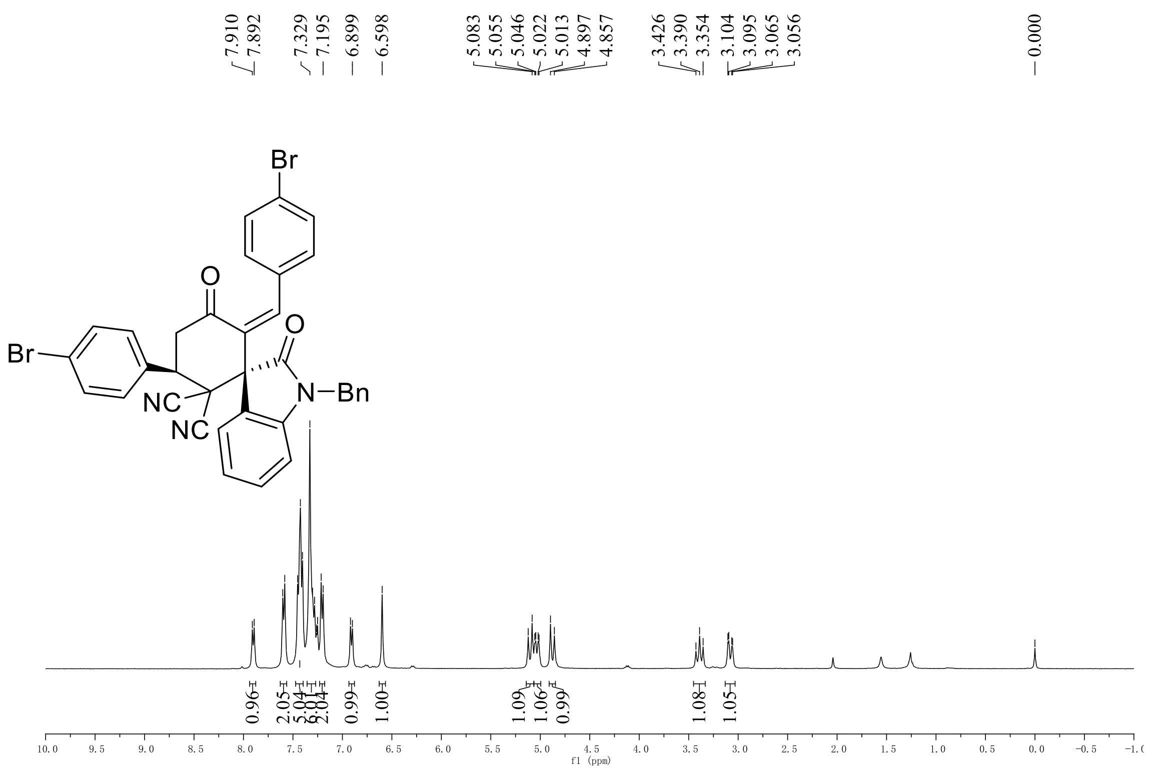

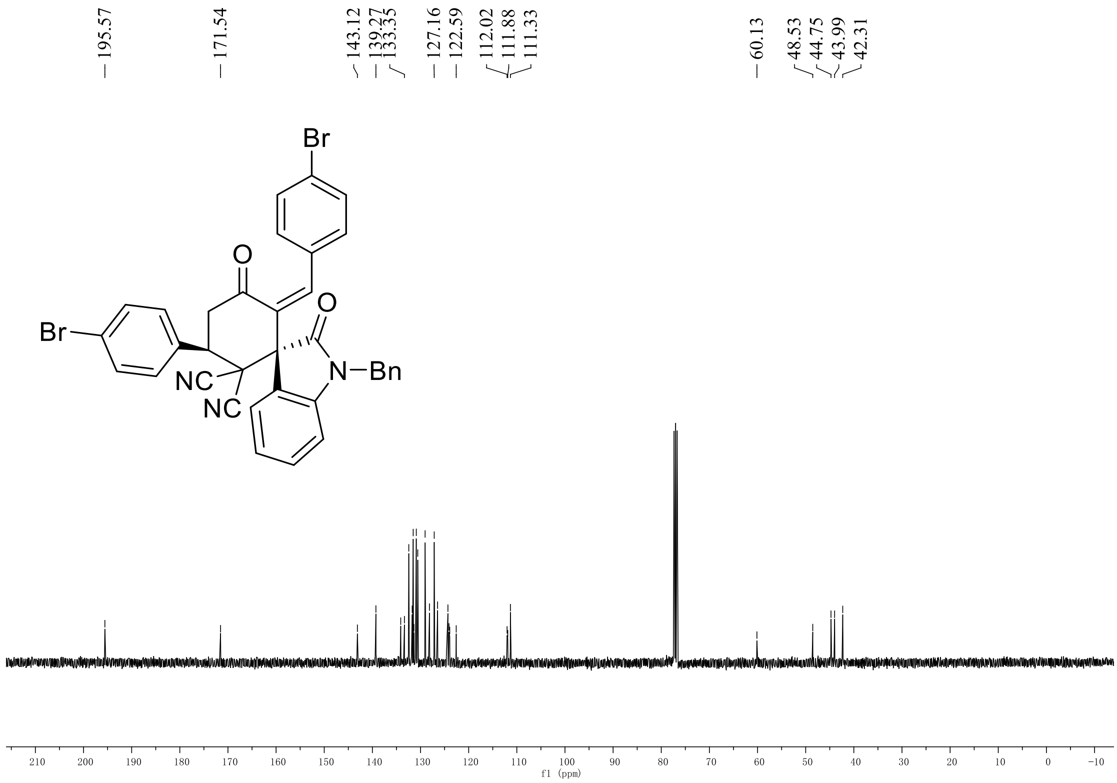
195.57
171.54
143.12
139.27
133.35
127.16
122.59
112.02
111.88
111.33
60.13
48.53
44.75
43.99
42.31
Br
O
O
Br
N−Bn
NC
NC
f1 (ppm)

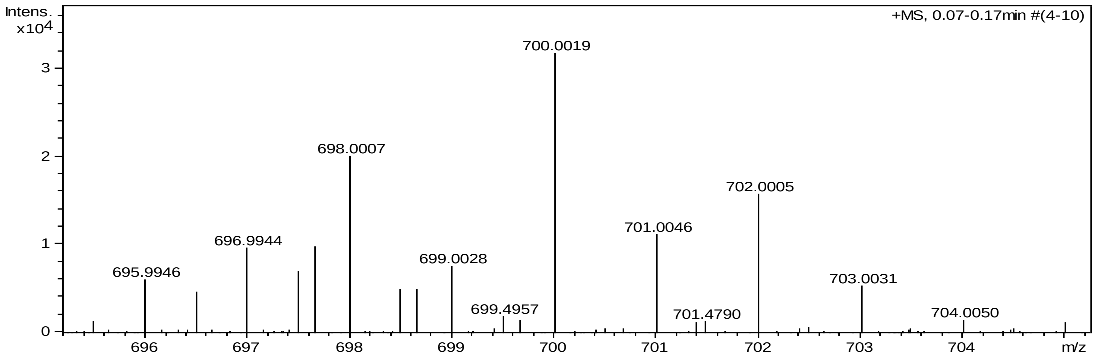
Intens.
x10^4
+MS, 0.07-0.17min #(4-10)
700.0019
698.0007
702.0005
701.0046
696.9944
695.9946
699.0028
703.0031
699.4957
701.4790
704.0050
m/z

***rel*-(1*R*,3*S*,*Z*)-1',5'-Dimethyl-2',5-dioxo-3-(thiophen-2-yl)-6-(thiophen-2-ylmethylene)spiro[cyclohexane-1,3'-indoline]-2,2-dicarbonitrile (3x)**: yellow solid, 52%, m.p. 154-156 °C; ^{1}H NMR (400 MHz, $CDCl_3$) δ 7.66 (s, 1H, ArH), 7.54 (d, *J* = 4.4 Hz, 1H, ArH), 7.37 (d, *J* = 5.6 Hz, 2H, ArH), 7.30 (S, 1H, ArH), 7.23 (s, 1H, ArH), 7.07-7.03 (m, 2H, ArH), 6.95 (d, *J* = 8.0 Hz, 1H, ArH), 6.78 (s, 1H, CH), 5.36 (t, *J* = 8.4 Hz, 1H, CH), 3.30 (s, 2H, CH_2), 3.28 (s, 3H, CH_3), 2.48 (s, 3H, CH_3). ^{13}C NMR (100 MHz, $CDCl_3$) δ 194.0, 172.2, 141.7, 138.5, 137.7, 136.3, 135.6, 134.3, 133.6, 132.1, 128.0, 127.4, 127.0, 126.7, 126.5, 123.9, 122.5, 112.3, 112.2, 109.8, 59.6, 49.2, 44.4, 37.8, 27.0, 21.5. IR (KBr) υ: 3458, 3108, 2905, 2352, 2318, 1707, 1610, 1587, 1490, 1464, 1424, 1388, 1355, 1328, 1264, 1105, 1063, 856, 812, 779, 706 cm^{-1}; MS (*m*/*z*): HRMS (ESI) Calcd. for $C_{26}H_{19}NaN_3O_2S_2$ ($[M+Na]^+$): 492.0816, found: 492.0806.

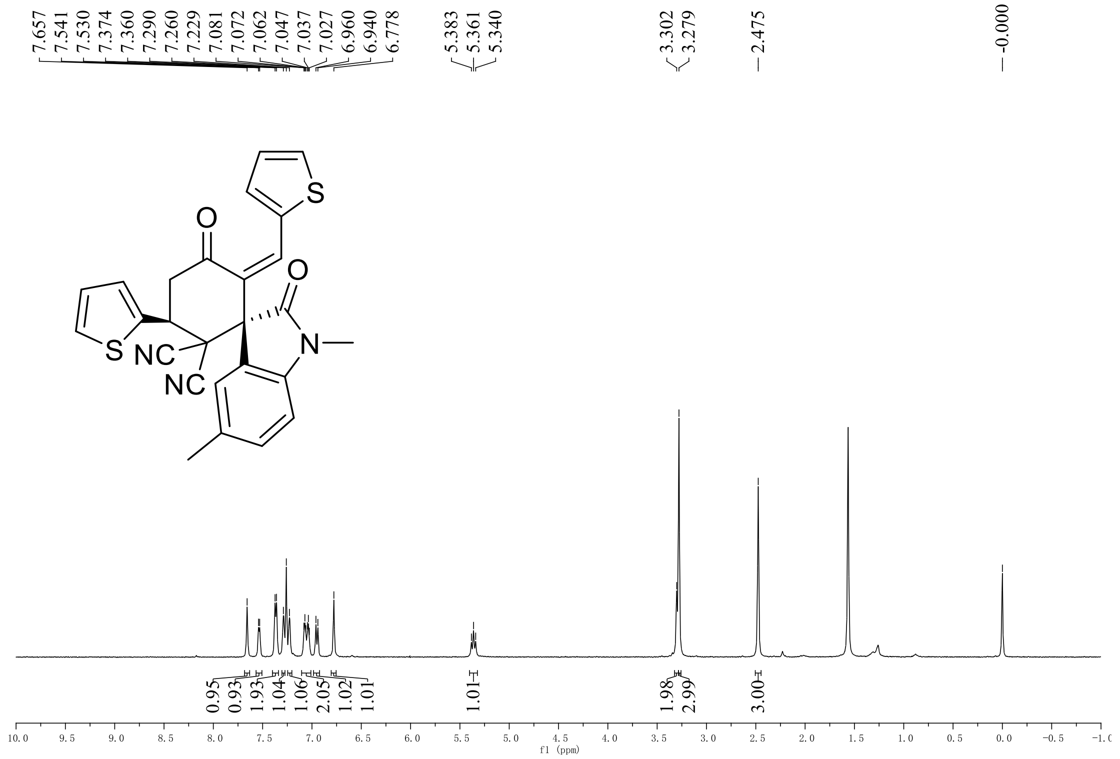

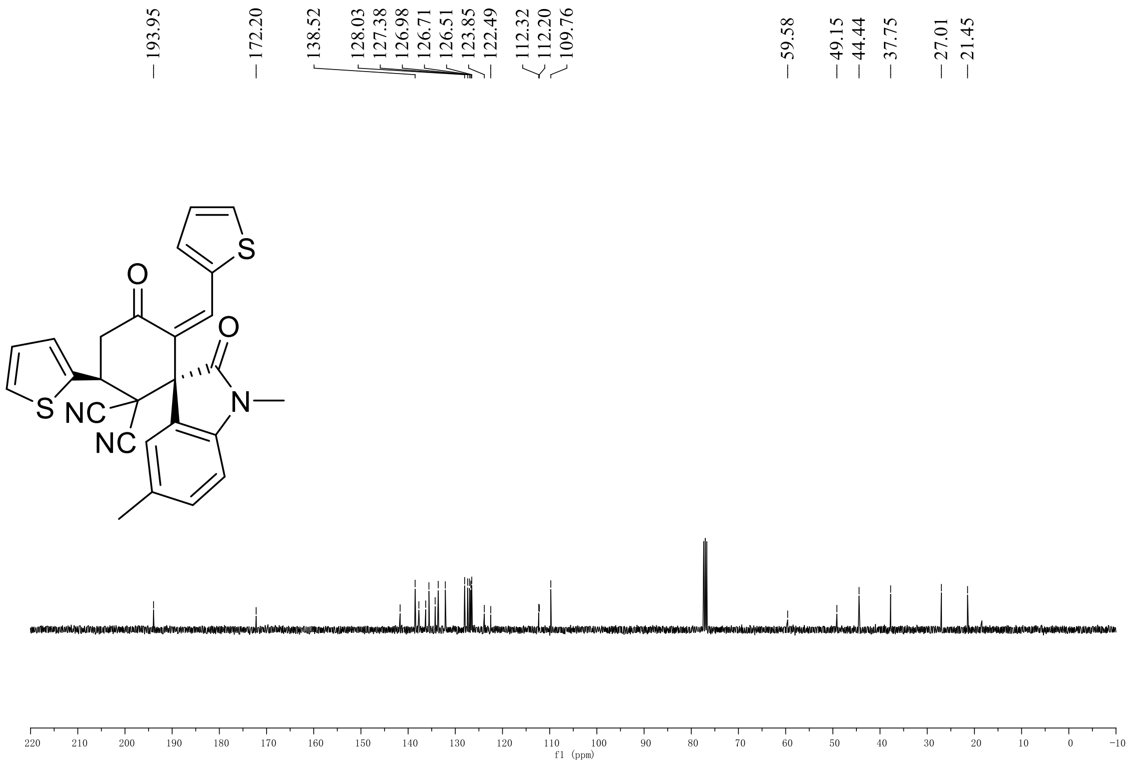
193.95
172.20
138.52
128.03
127.38
126.98
126.71
126.51
123.85
122.49
112.32
112.20
109.76
59.58
49.15
44.44
37.75
27.01
21.45
O
S
O
S
NC
NC
N
220 210 200 190 180 170 160 150 140 130 120 110 100 90 80 70 60 50 40 30 20 10 0 −10
f1 (ppm)

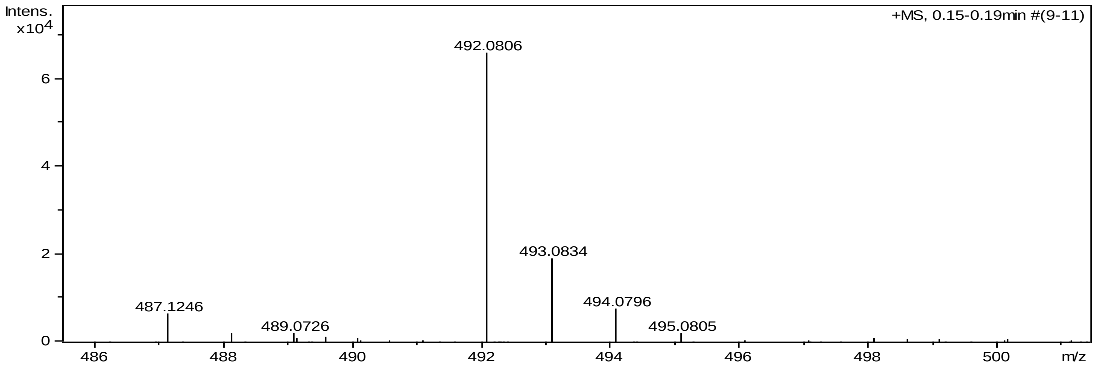
Intens.
x10^4
+MS, 0.15-0.19min #(9-11)
492.0806
493.0834
494.0796
487.1246
489.0726
495.0805
0 2 4 6
486 488 490 492 494 496 498 500 m/z

***rel*-(1*R*,3*S*,*Z*)-5'-Chloro-1'-methyl-2',5-dioxo-3-(thiophen-2-yl)-6-(thiophen-2-ylmethylene)spiro[cyclohexane-1,3'-indoline]-2,2-dicarbonitrile (3y)**: pale yellow solid, 45%, m.p. 160-162 °C; ^{1}H NMR (400 MHz, $CDCl_3$) δ 7.85 (s, 1H, ArH), 7.56 (d, *J* = 4.4 Hz, 2H, ArH), 7.38-7.28 (m, 3H, ArH), 7.07-7.00 (m, 3H, ArH), 6.74 (s, 1H, CH), 5.32 (t, *J* = 8.4 Hz, 1H, CH), 3.30 (s, 5H, CH_3, CH_2). ^{13}C NMR (100 MHz, $CDCl_3$) δ 174.9, 171.0, 161.3, 142.7, 142.5, 131.0, 130.3, 128.9, 128.5, 126.8, 124.8, 121.6, 119.2, 113.3, 112.7, 109.4, 109.1, 63.1, 60.2, 27.5, 26.9, 26.4, 26.3, 24.0, 23.9, 23.7, 23.7, 13.5. IR (KBr) υ: 3458, 3108, 2905, 2352, 2318, 1707, 1610, 1587, 1490, 1464, 1424, 1388, 1355, 1328, 1264, 1105, 1063, 856, 812, 779, 706 cm^{-1}; MS (*m*/*z*): HRMS (ESI) Calcd. for $C_{25}H_{16}ClNaN_3O_2S_2$ ([M+Na]$^{+}$): 512.0270, found: 512.0257.

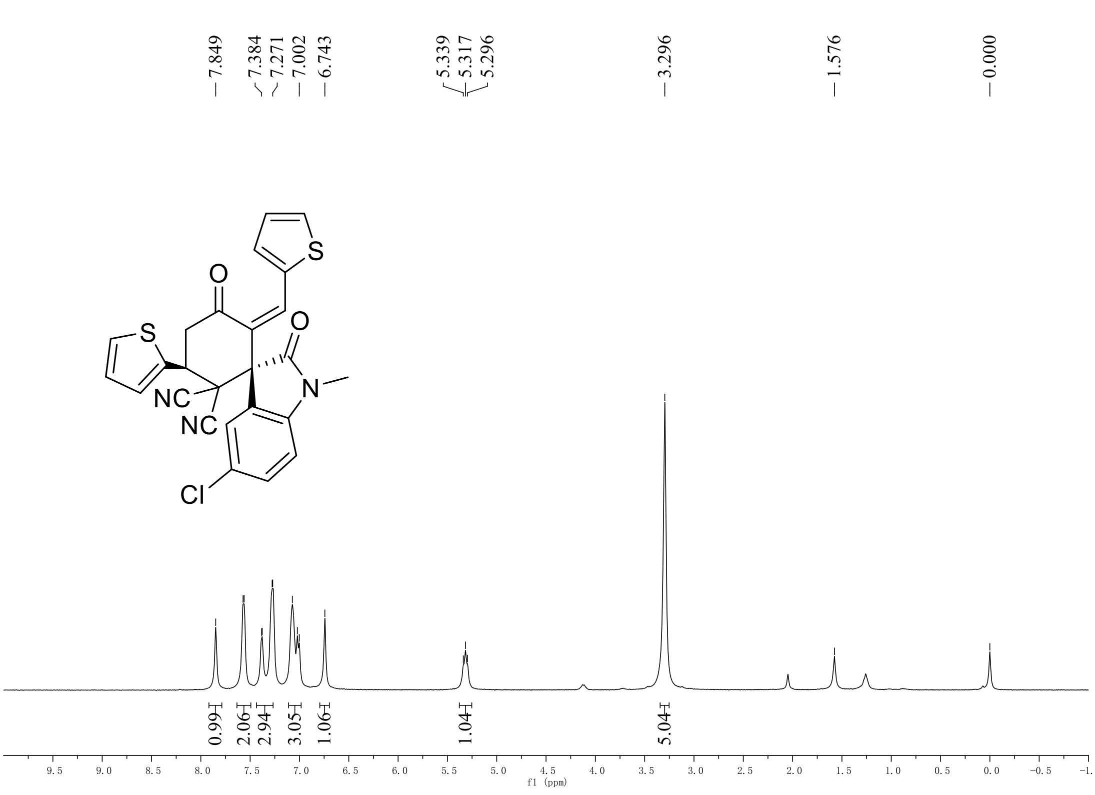

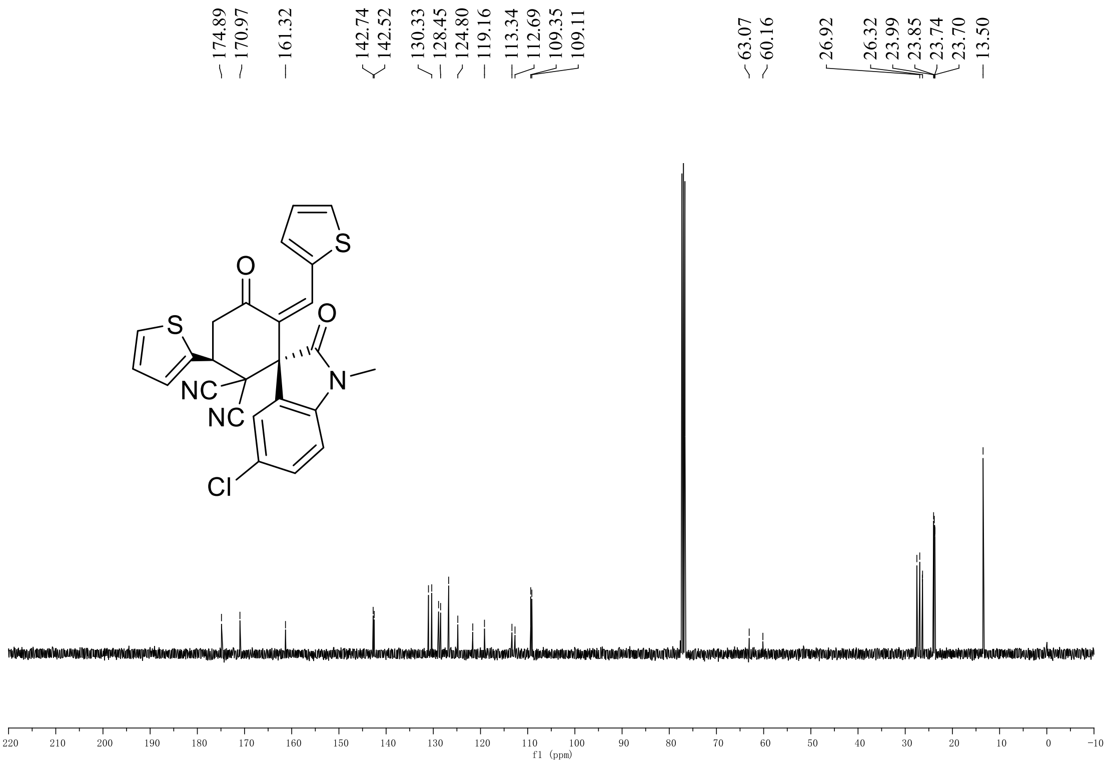
174.89
170.97
161.32
142.74
142.52
130.33
128.45
124.80
119.16
113.34
112.69
109.35
109.11
63.07
60.16
26.92
26.32
23.99
23.85
23.74
23.70
13.50
O
S
O
S
N
NC
NC
Cl
220 210 200 190 180 170 160 150 140 130 120 110 100 90 80 70 60 50 40 30 20 10 0 −10
f1 (ppm)

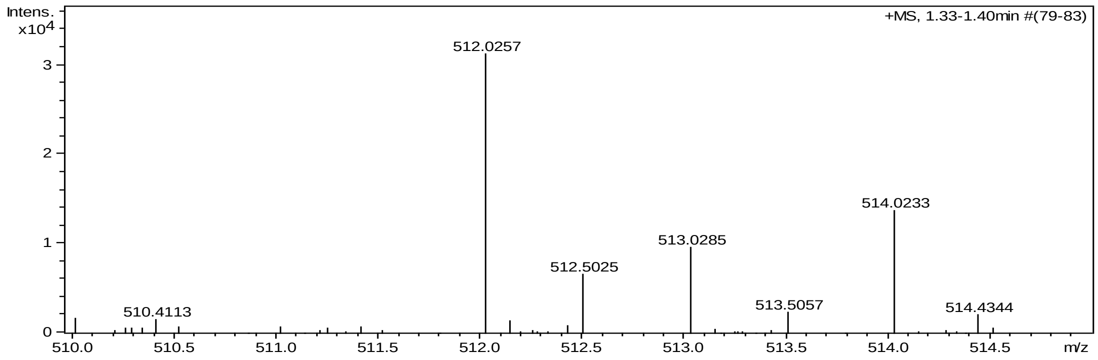
Intens.
x10^4
+MS, 1.33-1.40min #(79-83)
512.0257
514.0233
513.0285
512.5025
513.5057
514.4344
510.4113
0
1
2
3
510.0
510.5
511.0
511.5
512.0
512.5
513.0
513.5
514.0
514.5
m/z

***rel*-(1*R*,3*S*,*Z*)-1'-Benzyl-2',5-dioxo-3-(thiophen-2-yl)-6-(thiophen-2-ylmethylene)spiro[cyclohexane-1,3'-indoline]-2,2-dicarbonitrile (1z)**: yellow solid, 53%, m.p. 158-160 °C; ^{1}H NMR (400 MHz, $CDCl_3$) δ 7.88 (d, *J* = 7.6 Hz, 1H, ArH), 7.55 (d, *J* = 4.8 Hz, 1H, ArH), 7.46-7.42 (m, 1H, ArH), 7.38 (d, *J* = 4.8 Hz, 1H, ArH), 7.33-7.29 (m, 7H, ArH), 7.19 (d, *J* = 3.2 Hz, 1H, ArH), 7.09-7.08 (m, 1H, ArH), 7.06-7.03 (m, 1H, ArH), 6.90 (d, *J* = 7.6 Hz, 1H, ArH), 6.75 (s, 1H, CH), 5.43 (t, *J* = 8.8 Hz, 1H, CH), 5.05 (d, *J* = 15.6 Hz, 1H, CH_2), 4.91 (d, *J* = 15.6 Hz, 1H, CH_2), 3.33 (d, *J* = 9.2 Hz, 2H, CH_2). ^{13}C NMR (100 MHz, $CDCl_3$) δ 193.9, 172.7, 143.3, 138.8, 137.6, 136.3, 135.8, 134.2, 133.8, 131.6, 129.0, 128.2, 128.1, 127.4, 127.2, 127.1, 126.6, 126.3, 124.4, 124.0, 122.6, 112.3, 111.2, 59.3, 48.9, 44.7, 44.4, 37.9. IR (KBr) υ: 3095, 2914, 1708, 1604, 1488, 1466, 1417, 1366, 1271, 1233, 1188, 1059, 948, 894, 845, 756, 724 cm^{-1}; MS (*m*/*z*): HRMS (ESI) Calcd. for $C_{31}H_{21}NaN_3O_2S_2$ ($[M+Na]^+$): 554.0973, found: 554.0961.

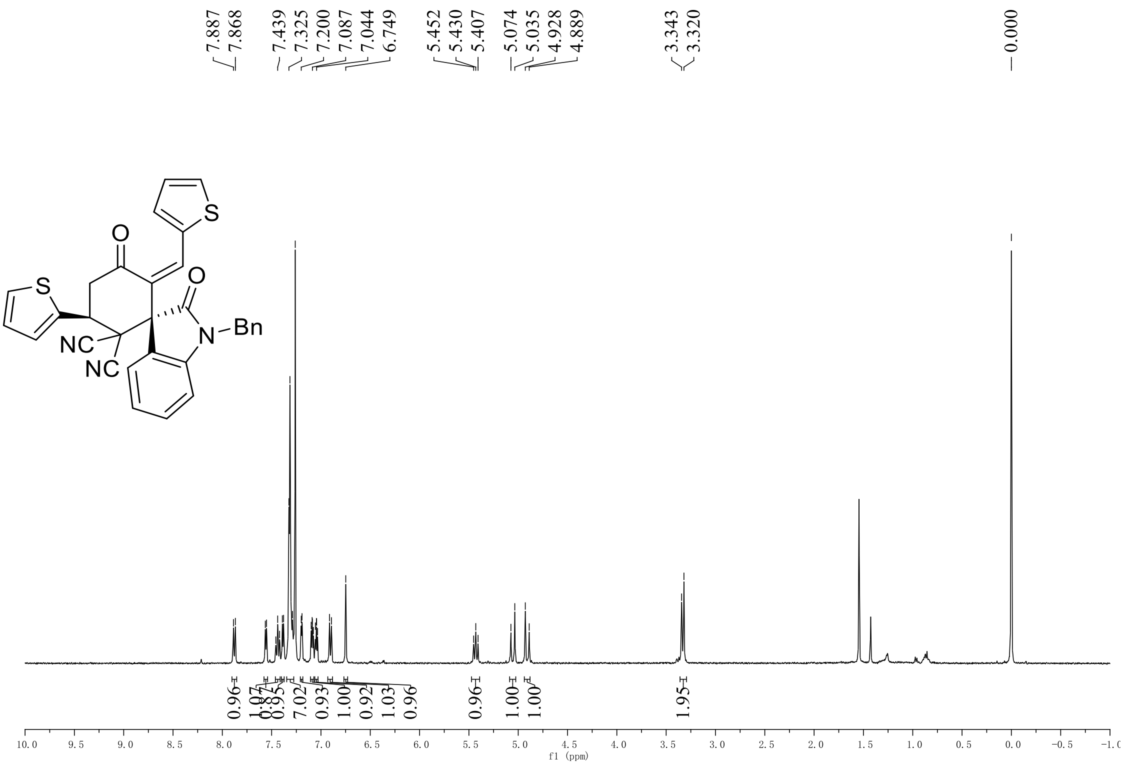

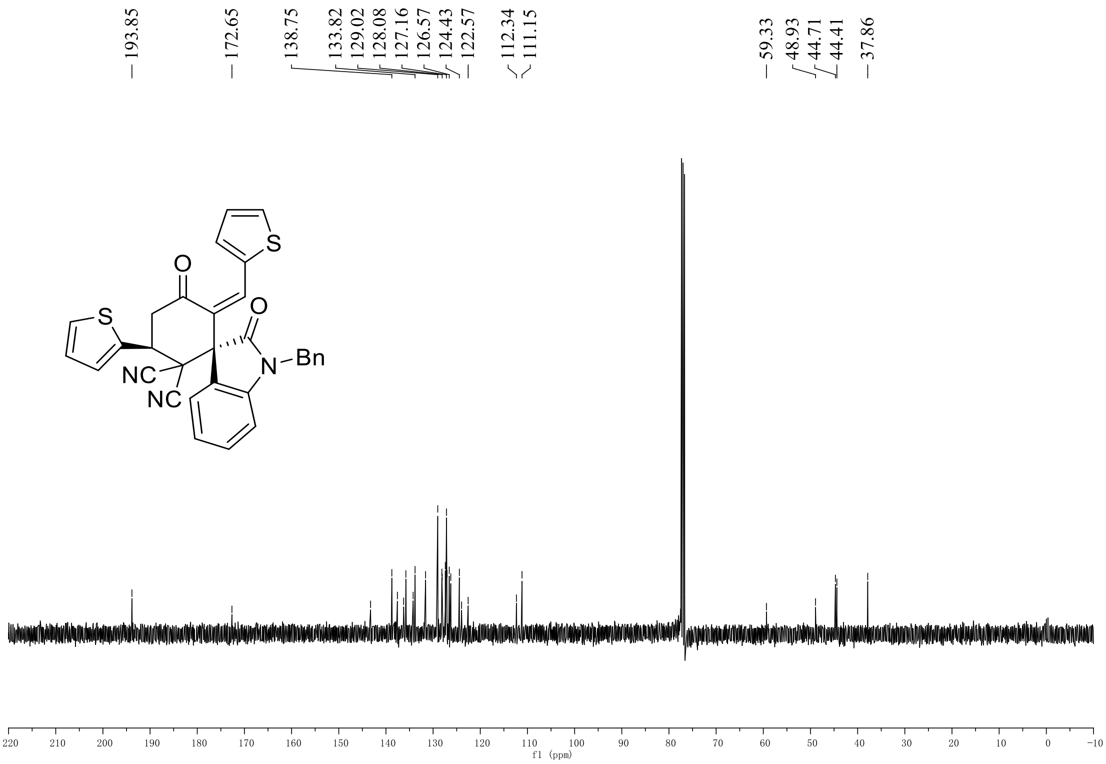

193.85
172.65
138.75
133.82
129.02
128.08
127.16
126.57
124.43
122.57
112.34
111.15
59.33
48.93
44.71
44.41
37.86
O
S
O
S
N
Bn
NC
NC
220 210 200 190 180 170 160 150 140 130 120 110 100 90 80 70 60 50 40 30 20 10 0 −10
f1 (ppm)

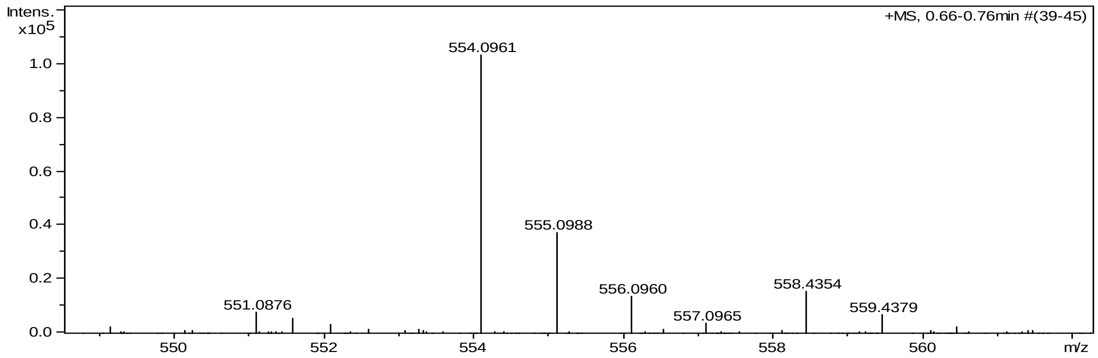

Intens.
x10^5
+MS, 0.66-0.76min #(39-45)
554.0961
555.0988
556.0960
557.0965
558.4354
559.4379
551.0876
1.0
0.8
0.6
0.4
0.2
0.0
550
552
554
556
558
560
m/z

***rel*-Ethyl (1*S*,2*S*,6*R*)-1'-benzyl-5'-chloro-3-((*E*)-4-methylbenzylidene)-2',4-dioxo-6-(*p*-tolyl)spiro[cyclohexane-1,3'-indoline]-2-carboxylate (5a)**: white solid, 60%, m.p. 221-223 °C; ^{1}H NMR (400 MHz, $CDCl_3$) δ 7.67 (s, 1H, ArH), 7.29 (d, *J* = 7.6 Hz, 2H, ArH), 7.21-7.14 (m, 4H, ArH), 7.07 (t, *J* = 7.6 Hz, 2H, ArH), 7.01 (d, *J* = 8.4 Hz, 1H, ArH), 6.91 (d, *J* = 8.0 Hz, 2H, ArH), 6.84 (d, *J* = 8.0 Hz, 2H, ArH), 6.77 (d, *J* = 7.6 Hz, 2H, ArH), 6.34 (d, *J* = 8.4 Hz, 1H, CH), 4.70 (d, *J* = 16.0 Hz, 1H, CH_2), 4.57 (d, *J* = 15.6 Hz, 1H, CH_2), 4.52-4.47 (m, 1H, CH_2), 4.36-4.24 (m, 2H, OCH_2), 3.94 (s, 1H, CH), 3.80-3.72 (m, 1H, CH), 3.00-2.94 (m, 1H, CH_2), 2.37 (s, 3H, CH_3), 2.20 (s, 3H, CH_3), 1.30 (t, *J* = 7.2 Hz, 3H, CH_3). ^{13}C NMR (100 MHz, $CDCl_3$) δ 200.0, 175.1, 170.6, 140.6, 139.0, 138.8, 136.8, 135.0, 134.8, 131.6, 131.2, 130.1, 129.8, 129.3, 129.0, 128.6, 128.5, 127.5, 127.4, 127.1, 124.5, 110.2, 61.8, 53.0, 50.7, 43.4, 42.5, 42.0, 21.4, 21.0, 14.1. IR (KBr) υ: 3723, 3412, 2933, 2871, 2324, 1925, 1817, 1703, 1604, 1474, 1442, 1339, 1172, 1091, 1010, 904, 824, 716 cm^{-1}; MS (*m/z*): HRMS (ESI) Calcd. for $C_{38}H_{34}ClNaNO_4$ ([M+Na]$^+$): 626.2069, found: 626.2066.

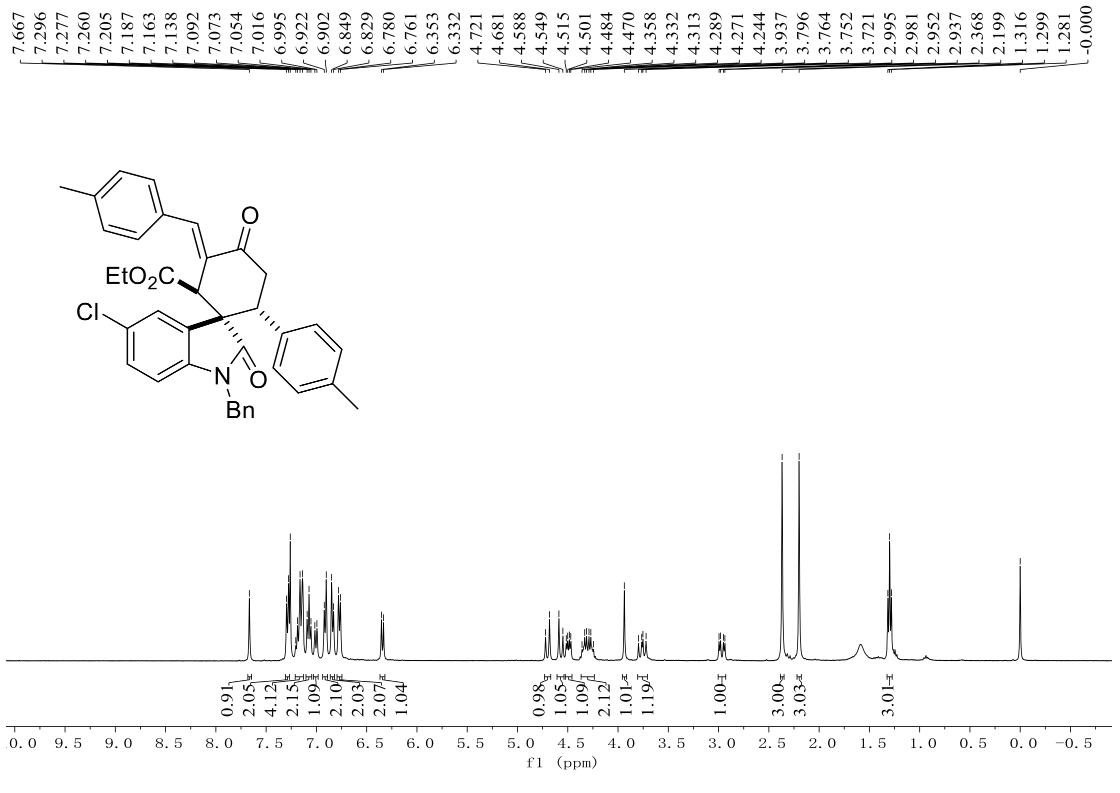

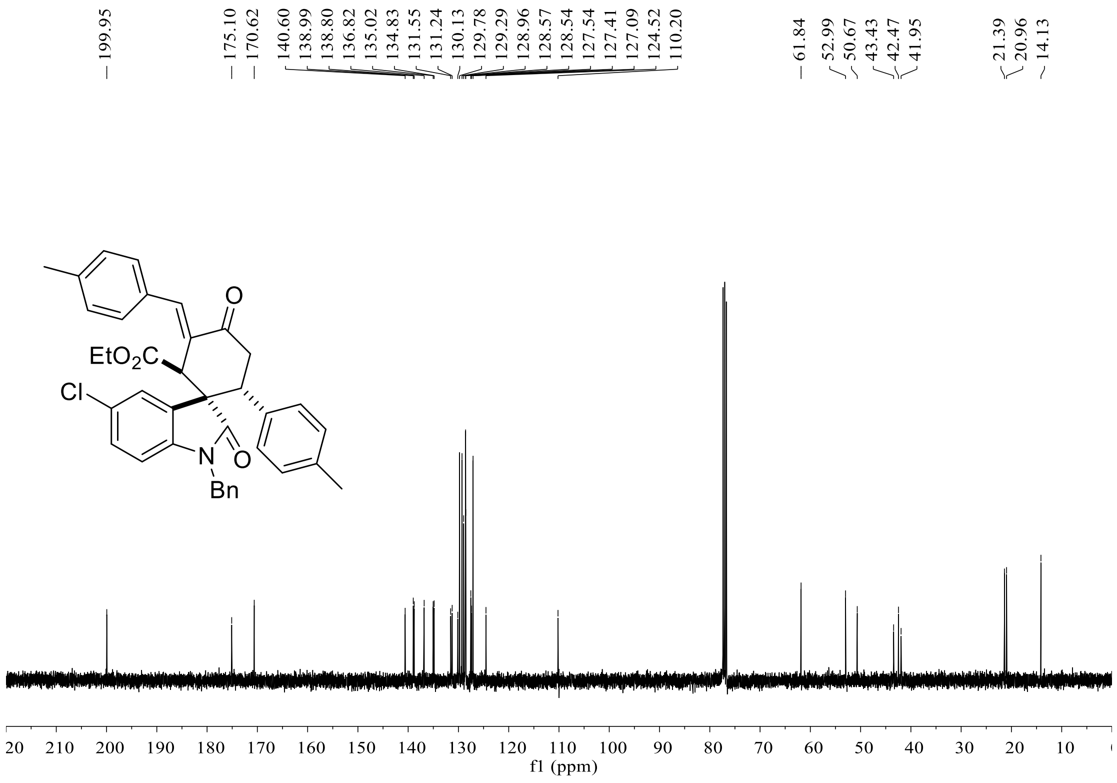
199.95
175.10
170.62
140.60
138.99
138.80
136.82
135.02
134.83
131.55
131.24
130.13
129.78
129.29
128.96
128.57
128.54
127.54
127.41
127.09
124.52
110.20
61.84
52.99
50.67
43.43
42.47
41.95
21.39
20.96
14.13
O
EtO2C
Cl
N
O
Bn
f1 (ppm)

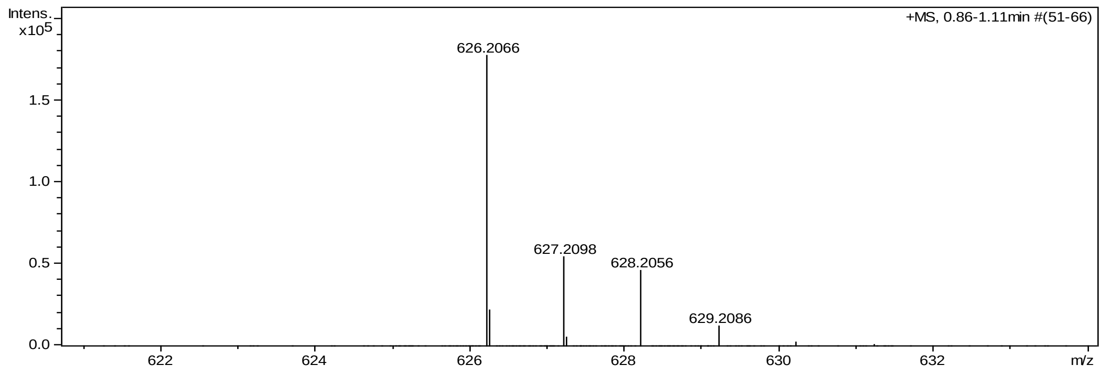
Intens.
x10^5
+MS, 0.86-1.11min #(51-66)
626.2066
627.2098
628.2056
629.2086
m/z

***rel*-Ethyl (1*S*,2*S*,6*R*)-1'-benzyl-5'-chloro-3-((*E*)-4-methoxybenzylidene)-6-(4-methoxyphenyl)-2',4-dioxospiro[cyclohexane-1,3'-indoline]-2-carboxylate (5b)**: white solid, 57%, m.p. 231-233 °C; ^{1}H NMR (400 MHz, $CDCl_3$) δ 7.65 (s, 1H, ArH), 7.39 (d, *J* = 8.0 Hz, 2H, ArH), 7.20-7.14 (m, 2H, ArH), 7.09 (t, *J* = 7.2 Hz, 2H, ArH), 7.02 (d, *J* = 8.0 Hz, 1H, ArH), 6.94 (d, *J* = 8.0 Hz, 2H, ArH), 6.87 (d, *J* = 8.4 Hz, 2H, ArH), 6.77 (d, *J* = 6.8 Hz, 2H, ArH), 6.56 (d, *J* = 8.0 Hz, 2H, ArH), 6.37 (d, *J* = 8.0 Hz, 1H, CH), 4.70 (d, *J* = 15.6 Hz, 1H, CH_2), 4.56 (d, *J* = 16.0 Hz, 1H, CH_2), 4.51-4.47 (m, 1H, CH_2), 4.37-4.29 (m, 2H, OCH_2), 3.96 (s, 1H, CH), 3.82 (s, 3H, OCH_3), 3.76-3.72 (m, 1H, CH), 3.69 (s, 3H, OCH_3), 2.99-2.94 (m, 1H, CH_2), 1.31 (t, *J* = 7.2 Hz, 3H, CH_3). ^{13}C NMR (100 MHz, $CDCl_3$) δ 199.8, 175.1, 170.6, 160.2, 158.5, 140.6, 138.6, 134.8, 131.8, 130.3, 130.2, 129.9, 129.7, 128.6, 128.6, 127.6, 127.5, 127.1, 126.9, 124.5, 114.0, 113.5, 110.2, 61.9, 55.3, 55.0, 53.1, 50.6, 43.4, 42.0, 41.9, 14.2. IR (KBr) υ: 3734, 3410, 2940, 2843, 2421, 1930, 1843, 1715, 1623, 1443, 1340, 1065, 1003, 876, 843, 721 cm^{-1}; MS (*m/z*): HRMS (ESI) Calcd. for $C_{38}H_{34}ClNaNO_6$ ($[M+Na]^+$): 658.1967, found: 658.1973.

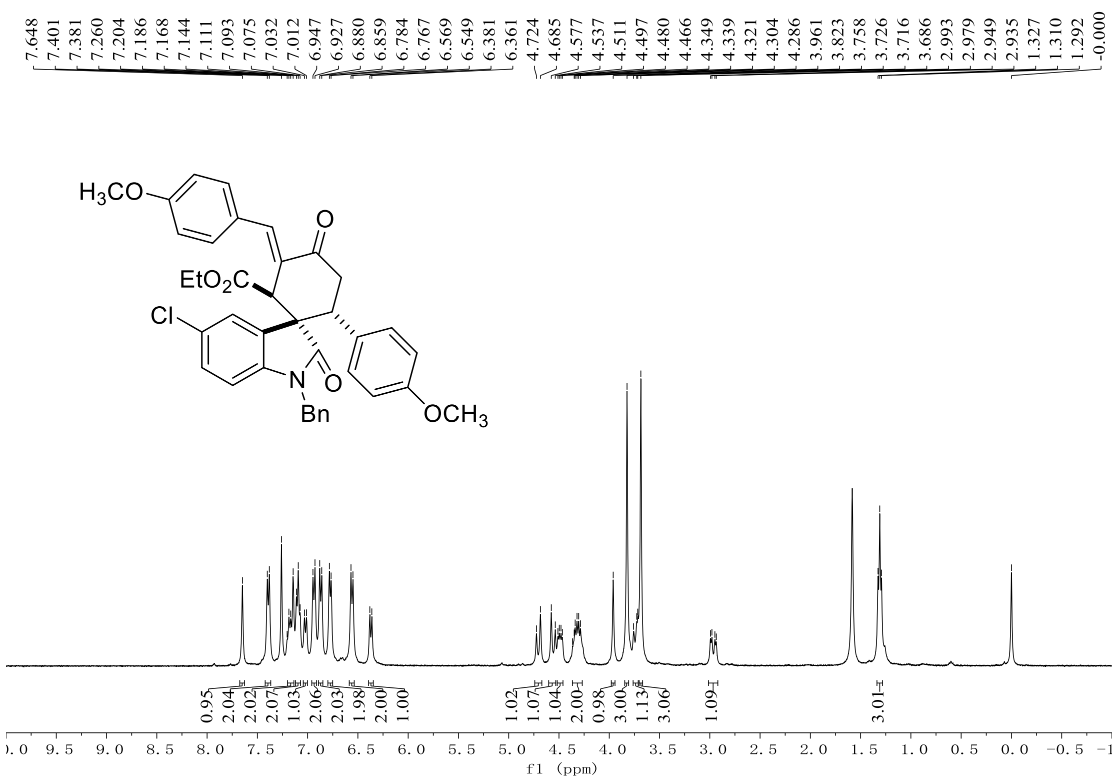

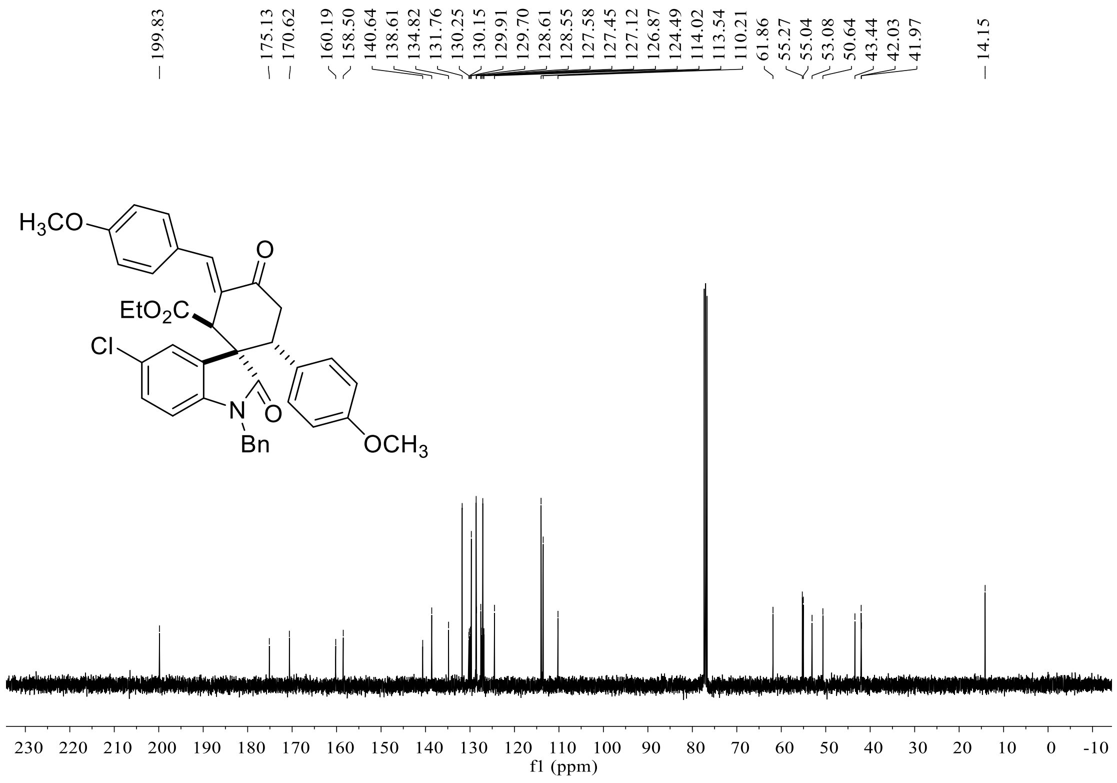
199.83
175.13
170.62
160.19
158.50
140.64
138.61
134.82
131.76
130.25
130.15
129.91
129.70
128.61
128.55
127.58
127.45
127.12
126.87
124.49
114.02
113.54
110.21
61.86
55.27
55.04
53.08
50.64
43.44
42.03
41.97
14.15
H_3CO
O
EtO_2C
Cl
N
O
Bn
OCH_3
230 220 210 200 190 180 170 160 150 140 130 120 110 100 90 80 70 60 50 40 30 20 10 0 -10
f1 (ppm)

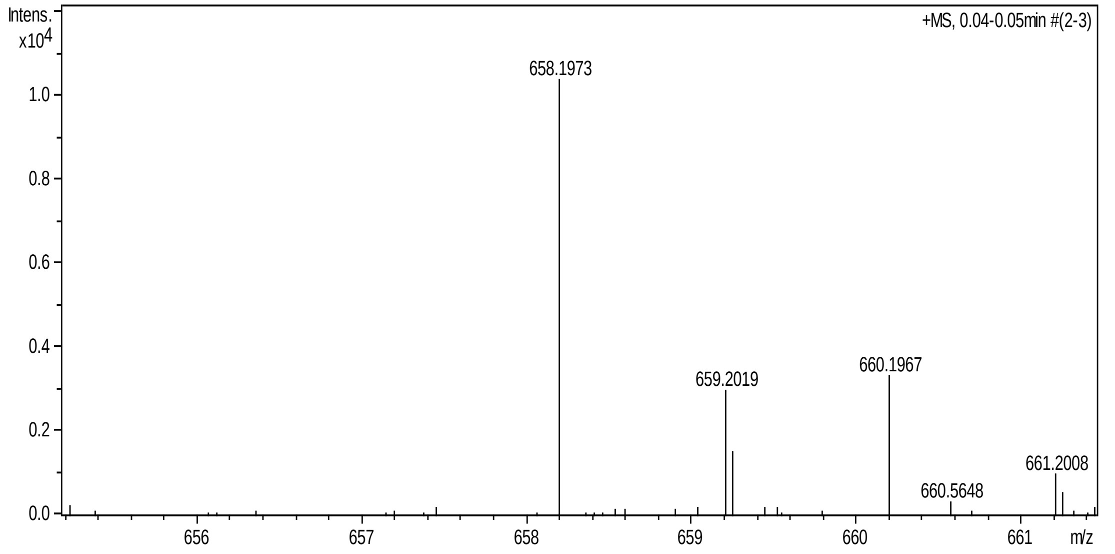
Intens.
x10^4
+MS, 0.04-0.05min #(2-3)
658.1973
659.2019
660.1967
660.5648
661.2008
1.0
0.8
0.6
0.4
0.2
0.0
656
657
658
659
660
661
m/z

***rel*-Methyl (1*S*,2*S*,6*R*)-1'-benzyl-5'-chloro-3-((*E*)-4-methylbenzylidene)-2',4-dioxo-6-(*p*-tolyl)spiro[cyclohexane-1,3'-indoline]-2-carboxylate (5c)**: white solid, 42%, m.p. 225-227 °C; **ratio of major/minor = 10:1**, ^{1}H NMR (400 MHz, $CDCl_3$) δ 7.66 (s, 1H, ArH), 7.27 (s, 1H, ArH), 7.20-7.12 (m, 4H, ArH), 7.09-7.05 (m, 3H, ArH), 7.01 (d, *J* = 8.4 Hz, 1H, ArH), 6.91 (d, *J* = 7.6 Hz, 2H, ArH), 6.84 (d, *J* = 7.6 Hz, 2H, ArH), 6.77 (d, *J* = 7.6 Hz, 2H, ArH), 6.32 (d, *J* = 8.4 Hz, 1H, CH), 4.72 (d, *J* = 15.6 Hz, 1H, CH_2), 4.55 (d, *J* = 16.0 Hz, 1H, CH_2), 4.48-4.43 (m, 1H, CH_2), 3.99 (s, 1H, CH), 3.82 (s, 3H, OCH_3), 3.78-3.73 (m, 1H, CH), 2.99-2.93 (m, 1H, CH_2), 2.37 (s, 3H, CH_3), 2.20 (s, 3H, CH_3). ^{13}C NMR (100 MHz, $CDCl_3$) δ 200.0, 175.1, 171.1, 140.6, 139.1, 138.9, 136.9, 134.9, 134.8, 131.5, 131.0, 130.1, 129.8 129.4, 129.0, 128.6, 128.5, 127.6, 127.5, 127.1, 124.3, 110.3, 53.0, 52.4, 50.7, 43.4, 42.5, 42.0, 21.4, 21.0. IR (KBr) υ: 3684, 3432, 2943, 2834, 2356, 1811, 1724, 1605, 1424, 1443, 1345, 1123, 1080, 904, 859, 746 cm^{-1}; MS (*m*/*z*): HRMS (ESI) Calcd. for $C_{37}H_{32}ClNaNO_4$ ([M+Na]$^+$): 612.1912, found: 612.1893.

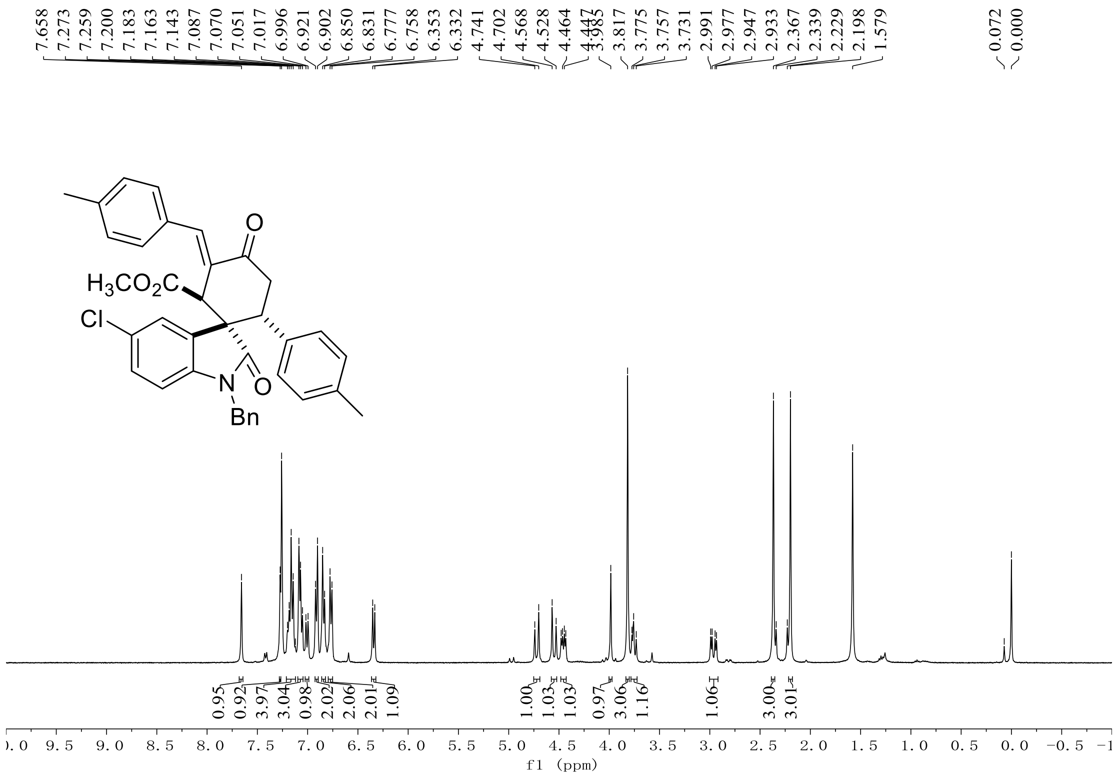

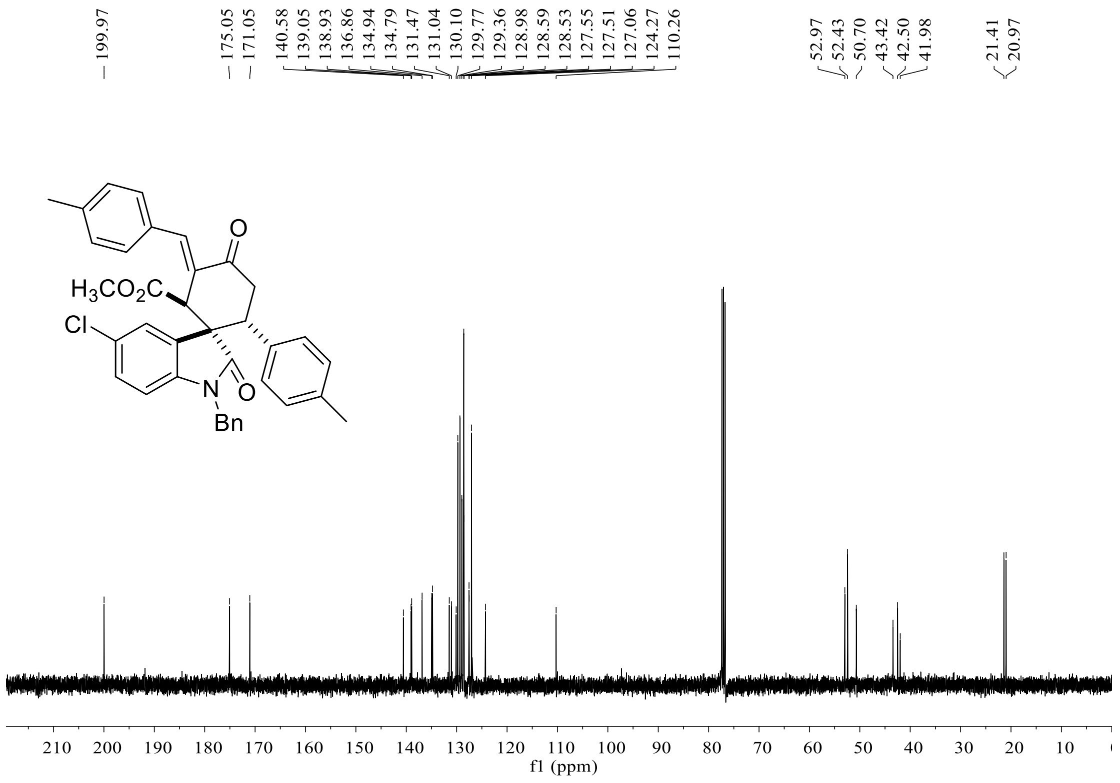
199.97
175.05
171.05
140.58
139.05
138.93
136.86
134.94
134.79
131.47
131.04
130.10
129.77
129.36
128.98
128.59
128.53
127.55
127.51
127.06
124.27
110.26
52.97
52.43
50.70
43.42
42.50
41.98
21.41
20.97
O
H3CO2C
Cl
N
O
Bn
210 200 190 180 170 160 150 140 130 120 110 100 90 80 70 60 50 40 30 20 10
f1 (ppm)

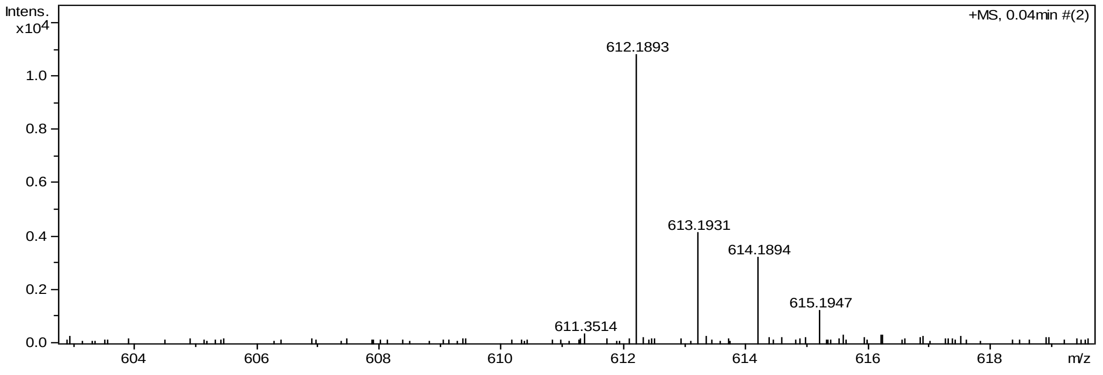
Intens.
x10^4
+MS, 0.04min #(2)
612.1893
613.1931
614.1894
615.1947
611.3514
1.0
0.8
0.6
0.4
0.2
0.0
604
606
608
610
612
614
616
618
m/z

***rel*-Methyl (1*S*,2*S*,6*R*)-1'-benzyl-5'-chloro-3-((*E*)-4-isopropylbenzylidene)-6-(4-isopropylphenyl)-2',4-dioxospiro[cyclohexane-1,3'-indoline]-2-carboxylate (5d)**: white solid, 58%, m.p. 226-228 °C; ^{1}H NMR (400 MHz, $CDCl_3$) δ 7.67 (s, 1H, ArH), 7.31 (d, *J* = 8.0 Hz, 2H, ArH), 7.21 (d, *J* = 8.0 Hz, 2H, ArH), 7.16 (t, *J* = 7.2 Hz, 1H, ArH), 7.09-7.05 (m, 3H, ArH), 7.01 (d, *J* = 8.4 Hz, 1H, ArH), 6.94 (d, *J* = 8.0 Hz, 2H, ArH), 6.89-6.84 (m, 4H, ArH), 6.35 (d, *J* = 8.0 Hz, 1H, CH), 4.74 (d, *J* = 15.6 Hz, 1H, CH_2), 4.54 (d, *J* = 15.6 Hz, 1H, CH_2), 4.47-4.42 (m, 1H, CH_2), 4.02 (s, 1H, CH), 3.82 (s, 3H, OCH_3), 3.80-3.72 (m, 1H, CH), 3.00-2.96 (m, 1H, CH_2), 2.94-2.88 (m, 1H, CH), 2.78-2.71 (m, 1H, CH), 1.25 (d, *J* = 6.8 Hz, 6H, CH_3), 1.14-1.12 (m, 6H, CH_3). ^{13}C NMR (100 MHz, $CDCl_3$) δ 199.8, 175.2, 171.1, 149.9, 147.7, 140.6, 139.0, 135.3, 134.9, 131.8, 130.8, 130.2, 130.0, 128.6, 128.5, 127.5, 127.5, 127.1, 126.8, 126.2, 124.3, 110.1, 52.9, 52.4, 50.7, 43.5, 42.6, 42.0, 33.9, 33.4, 23.8, 23.7. IR (KBr) υ: 3735, 3398, 2889, 2314, 1924, 1878, 1734, 1615, 1434, 1415, 1365, 1145, 1045, 904, 835, 745 cm^{-1}; MS (*m*/*z*): HRMS (ESI) Calcd. for $C_{41}H_{40}ClNaNO_4$ ($[M+Na]^+$): 668.2538, found: 668.2513.

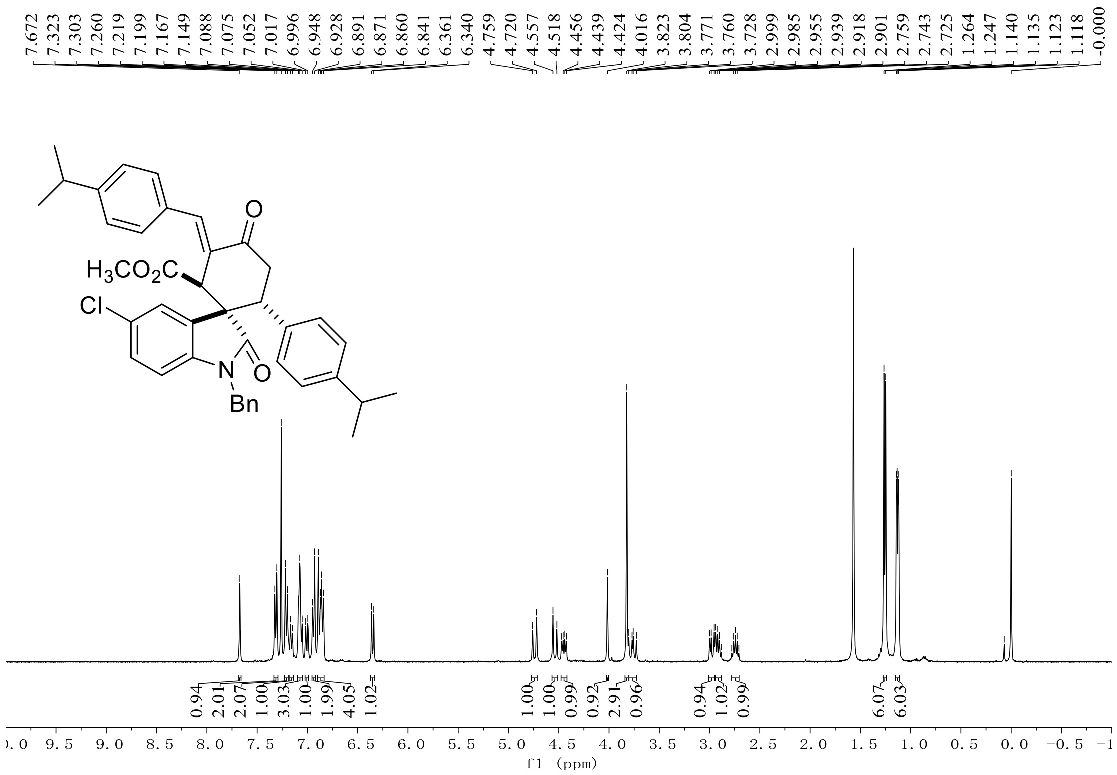

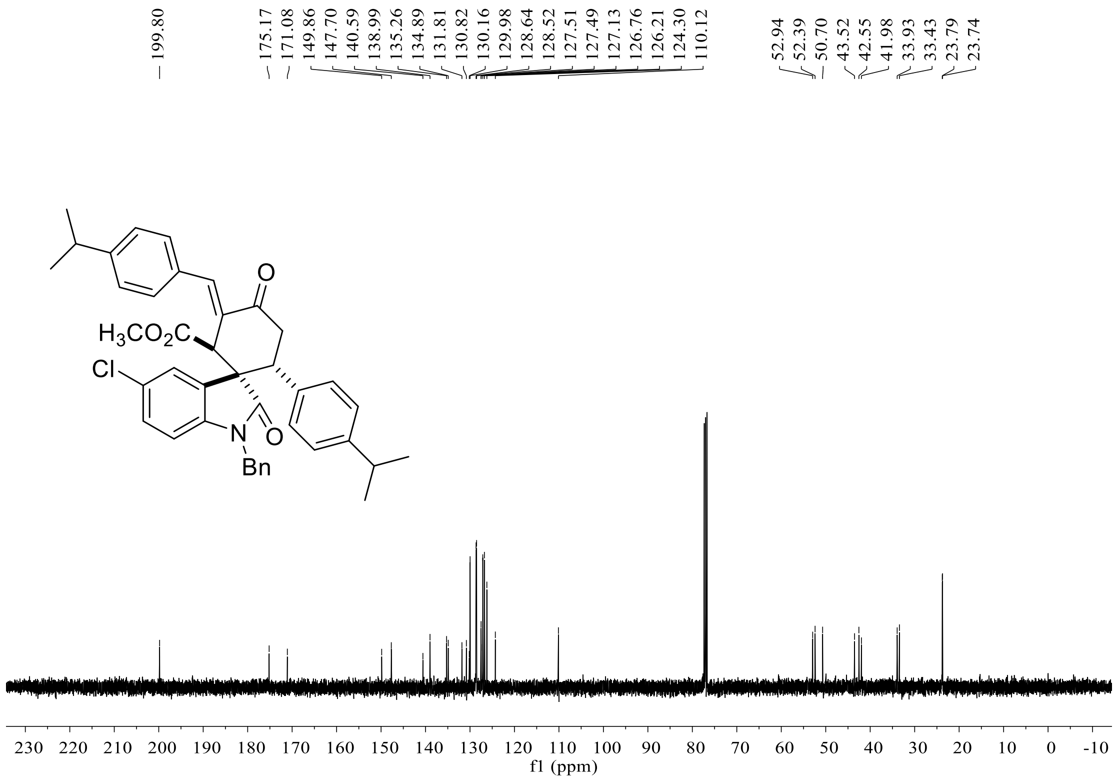
199.80
175.17
171.08
149.86
147.70
140.59
138.99
135.26
134.89
131.81
130.82
130.16
129.98
128.64
128.52
127.51
127.49
127.13
126.76
126.21
124.30
110.12
52.94
52.39
50.70
43.52
42.55
41.98
33.93
33.43
23.79
23.74
O
H_3CO_2C
Cl
N
O
Bn
230 220 210 200 190 180 170 160 150 140 130 120 110 100 90 80 70 60 50 40 30 20 10 0 -10
f1 (ppm)

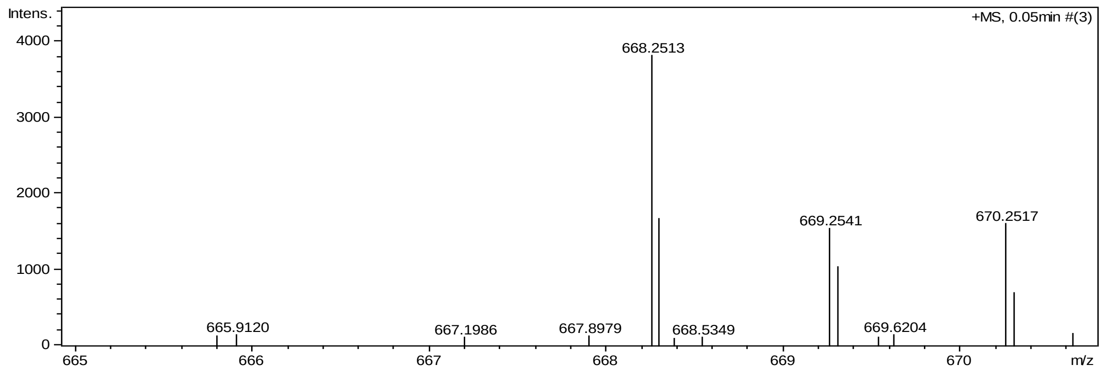
Intens.
+MS, 0.05min #(3)
4000
3000
2000
1000
0
668.2513
669.2541
670.2517
665.9120
667.1986
667.8979
668.5349
669.6204
665
666
667
668
669
670
m/z

***rel*-Ethyl (1*S*,2*S*,6*R*)-1'-benzyl-3-((*E*)-4-methylbenzylidene)-2',4-dioxo-6-(*p*-tolyl)spiro[cyclohexane-1,3'-indoline]-2-carboxylate (5e)**: white solid, 56%, m.p. 236-238 °C; **ratio of major/minor = 9:1,** ^{1}H NMR (400 MHz, $CDCl_3$) δ 7.65 (s, 1H, ArH), 7.29 (d, *J* = 7.6 Hz, 2H, ArH), 7.19-7.13 (m, 5H, ArH), 7.08 (d, *J* = 7.2 Hz, 1H, ArH), 7.03 (d, *J* = 8.0 Hz, 1H, ArH), 6.95 (d, *J* = 7.6 Hz, 1H, ArH), 6.90 (d, *J* = 7.6 Hz, 2H, ArH), 6.81-6.78 (m, 4H, ArH), 6.44 (d, *J* = 8.0 Hz, 1H, CH), 4.72 (d, *J* = 15.6 Hz, 1H, CH_2), 4.57 (d, *J* = 15.6 Hz, 1H, CH_2), 4.55-4.52 (m, 1H, CH_2), 4.39-4.31 (m, 1H, OCH_2), 4.23-4.16 (m, 1H, OCH_2), 3.96 (s, 1H, CH), 3.83-3.75 (1, 1H, CH), 2.99-2.93 (m, 1H, CH_2), 2.36 (s, 3H, CH_3), 2.18 (s, 3H, CH_3), 1.27 (t, *J* = 7.2 Hz, 3H, CH_3). ^{13}C NMR (100 MHz, $CDCl_3$) δ 200.4, 175.6, 170.7, 142.1, 138.9, 138.4, 136.6, 135.4, 135.3, 131.7, 131.7, 129.8, 129.3, 128.7, 128.6, 128.5, 127.4, 127.2, 124.0, 121.9, 109.3, 61.6, 52.8, 50.9, 43.3, 42.6, 42.1, 21.4, 21.0, 14.1. IR (KBr) υ: 3710, 2987, 2925, 1893, 1795, 1670, 1600, 1463, 1370, 1308, 1175, 1105, 1028, 900, 816, 744 cm^{-1}; MS (*m*/*z*): HRMS (ESI) Calcd. for $C_{38}H_{36}NO_4$ ($[M+H]^+$): 570.2639, found: 570.2644.

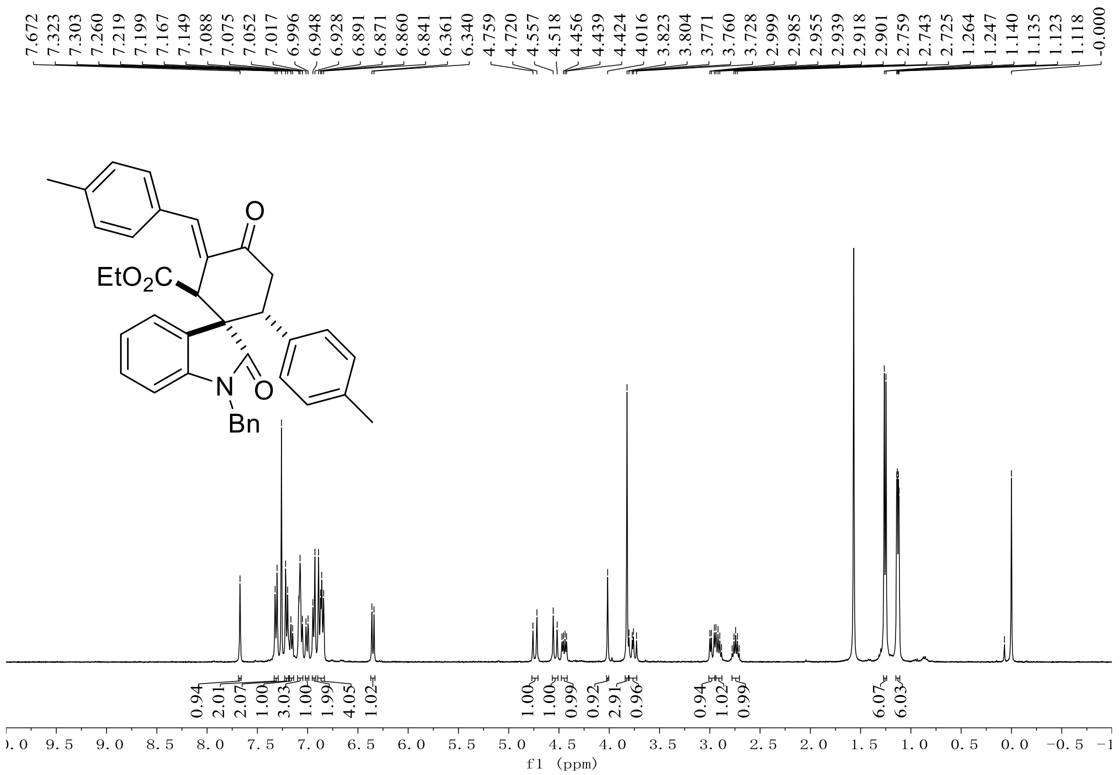

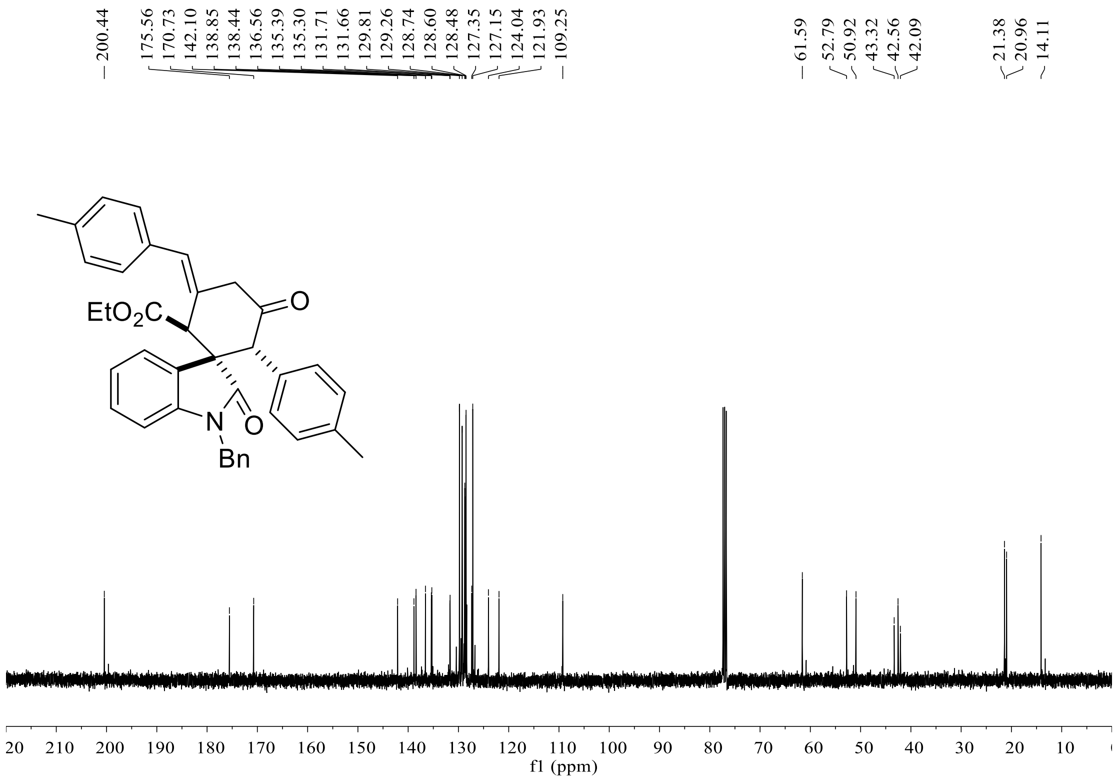
200.44
175.56
170.73
142.10
138.85
138.44
136.56
135.39
135.30
131.71
131.66
129.81
129.26
128.74
128.60
128.48
127.35
127.15
124.04
121.93
109.25
61.59
52.79
50.92
43.32
42.56
42.09
21.38
20.96
14.11
EtO2C
O
N
O
Bn
f1 (ppm)

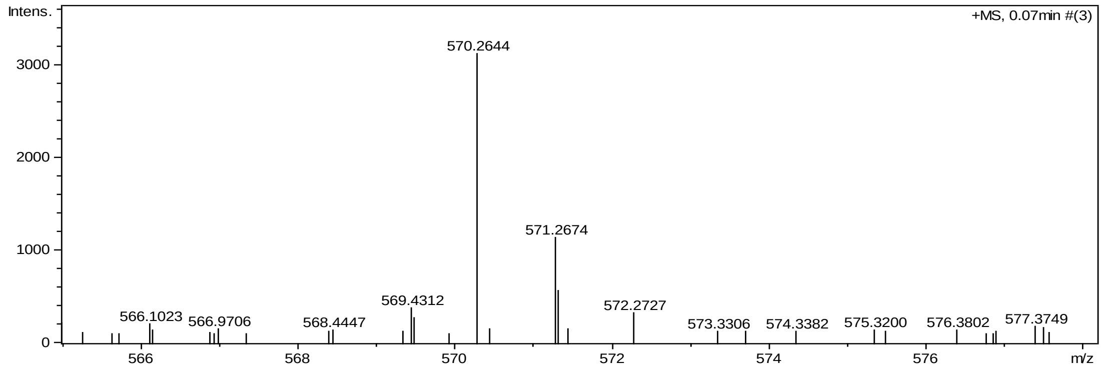
Intens.
+MS, 0.07min #(3)
570.2644
571.2674
566.1023
566.9706
568.4447
569.4312
572.2727
573.3306
574.3382
575.3200
576.3802
577.3749
m/z

***rel*-(3*R*,3'*R*)-1,1''-Dibenzyl-5''-chloro-5'-ethoxy-5-methyl-2,2''-dioxodispiro[indoline-3,2'-furan-3',3''-indoline]-4'-carbonitrile (8a)**: white solid, 71%, m.p. 175-177 °C; ^{1}H NMR (400 MHz, $CDCl_3$) δ 7.64 (s, 1H, ArH), 7.53 (s, 1H, ArH), 7.18-7.08 (m, 5H, ArH), 7.03 (t, *J* = 6.8 Hz, 3H, ArH), 6.71 (d, *J* = 7.6 Hz, 2H, ArH), 6.54 (d, *J* = 7.2 Hz, 2H, ArH), 6.39 (d, *J* = 8.0 Hz, 1H, ArH), 6.34 (d, *J* = 8.4 Hz, 1H, ArH), 5.17 (d, *J* = 16.4 Hz, 1H, CH_2), 5.07 (d, *J* = 16.0 Hz, 1H, CH_2), 4.65 (q, *J* = 7.2 Hz, 2H, CH_2), 4.35-4.30 (m, 2H, CH_2), 2.12 (s, 3H, CH_3), 1.54 (t, *J* = 7.2 Hz, 3H, CH_3). ^{13}C NMR (100 MHz, $CDCl_3$) δ 174.0, 173.1, 170.9, 142.4, 141.5, 134.2, 134.2, 133.6, 132.2, 130.4, 129.2, 128.8, 128.7, 128.6, 127.7, 127.6, 127.4, 126.4, 126.0, 124.0, 120.7, 114.2, 110.4, 109.6, 89.0, 69.1, 62.8, 60.1, 43.9, 20.9, 14.7. IR (KBr) υ: 3467, 3063, 3035, 2990, 2919, 2205, 1739, 1706, 1632, 1602, 1496, 1454, 1434, 1408, 1381, 1361, 1333, 1293, 1258, 1215, 1199, 1186, 1167, 1133, 1089, 1070, 1026, 997, 962, 933, 911, 895, 875, 836, 818, 776, 747 cm^{-1}; MS (*m/z*): HRMS (ESI) Calcd. for $C_{36}H_{28}NaClN_3O_4$ ($[M+Na]^+$): 624.1666, found: 624.1660.

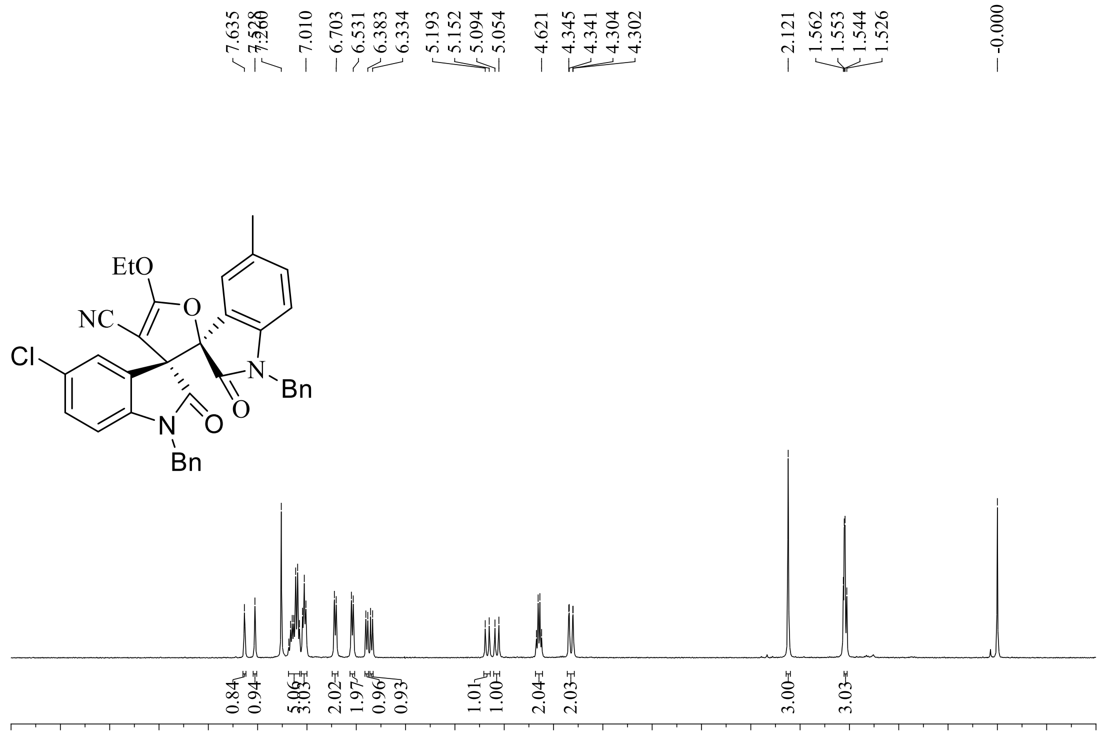

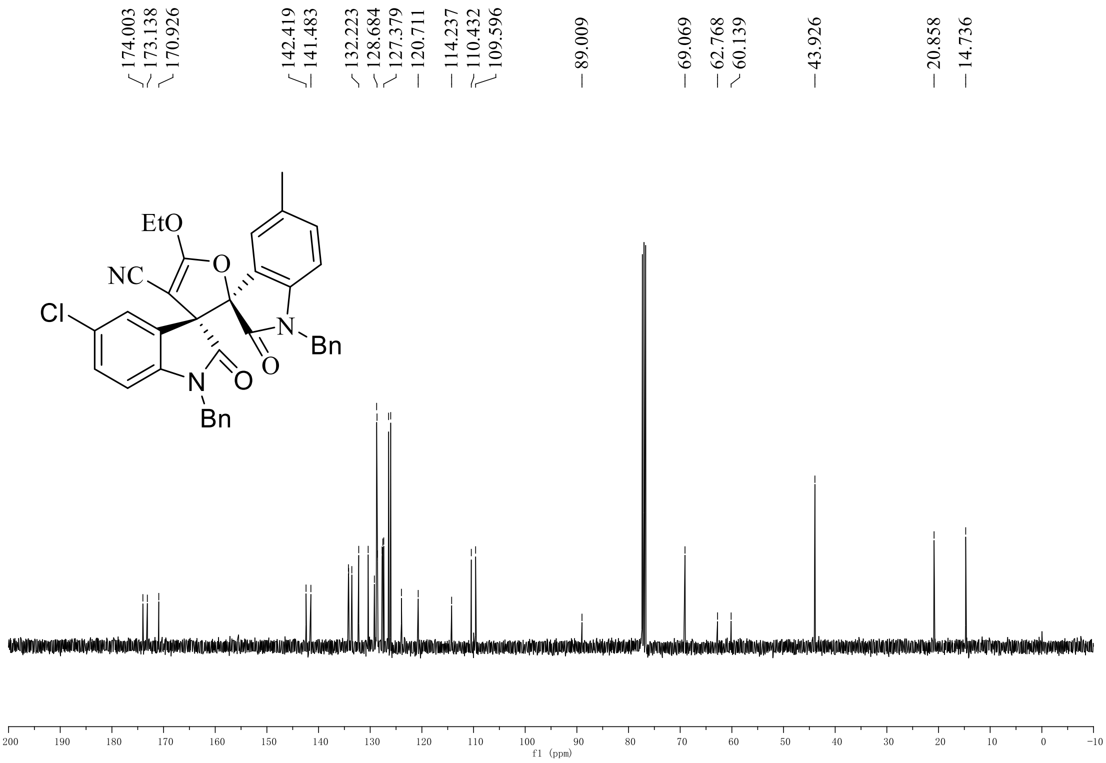
174.003
173.138
170.926
142.419
141.483
132.223
128.684
127.379
120.711
114.237
110.432
109.596
89.009
69.069
62.768
60.139
43.926
20.858
14.736
EtO
NC
O
Cl
N
Bn
O
N
O
Bn
f1 (ppm)

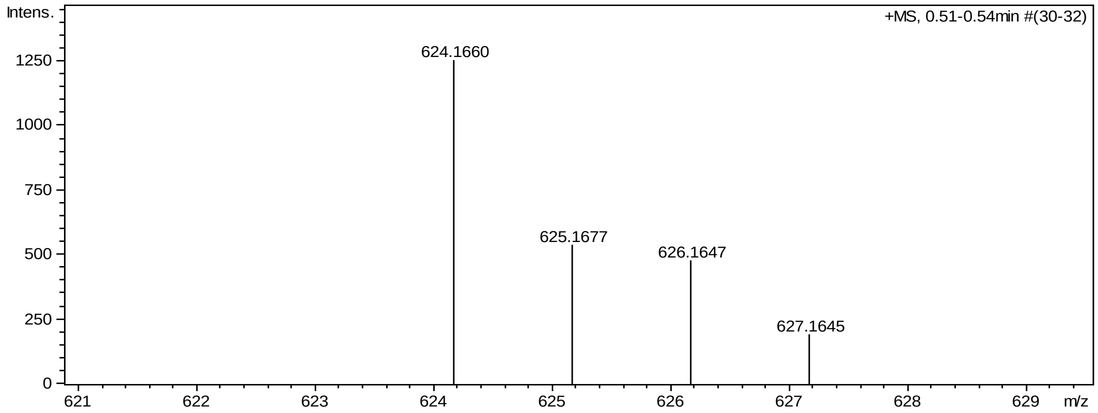
Intens.
+MS, 0.51-0.54min #(30-32)
624.1660
625.1677
626.1647
627.1645
m/z

***rel*-(3*R*,3'*R*)-1''-Benzyl-5''-chloro-5'-ethoxy-5-methyl-2,2''-dioxodispiro[indoline-3,2'-furan-3',3''-indoline]-4'-carbonitrile (8b)**: white solid, 70%, m.p. 140-142 °C; ^{1}H NMR (400 MHz, $CDCl_3$) δ 7.57 (d, *J* = 2.0 Hz, 1H, ArH), 7.51 (s, 1H, NH), 7.31 (s, 1H, ArH), 7.19-7.13 (m, 2H, ArH), 7.11-7.07 (m, 3H, ArH), 6.66 (d, *J* = 8.0 Hz, 1H, ArH), 6.57 (d, *J* = 7.2 Hz, 2H, ArH), 6.34 (d, *J* = 8.4 Hz, 1H, ArH), 5.21 (d, *J* = 16.0 Hz, 1H, CH_2), 4.62 (q, *J* = 7.2 Hz, 2H, CH_2), 4.30 (d, *J* = 16.4 Hz, 1H, CH_2), 2.15 (s, 3H, CH_3), 1.52 (t, *J* = 7.2 Hz, 3H, CH_3).^{13}C NMR (100 MHz, $CDCl_3$) δ 173.9, 173.2, 172.7, 142.3, 139.1, 134.2, 133.7, 132.4, 130.5, 128.9, 128.9, 128.7, 127.5, 127.1, 126.1, 123.6, 120.9, 114.2, 110.6, 110.6, 89.1, 69.1, 62.5, 60.1, 43.9, 20.9, 14.7. IR (KBr) υ: 3610, 3281, 3087, 3032, 3005, 2920, 2862, 2206, 1743, 1724, 1700, 1628, 1481, 1454, 1430, 1406, 1380, 1342, 1260, 1214, 1169, 1150, 1115, 1064, 1033, 999, 951, 928, 897, 824, 791, 765, 744, 722 cm^{-1}; MS (*m*/*z*): HRMS (ESI) Calcd. for $C_{29}H_{22}NaClN_3O_4$ ($[M+Na]^+$): 534.1197, found: 534.1190.

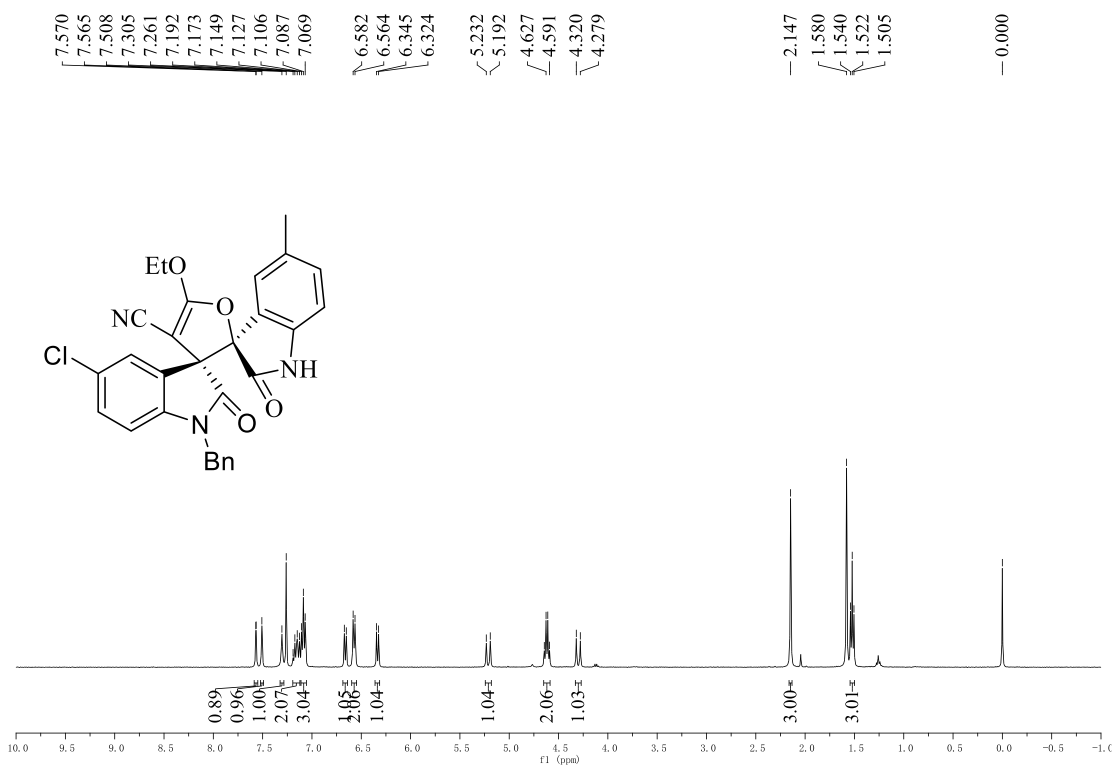

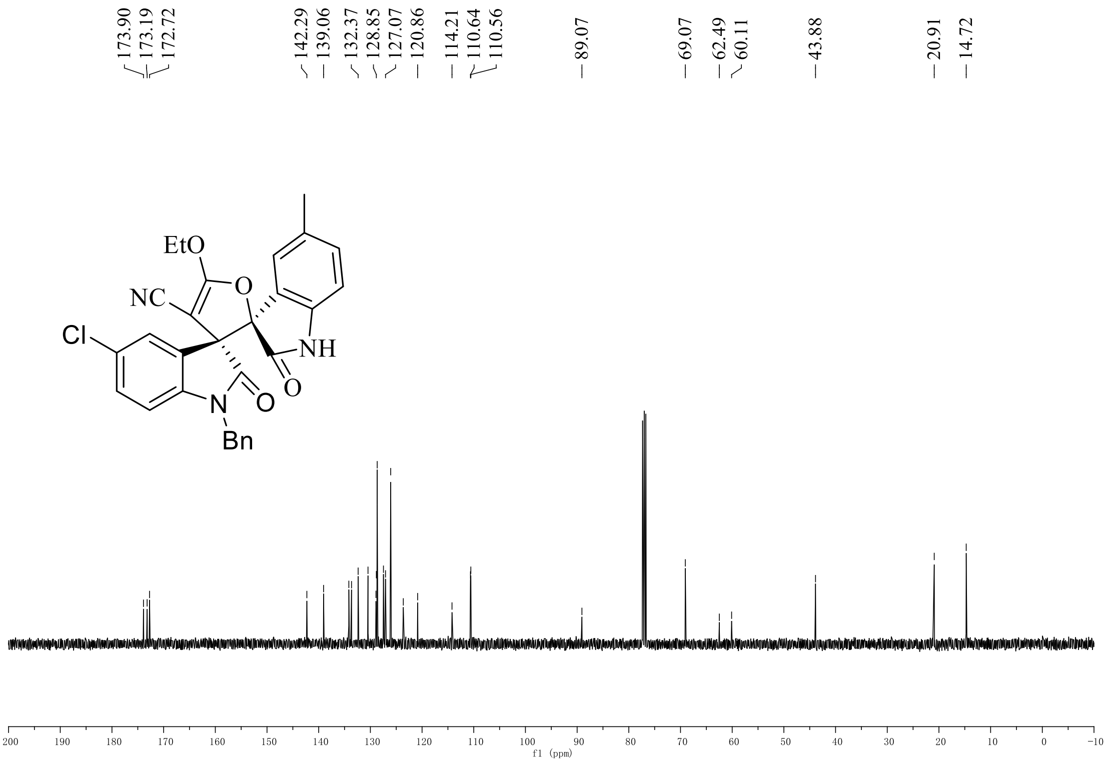
173.90
173.19
172.72
142.29
139.06
132.37
128.85
127.07
120.86
114.21
110.64
110.56
89.07
69.07
62.49
60.11
43.88
20.91
14.72
EtO
NC
O
Cl
NH
O
N
O
Bn
f1 (ppm)

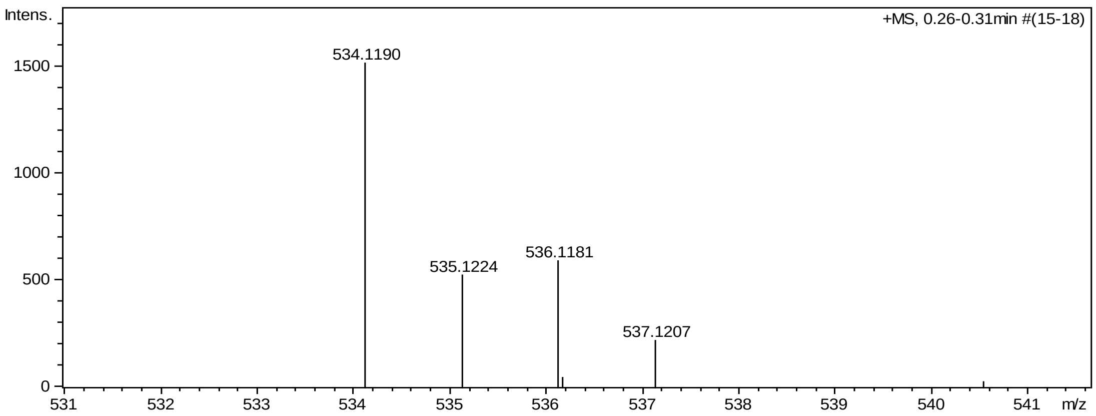
Intens.
+MS, 0.26-0.31min #(15-18)
534.1190
535.1224
536.1181
537.1207
m/z

rel-(3R,3'R)-1,1''-Dibenzyl-5,5''-dichloro-5'-ethoxy-2,2''-dioxodispiro[indoline-3,2'-furan-3',3''-indoline]-4'-carbonitrile (8c): lilac solid, 57%, m.p. 168-170 °C; ^{1}H NMR (400 MHz, $CDCl_3$) δ 7.75-7.74 (m, 1H, ArH), 7.62-7.61 (m, 1H, ArH), 7.19-7.08 (m, 8H, ArH), 6.70 (d, J = 7.2 Hz, 4H, ArH), 6.45-6.40 (m, 2H, ArH), 5.10 (t, J = 16.4 Hz, 2H, CH_2), 4.65 (q, J = 6.8 Hz, 2H, CH_2), 4.40 (d, J = 16.0 Hz, 1H, CH_2), 4.31 (d, J = 15.6 Hz, 1H, CH_2), 1.55 (t, J = 7.2 Hz, 3H, CH_3).^{13}C NMR (100 MHz, $CDCl_3$) δ 173.7, 172.9, 170.6, 142.4, 134.1, 133.7, 131.9, 130.6, 129.3, 129.3, 128.9, 128.8, 128.4, 127.9, 127.7, 127.6, 126.5, 126.4, 123.6, 122.4, 113.9, 110.8, 110.6, 110.0, 88.2, 69.3, 62.6, 60.4, 44.2, 44.1, 14.7. IR (KBr) υ: 3108, 3061, 3034, 2993, 2928, 2868, 2208, 1895, 1816, 1729, 1644, 1609, 1484, 1428, 1413, 1377, 1357, 1334, 1250, 1217, 1177, 1151, 1121, 1079, 995, 946, 903, 826, 752 cm^{-1}; MS (m/z): HRMS (ESI) Calcd. for $C_{35}H_{25}NaCl_2N_3O_4$ ([M+Na]$^+$): 644.1120, found: 644.1128.

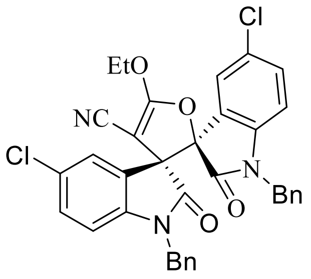

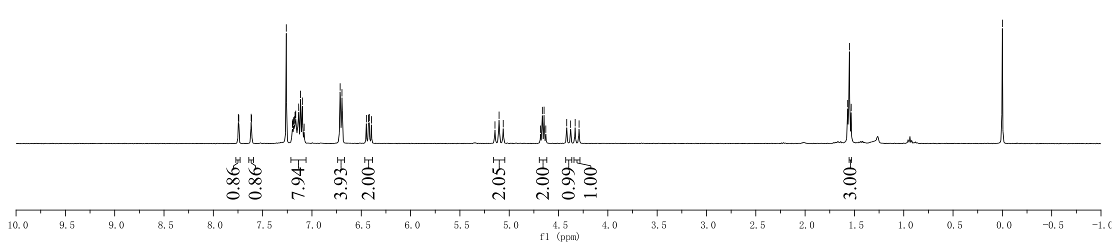

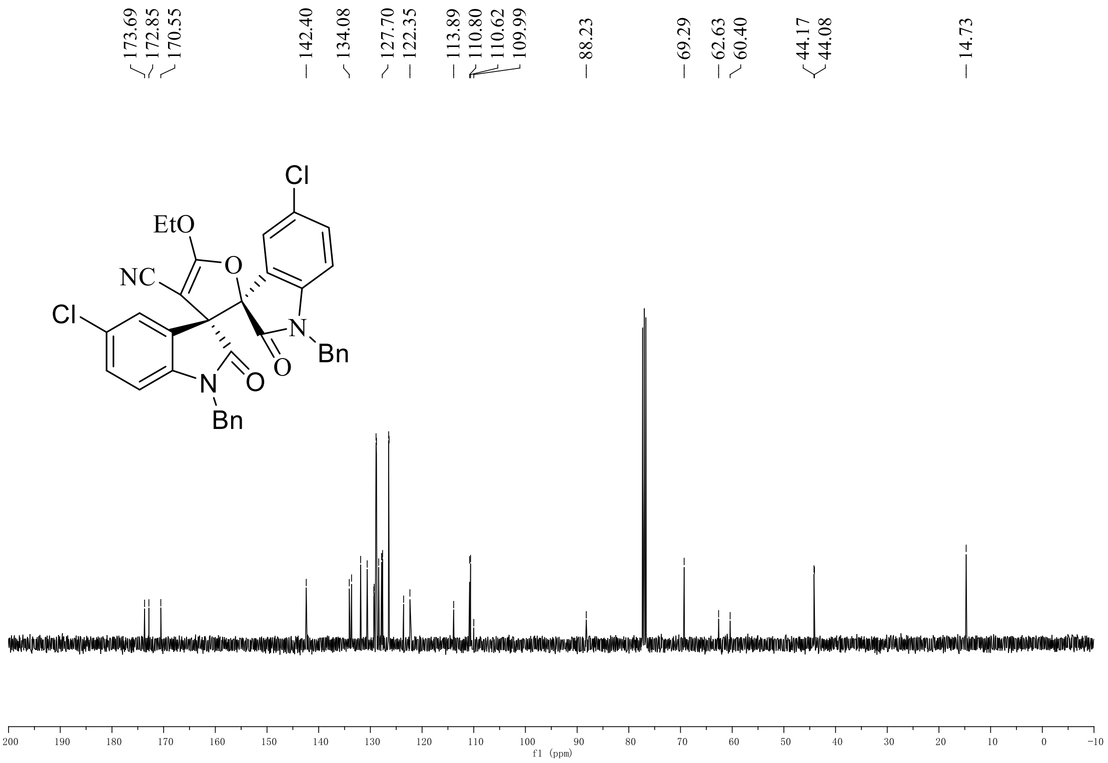
173.69
172.85
170.55
142.40
134.08
127.70
122.35
113.89
110.80
110.62
109.99
88.23
69.29
62.63
60.40
44.17
44.08
14.73
Cl
EtO
NC
O
Cl
N
Bn
O
N
O
Bn
f1 (ppm)

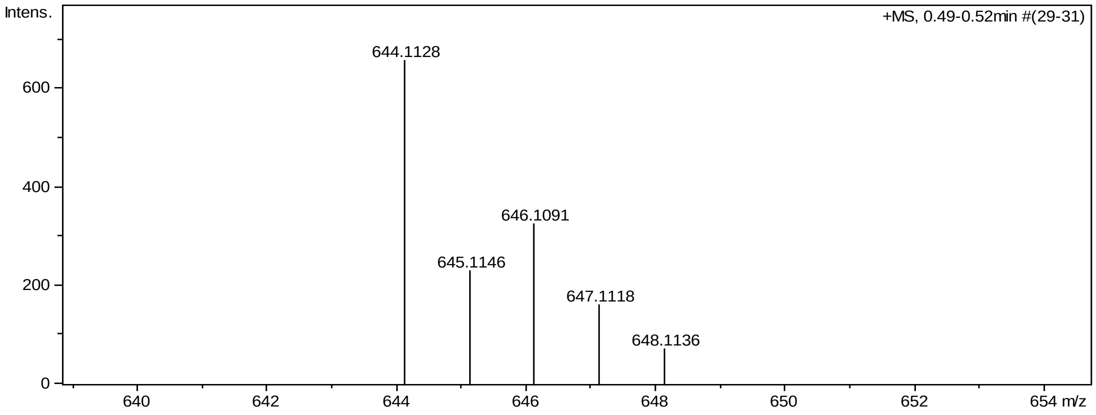
Intens.
+MS, 0.49-0.52min #(29-31)
644.1128
646.1091
645.1146
647.1118
648.1136
m/z

***rel*-(3*R*,3'*R*)-1,1''-Dibenzyl-5''-chloro-5'-ethoxy-5-fluoro-2,2''-dioxodispiro[indoline-3,2'-furan-3',3''-indoline]-4'-carbonitrile (8d)**: white solid, 56%, m.p. 137-139 °C; ^{1}H NMR (400 MHz, $CDCl_3$) δ 7.64 (s, 1H, ArH), 7.50 (d, *J* = 7.2 Hz, 1H, ArH), 7.20-7.06 (m, 7H, ArH), 6.90 (t, *J* = 7.6 Hz, 1H, ArH), 6.70-6.67 (m, 4H, ArH), 6.44-6.40 (m, 2H, ArH), 5.09 (t, *J* = 15.2 Hz, 2H, CH_2), 4.65 (q, *J* = 6.8 Hz, 2H, CH_2), 4.43 (d, *J* = 16.0 Hz, 1H, CH_2), 4.29 (d, *J* = 15.6 Hz, 1H, CH_2), 1.55 (t, *J* = 9.2 Hz, 3H, CH_3). ^{13}C NMR (100 MHz, $CDCl_3$) δ 173.7, 172.9, 170.8, 159.1 (d, *J* = 243.0 Hz), 142.4, 139.8, 134.0, 133.8, 130.6, 129.3, 128.9, 128.8, 127.8, 127.6, 126.5, 126.3, 123.7, 122.2 (d, *J* = 9.0 Hz), 118.4 (d, *J* = 24.0 Hz), 116.3 (d, *J* = 26.0 Hz), 113.9, 110.6, 110.5 (d, *J* = 8.0 Hz), 88.4, 69.3, 62.7, 60.3, 44.1, 14.7. IR (KBr) υ: 3062, 3036, 2993, 2929, 2868, 2209, 1883, 1728, 1643, 1489, 1453, 1412, 1378, 1336, 1273, 1217, 1178, 1152, 1117, 1078, 998, 954, 925, 899, 879, 846, 828, 785, 763, 748, 700 cm^{-1}; MS (*m*/*z*): HRMS (ESI) Calcd. for $C_{35}H_{25}NaClFN_3O_4$ ([M+Na]$^+$): 628.1415, found: 628.1409.

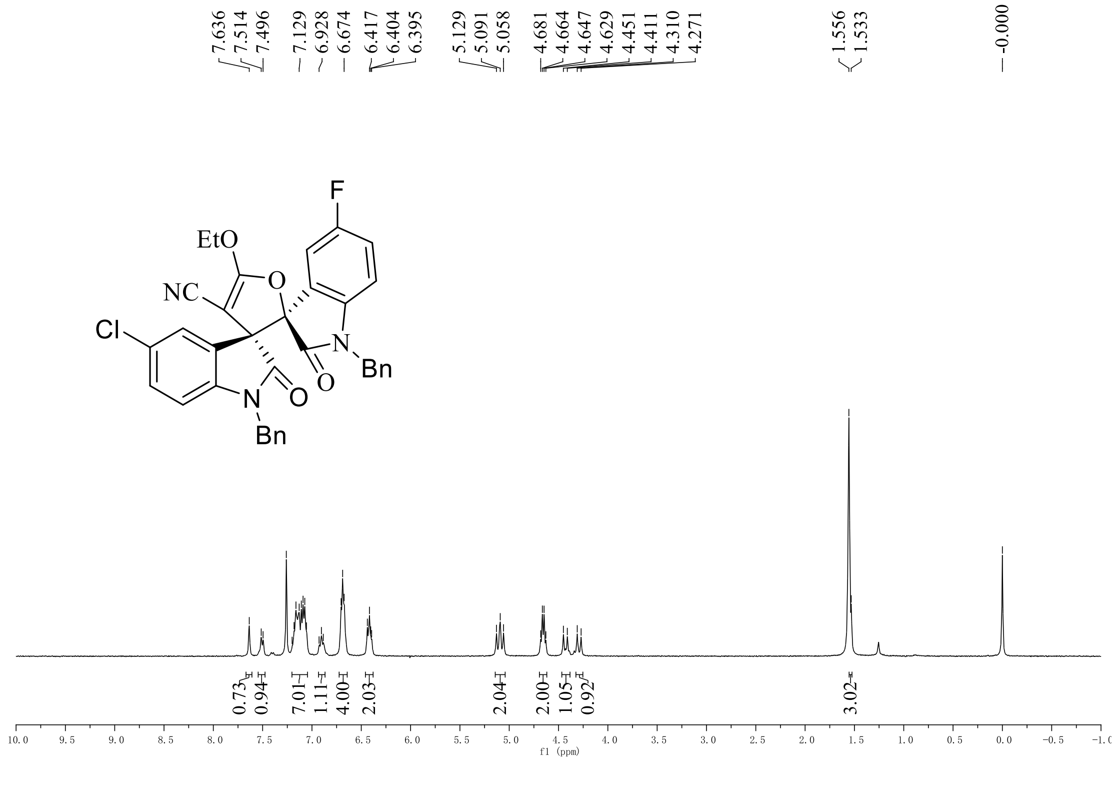

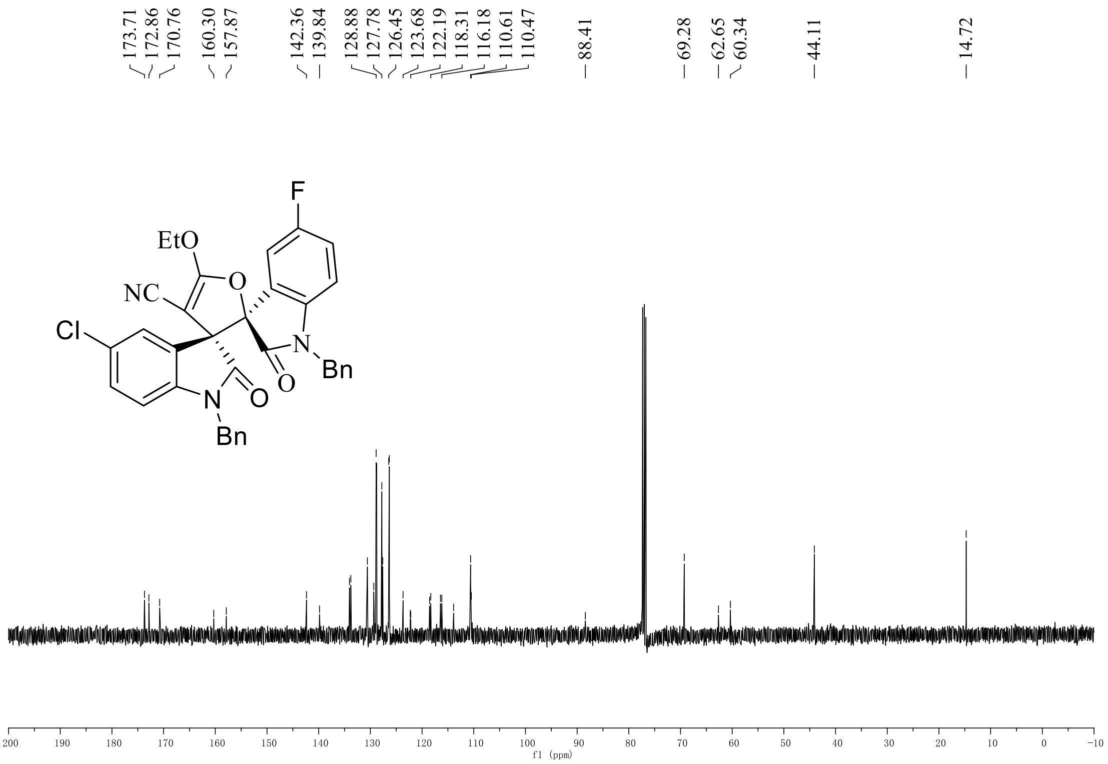
173.71
172.86
170.76
160.30
157.87
142.36
139.84
128.88
127.78
126.45
123.68
122.19
118.31
116.18
110.61
110.47
88.41
69.28
62.65
60.34
44.11
14.72
F
EtO
O
NC
Cl
N
Bn
O
O
N
Bn
f1 (ppm)

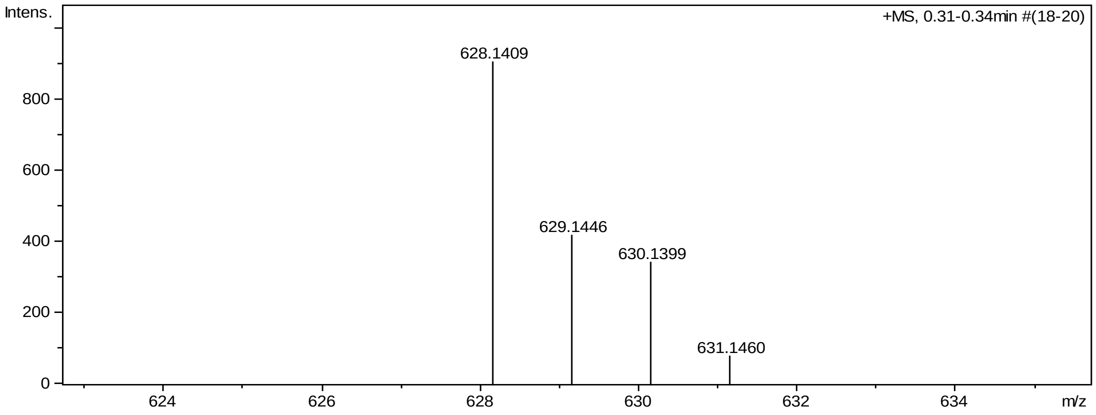
Intens.
+MS, 0.31-0.34min #(18-20)
628.1409
629.1446
630.1399
631.1460
m/z

***rel*-(3*R*,3'*R*)-1,1''-Dibenzyl-5''-chloro-5'-ethoxy-2,2''-dioxodispiro[indoline-3,2'-furan-3',3''-indoline]-4'-carbonitrile (8e)**: white solid, 54%, m.p. 170-172 °C; ^{1}H NMR (400 MHz, $CDCl_3$) δ 7.72 (d, *J* = 7.6 Hz, 1H, ArH), 7.63 (d, *J* = 1.2 Hz, 1H, ArH), 7.22 (d, *J* = 7.2 Hz, 1H, ArH), 7.18-7.06 (m, 5H, ArH), 7.02 (t, *J* = 7.6 Hz, 2H, ArH), 6.96 (t, *J* = 7.6 Hz, 1H, ArH), 6.69 (d, *J* = 7.6 Hz,, 2H, ArH), 6.55-6.50 (m, 3H, ArH), 6.33 (d, *J* = 8.0 Hz, 1H, ArH), 5.14-5.07 (m, 2H, CH_2), 4.65 (q, *J* = 7.2 Hz, 2H, CH_2), 4.36 (d, *J* = 16.4 Hz, 1H, CH_2), 4.31 (d, *J* = 15.6 Hz, 1H, CH_2), 1.55 (t, *J* = 4.8 Hz, 3H, CH_3).^{13}C NMR (100 MHz, $CDCl_3$) δ 173.9, 173.1, 171.0, 144.0, 142.3, 134.1, 134.0, 132.0, 130.4, 129.2, 128.8, 128.7, 128.0, 127.8, 127.6, 127.4, 126.4, 126.3, 124.0, 123.64, 120.8, 114.1, 110.6, 109.8, 88.9, 69.1, 62.8, 60.1, 44.0, 43.9, 14.7. IR (KBr) υ: 3063, 3034, 2986, 2936, 2207, 1727, 1641, 1611, 1484, 1467, 1429, 1412, 1378, 1336, 1251, 1213, 1178, 1150, 1112, 1079, 991, 942, 917, 904, 823, 769, 745 cm^{-1}; MS (*m*/*z*): HRMS (ESI) Calcd. for $C_{35}H_{26}NaClN_3O_4$ ([M+Na]$^+$): 610.1510, found: 610.1491.

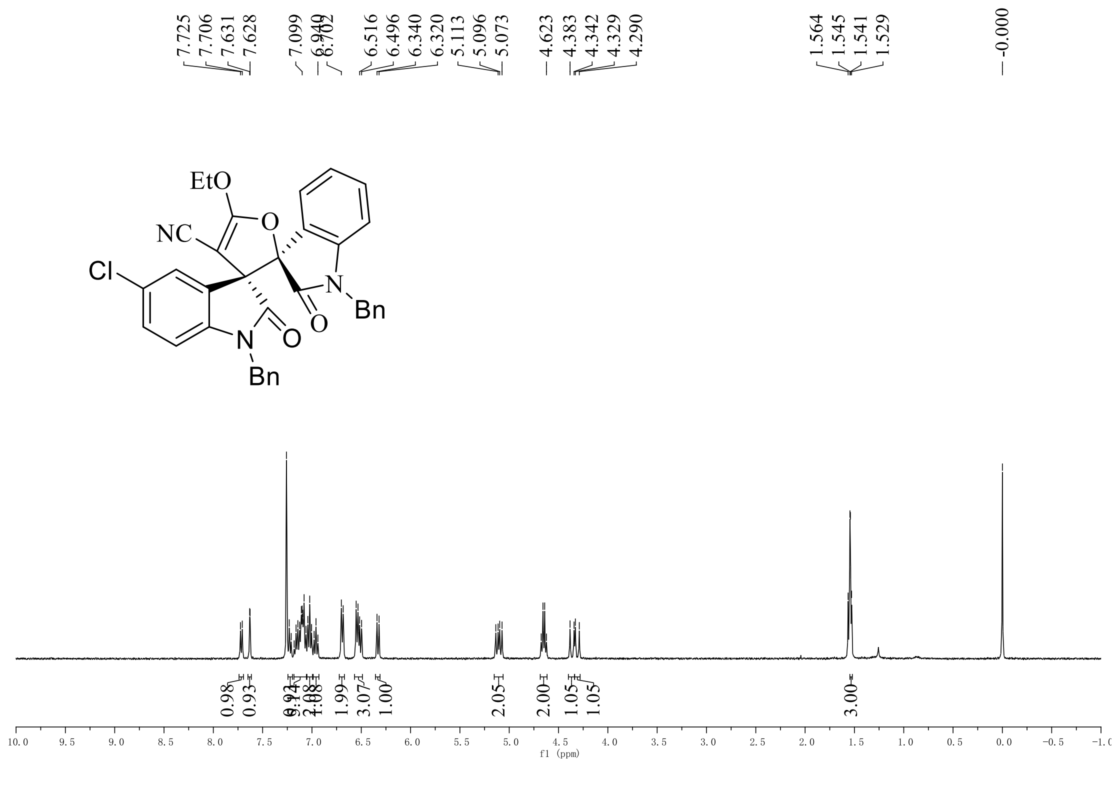

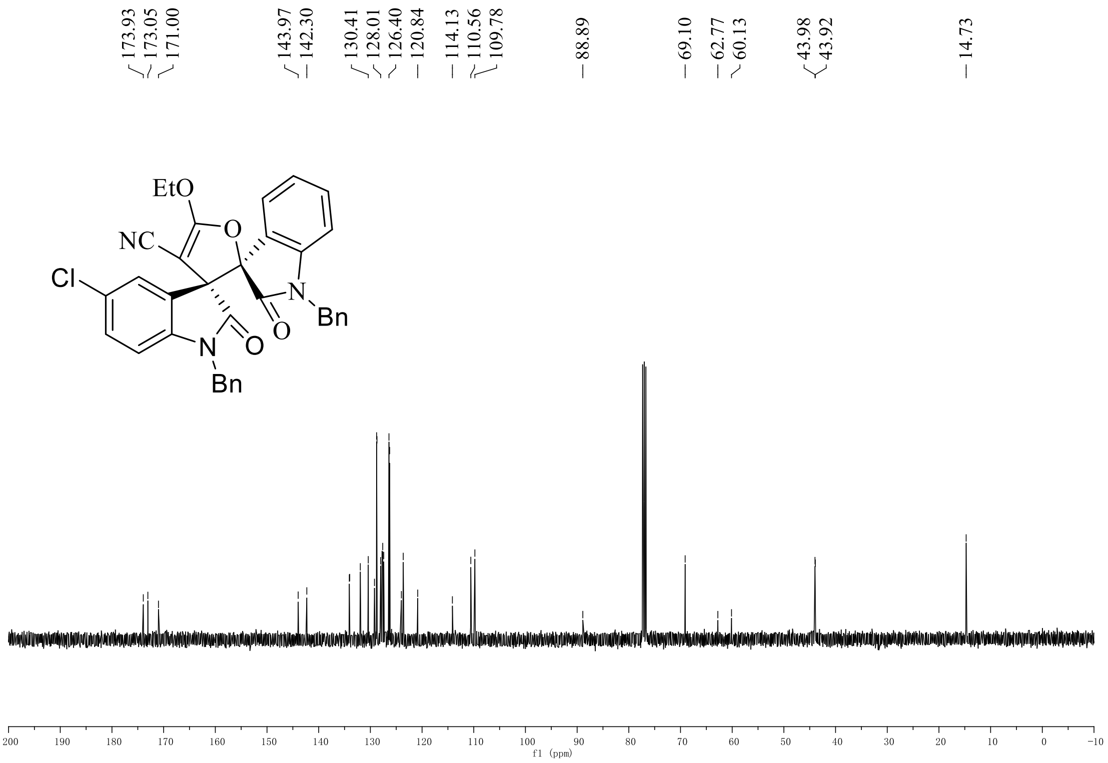
173.93
173.05
171.00
143.97
142.30
130.41
128.01
126.40
120.84
114.13
110.56
109.78
88.89
69.10
62.77
60.13
43.98
43.92
14.73
EtO
NC
O
Cl
N
Bn
O
O
N
Bn
f1 (ppm)

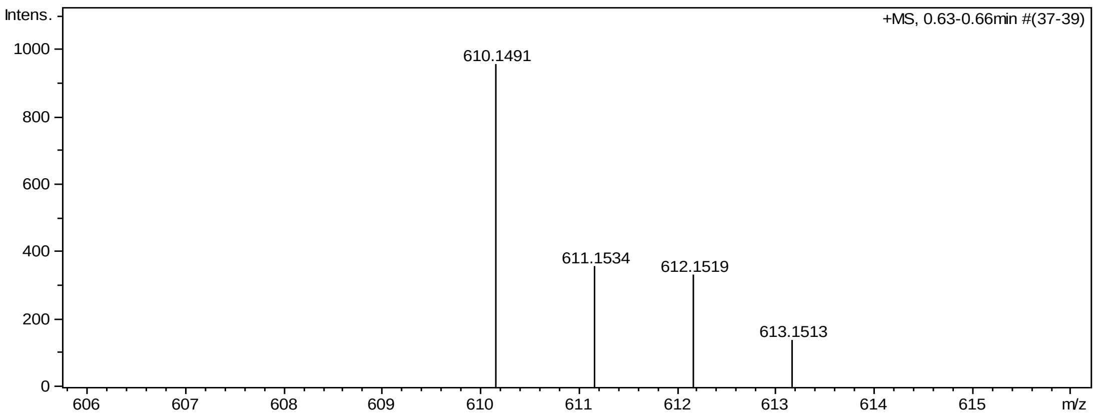
Intens.
+MS, 0.63-0.66min #(37-39)
610.1491
611.1534
612.1519
613.1513
m/z

***rel*-(3*R*,3'*R*)-1-Benzyl-1''-butyl-5''-chloro-5'-ethoxy-5-methyl-2,2''-dioxodispiro[indoline-3,2'-furan-3',3''-indoline]-4'-carbonitrile (8f)**: white solid, 76%, m.p. 142-144 °C; ^{1}H NMR (400 MHz, $CDCl_3$) δ 7.62 (s, 1H, ArH), 7.52 (s, 1H, ArH), 7.24 (s, 1H, ArH), 7.20-7.18 (m, 3H, ArH), 6.97 (d, *J* = 7.6 Hz, 1H, ArH), 6.77 (d, *J* = 6.4 Hz, 2H, ArH), 6.59 (d, *J* = 8.4 Hz, 1H, ArH), 6.33 (d, *J* = 8.0 Hz, 1H, ArH), 5.07 (d, *J* = 15.6 Hz,, 1H, CH_2), 4.62 (q, *J* = 6.8 Hz, 2H, CH_2), 4.27 (d, *J* = 15.6 Hz, 1H, CH_2), 3.76-3.69 (m, 1H, CH_2), 3.27-3.20 (m, 1H, CH_2), 2.27 (s, 3H, CH_3), 1.53 (t, *J* = 6.8 Hz, 3H, CH_3), 1.30-1.20 (m, 2H, CH_2), 1.04-0.97 (m, 2H, CH_2), 0.77 (t, *J* = 7.2 Hz, 3H, CH_3).^{13}C NMR (100 MHz, $CDCl_3$) δ 173.7, 173.1, 170.9, 142.9, 141.4, 134.4, 132.9, 132.2, 130.3, 128.8, 128.5, 127.7, 127.6, 126.6, 124.0, 120.4, 114.3, 109.4, 88.9, 69.0, 62.3, 60.1, 44.0, 40.2, 29.3, 21.0, 19.8, 14.7, 13.7. IR (KBr) υ: 3664, 3434, 3068, 3035, 2959, 2933, 2871, 2741, 2202, 1952, 1885, 1724, 1659, 1635, 1607, 1494, 1430, 1408, 1379, 1336, 1261, 1210, 1168, 1147, 1107, 1072, 997, 951, 928, 897, 846, 819, 729 cm^{-1}; MS (*m/z*): HRMS (ESI) Calcd. for $C_{33}H_{31}ClN_3O_4$ ($[M+H]^+$): 568.2003, found: 568.2000.

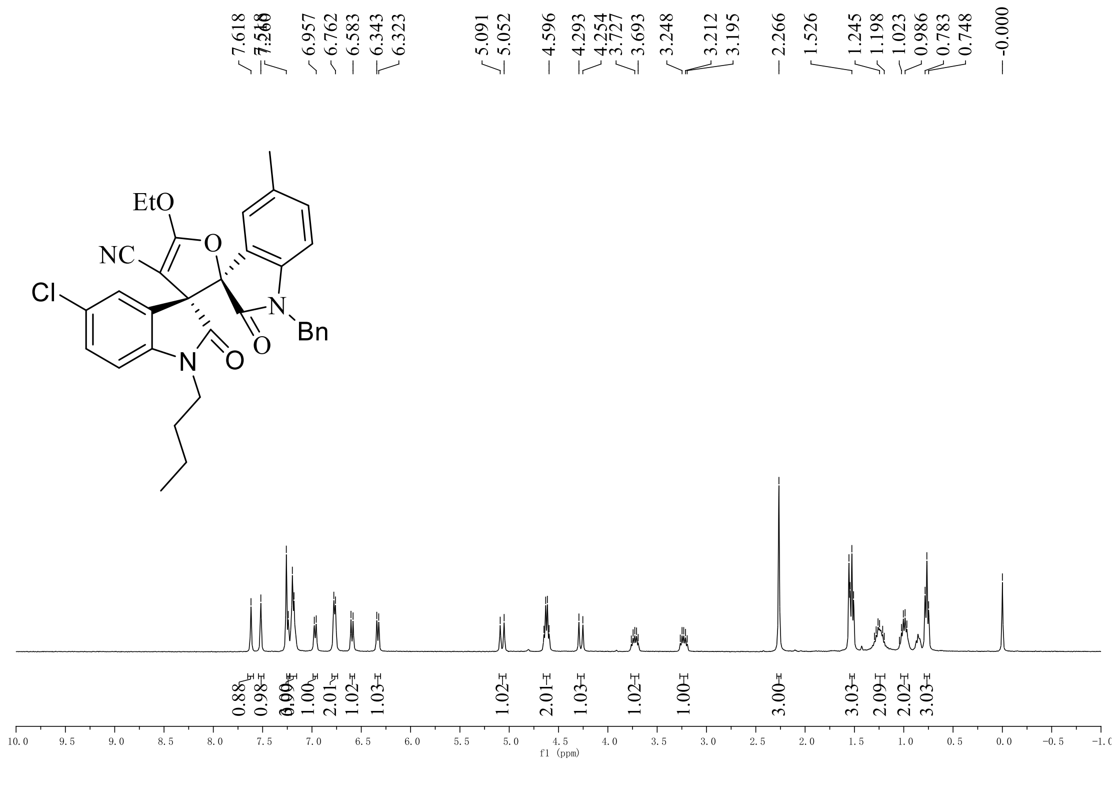

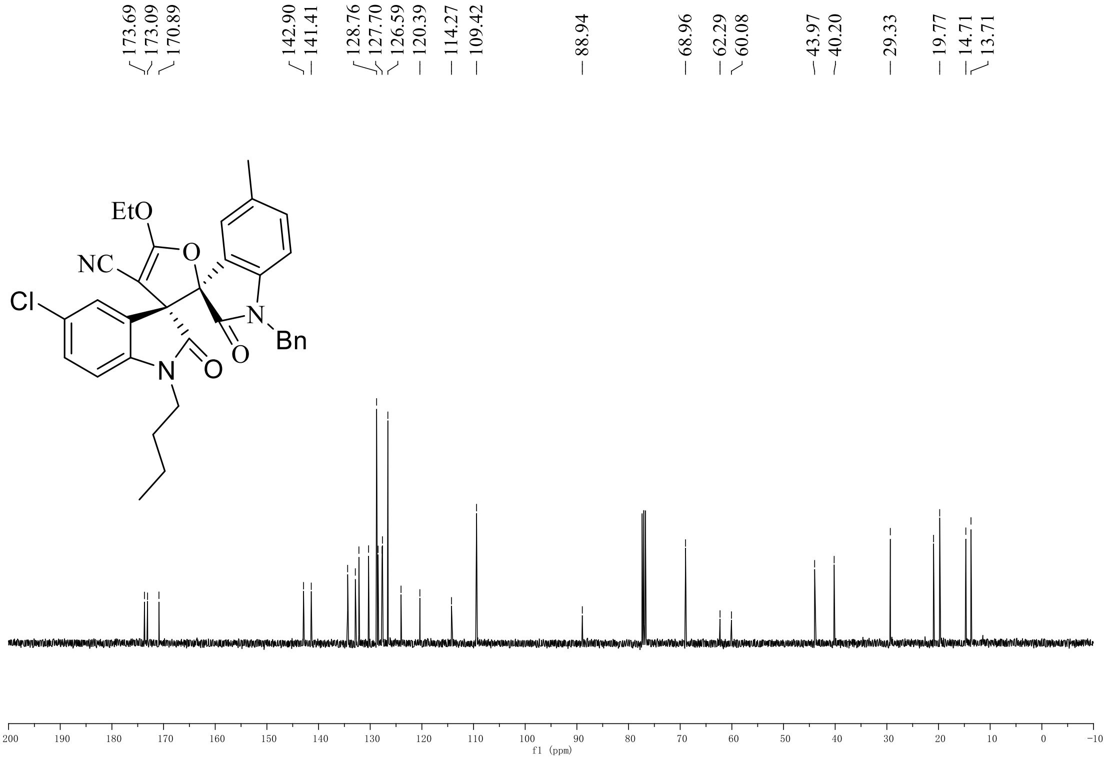
173.69
173.09
170.89
142.90
141.41
128.76
127.70
126.59
120.39
114.27
109.42
88.94
68.96
62.29
60.08
43.97
40.20
29.33
19.77
14.71
13.71
EtO
O
NC
Cl
N
Bn
O
N
O
f1 (ppm)

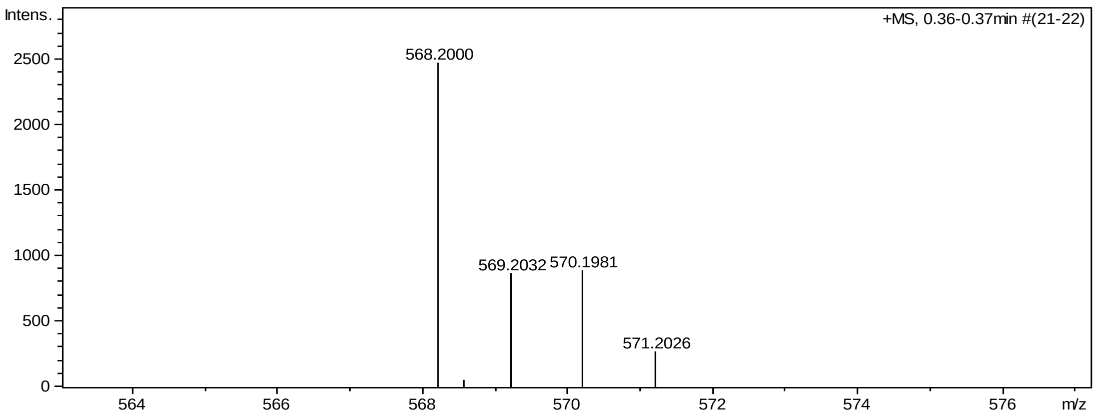
Intens.
+MS, 0.36-0.37min #(21-22)
568.2000
569.2032
570.1981
571.2026
m/z

***rel*-(3*R*,3'*R*)-1''-Butyl-5''-chloro-5'-ethoxy-5-methyl-2,2''-dioxodispiro[indoline-3,2'-furan-3',3''-indoline]-4'-carbonitrile (8g)**: white solid, 73%, m.p. 189-191 °C; ^{1}H NMR (400 MHz, $CDCl_3$) δ 7.98 (s, 1H, NH), 7.50 (d, *J* = 6.8 Hz, 2H, ArH), 7.18 (d, *J* = 7.6 Hz, 1H, ArH), 7.08 (d, *J* = 6.8 Hz, 1H, ArH), 6.66-6.58 (m, 2H, ArH), 4.59-4.57 (m, 2H, CH_2), 3.80-3.73 (m, 1H, CH_2), 3.26-3.19 (m, 1H, CH_2), 2.30 (s, 3H, CH_3), 1.49 (t, *J* = 6.4 Hz, 3H, CH_3), 1.31-1.25 (m, 2H, CH_2), 1.00-0.97 (m, 2H, CH_2), 0.80 (t, *J* = 6.4 Hz, 3H, CH_3).^{13}C NMR (100 MHz, $CDCl_3$) δ 173.6, 173.1, 172.7, 142.8, 138.9, 132.9, 132.3, 130.4, 128.8, 128.5, 127.1, 123.8, 120.6, 114.2, 110.4, 109.5, 89.0, 69.0, 62.1, 60.0, 40.2, 29.4, 21.0, 19.8, 14.7, 13.7. IR (KBr) υ: 3236, 3072, 2968, 2935, 2864, 2208, 1748, 1719, 1643, 1606, 1487, 1411, 1382, 1347, 1301, 1266, 1244, 1208, 1188, 1166, 1150, 1106, 1075, 1045, 1000, 951, 923, 900, 879, 844, 814, 794, 715 cm^{-1}; MS (*m/z*): HRMS (ESI) Calcd. for $C_{26}H_{24}NaClN_3O_4$ ($[M+Na]^+$): 500.1353, found: 500.1357.

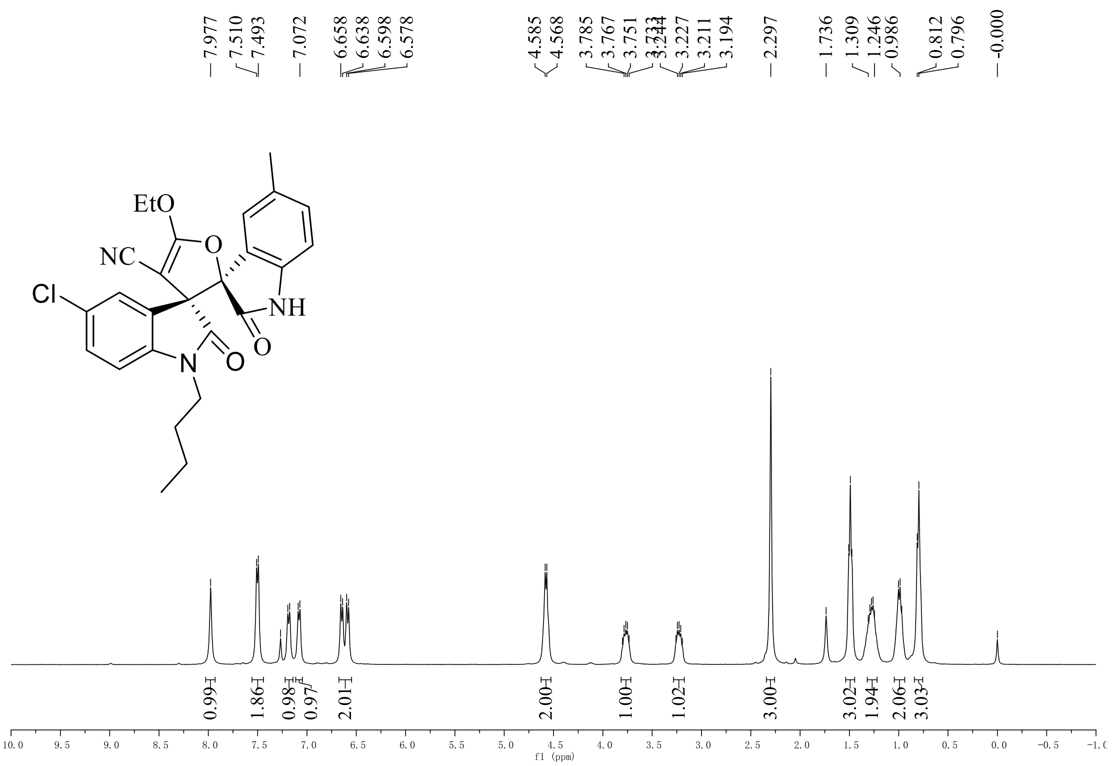

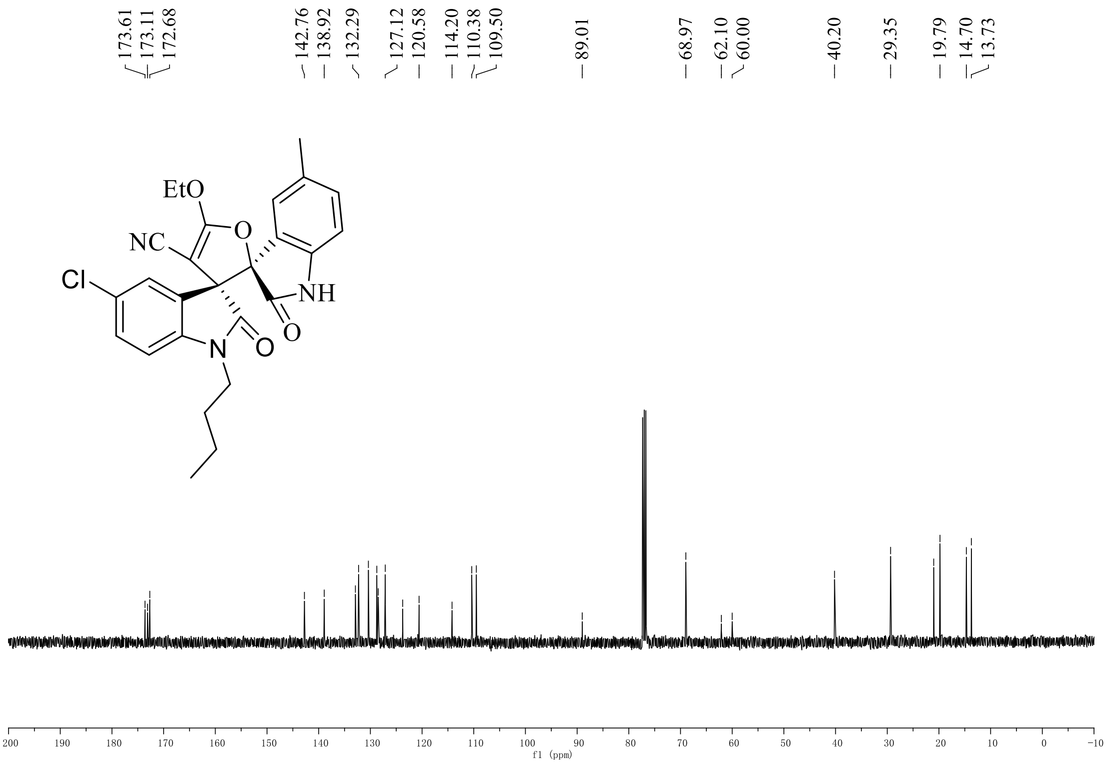
173.61
173.11
172.68
142.76
138.92
132.29
127.12
120.58
114.20
110.38
109.50
89.01
68.97
62.10
60.00
40.20
29.35
19.79
14.70
13.73
EtO
NC
O
Cl
NH
O
N
O
200 190 180 170 160 150 140 130 120 110 100 90 80 70 60 50 40 30 20 10 0 −10
f1 (ppm)

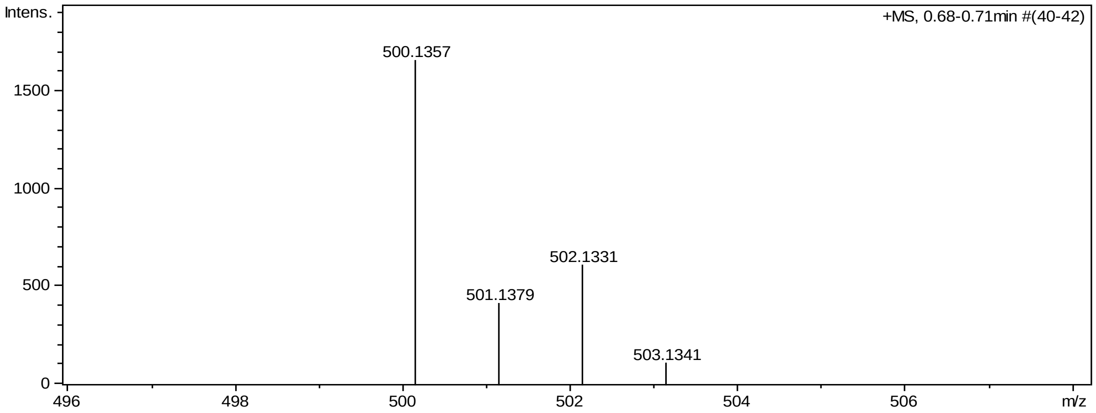
Intens.
+MS, 0.68-0.71min #(40-42)
500.1357
501.1379
502.1331
503.1341
1500
1000
500
0
496
498
500
502
504
506
m/z

***rel*-(3*R*,3'*R*)-1,1''-Dibenzyl-5'-ethoxy-5''-fluoro-5-methyl-2,2''-dioxodispiro[indoline-3,2'-furan-3',3''-indoline]-4'-carbonitrile (8h)**: white solid, 69%, m.p. 158-160 °C; ^{1}H NMR (400 MHz, $CDCl_3$) δ 7.55 (s, 1H, ArH), 7.40 (dd, J_1 = 8.4 Hz, J_2 = 2.4 Hz, 1H, ArH), 7.17-7.11 (m, 2H, ArH), 7.08-7.00 (m, 5H, ArH), 6.85-6.80 (m, 1H, ArH), 6.69 (d, *J* = 7.6 Hz, 2H, ArH), 6.54 (d, *J* = 7.2 Hz, 2H, ArH), 6.39 (d, *J* = 8.0 Hz, 1H, ArH), 6.36-6.33 (m, 1H, ArH), 5.20 (d, *J* = 16.4 Hz, 1H, CH_2), 5.04 (d, *J* = 16.0 Hz, 1H, CH_2), 4.65 (q, *J* = 7.2 Hz, 2H, CH_2), 4.33 (t, *J* = 15.6 Hz, 2H, CH_2), 2.13 (s, 3H, CH_3), 1.55 (t, *J* = 9.2 Hz, 3H, CH_3).^{13}C NMR (100 MHz, $CDCl_3$) δ 174.2, 173.1, 170.9, 159.4 (d, *J* = 242.0 Hz), 141.4, 139.8, 134.3, 134.2, 133.6, 132.2, 128.7, 128.6, 127.5, 127.3, 126.4, 126.1, 123.9 (d, *J* = 9.0 Hz), 120.8, 116.9 (d, *J* = 23.0 Hz), 115.6 (d, *J* = 26.0 Hz), 114.3, 110.1 (d, *J* = 8.0 Hz), 109.6, 89.0, 69.0, 63.0, 60.2, 44.0, 43.8, 20.9, 14.7. IR (KBr) υ: 3064, 3033, 2989, 2918, 2208, 1723, 1633, 1491, 1453, 1411, 1378, 1337, 1281, 1261, 1224, 1167, 1131, 1079, 1027, 996, 965, 928, 888, 846, 819.63, 773, 732, 702 cm^{-1}; MS (*m*/*z*): HRMS (ESI) Calcd. for $C_{36}H_{28}NaFN_3O_4$ ($[M+Na]^+$): 608.1962, found: 608.1960.

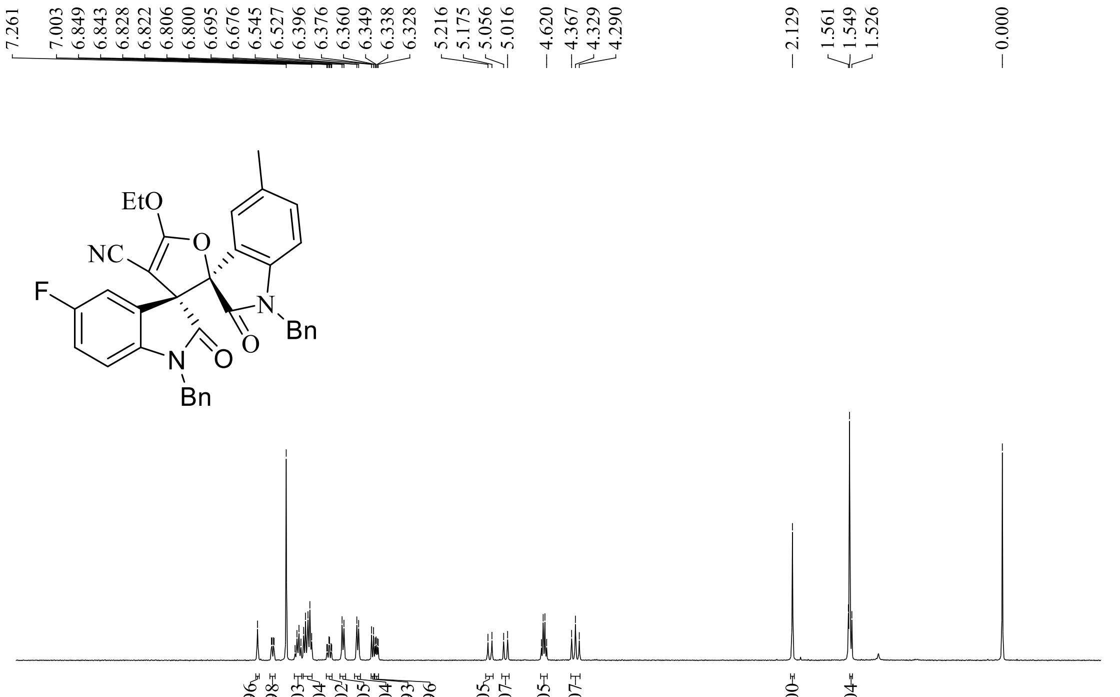

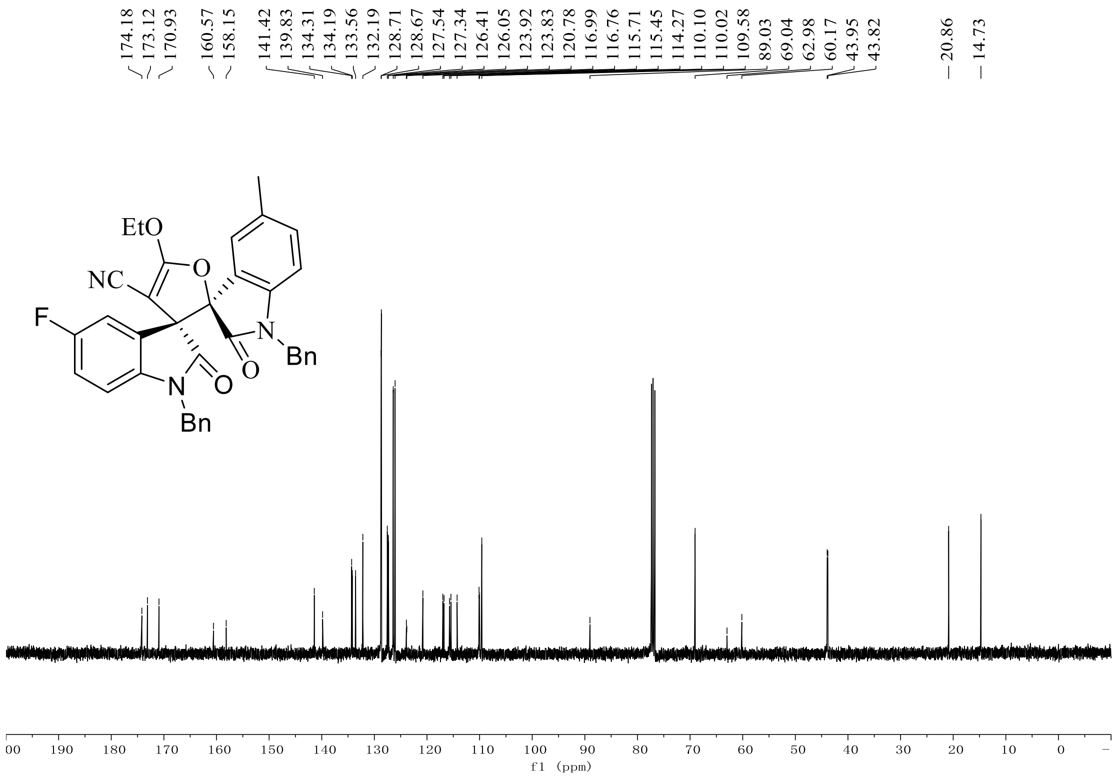
174.18
173.12
170.93
160.57
158.15
141.42
139.83
134.31
134.19
133.56
132.19
128.71
128.67
127.54
127.34
126.41
126.05
123.92
123.83
120.78
116.99
116.76
115.71
115.45
114.27
110.10
110.02
109.58
89.03
69.04
62.98
60.17
43.95
43.82
20.86
14.73
EtO
NC
O
F
N
Bn
O
N
O
Bn
f1 (ppm)

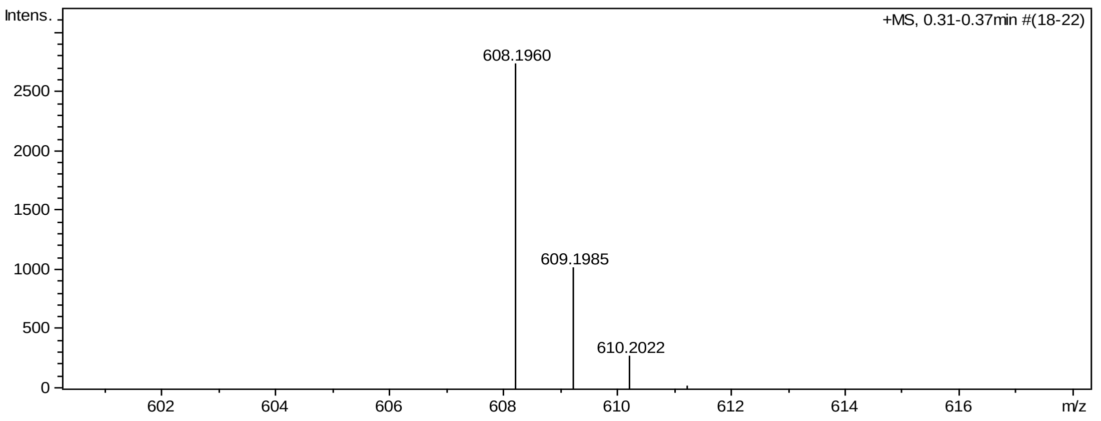
Intens.
+MS, 0.31-0.37min #(18-22)
608.1960
609.1985
610.2022
m/z

***rel*-(3*R*,3'*R*)-1,1''-Dibenzyl-5-chloro-5'-ethoxy-5''-fluoro-2,2''-dioxodispiro[indoline-3,2'-furan-3',3''-indoline]-4'-carbonitrile (8i)**: white solid, 47%, m.p. 150-152 °C; ^{1}H NMR (400 MHz, $CDCl_3$) δ 7.76 (d, *J* = 2.0 Hz, 1H, ArH), 7.38 (dd, J_1 = 8.4 Hz, J_2 = 2.4 Hz, 1H, ArH), 7.18-7.14 (m, 3H, ArH), 7.11-7.07 (m, 4H, ArH), 6.89-6.84 (m, 1H, ArH), 6.69 (t, *J* = 7.6 Hz, 4H, ArH), 6.45-6.42 (m, 1H, ArH), 6.40 (d, *J* = 8.8 Hz, 1H, ArH), 5.15 (d, *J* = 16.0 Hz, 1H, CH_2), 5.03 (d, *J* = 16.0 Hz, 1H, CH_2), 4.65 (q, *J* = 7.2 Hz, 2H, CH_2), 4.36 (t, *J* = 15.6 Hz, 2H, CH_2), 1.54 (t, *J* = 4.4 Hz, 3H, CH_3).^{13}C NMR (100 MHz, $CDCl_3$) δ 173.9, 172.8, 170.6, 160.6, 158.2, 141.1 (d, *J* = 250.0 Hz),139.8, 134.2, 133.6, 131.7, 129.3, 128.8, 128.5, 127.8, 127.6, 126.4, 126.3, 123.5 (d, *J* = 8.0 Hz), 122.4, 117.1 (d, *J* = 24.0 Hz), 115.6 (d, *J* = 26.0 Hz), 113.9, 110.8, 110.3 (d, *J* = 7.0 Hz), 88.3, 69.3, 62.9, 60.4, 44.2, 44.0, 14.7. IR (KBr) υ: 3082, 3032, 2980, 2936, 2361, 2206, 1885, 1724, 1637, 1612, 1487, 1452, 1437, 1408, 1379, 1334, 1263, 1222, 1178, 1130, 1078, 1029, 994, 963, 922, 902, 881, 841, 817, 746 cm^{-1}; MS (*m/z*): HRMS (ESI) Calcd. for $C_{35}H_{25}NaClFN_3O_4$ ([M+Na]$^+$): 628.1415, found: 628.1416.

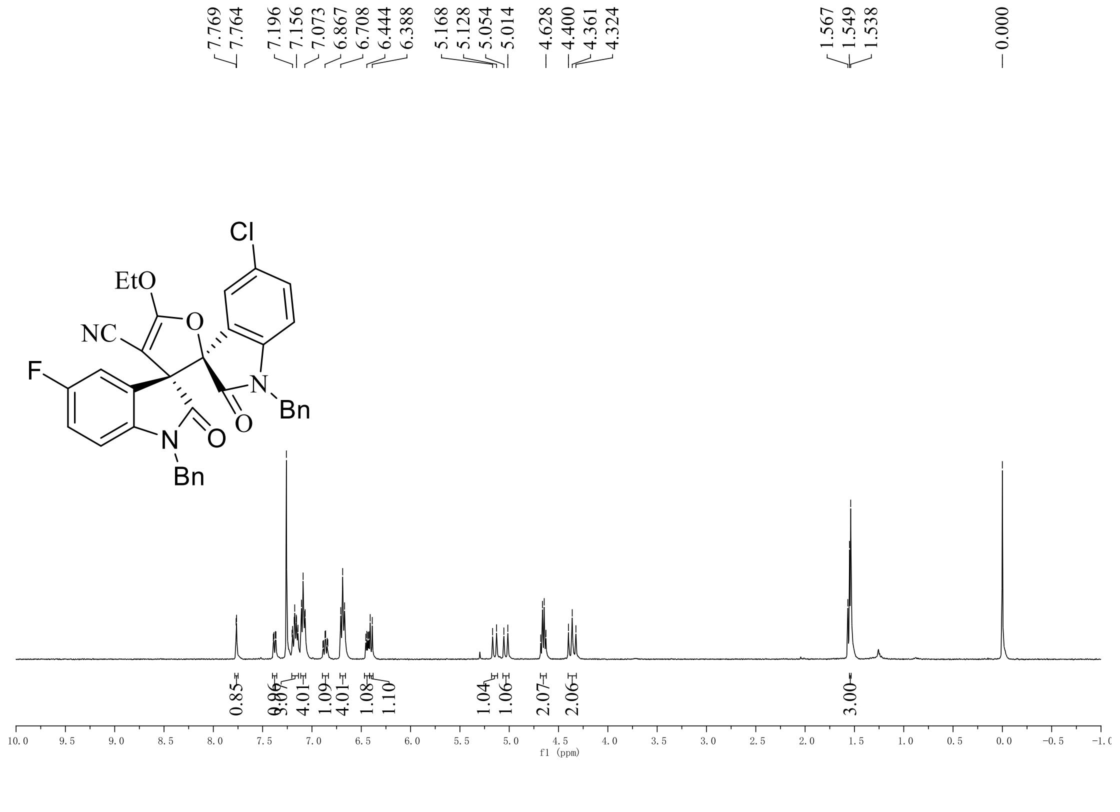

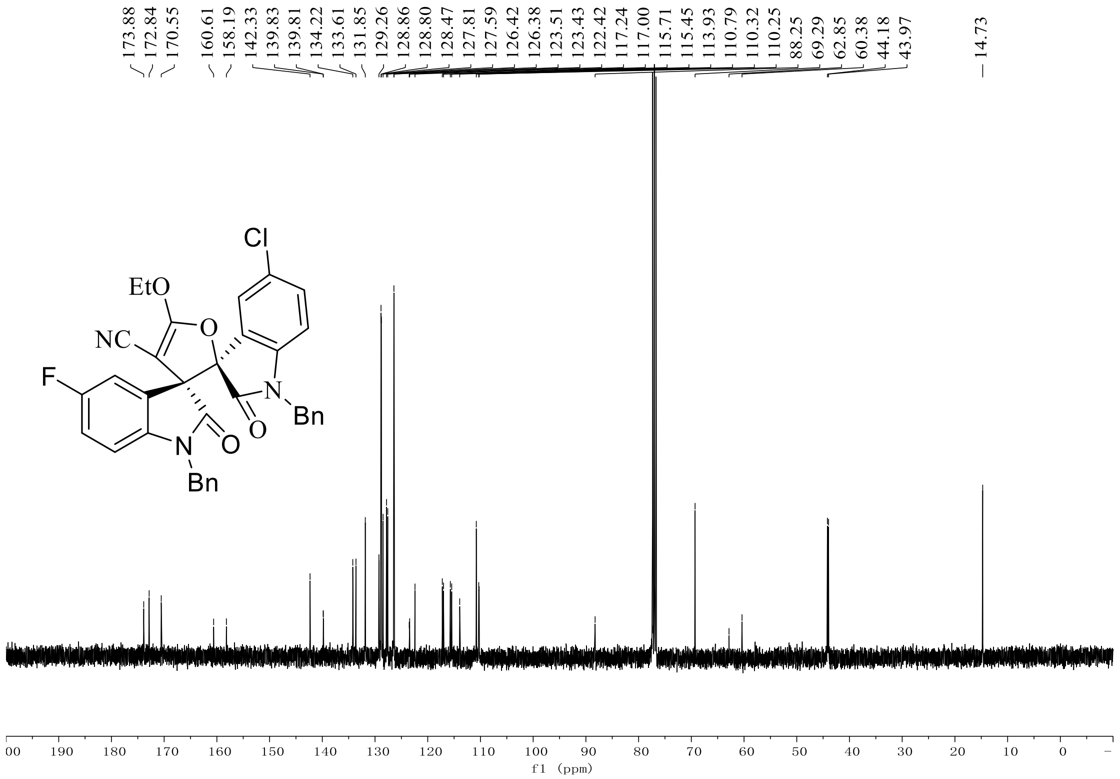
173.88
172.84
170.55
160.61
158.19
142.33
139.83
139.81
134.22
133.61
131.85
129.26
128.86
128.80
128.47
127.81
127.59
126.42
126.38
123.51
123.43
122.42
117.24
117.00
115.71
115.45
113.93
110.79
110.32
110.25
88.25
69.29
62.85
60.38
44.18
43.97
14.73
Cl
EtO
NC
O
F
N
Bn
O
N
O
Bn
f1 (ppm)

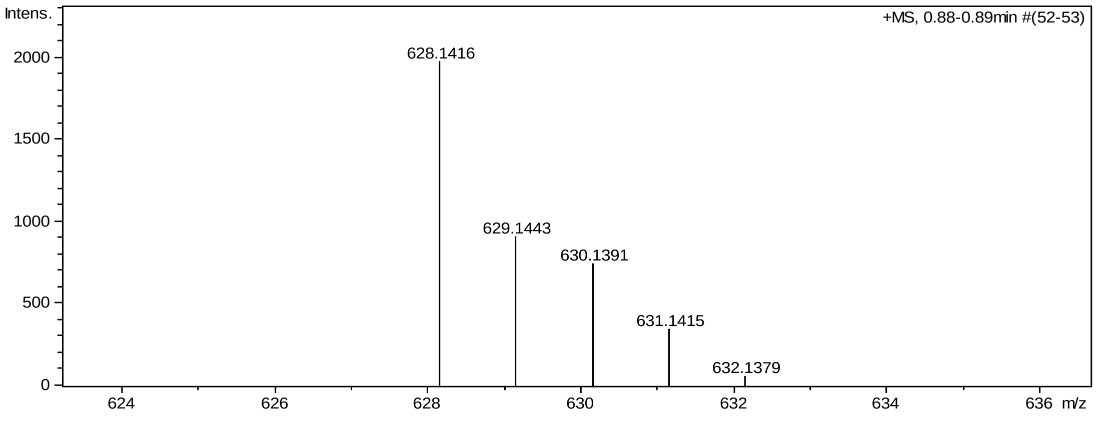
Intens.
+MS, 0.88-0.89min #(52-53)
628.1416
629.1443
630.1391
631.1415
632.1379
m/z

***rel*-(3*R*,3'*R*)-1,1''-Dibenzyl-5'-ethoxy-5,5''-difluoro-2,2''-dioxodispiro[indoline-3,2'-furan-3',3''-indoline]-4'-carbonitrile (8j)**: white solid, 35%, m.p. 168-170 °C; ^{1}H NMR (400 MHz, $CDCl_3$) δ 7.53 (dd, J_1 = 8.0 Hz, J_2 = 2.4 Hz, 1H, ArH), 7.40 (dd, J_1 = 8.4 Hz, J_2 = 2.8 Hz, 1H, ArH), 7.19-7.13 (m, 2H, ArH), 7.10-7.05 (m, 4H, ArH), 6.93-6.84 (m, 2H, ArH), 6.67 (d, *J* = 7.6 Hz, 4H, ArH), 6.44-6.38 (m, 2H, ArH), 5.09 (t, *J* = 16.0 Hz, 2H, CH_2), 4.65 (q, *J* = 6.8 Hz, 2H, CH_2), 4.41 (d, *J* = 16.0 Hz, 1H, CH_2), 4.32 (d, *J* = 16.0 Hz, 1H, CH_2), 1.55 (t, *J* = 8.0 Hz, 3H, CH_3).^{13}C NMR (100 MHz, $CDCl_3$) δ 173.9, 172.9, 170.8, 159.4 (d, *J* = 242.0 Hz), 159.1 (d, *J* = 242.0 Hz), 139.7, 134.2, 133.7, 128.8, 128.7, 127.8, 127.6, 126.4, 126.3, 123.6 (d, *J* = 9.0 Hz), 122.4 (d, *J* = 9.0 Hz), 118.4 (d, *J* = 24.0 Hz), 117.1 (d, *J* = 23.0 Hz), 116.4 (d, *J* = 26.0 Hz), 115.7 (d, *J* = 26.0 Hz), 114.0, 110.5 (d, *J* = 8.0 Hz), 110.3 (d, *J* = 8.0 Hz), 88.4, 69.3, 62.9, 60.3, 44.1, 44.0, 14.7. IR (KBr) υ: 3085, 3035, 2980, 2942, 2361, 2206, 1887, 1723, 1638, 1490, 1452, 1410, 1382, 1336, 1272, 1222, 1176, 1131, 1077, 1029, 997, 964, 930, 882, 846, 814, 776, 738, 701 cm^{-1}; MS (*m*/*z*): HRMS (ESI) Calcd. for $C_{35}H_{25}NaF_2N_3O_4$ ($[M+Na]^+$): 612.1711, found: 612.1698.

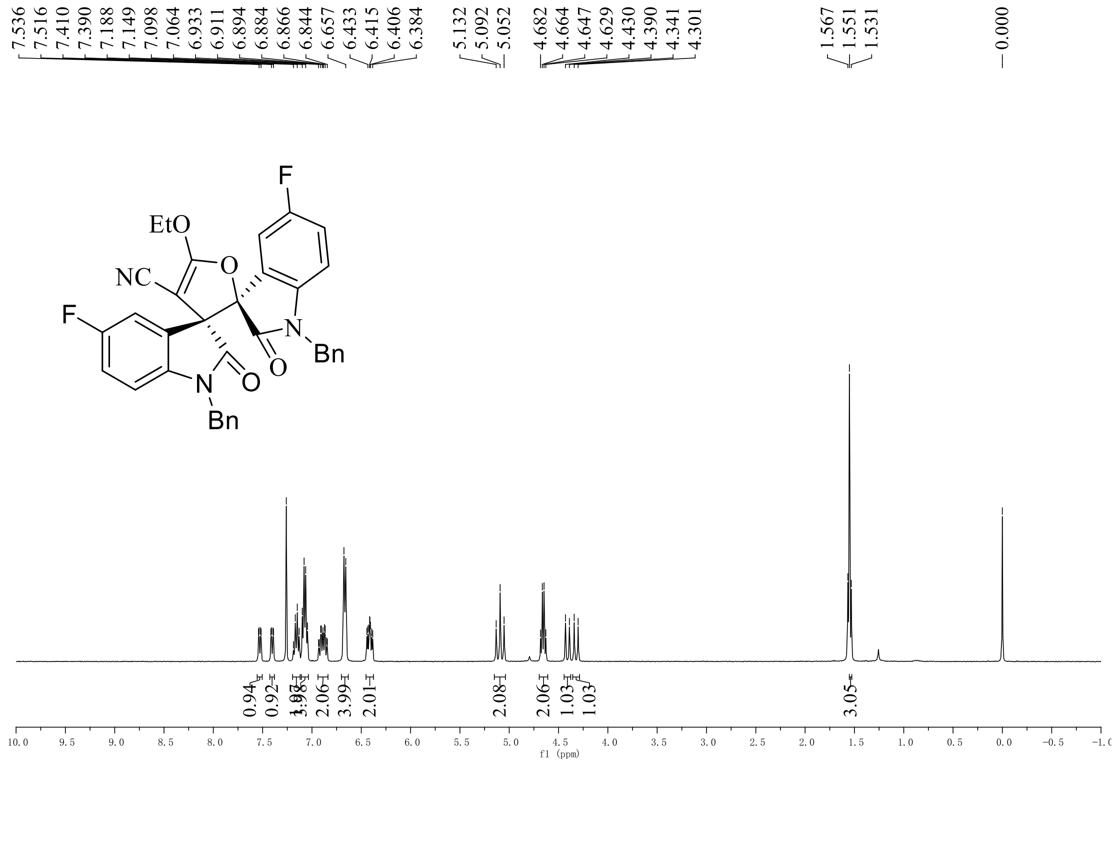

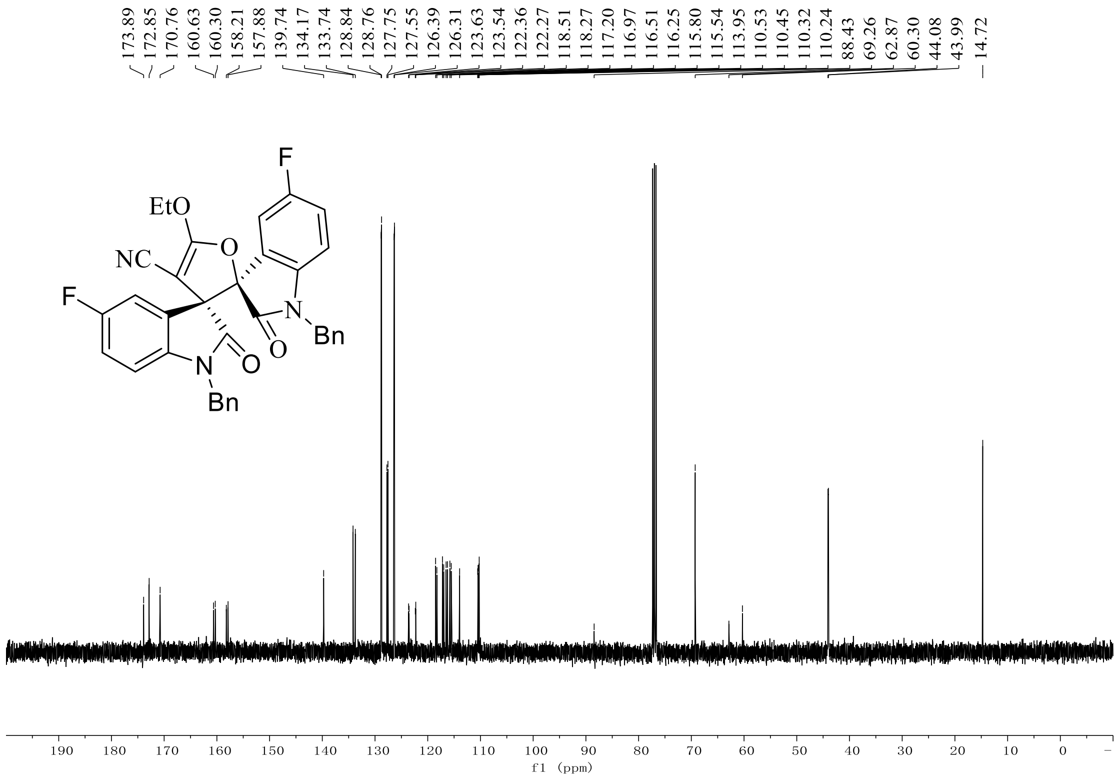
173.89
172.85
170.76
160.63
160.30
158.21
157.88
139.74
134.17
133.74
128.84
128.76
127.75
127.55
126.39
126.31
123.63
123.54
122.36
122.27
118.51
118.27
117.20
116.97
116.51
116.25
115.80
115.54
113.95
110.53
110.45
110.32
110.24
88.43
69.26
62.87
60.30
44.08
43.99
14.72
F
EtO
NC
O
N
Bn
O
F
N
O
Bn
190
180
170
160
150
140
130
120
110
100
90
80
70
60
50
40
30
20
10
0
f1 (ppm)

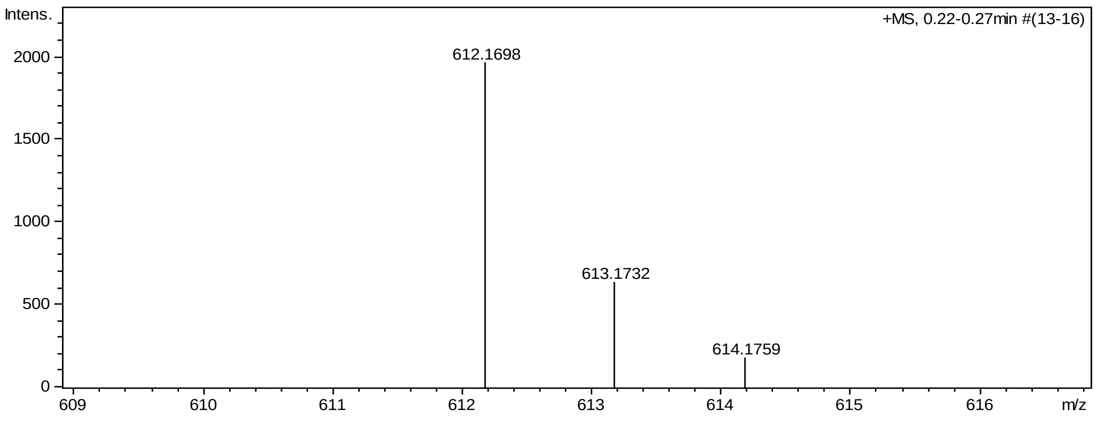
Intens.
+MS, 0.22-0.27min #(13-16)
612.1698
613.1732
614.1759
2000
1500
1000
500
0
609
610
611
612
613
614
615
616
m/z

***rel*-(3*R*,3'*R*)-1-Benzyl-5'-ethoxy-1'',5,5''-trimethyl-2,2''-dioxodispiro[indoline-3,2'-furan-3',3''-indoline]-4'-carbonitrile (8k)**: white solid, 77%, m.p. 173-175 °C; ^{1}H NMR (400 MHz, $CDCl_3$) δ 7.55 (s, 1H, ArH), 7.39 (s, 1H, ArH), 7.20-7.16 (m, 1H, ArH), 7.11 (t, *J* = 7.6 Hz, 3H, ArH), 6.95 (d, *J* = 8.0 Hz, 1H, ArH), 6.61 (d, *J* = 7.6 Hz, 2H, ArH), 6.56 (d, *J* = 8.0 Hz, 1H, ArH), 6.25 (d, *J* = 8.0 Hz, 1H, ArH), 5.15 (d, *J* = 16.0 Hz, 1H, CH_2), 4.63 (q, *J* = 7.2 Hz, 2H, CH_2), 4.20 (d, *J* = 16.4 Hz, 1H, CH_2), 2.97 (s, 3H, CH_3), 2.27 (s, 3H, CH_3), 2.14 (s, 3H, CH_3), 1.53 (t, *J* = 7.2 Hz, 3H, CH_3). ^{13}C NMR (100 MHz, $CDCl_3$) δ 174.2, 173.1, 171.1, 142.2, 141.5, 134.4, 133.3, 132.9, 132.1, 130.6, 128.6, 128.0, 127.8, 127.4, 126.2, 121.9, 120.6, 114.7, 109.3, 108.2, 89.1, 68.8, 62.6, 60.2, 43.7, 26.3, 21.0, 20.9, 14.7. IR (KBr) υ: 3467, 3063, 3035, 2990, 2919, 2205, 1739, 1706, 1632, 1602, 1496, 1454, 1434, 1408, 1381, 1361, 1333, 1293, 1258, 1215, 1199, 1186, 1167, 1133, 1089, 1070, 1026, 997, 962, 933, 911, 895, 875, 836, 818, 776, 747 cm^{-1}; MS (*m*/*z*): HRMS (ESI) Calcd. for $C_{31}H_{27}NaN_3O_4$ ($[M+Na]^+$): 528.1899, found: 528.1896.

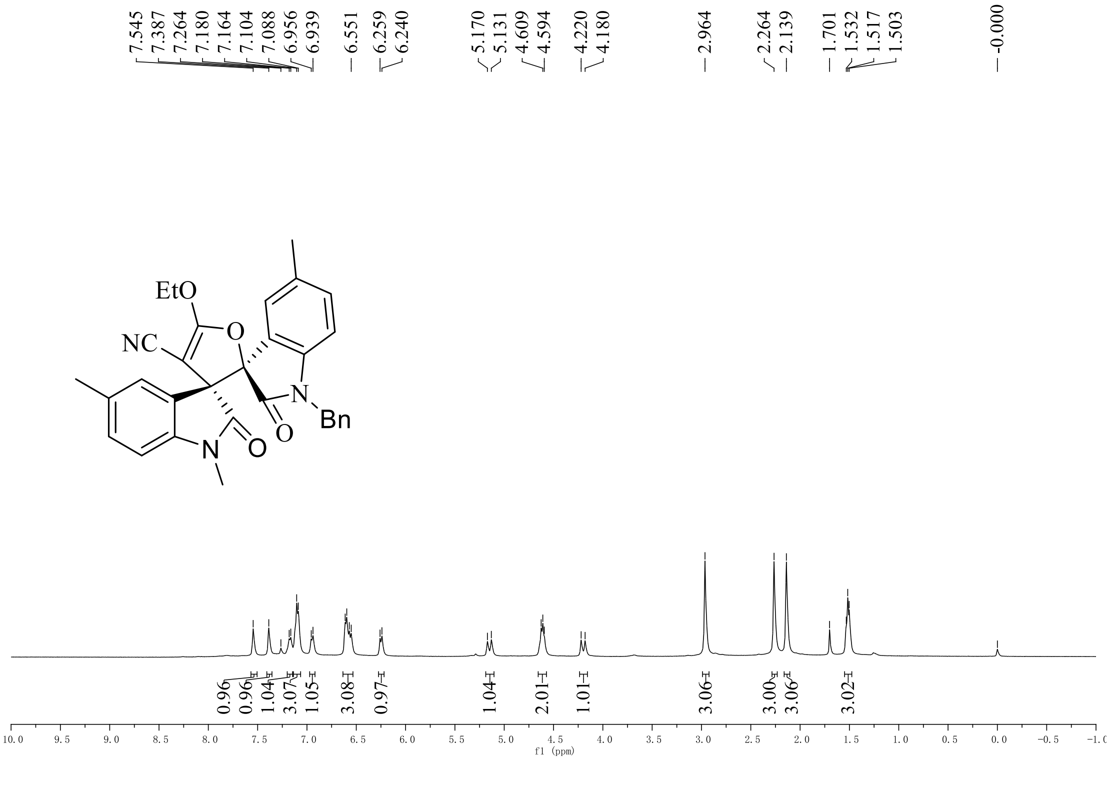

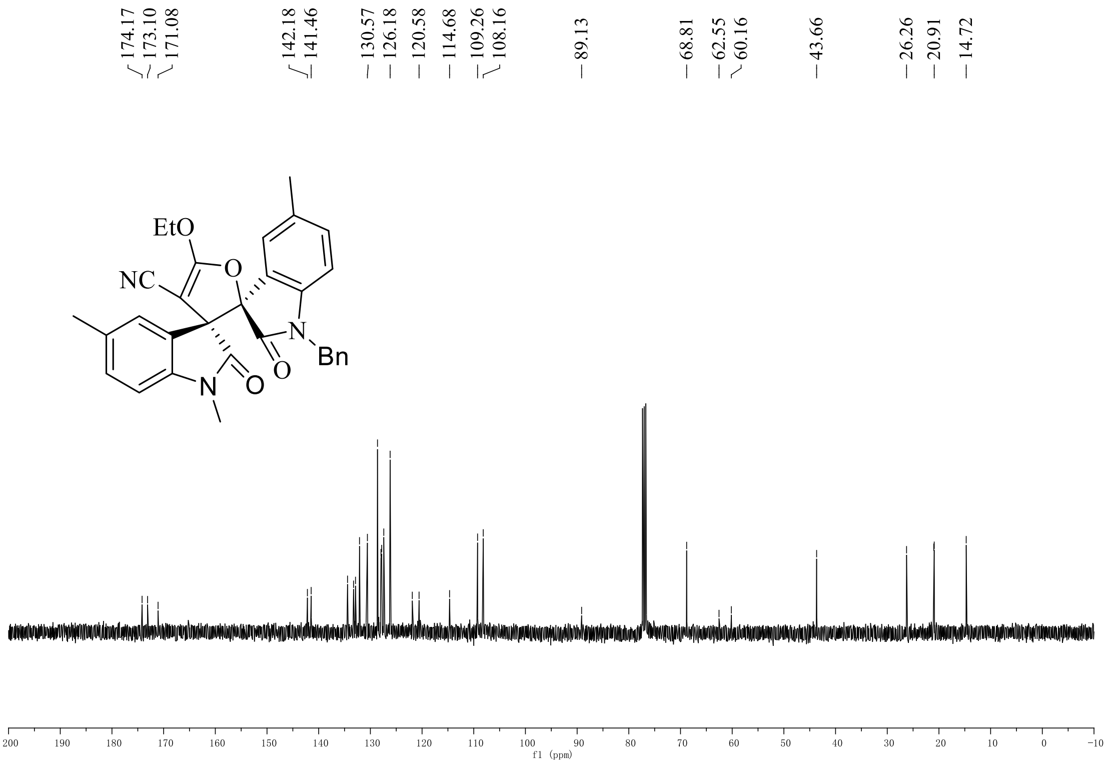
174.17
173.10
171.08
142.18
141.46
130.57
126.18
120.58
114.68
109.26
108.16
89.13
68.81
62.55
60.16
43.66
26.26
20.91
14.72
EtO
O
NC
N
Bn
O
N
O
200
190
180
170
160
150
140
130
120
110
100
90
80
70
60
50
40
30
20
10
0
-10
f1 (ppm)

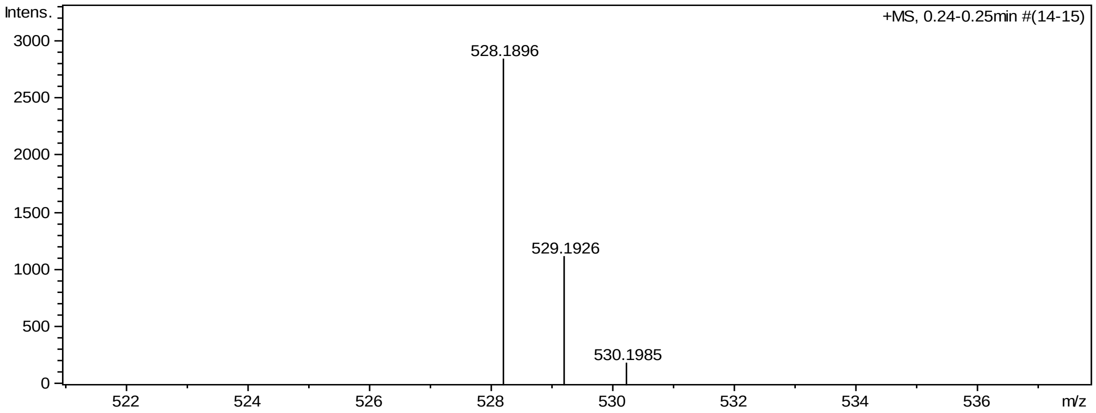
Intens.
+MS, 0.24-0.25min #(14-15)
528.1896
529.1926
530.1985
3000
2500
2000
1500
1000
500
0
522
524
526
528
530
532
534
536
m/z

***rel*-(3*R*,3'*R*)-1,1''-Dibenzyl-5'-ethoxy-5-methyl-2,2''-dioxodispiro[indoline-3,2'-furan-3',3''-indoline]-4'-carbonitrile (8l)**: white solid, 83%, m.p. 141-143 °C; ^{1}H NMR (400 MHz, $CDCl_3$) δ 7.61 (d, *J* = 7.6 Hz, 1H, ArH), 7.54 (s, 1H, ArH), 7.16-7.09 (m, 3H, ArH), 7.04-6.93 (m, 6H, ArH), 6.55 (d, *J* = 7.6 Hz, 4H, ArH), 6.44 (d, *J* = 8.0 Hz, 1H, ArH), 6.29 (d, *J* = 8.0 Hz, 1H, ArH), 5.20 (d, *J* = 16.0 Hz, 1H, CH_2), 5.04 (d, *J* = 16.0 Hz, 1H, CH_2), 4.65 (q, *J* = 6.8 Hz, 2H, CH_2), 4.33 (d, *J* = 16.0 Hz, 1H, CH_2), 4.28 (d, *J* = 16.4 Hz, 1H, CH_2), 2.10 (s, 3H, CH_3) 1.54 (t, *J* = 6.8 Hz, 3H, CH_3).^{13}C NMR (100 MHz, $CDCl_3$) δ 174.5, 173.1, 171.2, 144.0, 141.4, 134.6, 134.2, 133.4, 132.0, 130.3, 128.7, 128.6, 128.5, 127.4, 127.2, 126.4, 126.0, 123.7, 122.2, 121.0, 114.5, 109.6, 109.5, 89.3, 68.9, 62.9, 60.3, 43.8, 43.7, 20.9, 14.8.IR (KBr) υ: 3675, 3032, 2984, 2920, 2859, 2361, 2206, 1724, 1637, 1496, 1467, 1438, 1408, 1376, 1335, 1214, 1166, 1094, 1077, 1053, 1029, 994, 927, 902, 874, 848, 816, 779, 760, 737, 700 cm^{-1}; MS (*m*/*z*): HRMS (ESI) Calcd. for $C_{36}H_{29}NaN_3O_4$ ([M+Na]$^+$): 590.2056, found: 590.2047.

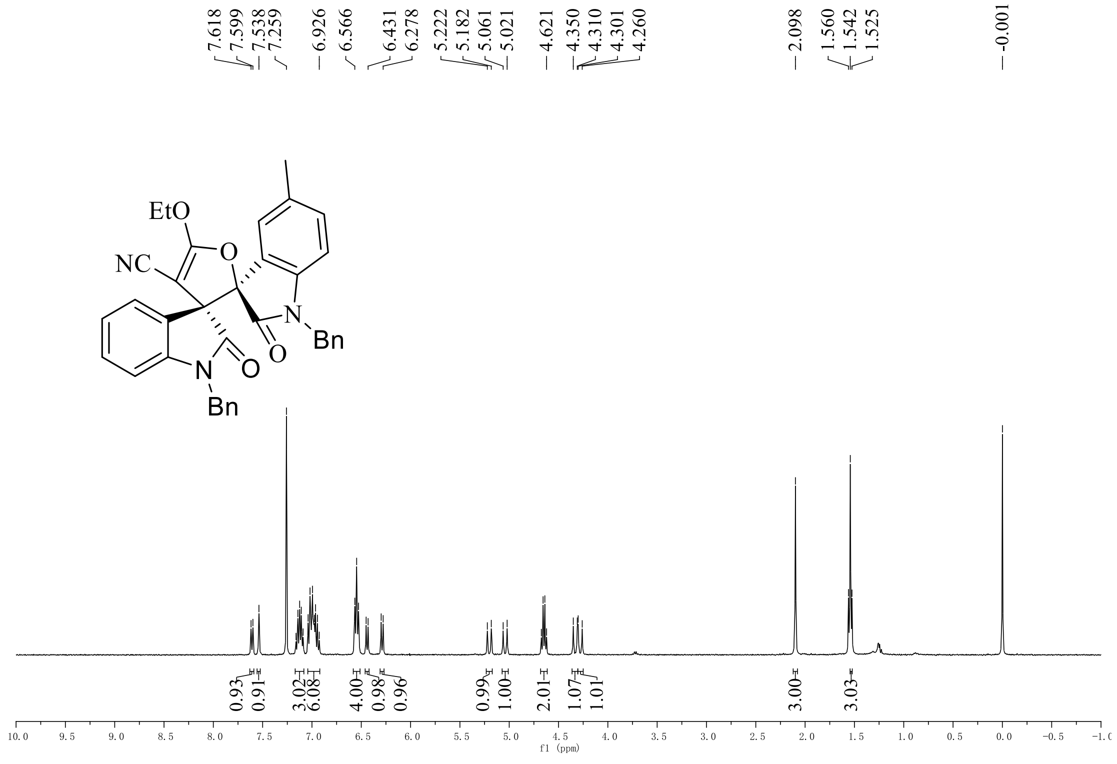

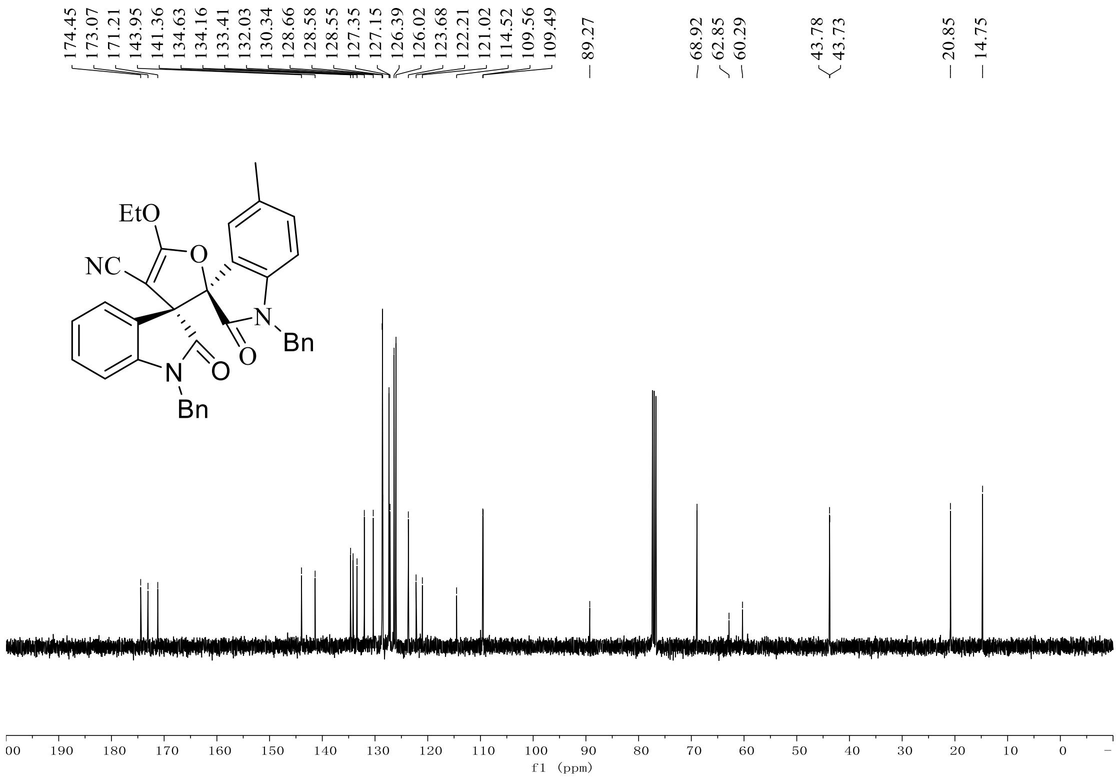
174.45
173.07
171.21
143.95
141.36
134.63
134.16
133.41
132.03
130.34
128.66
128.58
128.55
127.35
127.15
126.39
126.02
123.68
122.21
121.02
114.52
109.56
109.49
89.27
68.92
62.85
60.29
43.78
43.73
20.85
14.75
EtO
NC
O
N
Bn
O
N
O
Bn
f1 (ppm)

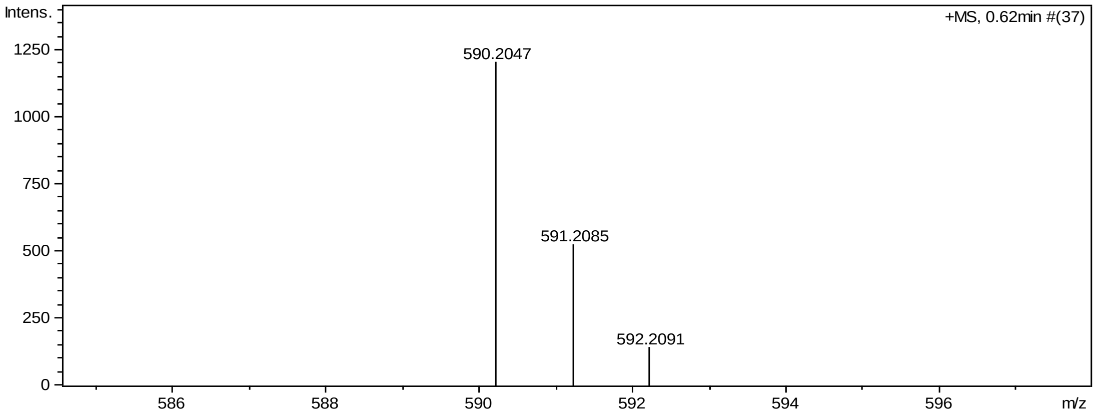
Intens.
+MS, 0.62min #(37)
590.2047
591.2085
592.2091
m/z

***rel*-(3*R*,3'*R*)-1''-Benzyl-5'-ethoxy-1,5-dimethyl-2,2''-dioxodispiro[indoline-3,2'-furan-3',3''-indoline]-4'-carbonitrile (8m)**: white solid, 62%, m.p. 174-175 °C; ^{1}H NMR (400 MHz, $CDCl_3$) δ 7.57 (d, *J* = 7.6 Hz, 1H, ArH), 7.53 (s, 1H, ArH), 7.19-7.15 (m, 2H, ArH), 7.11-7.08 (m, 3H, ArH), 7.00 (t, *J* = 8.0 Hz, 1H, ArH), 6.62 (d, *J* = 7.2 Hz, 2H, ArH), 6.55 (d, *J* = 8.0 Hz, 1H, ArH), 6.40 (d, *J* = 7.6 Hz, 1H, ArH), 5.21 (d, *J* = 16.0 Hz, 1H, CH_2), 4.62 (q, *J* = 6.8 Hz, 2H, CH_2), 4.32 (d *J* = 16.0 Hz, 1H, CH_2), 2.92 (s, 3H, CH_3), 2.15 (s, 3H, CH_3), 1.52 (t, *J* = 7.2 Hz, 3H, CH_3).^{13}C NMR (100 MHz, $CDCl_3$) δ 174.4, 173.2, 171.2, 143.7, 142.1, 134.8, 133.4, 132.0, 130.3, 128.6, 128.4, 127.3, 126.7, 126.2, 123.2, 121.8, 120.9, 115.5, 109.4, 108.4, 89.1, 68.9, 62.5, 60.6, 43.8, 26.1, 20.9, 14.7. IR (KBr) υ: 3057, 3029, 2987, 2964, 2927, 2360, 2204, 1955, 1731, 1650, 1609, 1499, 1465, 1410, 1380, 1343, 1293, 1242, 1214, 1179, 1136, 1097, 1063, 1006, 937, 906, 858, 815, 755, 739 cm^{-1}; MS (*m/z*): HRMS (ESI) Calcd. for $C_{30}H_{25}NaN_3O_4$ ([M+Na]$^+$): 514.1743, found: 514.1744.

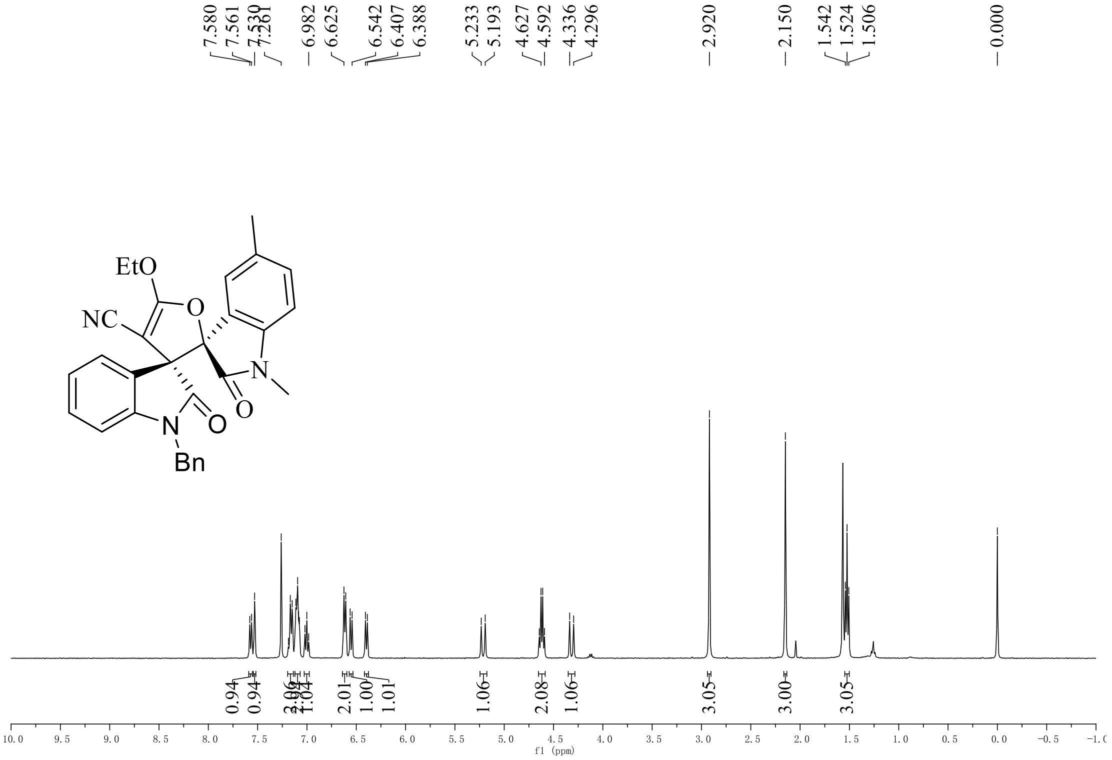

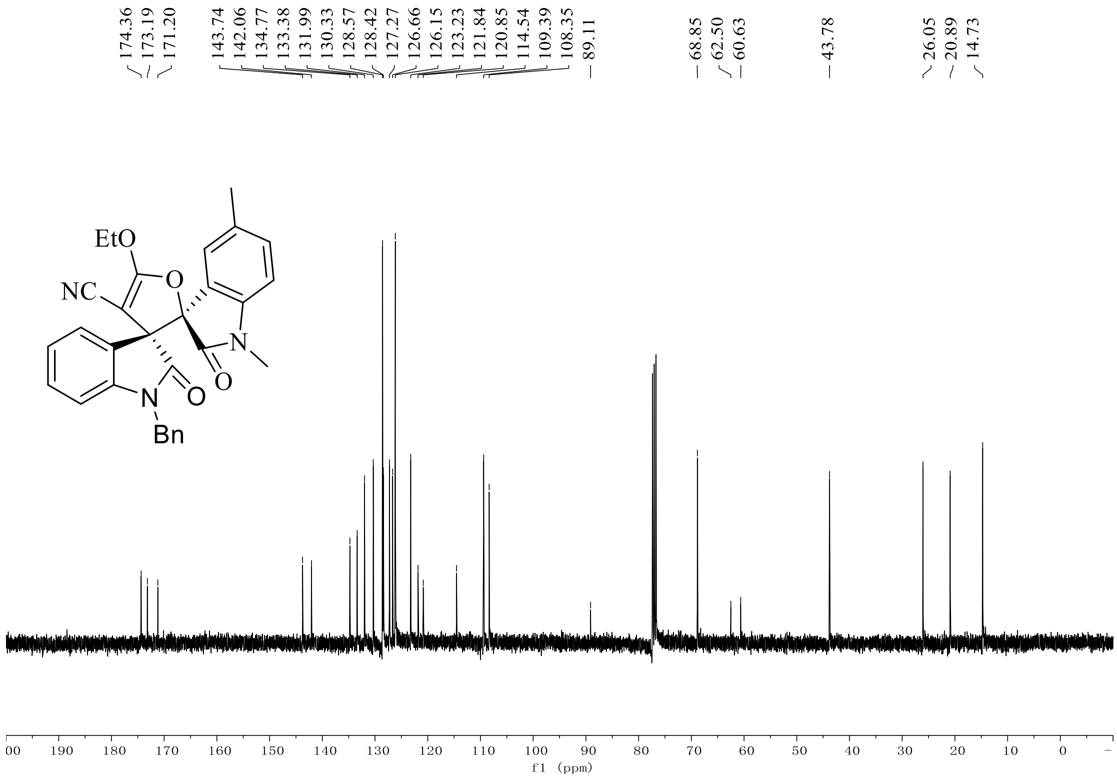
174.36
173.19
171.20
143.74
142.06
134.77
133.38
131.99
130.33
128.57
128.42
127.27
126.66
126.15
123.23
121.84
120.85
114.54
109.39
108.35
89.11
68.85
62.50
60.63
43.78
26.05
20.89
14.73
EtO
NC
O
N
O
N
O
Bn
f1 (ppm)

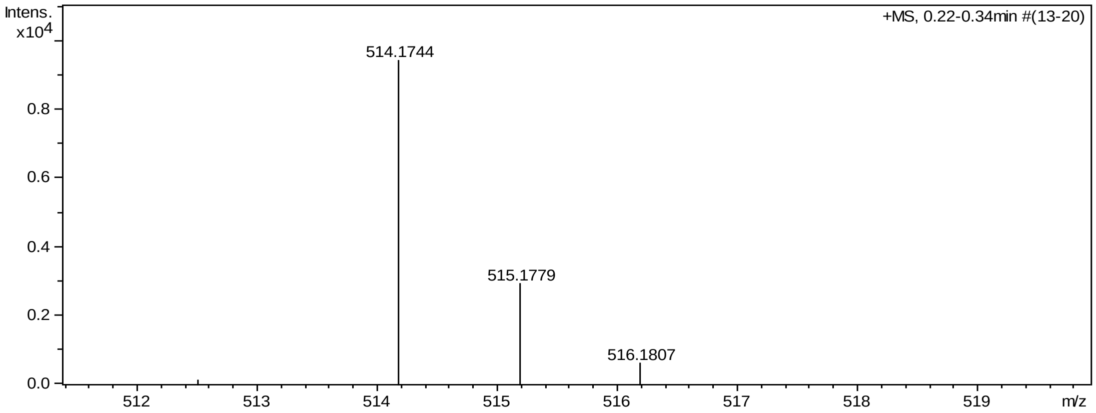
Intens.
x10^4
+MS, 0.22-0.34min #(13-20)
514.1744
515.1779
516.1807
m/z
